# *Nigella sativa*: A Dietary Supplement as an Immune-Modulator on the Basis of Bioactive Components

**DOI:** 10.3389/fnut.2021.722813

**Published:** 2021-08-17

**Authors:** Yun Niu, Baoguang Wang, Li Zhou, Changyang Ma, Geoffrey I. N. Waterhouse, Zhenhua Liu, Adel F. Ahmed, Dongxiao Sun-Waterhouse, Wenyi Kang

**Affiliations:** ^1^National R&D Center for Edible Fungus Processing Technology, Henan University, Kaifeng, China; ^2^Functional Food Engineering Technology Research Center, Kaifeng, China; ^3^Joint International Research Laboratory of Food and Medicine Resource Function, Kaifeng, China; ^4^School of Chemical Sciences, University of Auckland, Auckland, New Zealand; ^5^Medicinal and Aromatic Plants Researches Department, Agricultural Research Center, Horticulture Research Institute, Giza, Egypt

**Keywords:** *Nigella sativa*, monoterpene glucoside, monosaccharide derivatives, immune, anti-inflammatory, RAW264.7

## Abstract

Nutrients can be considered as functional foods, which exert physiological benefits on immune system. The seeds of *Nigella sativa*, which have many active constituents, are mainly used for medicine, food spice, and nutritional supplements in Egypt. Much attention has been paid to *N. sativa* seeds for their anticancer, antibacterial, anti-inflammatory, and immune properties. However, their active constituents and mechanisms underlying functions from *N. sativa* seeds is unclear. Thus, the bioactive constituents with immune regulation in *N. sativa* seeds were systematically studied. A new compound (3-methoxythymol-6-*O*-β-D-apiofuranosyl-(1→6)-β-D-glucopyranoside **1**) and 11 known compounds (**2–12**) were separated from the *N. sativa* seeds by chromatographic methods. Their structures were then elucidated by spectroscopic analysis of MS, UV, IR, ^1^H-, and ^13^C-NMR. Furthermore, immunomodulatory effects of those compounds in RAW 264.7 cells were evaluated by phagocytosis, nitric oxide (NO) and cytokine release, related mRNA transcription, and key proteins expression *in vitro*. Monosaccharide derivatives, Ethyl-α-D-furaarabinose (**5**), and Ethyl-β-D-fructofuranoside (**8**) were shown to played bidirectional regulatory roles in immunity and anti-inflammation through the regulation of nuclear factor-κB (NF-κB) signaling pathways. The results showed the active compounds and mechanisms of immune regulation in *N. sativa*, thus indicating that *N. sativa* seeds could be used as dietary supplements in immunomodulation.

## Introduction

The interaction between inflammation and the immune system is very complex (1). Inflammation is a physiological and pathological reaction caused by the harmful stimulation of living tissue with the vascular system by the internal and external environment (1). A moderate inflammatory reaction can stimulate the immune system and promote the proliferation and activation of immune cells, while an excessive inflammatory reaction will cause immune system dysfunction, resulting in damage to the body (2). Inflammation is the outcome of effective immune response actions that prevent the organism from infections (3). Acute inflammation is the response of the immune system microorganism and environmental stresses and is crucial to tissue healing. On the other hand, chronic inflammation generally refers to chronic illnesses like diabetes and neurodegenerative, cardiovascular, and metabolic diseases (4, 5). When chronic inflammation develops, it can cause pathological change to signaling pathways [especially nuclear factor-κB (NF-κB)] and the signal transducer, which cause an increased level of oxidative stress leading to excessive release of reactive oxygen species (ROS) (6, 7). NF-κB pathway activation leads to the expression of genes regulating immune-regulation, inflammation, apoptosis, and carcinogenesis, with the release of pro-inflammatory cytokines and chemokines (8). Immunotherapy is an effective therapeutics *via* activating or suppressing the immunologic system through synthetic, natural drugs and antibodies to combat disorders (5). Intake of immune modulators is an essential approach to immunotherapy. Synthetic agents show typical side effects manifested as infection, blood constipation, disorders, and so on, while natural drugs are comparatively safe (9). Some dietary constituents of phytochemicals are essential to the balance and development of the immune system and in the amelioration of chronic inflammation (10).

Diet therapy and diet health gradually became the focus of attention. Diets rich in plants such as spices, fruits, and vegetables were demonstrated to restrict the emergence and growth of chronic illness *via* the inhibition of chronic inflammation (11, 12). Balanced healthy diets along with nutrient supplementation are also essential to maintain the normal physiology of the human body as it plays an essential role in boosting up individual immunity (13). Nutrient supplementation containing botanicals can meet the physiological and nutritional needs of some patients (14). The use of botanicals has drawn much attention, particularly for minimizing adverse events on the immune system (15).

*Nigella sativa*, belonging to the family Ranunculaceae, is distributed in southwest Asia, North Africa, and Southern Europe (16). *N. sativa* seeds are exhibited in time-honored traditions through their usage as wind dispelling agents, diuretics, insect repellents, and dietary supplements (17–19). The seeds of *N. sativa* have been shown to exert anti-cancer, immunomodulative, anti-inflammatory, anti-bacterial, antioxidant, hypoglycemic, stomach protection, liver protection, and renal protection activities (20–22). The seeds and oil of *N. sativa* are widely used in food preparation and medicine (23, 24). In addition, literature has shown that the co-delivery of nutrient supplements and drugs could contribute to promising results. *N. sativa* components as nutritional supplements are effective for the adjuvant treatment of COVID-19 cases (25, 26). In addition, *N. sativa* seeds and oil has been shown to supplement cardiovascular protective functions for patients with type 2 diabetes mellitus (T2DM) (27). Like other supplements, *N. sativa* seeds can strengthen the resistance of the immune system to diseases (28). Alshatwi A A found that *N. sativa* seed extract could stimulate the proliferation of human peripheral blood monocytes, which are usually stimulated by non-phytohemagglutinin through flow cytometry and PCR methods (29).

It is obvious that *N. sativa* seeds could meet the nutritional need to improve the immune system of an organism (30). Research on the chemical constituents of this genus began in the middle of the 20th century (31), which showed that *N. sativa* seeds contain various chemical components like essential oils, alkaloids, phenols, saponins, and steroids (32, 33).

**Figure 1:**
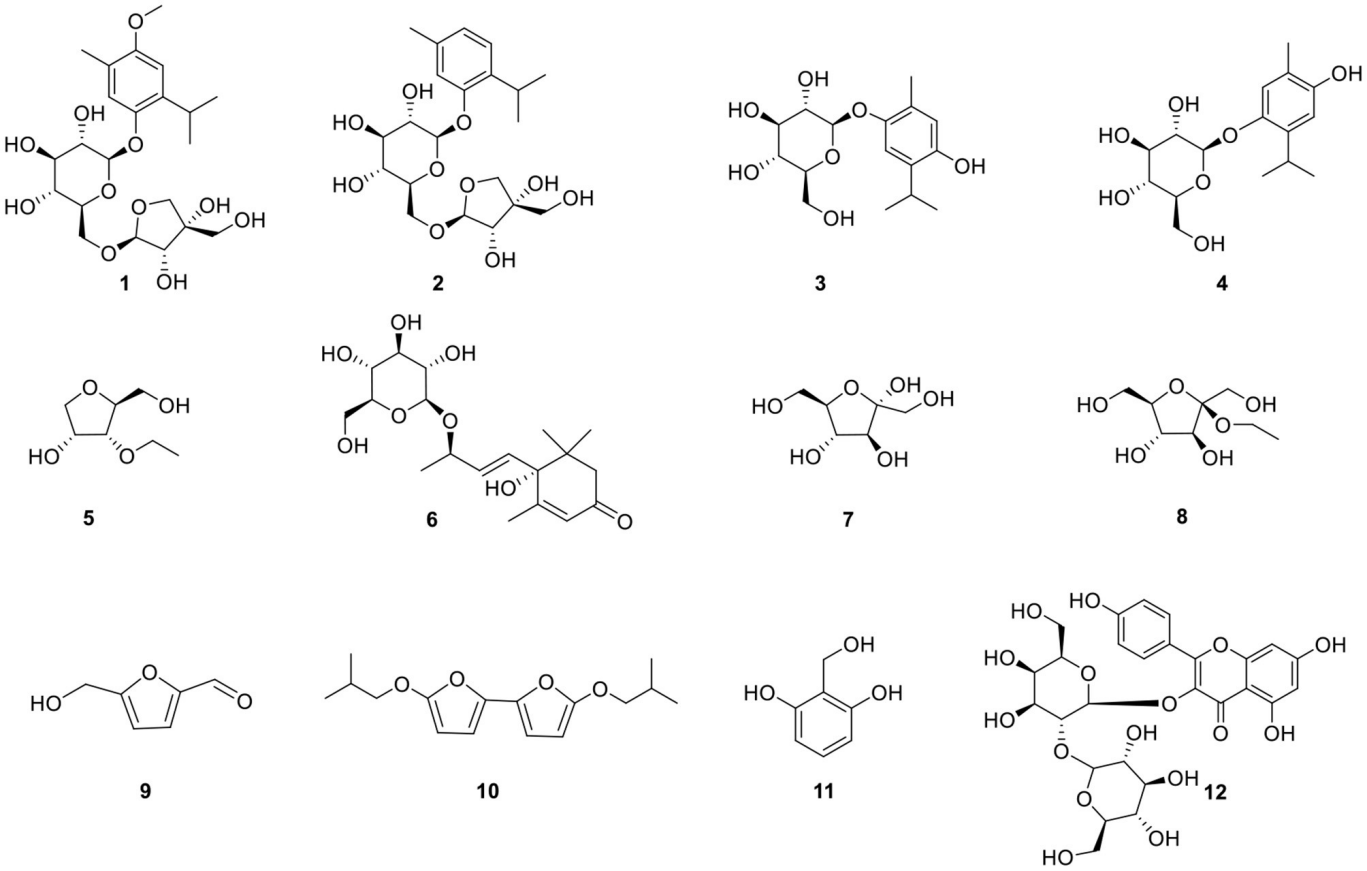
Structures of compounds isolated from *N. sativa*.

Our work focused on the identification of the relevant bioactive compounds in *N. sativa* seeds. The ethanol extract of *N. sativa* was investigated and a new monoterpene glucoside, 3-methoxythymol-6-*O*-β-D-apiofuranosyl-(1→6)-β-D-glucopyranoside, and 11 known compounds were isolated and identified. Then, the anti-inflammatory and immunomodulative activities of the compounds (Figure 1) isolated from *N. sativa* seeds were evaluated *in vitro*. The mechanism of the monosaccharide derivatives was demonstrated on RAW264.7 macrophage.

## Materials and Methods

### Reagents and Instruments

The following technologies and materials were used in this research: UV spectra (Shimadzu, Kyoto, Japan); IR spectra (Bruker Vector 22 spectrophotometer, Bruker Optics GmbH, Ettlingen, Germany); Mass spectra (API QSTAR time-of-flight spectrometer, MDS Sciqaszex, Concord, Ontario, Canada). NMR spectra (Bruker AM-400, Bremerhaven, Germany); silica gel (200–300 and 300–400 mesh, Qingdao Marine Chemical Inc., China); Rp-18 gel (40–63 μm, Merck, Darmstadt, Germany); Sephadex LH-20 (20–150 μm, Amersham Biosciences, Uppsala, Sweden); YMC*GEL ODS-A-HG (50 μm, YMC Co. Ltd., Kyoto, Japan) (34).

The following reagents were used in this research: FBS (Gibco, Grand Island, NE, USA); DMEM, neutral red (Solarbio, Beijing, China); Nitric oxide kit (Nanjing Jiancheng Bioengineering Institute); IL-6 and TNF-α ELISA kit (Beijing 4A Biotech Co., Ltd., Beijing, China); Primer iNOS, TNF-α, IL-6 and Cox-2 (Thermo Fisher Scientific, Shanghai, China); Reactive Oxygen Species Assay Kit (Beyotime Biotechnology, Shanghai, China); PrimeScriptTMRT reagent kit with gDNA Eraser kit and TB Green TM Ex TaqTM II (Tli RNadeH Plus, Accurate Biology, Hunan, China), Bulk kit (TaKaRa, Accurate Biology, Hunan, China); Antibody NF-κB p65, phospho-NF-κB p65, iNOS, COX-2, IκBα, and phospho-IκBα (Cell Signaling, Beverly, MA, USA); LPS (Sigma-Aldrich, St. Louis, MO, USA) (35).

### Extraction and Isolation

The air-dried and powdered *N. sativa* seeds (5 kg) were degreased with petroleum ether and the residue was extracted by 70% ethanol (3 ×10 L) under room temperature. The ethanol extract (550 g) was separated by silica gel column chromatography eluting with a gradient of CHCl_3_-MeOH (20:1→1:1, v/v) to afford seven fractions 1–7 by TLC plate analysis. Fr.2 (15 g) was subjected to Rp-18 column chromatography (MeOH-H_2_O, 20:80→100:0, v/v) to afford five subfractions (Fr.2-1 to Fr.2-6). Sephadex LH-20 column chromatography (MeOH) was performed on 2–4 (300 mg) and then purified by semi-prep. HPLC (MeOH-H_2_O, 40:60, v/v) was performed to obtain **3** (4 mg), **4** (4 mg), and **5** (25 mg). Fr.3 (50 g) was then separated by Rp-18 column chromatography (MeOH-H_2_O, 40:90→60:0, v/v), performed by silica gel column chromatography, eluted with a gradient system of EAC-MeOH (10:1→1:1, v/v), purified by Sephadex LH-20 column chromatography (MeOH), and further purified by semi-prep. Another HPLC (MeOH-H_2_O, 30:70, v/v) was also performed to afford **6** (4 mg), **7** (3.5 mg), and **8** (35 mg). Fraction 4 (19.5 g) was subjected to silica gel column chromatography, eluting with a gradient system of CHCl_3_-MeOH (8:1→1:1, v/v) to afford five subfractions (Fr.4-1 to Fr.4-5). Fr.4-2 (15 g) was performed by Rp-18 column chromatography (MeOH-H_2_O, 10:90→100:0, v/v), Sephadex LH-20 column chromatography (MeOH), and further purified by semi-prep. An additional HPLC (MeOH-H_2_O, 52:48, v/v) to afford **1** (4 mg) and **2** (3 mg) was performed. Fr.6 (100 g) was separated by silica gel column chromatography, eluting with a gradient system of CHCl_3_-MeOH (8:1→1:1, v/v) to afford five subfractions (Fr.6-1 to Fr.6-5). Fr.6-1 (300 mg) was purified by semi-prep. Once again, HPLC was performed (MeOH-H_2_O, 28:82, v/v) to afford **9** (7 mg) and **10** (3 mg). Fr.6-3 (150 mg) was purified by Sephadex LH-20 column chromatography (MeOH), further by semi-prep. Another HPLC (MeOH-H_2_O, 15:85, v/v) to afford **11** (2 mg) was performed. Fr.7 (10 g) was separated by silica gel column chromatography, eluting with a gradient system of CHCl_3_-MeOH (5:1→1:1, v/v), then Sephadex LH-20 column chromatography (MeOH), and further purified by semi-prep. One more HPLC (MeOH-H_2_O, 52:48, v/v) was performed to afford **12** (5 mg). Spectral data was seen in Supplementary Materials.

### Plant Material

*Nigella sativa* seeds were provided by the Department of Medicinal and Aromatic Plants, Horticultural Research Institute, Egyptian Agricultural Research Center. The plants of *N. sativa* were rich in local resources; thus, the collection was permitted.

### Cell Culture and Cell Viability Assay

RAW264.7 macrophages were cultured by the same assay as Zhang H (36). Cell viability was assessed by 3-(4,5-Dimethylthiazol-2-yl)-2,5-diphenyltetrazolium bromide (MTT) assay. The starting inoculum of 1 × 10^5^ cells/ml/well was cultured in a 96-well cell culture plate at 100 μl per well-incubating at 37°C with 5% carbon dioxide in an incubator for 24 h. Cells were also exposed to 100-μl culture mediums with different concentrations (6.25, 12.5, 25, 50, 100, 200, and 400 μmol/L) of compounds **5** and **8** for 24 h, respectively. Lipopolysaccharide (LPS, 1 μg/ml) was used as a positive control. In addition, 10 μl/well MTT solution was added in the dark environment, cells were cultured in an incubator for 4 h, and dimethyl sulfoxide (DMSO, 100 μl) was added to each well to solubilize the blue-purple crystal, with the best absorption at 490 nm.

### Phagocytic Activity

RAW264.7 cells were seeded in 96-well plates (1 × 10^6^ cells/ml) and exposed to specified concentrations of compounds **5** and **8**. The supernatant was treated with 0.075% neutral red solution after 24 h. Cell lysis solution containing 1% acetic acid-anhydrous alcohol (1:1, V:V) was also added into each well, and then the absorbance value was determined at 540 nm.

### Measurement of NO

The experiment referred to normal and LPS-induced conditions of the RAW264.7 macrophages model. RAW264.7 cells were seeded in 24-well plates (1 × 10^6^ cells/ml) and treated with compounds **5** and **8** for 24 h under normal culture conditions. The administration group was pretreated with compounds **5** and **8** for 1 h and LPS (1 μg/ml) for 24 h under the LPS-induced inflammation model. Then, supernatants were collected for testing of the nitric oxide (NO) concentration by the Nitric oxide kit according to the instructions of the manufacturer.

### Determination of IL-6 and TNF-α

Macrophages were treated as described above, cellular supernatants were collected, and the cytokines [interleukin- (IL-) 6 and tumor necrosis factor- (TNF-) α] were analyzed using corresponding ELISA kits.

### qRT-PCR

Cell processing was done similarly to the above methods, and PCR was performed by the same assay as Zhang H (36). The sequences are listed in Table 1.

**Table 1:** Primers sequences.

**Name**	**Primer**	**Sequence (5'→3')**
COX-2	Forward	GGGCTCAGCCAGGCAGCAAAT
	Reverse	GCACTGTGTTTGGGGTGGGCT
iNOS	Forward	GCTCGCTTTGCCACGGACGA
	Reverse	AAGGCAGCGGGCACATGCAA
IL-6	Forward	AGACAAAGCCAGAGTCCTTCAGAGA
	Reverse	GCCACTCCTTCTGTGACTCCAGC
TNF-α	Forward	CCCTCCTGGCCAACGGCATG
	Reverse	TCGGGGCAGCCTTGTCCCTT
GAPDH	Forward	ACCCCAGCAAGGACACTGAGCAAG
	Reverse	GGCCCCTCCTGTTATTATGGGGGT

### Western Blot

RAW264.7 cells were collected as described above, and then the total proteins were extracted using the weak RIPA Lysis Buffer. The methods of sodium dodecyl sulfate-polyacrylamide gel electrophoresis (SDS-PAGE) and Western blot were the same as the assay done by Wang Honglin (35). The protein signals were visualized with an ECL chemiluminescence detection kit (Solarbio, Beijing, China) and the band gray value was quantitatively analyzed using the Image J software (36).

### Determination of Intracellular ROS

RAW264.7 cells were plated in 6-well plates with 5 × 10^5^ cells/ml cells suspension and cultured at 37°C with 5% CO_2_ for 24 h. The control group, administration group (compounds **5** and **8**), and LPS group (1 μg/ml) were conducted on RAW 264.7 cells, respectively. The supernatant was discarded after 24 h, and the cells were gently rinsed and centrifuged by adding serum-free Dulbecco's modification of eagle's medium (DMEM). Each group was added with 1 ml of 10 μM DCFH-DA to resuspension cells. Cells were incubated at 37°C for 20 min and reversed every 3–5 min to allow the probe to have full contact with the cells. Eventually, the probe was determined by flow cytometry after using a 300-mesh nylon screen.

### Statistical Analysis

Experimental data were expressed as mean ± standard deviation, and the numerical statistics were handled using a one-way ANOVA of the SPSS 19.0 software. All column images were made *via* the GraPhPad Prism 6.0 software.

## Results

### Identification of a New Compound

**Figure 2:**
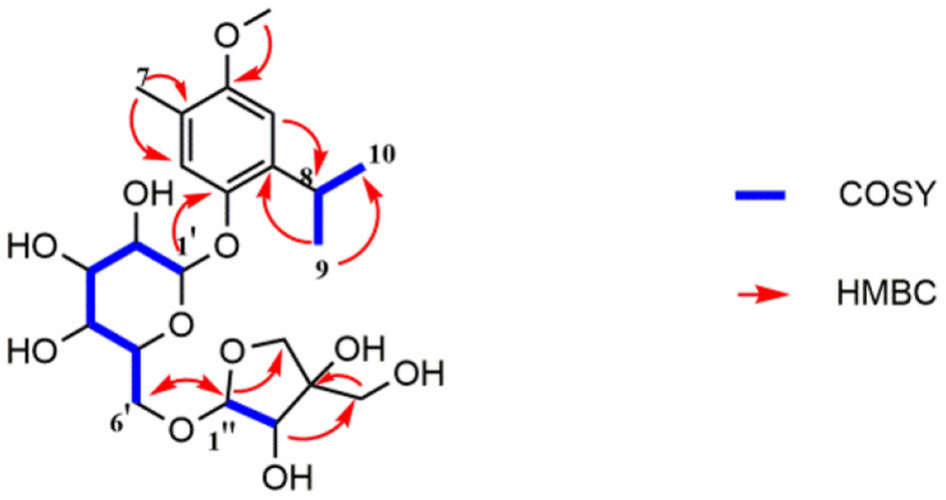
Key ^1^H-^1^H COSY and HMBC correlations of compound **1**.

Compound **1** was obtained as a colorless oil and its formula was determined as C_22_H_34_O_11_ by HR-EI-MS at *m/z* 473.2027 [M-H]^−^ (calcd for C_22_H_34_O_11_, 473.2023). The ^1^H-NMR spectrum (Table 1) indicated 2 aromatic H-atoms at δ_H_ 6.72(s) and 6.95(s), 2 anomeric H-atoms at δ(H) 4.67 (1H, t, *J* = 3.6 Hz) and 4.96 (1H, d, *J* = 2.4 Hz, H-1″), and 3 Me groups at 2.14 (3H, s) and 1.20 (6H, d, *J* = 7 Hz). The ^13^C-NMR spectrum of 4 (Table 2) revealed 22 C-atom signals, corresponding to a 1,3,4,6- substituted aromatic ring, three Me groups at δ(C) 16.2 and 23.6, a CH group at δ(C) 27.4, a β-D-glucosyl moiety, and a terminal β-D-apiosyl moiety (9, 10). In the HMBC spectrum (Figure 2), the key correlations from H-7/C-5, C-4 and C-3, H-2/C-8, H-9/C-1, and C-8 and C-10, together with ^1^H-^1^H-COSY correlations of H-9/H-8 and H-10/H-8 showed that the aglycone of **1** was thymoquinol (11). The key correlations from H-1′ [δ_H_ 4.67] to C-6 (δ_C_ 149.6) in the HMBC spectrum indicated that β-D-glucosyl moiety was attached to C(6). We also speculated that the β-D-apiose moiety was attached to C(6') and the β-D-glucosyl moiety (12). This conclusion was elucidated by the HMBC spectrum, in which the key correlation from H-1″ [δ_H_ 4.96] to C-6′ (δ_C_ 68.7) was observed (Figure 2) (9). Hence, the structure of compound **1** was defined as 3-methoxythymol-6-*O*-β-D-apiofuranosyl-(1→6)-β-D-glucopyranoside.

**Table 2:** The ^1^H and ^13^C NMR data of 1 (CD_3_OD, δ in ppm, *J* in Hz).

**Position**	**δ_**H**_**	**δ_**C**_**
1		138.3
2	6.72 (1H, s)	108.9
3		154.9
4		125.5
5	6.95 (1H, s)	120.8
6		149.6
7	2.14 (3H, s)	16.2
8	3.43 (1H, m)	27.4
9	1.20 (6H, d, *J* = 7 Hz, 3H)	23.7
10	1.20 (6H, d, *J* = 7 Hz, 3H)	23.6
OMe	3.78 (3H, m)	56.2
1′	4.67 (1H, t, *J* = 3.6Hz)	104.6
2′	3.42 (1H, m)	75.2
3′	3.37 (1H, m)	78.2
4′	3.35 (1H, m)	71.6
5′	3.47 (1H, m)	76.8
6′a	3.99 (1H, m)	
6′b	3.55 (1H, m)	68.7
1″	4.96 (1H, d, *J* = 2.4 Hz)	110.9
2″	3.89 (1H, m)	78.1
3″		80.6
4″a	3.91 (1H, s)	
4″b	3.73 (1H, d, *J* = 9.6 Hz)	75.0
5″	3.51 (2H, s)	65.7

### Effects of Compounds 5 and 8 on Cell Viability of RAW264.7 Macrophages

**Figure 3:**
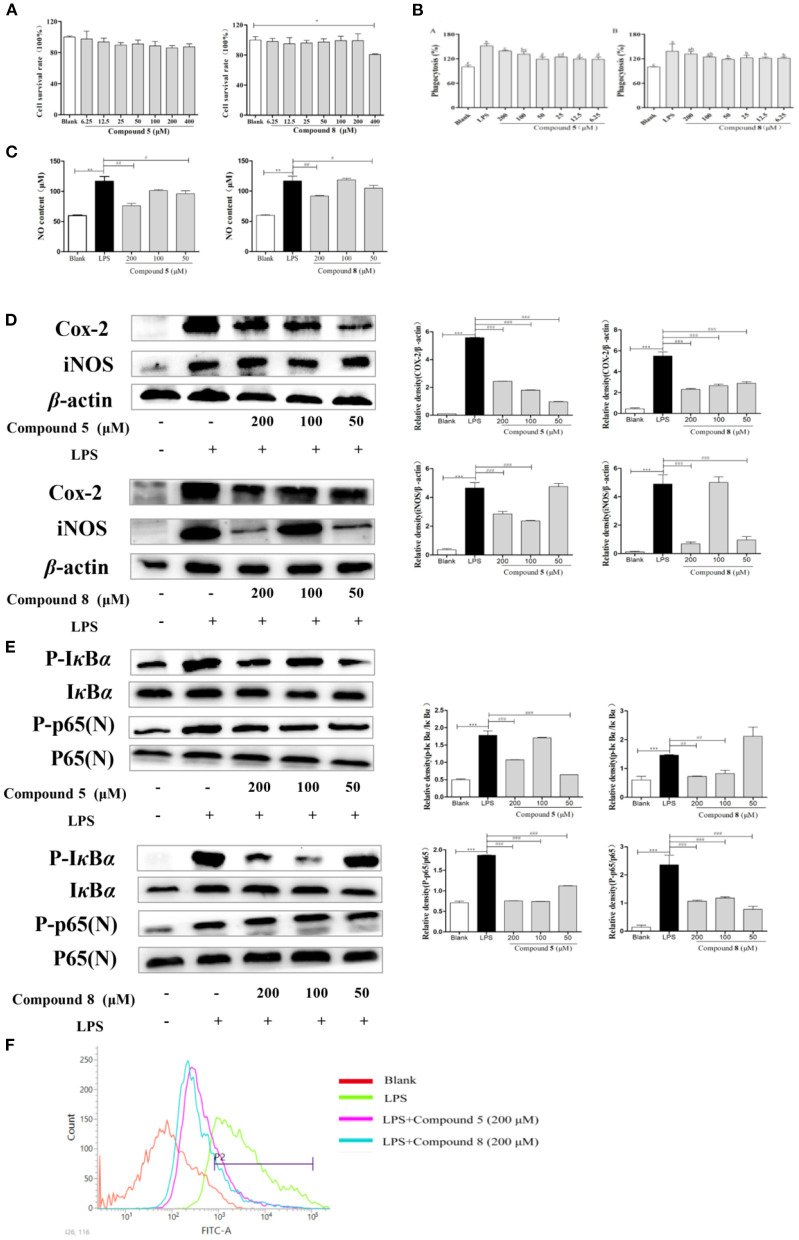
Effects of compounds **5** and **8** on the nuclear factor-κB (NF-κB) pathways in lipopolysaccharide- (LPS) induced RAW264.7 Cells. **(A)** Cell survival rates. **(B)** Phagocytic activity of RAW264.7. **(C)** The expression levels of nitric oxide (NO) production. **(D)** mRNA and protein expression of cyclooxygenase- (COX) 2 and inducible nitric oxide synthase (iNOS). **(E)** Expression levels of key proteins in the NF-κB pathways. **(F)** Production of reactive oxygen species (ROS). β-Actin served as a control. Data shown are means ± SEM. ***p < 0.001, **p < 0.01, *p < 0.05. ^###^*p* < 0.001, ^##^*p* < 0.01, ^#^*p* < 0.05.

RAW264.7 cells were exposed to different concentrations of compounds **5** and **8** for 24 h. Results showed that compound **5** had no significant inhibitory effect on the proliferation of RAW264.7 cells at 6.25–400 μmol/L in Figure 3. Compound **8** did not significantly inhibit the proliferation of RAW264.7 cells at 6.25–200 μmol/L, but significantly inhibited the activity cells at 400 μmol/L. Therefore, the concentrations at 50, 100, and 200 μmol/L of compounds **5** and **8** were selected as the concentration gradients for subsequent experiments in Figure 3A.

### Effects of Compounds 5 and 8 on Phagocytic Activity

In Figure 3, the phagocytosis of LPS-treated cells was consistent with the expectation, which increased nearly twice more than that of the blank group. Compared with the normal control group, the phagocytic activity of RAW264.7 cells was significantly enhanced after being exposed to compounds **5** and **8** at a concentration of 6.25–200 μmol/L for 24 h in Figure 3B. They could promote the phagocytosis of macrophages at a certain concentration range, which may be essential to immune-regulation.

### Effects of Compounds 5 and 8 on NO Secretion in LPS-Induced RAW264.7 Cells

There is a significant increase in the concentration of NO in normal cells after LPS stimulation, increasing to approximately twice as much as that in the blank group, which indicated that the model was successful. However, the excessive expression of NO was inhibited significantly by the high-dose and low-dose groups of compounds **5** and **8** after LPS stimulation, while the middle-dose group showed significant effects in Figure 3C.

### Effects of Compounds 5 and 8 on Gene and Protein Expression of iNOS and COX-2 in LPS-Induced RAW264.7 Cells

The increase of NO during inflammation was generally attributed to the upregulation of inducible nitric oxide synthase (iNOS) and cyclooxygenase- (COX) 2, respectively. In Figure 3D, LPS significantly stimulated the transcription of targeted mRNA, while compounds **5** and **8** reversed the mRNA upregulation. Compounds **5** and **8** at 50, 100, or 200 μmol/L, significantly reduced COX-2 and iNOS protein production in LPS-induced macrophages, which was in accordance with the PCR results.

### Effects of Compounds 5 and 8 on the Expression of Proteins in NF-κB Signaling Pathways in LPS-Induced RAW264.7 Cells

NF-κB signaling pathways are key cellular signaling pathways mediated by LPS. The results showed that the protein expressions of p-IκBα and p-NF-κB p65 were significantly upregulated in RAW264.7 cells stimulated by LPS compared with the blank group. These results indicated that compounds **5** and **8** could inhibit the over-expression of key proteins p-IκBα and N-NF-κB p65 in the NF-κB pathways of RAW264.7 cells in Figure 3E, thereby inhibiting the over-activation of the NF-κB pathways and playing an anti-inflammatory role.

### Effects of Compounds 5 and 8 on LPS-Induced Oxidative Stress

To release abundant ROS, LPS is generally used to stimulate RAW264.7 cells. These ROS mediate inflammatory signals in cells. As is shown in Figure 3F, compared with the blank group, the peak shape of the LPS group shifted significantly to the right, indicating that LPS improved the production of oxygen, while compounds **5** and **8** at 200 μmol/L could inhibit the excessive production of reactive oxygen in stimulated RAW264.7 cells.

### Effects of Compounds 5 and 8 on NO Secretion in Normal Cultured RAW264.7 Cells

**Figure 4:**
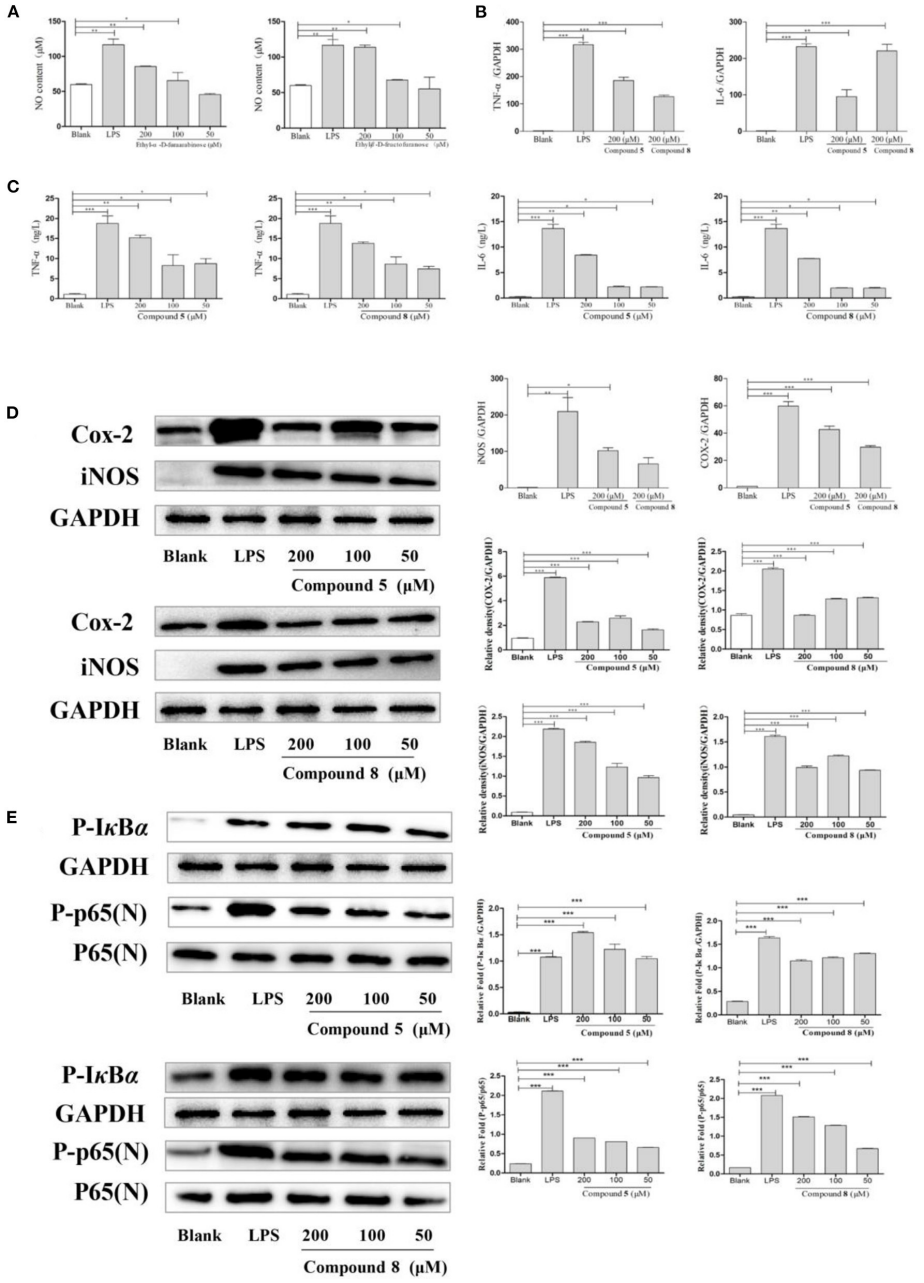
Effects of compounds **5** and **8** on the nuclear factor-κB (NF-κB) signaling pathways in normal cultured RAW264.7 cells. **(A)** Expression of nitric oxide (NO) production. **(B)** Inflammatory cytokine tumor necrosis factor- (TNF-) α, interleukin- (IL-) 6 mRNA transcription. **(C)** Secretion of TNF-α, IL-6. **(D)** mRNA and protein expression of cyclooxygenase- (COX-) 2 and inducible nitric oxide synthase (iNOS). **(E)** Expression of key proteins in the NF-κB signaling pathways. Glyceraldehyde-3-phosphate dehydrogenase (GAPDH) served as control. Data shown are means ± SEM. ***p < 0.001, **p < 0.01, *p < 0.05.

As shown in Figure 4A, compounds **5** and **8** in high- and medium-dose groups significantly promoted the production of NO in normal cultured RAW264.7 cells, while there was no significant effect at 50 μmol/L.

### Effects of Compounds 5 and 8 on the Secretion of Cytokines and mRNA Expression in RAW264.7 Cells in Normal Cultured RAW264.7 Cells

In Figure 4C, compared with the blank group, the release levels of TNF-α and IL-6 by RAW264.7 cells treated with LPS or compounds were significantly increased. The results showed that compounds **5** and **8** could promote the release of TNF-α and IL-6 in RAW264.7 cells, but the optimal dose was 200 μmol/L. On this basis, the effects of compounds **5** and **8** (200 μmol/L, respectively), on the mRNA levels of cytokines in normal cultured RAW264.7 cells were determined by qRT-PCR. As shown in Figure 4B, mRNA transcription of TNF-α and IL-6 was significantly raised following LPS treatment in the positive control group. Furthermore, compared with the control group, compounds **5** and **8** (200 μmol/L, respectively), significantly increased the levels of TNF-α and IL-6. These indicated that compounds **5** and **8** could upregulate the mRNA levels of TNF-α and IL-6 in macrophages and promote the production of IL-6 and TNF-α.

### Effects of Compounds 5 and 8 on iNOS and COX-2 in Normal Cultured RAW264.7 Cells

To determine the effects of compounds **5** and **8** (200 μmol/L, respectively), on the mRNA expression of iNOS and COX-2 in cells, PCR was used. Western blot was applied to measure the effects of compounds **5** and **8** (50, 100, and 200 μmol/L, respectively), on the expression of COX-2 and iNOS under normal culture conditions. As shown in Figure 4D, LPS could significantly increase the production of iNOS and COX-2 mRNA. High-dose groups of compounds **5** and **8** could significantly upregulate the mRNA transcription levels of COX-2 and iNOS in normal cultured cells. Moreover, compared with the blank group, compounds **5** and **8** significantly promoted the production of COX-2 and iNOS proteins. In summary, compounds **5** and **8** could significantly promote the production of COX-2 and iNOS at the gene and protein levels.

### Effects of Compounds 5 and 8 on the Expression of Proteins in NF-κB Signaling Pathway in Normal Cultured RAW264.7 Cells

The NF-κB pathway is crucial to cellular and body immunity, inflammatory response, and apoptosis. As shown in Figure 4E, the results showed that, compared with the blank group, compounds **5** and **8** could promote the expression of key proteins p-IκBα and p-p65 in the NF-κB signaling pathways of RAW264.7 cells in normal culture, thus moderately activating NF-κB signaling pathways and enhancing immunity.

## Discussion

Our immune response system is continuously conducting on protecting the host from microbes by recognizing, answering, and acting on antigens (37). Generally, macrophages are crucial to innate cellular immune responses and play a significant role in the phagocyte cell system, which is programmed to identify, engulf, and eliminate apoptotic cells, bacteria, etc. (10, 38). The immune system functions to maintain the physiological health of the body and protect it from pathogens through an increased release of inflammatory cytokines (39). Cytokines released by different cells with specific effects on cellular signaling through combination to their receptors on the cell surface are essential modulators of the immune process *via* a complex network engaged in various immune processes, which include proliferation, phagocytosis, and inflammation (40, 41).

**Figure 5:**
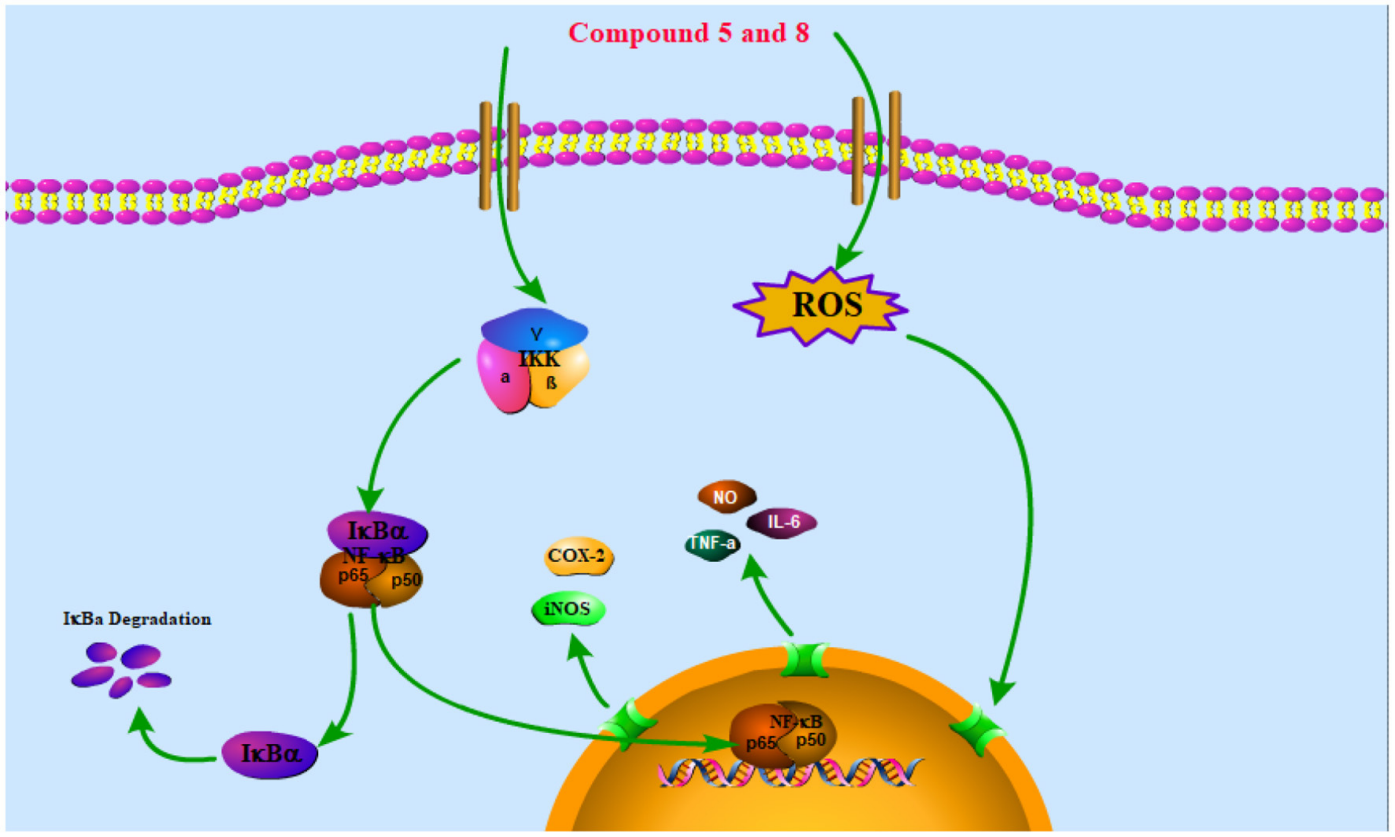
Schematic of the potential mechanisms of the effects of compounds **5** and **8** on the NF-κB signaling pathway in RAW264.7 macrophages. Compounds **5** and **8** exert anti-inflammatory and immune bidirectional regulation by promoting reactive oxygen species (ROS) generation and affecting the nuclear factor-κB (NF-κB) signaling pathway as discussed in the text. The arrows indicate the direction of the signaling pathway.

The NF-κB pathway is crucial to cellular and body immunity, inflammatory response, and apoptosis. The NF-κB pathway activation leads to the expression of genes associated with inflammation and immunoregulation and the release of pro-inflammatory cytokines and chemokines, which eventually results in the transcription of genes (42). It participates in many biological processes such as apoptosis, cellular immune regulation, inflammatory reaction, and tumorigenesis by regulating the expression of related inflammatory factors, chemokines, growth factors, COX-2, and nitric oxide synthase (NOS) (43). Thus, reducing the translocation of NF-κB can decrease the production of pro-inflammatory cytokines and inflammatory mediators like iNOS and COX-2 (44). The NF-κB in the cytoplasm binds to its inhibitor IκB in the resting state to form a trimer p50-p65-IκB, which is in an inactive state. When stimulated by LPS, IκB is phosphorylated by its kinase IKK and under the action of the ubiquitin enzyme, p50-p65-IκB is disintegrated and NF-κB translocation into the nucleus is phosphorylated and activated, which mediates a series of downstream reactions (Figure 5) (45).

In this study, monosaccharide derivatives isolated from *N. sativa* seeds were studied to illustrate their effect on RAW 264.7 macrophage. To evaluate the immunobiological activity of these monosaccharide derivatives, studies based on various immune responses containing cytotoxicity, phagocytosis, transcription, and expression were used. As a result, we found two monosaccharide derivatives **(5**, **8)** (Figure 1) that showed a two-way regulation of immunity and anti-inflammation on normal and LPS-stimulated RAW264.7 macrophages *in vitro* through regulating the expression of key proteins in the NF-κB pathway. Monosaccharide derivatives might be possibly used in immunomodulating on the basis of their function to affect the macrophage. In addition, investigation has also shown that arabinoxylans with immunomodulatory effect could be considered effective bioactive food supplements associated with many health amelioration functions (46). *In vitro* immunobiological evaluation of chitin- and chitosan-derived oligosaccharides on RAW 264.7 cells obtained results that indicated a beneficial immunomodulation effect referring to the development of cytokine release, phagocytosis, cell proliferation, and respiratory burst (10).

Nutrition plays a significant role in every stage of the immune response. In this regard, food containing balanced nutrients with therapeutic functions play an essential role in promoting the immunity of individuals (46). Nutritional interventions, particularly the beneficial constituents with special immune and anti-inflammatory effects, show the potential to regulate and enhance the immune system (47, 48). Since the assessment of the efficacy of plants is laborious, conducting research on more homogeneous populations to take the heterogeneity of the plant preparations into account should be considered in the future (49). Despite the fact that dietary supplements have appealing and considerable interest to nutritionists, the safety and effectiveness of these dietary supplements are still a highly controversial issue (50, 51).

*N. sativa* seeds are quite safe and effective for treating patients with chronic disease (52–55). Thymoquinone obtained in *N. sativa* seeds can reduce oxidative stress and recover the balance between anti- and pro-inflammatory cytokines (56). Another study indicated that supplementing 2 g/day of *N. sativa* oil capsules showed considerable improvements on cardiometabolic parameters of high-density lipoprotein cholesterol (HDL-C), low-density lipoprotein cholesterol (LDL-C), and glutamic-oxaloacetic transaminase (GOT) levels (57) of the serum. However, further research into clinical situations might be required to evaluate the functions of *N. sativa* seeds on the inflammation and immune mechanisms responsible for this behavior. Every supplementation could be considered prudently by the nutritionist and used within the recommended safety dose (58). In addition, monosaccharide derivatives are generally found at low levels in our diet, providing essential contribution by some dietary habits. More growing research with reliable and advanced technologies could be carried out to assess the dietary intake of monosaccharide derivatives in the future.

*N. sativa* seeds were applied as value immune modulators in the therapy of chronic diseases (14). Thus, taking both properties of the compounds and the optimal dose into account, research on *N. sativa* food supplements can provide fundamental information regarding body health suggestions with *N. sativa* in the future, especially against chronic inflammation which leads to some diseases. Our research indicated that an *N. sativa* supplement might be beneficial as a complementary method for therapy of immune and inflammatory complications in patients.

## Conclusion

A new monoterpene glucoside (**1**) and 11 known compounds (**2–12**) were isolated and identified in *N. sativa* seeds. Monosaccharide derivatives **5** and **8** exerted bidirectional regulatory effects on immunity and anti-inflammation through NF-κB signaling pathways. The mechanism of immunity may relate to the increased release of cytokines and the level of mRNA transcription in normal RAW264.7 cells, and anti-inflammatory properties may associate with the inhibition of the excessive release of pro-inflammatory cytokines and excessive transcription at the mRNA level under LPS stimulation. These data will be valuable for further research on *N. sativa* seeds as a dietary supplement for immune-modulation.

## Data Availability Statement

The original contributions presented in the study are included in the article/Supplementary Material, further inquiries can be directed to the corresponding author/s.

## Author Contributions

YN and BW performed the experiments, wrote, and prepared the original draft. LZ analyzed and summarized data. CM and ZL critically reviewed the manuscript. AA contributed to the data acquisition. GW and DS-W supervised project administration. WK provided resources, funding, and reviewed the manuscript. All authors contributed to the article and approved the submitted version.

## Conflict of Interest

The authors declare that the research was conducted in the absence of any commercial or financial relationships that could be construed as a potential conflict of interest.

## Publisher's Note

All claims expressed in this article are solely those of the authors and do not necessarily represent those of their affiliated organizations, or those of the publisher, the editors and the reviewers. Any product that may be evaluated in this article, or claim that may be made by its manufacturer, is not guaranteed or endorsed by the publisher.

## Abbreviations

NO: nitric oxide
COX: cyclooxygenase
IL: interleukin
NF-κB: nuclear factor-κB
gDNA: genomic DNA
PVDF: polyvinylidene fluoride
RNA: ribonucleic acid
PBS: phosphate buffered saline
iNOS: inducible nitric oxide synthase
FBS: fatal bovine serum
PCR: polymerase chain reaction
IκB: inhibitor of NF-κB
LPS: lipopolysaccharide
ELISA: enzyme-linked immunosorbent assay
DMSO: dimethyl sulfoxide
cDNA: complementation deoxyribonucleic acid
BCA: butyleyanoacrylate
GAPDH: glyceraldehyde-3-phosphate dehydrogenase
TNF: tumor necrosis factor
DMEM: Dulbecco's modification of eagle's medium
SDS-PAGE: sodium dodecyl sulfate-polyacrylamide gel electrophoresis
MTT: 3-(4, 5-Dimethylthiazol-2-yl)-2,5-diphenyltetrazolium bromide
COVID-19: coronavirus disease 2019
T2DM: type 2 diabetes mellitus
ROS: reactive oxygen species
HDL-C: high-density lipoprotein cholesterol
LDL-C: low-density lipoprotein cholesterol
GOT: glutamic-oxaloacetic transaminase
HMBC: heteronuclear multiple-bond correlation
HPLC: high-pressure liquid chromatography
TLC: thin layer chromatography.
