# Supplementary material for: Nigella sativa: A Dietary Supplement as an Immune-Modulator on the Basis of Bioactive Components
Source: Front Nutr. 2021 Aug 17;8:722813. doi: 10.3389/fnut.2021.722813 (PMC8415885; doi:10.3389/fnut.2021.722813)
Supplement: Supplementary file 1 [file Data_Sheet_1.docx]

**Supporting Material**

New monoterpene glycoside from *Nigella sativa*

| **Figure S1.** | Mass spectrum of compound **1**. |
| --- | --- |
| **Figure S2.** | ^1^H-NMR spectrum of compound **1** in CD_3_OD. |
| **Figure S3**  **Figure S4** | ^13^C-NMR spectrum of compound **1** in CD_3_OD.  ^1^H-^1^H COSY spectrum of compound **1** in CD_3_OD. |
| **Figure S5.** | HSQC spectrum of compound **1** in CD_3_OD. |
| **Figure S6** | HMBC spectrum of compound **1** in CD_3_OD. |
| **Figure S7**  **Figure S8** | IR spectrum of compound **1** in CD_3_OD.  UV spectrum of compound **1** in CD_3_OD. |

**Figure S1.** High resolution ESI mass spectrum of compound **1**.


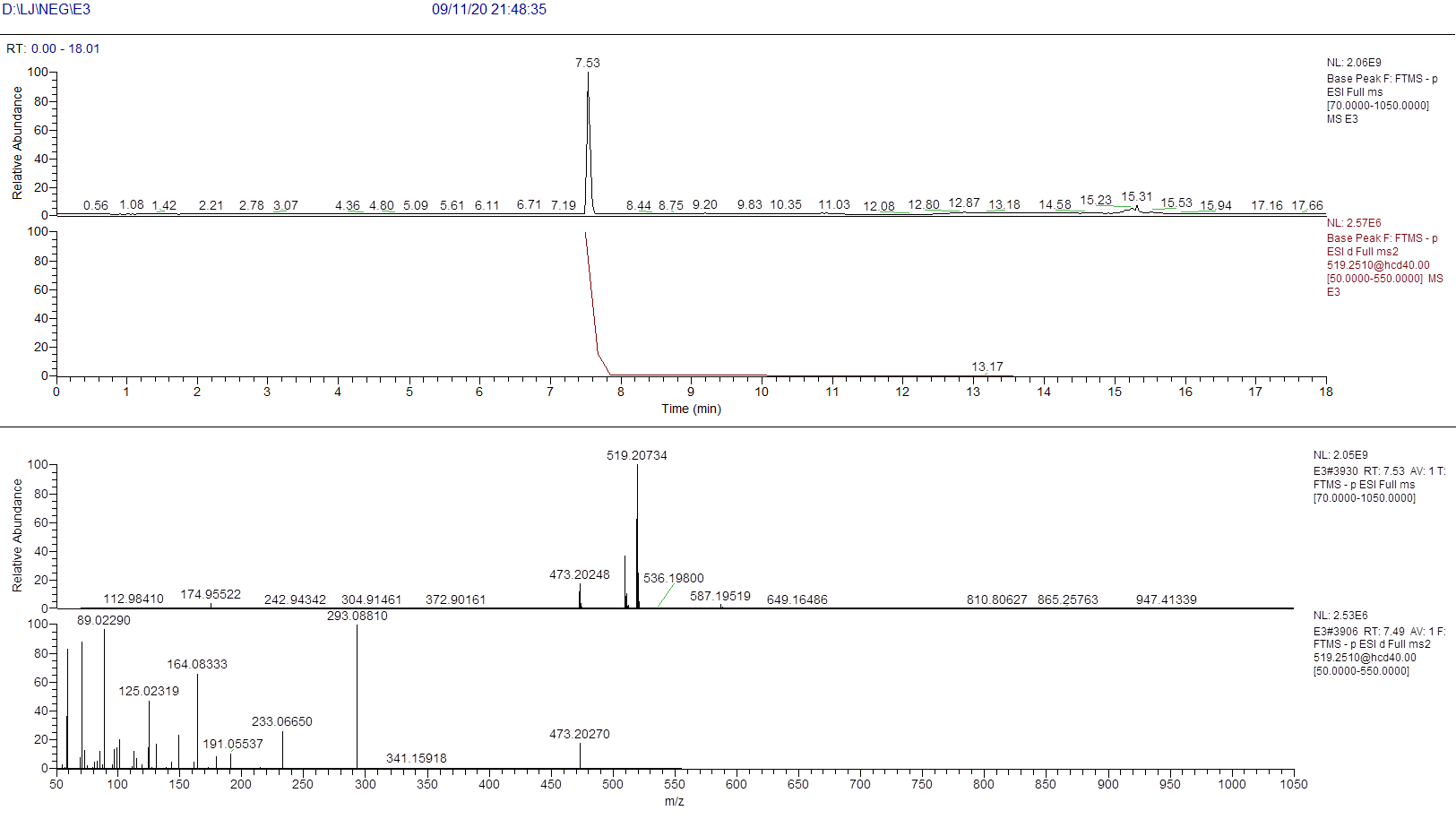


**Figure S2.** ^1^H-NMR spectrum of compound **1** in CD3OD.


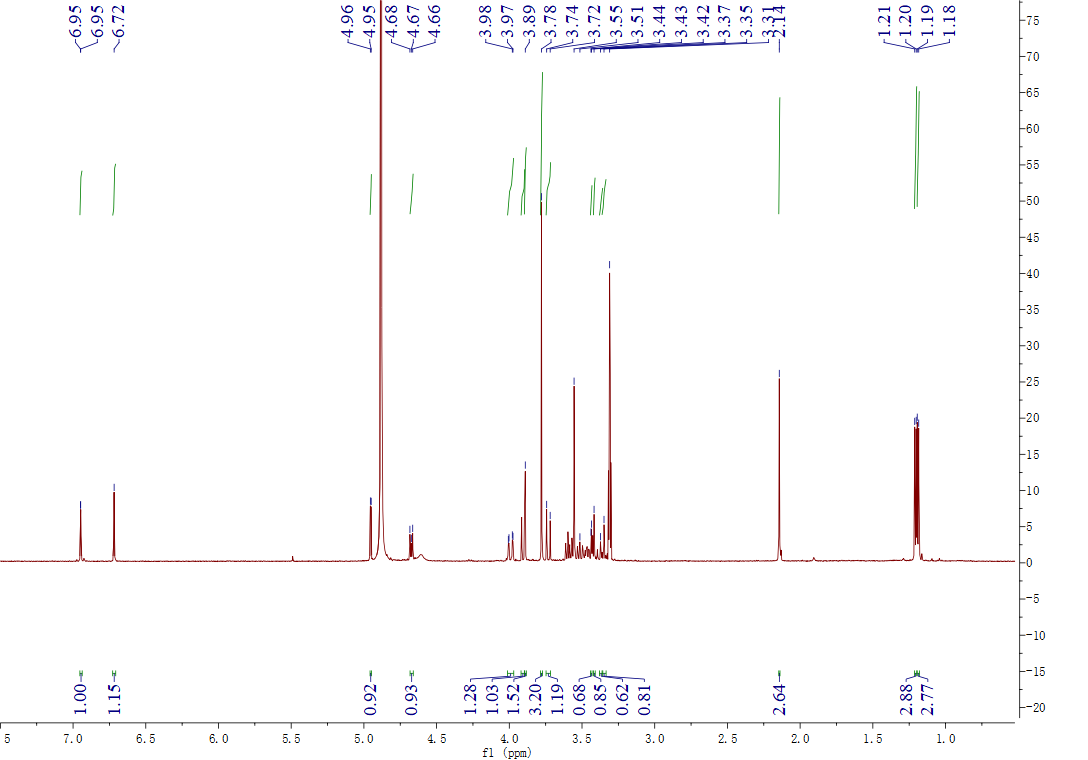


**Figure S3.** ^13^C-NMR spectrum of compound **1** in CD3OD.


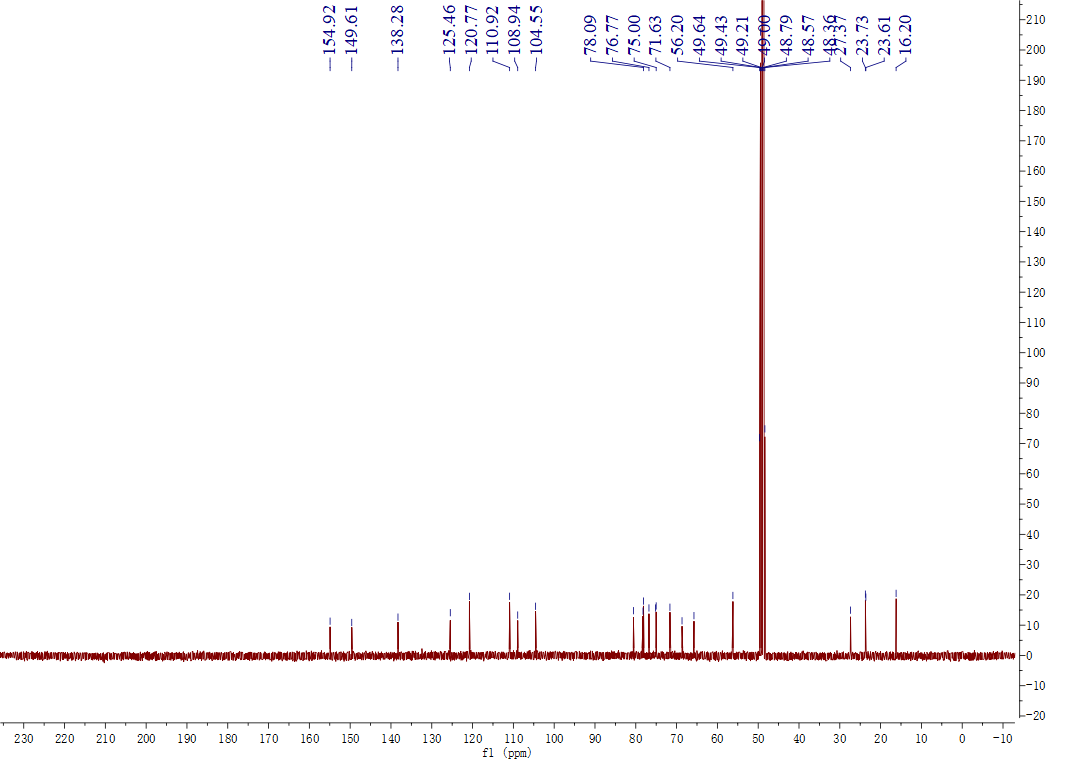


**Figure S4** ^1^H-^1^H COSY spectrum of compound **1** in CD_3_OD.


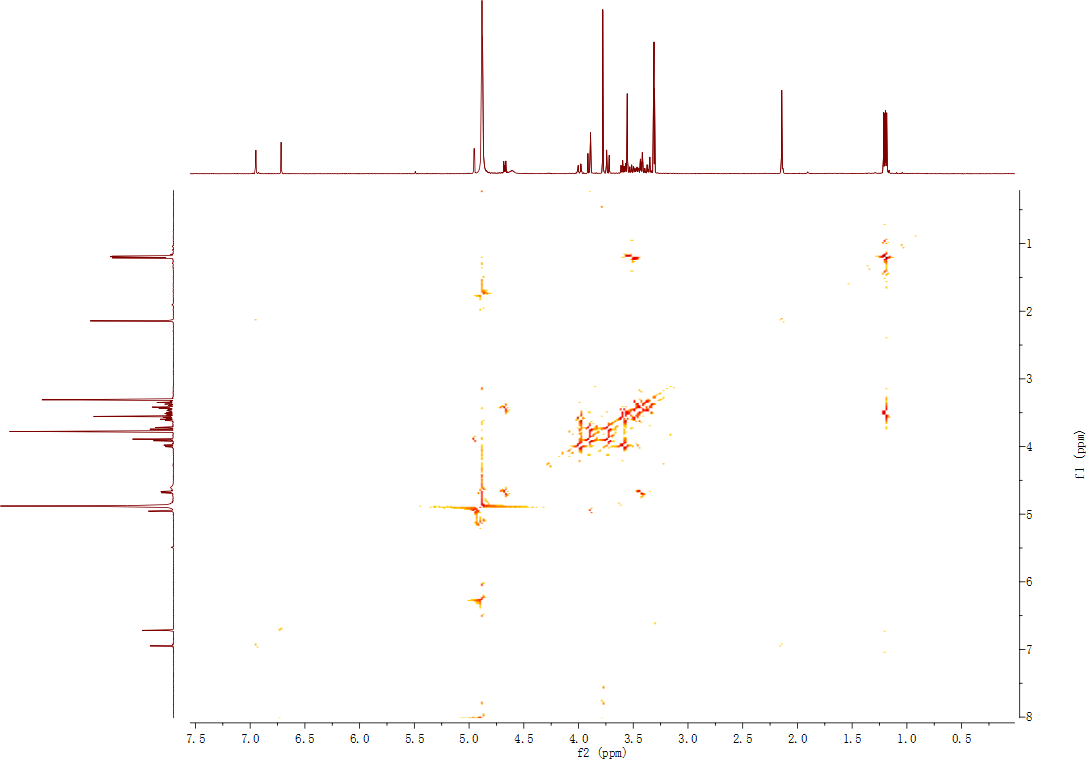


**Figure S5.** HSQC spectrum of compound **1** in CD_3_OD.


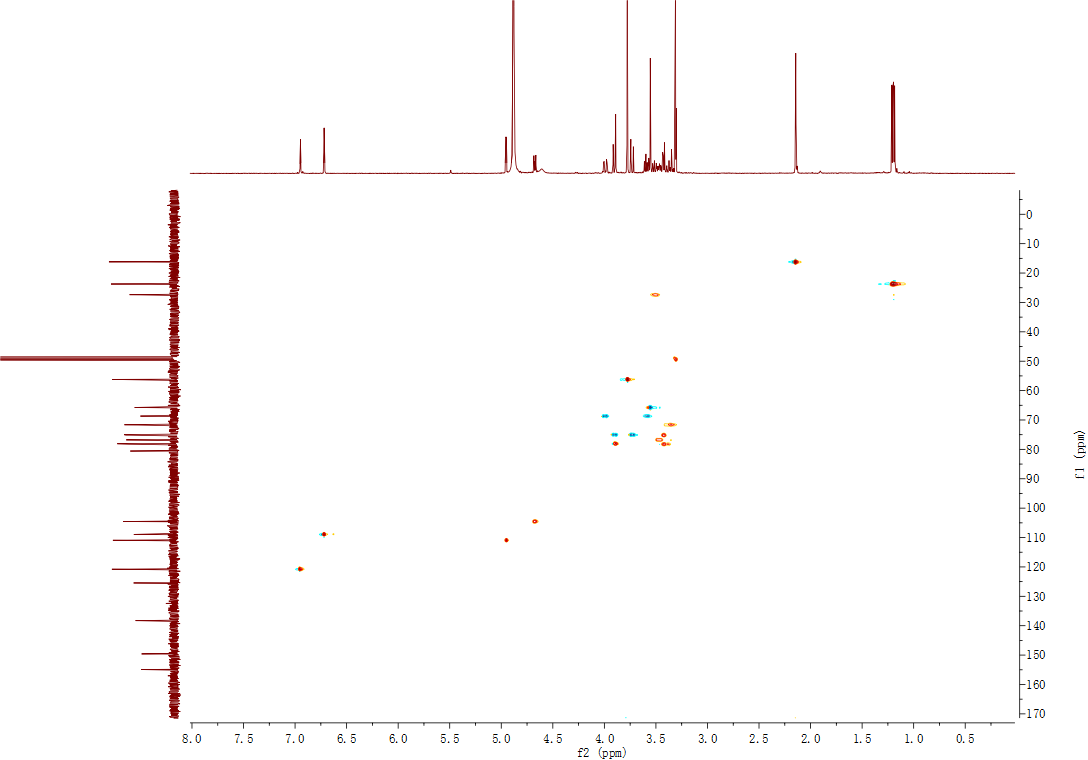


**Figure S6** HMBC spectrum of compound **1** in CD_3_OD.


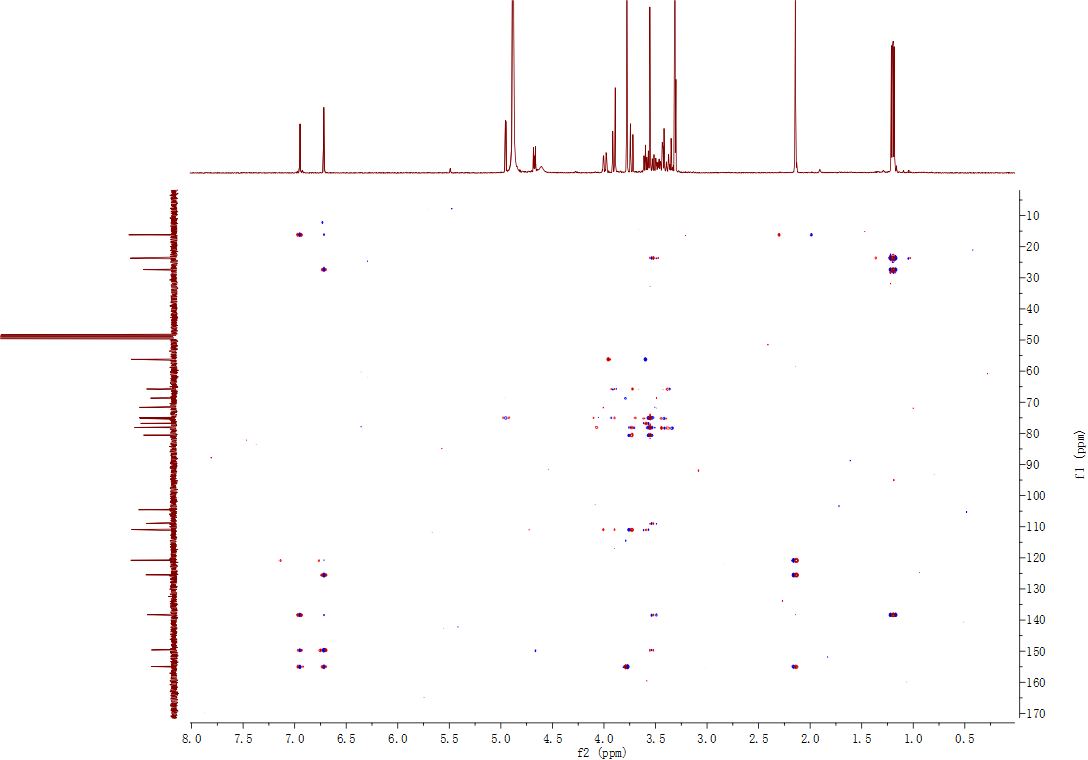


| **Figure S7** | IR spectrum of compound **1** in CD_3_OD.  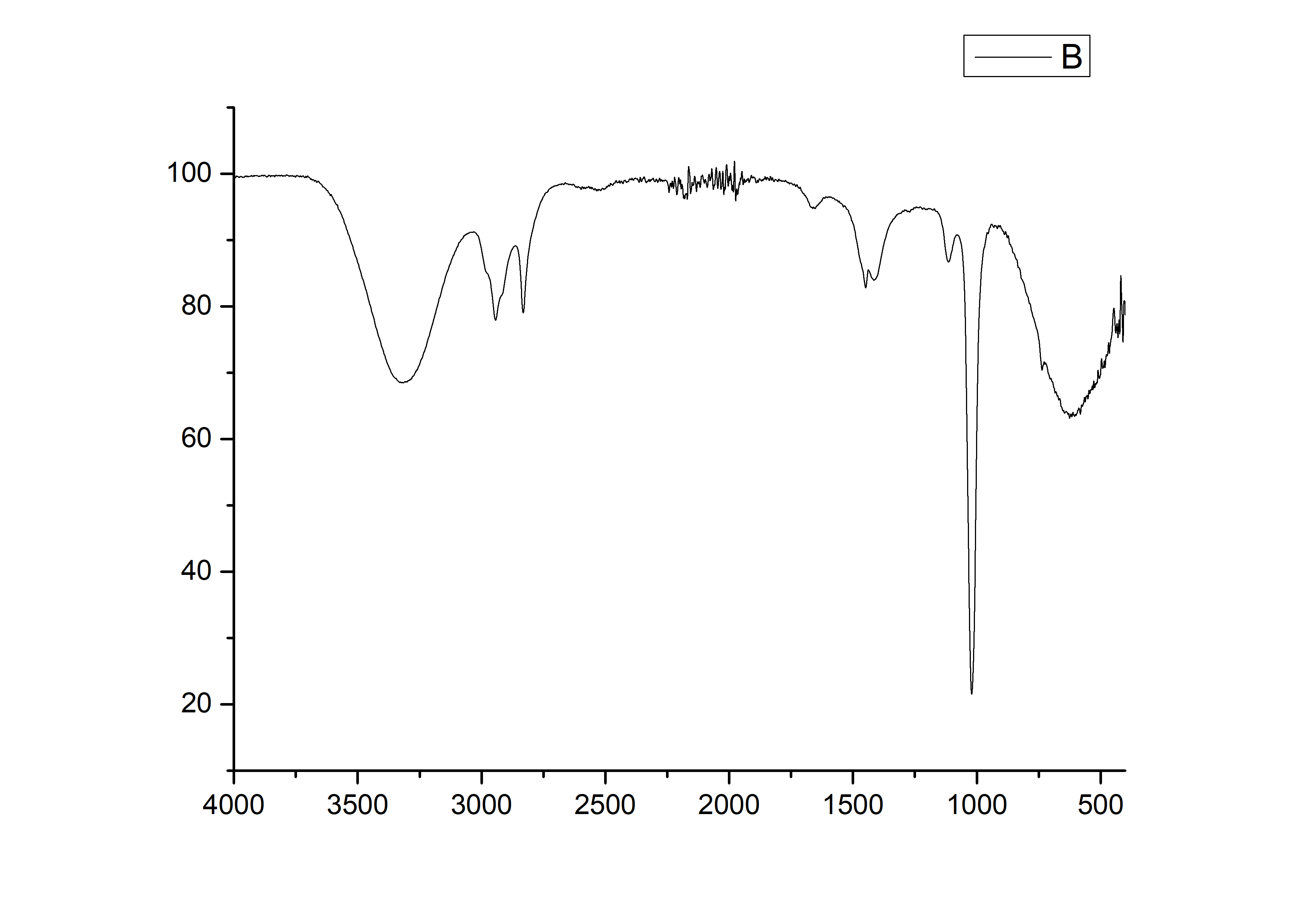 |
| --- | --- |

**Figure S8** UV spectrum of compound **1** in CD_3_OD.

**
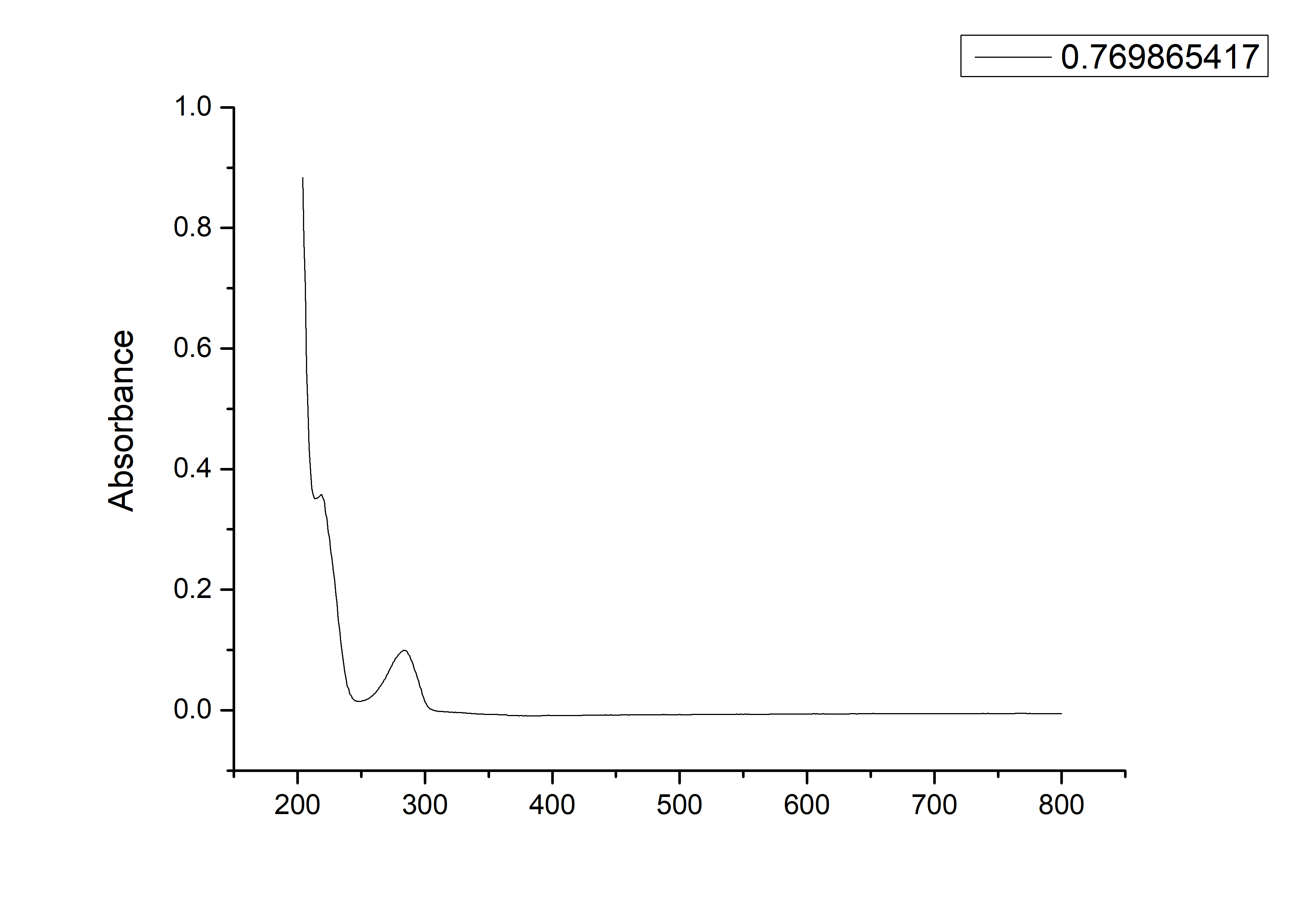
**
