# Supplementary figures and images for: Nigella sativa: A Dietary Supplement as an Immune-Modulator on the Basis of Bioactive Components (part 1 of 3)
Source: Front Nutr. 2021 Aug 17;8:722813. doi: 10.3389/fnut.2021.722813 (PMC8415885; doi:10.3389/fnut.2021.722813)

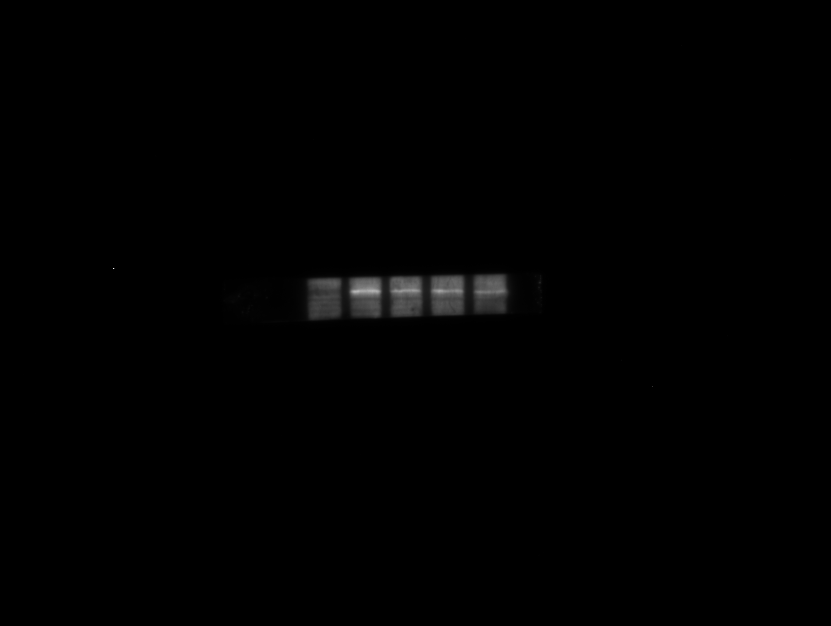

Supplement: Supplementary file 3 [file Data_Sheet_2.ZIP › Proteins for Anti-inflammation of compound 5/COX-2/2021-04-30_b-cox2_1_16bit.png]

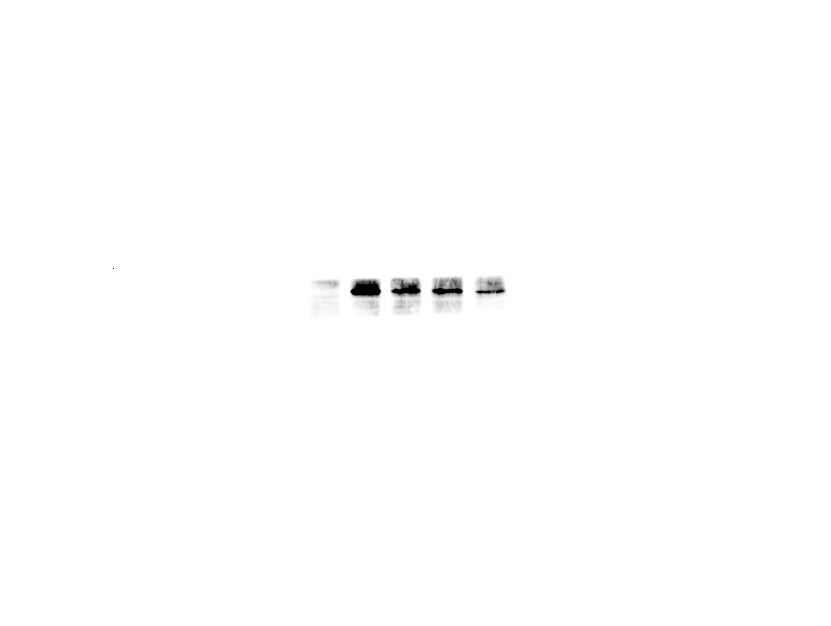

Supplement: Supplementary file 3 [file Data_Sheet_2.ZIP › Proteins for Anti-inflammation of compound 5/COX-2/2021-04-30_b-cox2_8bit.png]

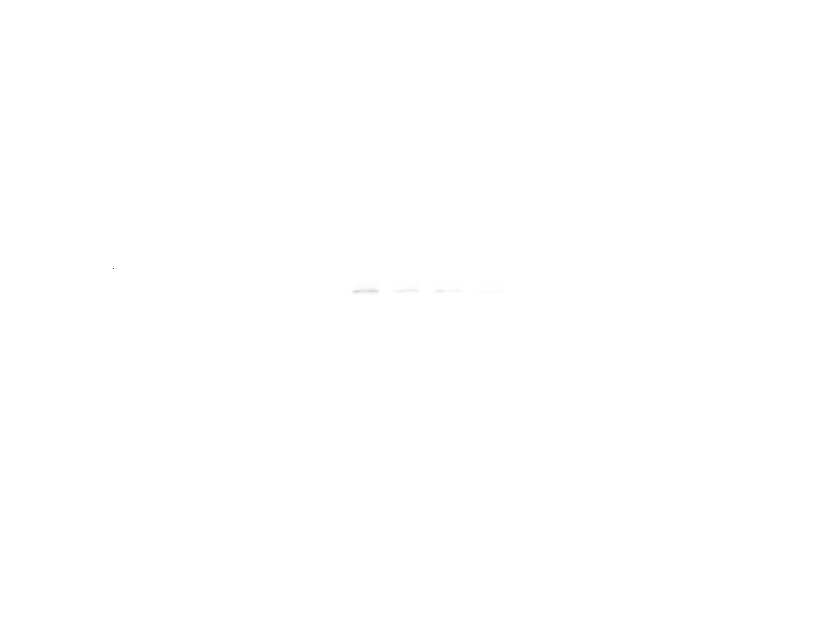

Supplement: Supplementary file 3 [file Data_Sheet_2.ZIP › Proteins for Anti-inflammation of compound 5/COX-2/contrast/contrast_0.png]

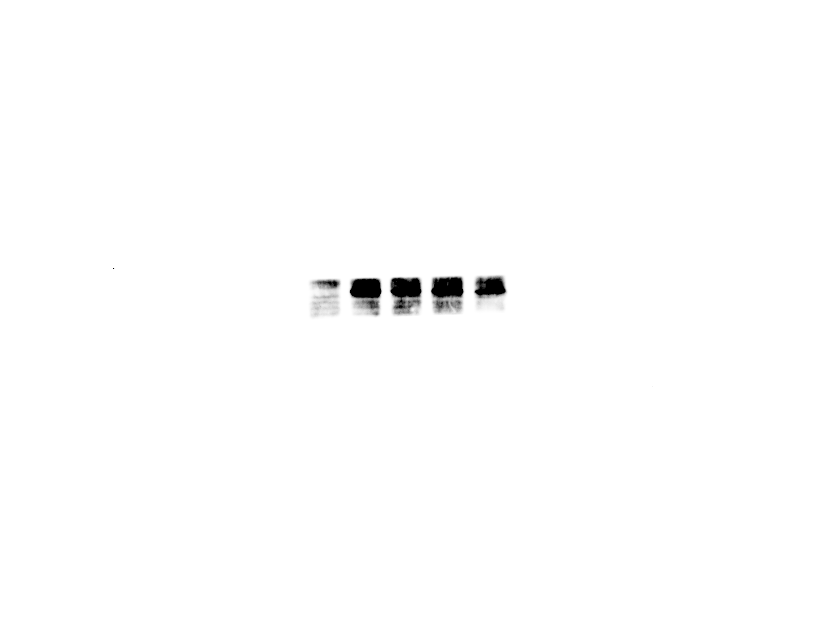

Supplement: Supplementary file 3 [file Data_Sheet_2.ZIP › Proteins for Anti-inflammation of compound 5/COX-2/contrast/contrast_2.png]

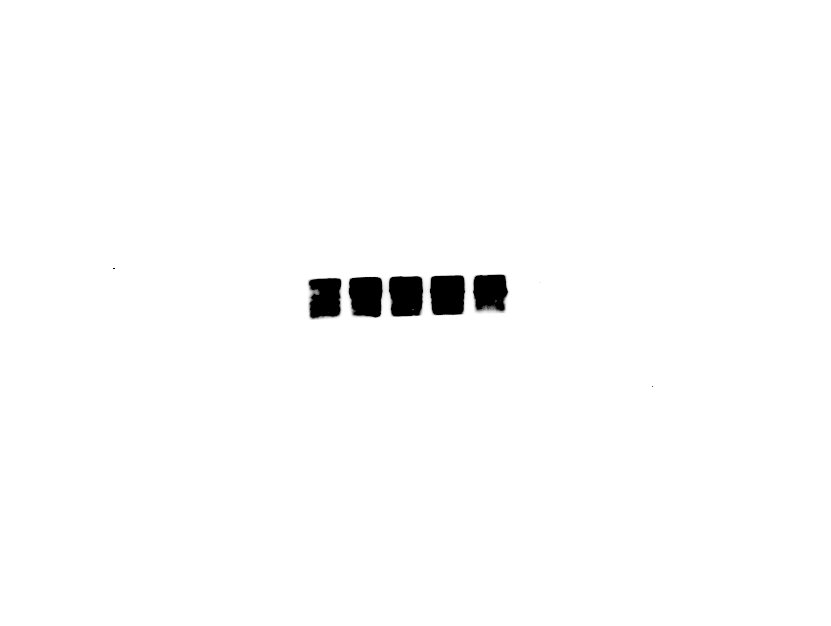

Supplement: Supplementary file 3 [file Data_Sheet_2.ZIP › Proteins for Anti-inflammation of compound 5/COX-2/contrast/contrast_3.png]

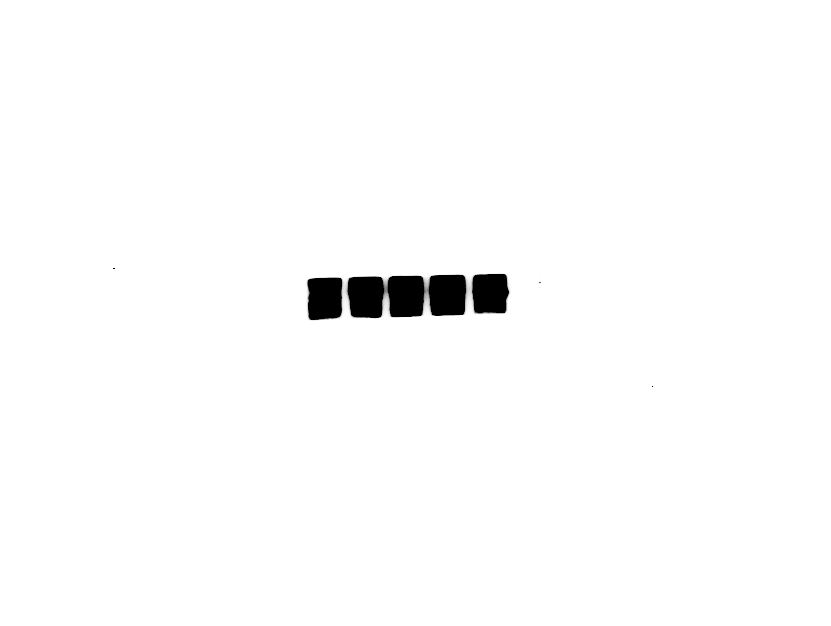

Supplement: Supplementary file 3 [file Data_Sheet_2.ZIP › Proteins for Anti-inflammation of compound 5/COX-2/contrast/contrast_4.png]

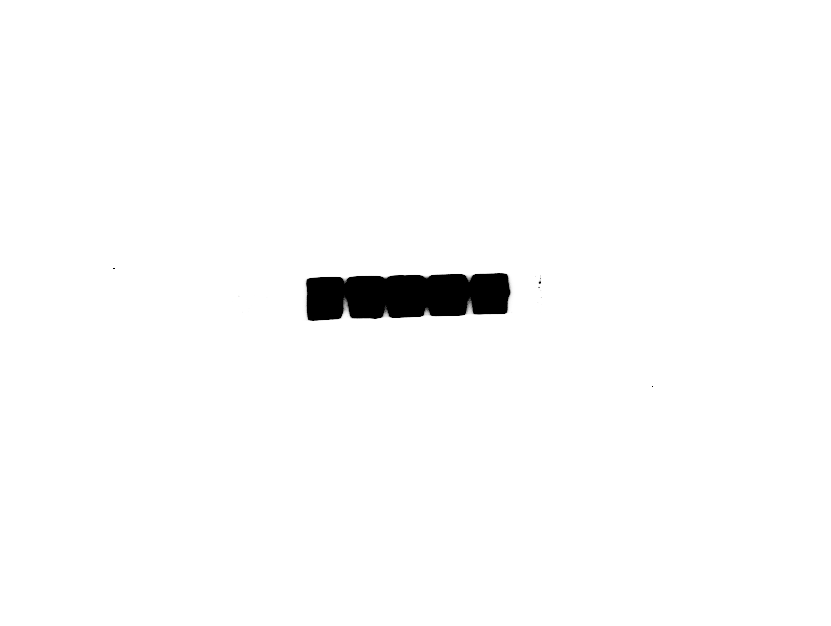

Supplement: Supplementary file 3 [file Data_Sheet_2.ZIP › Proteins for Anti-inflammation of compound 5/COX-2/contrast/contrast_5.png]

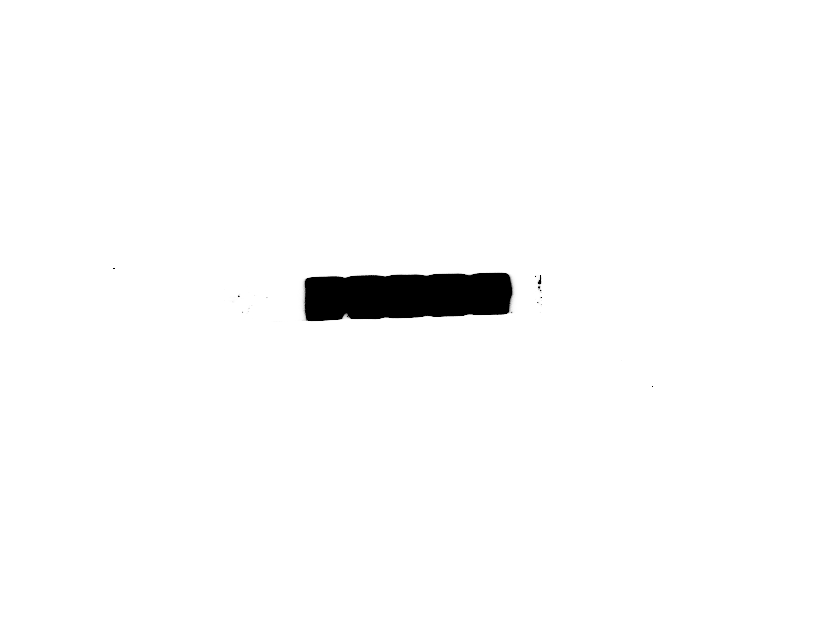

Supplement: Supplementary file 3 [file Data_Sheet_2.ZIP › Proteins for Anti-inflammation of compound 5/COX-2/contrast/contrast_6.png]

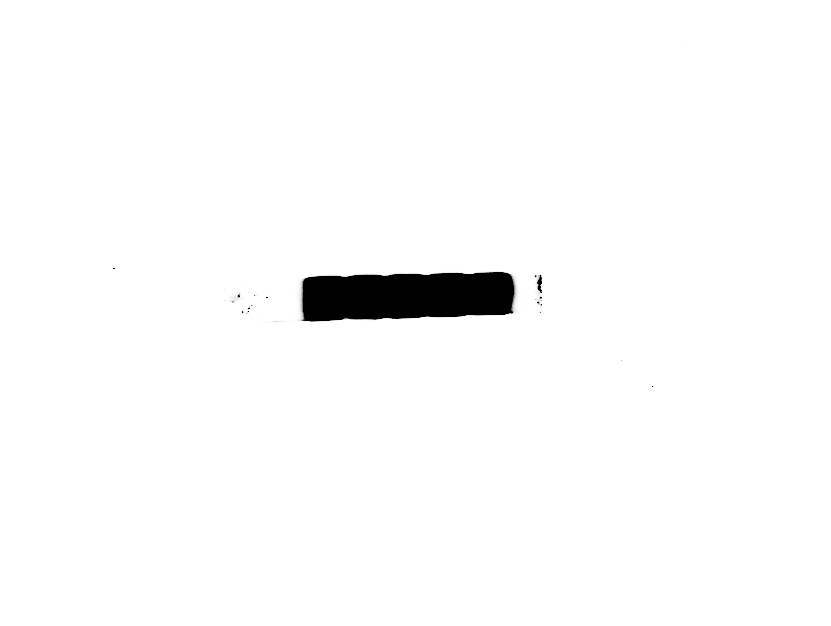

Supplement: Supplementary file 3 [file Data_Sheet_2.ZIP › Proteins for Anti-inflammation of compound 5/COX-2/contrast/contrast_7.png]

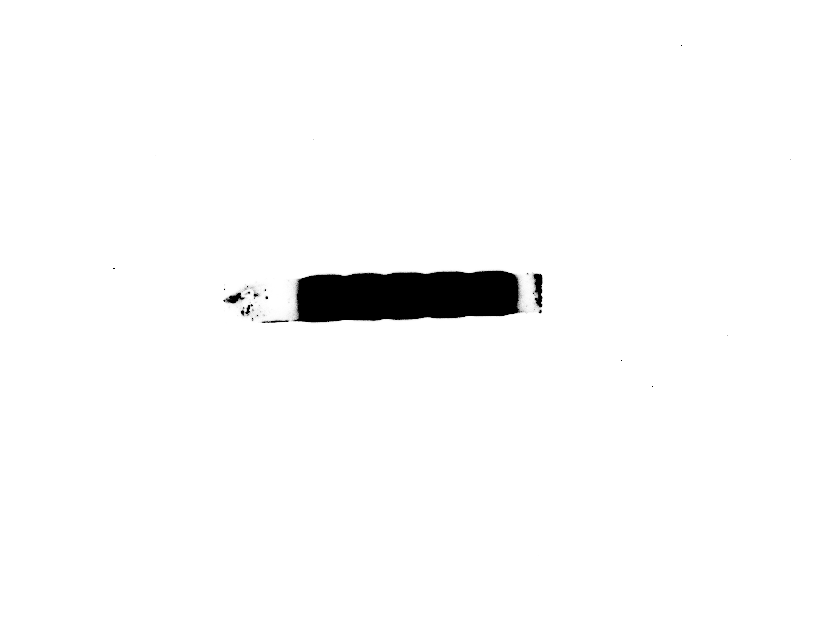

Supplement: Supplementary file 3 [file Data_Sheet_2.ZIP › Proteins for Anti-inflammation of compound 5/COX-2/contrast/contrast_8.png]

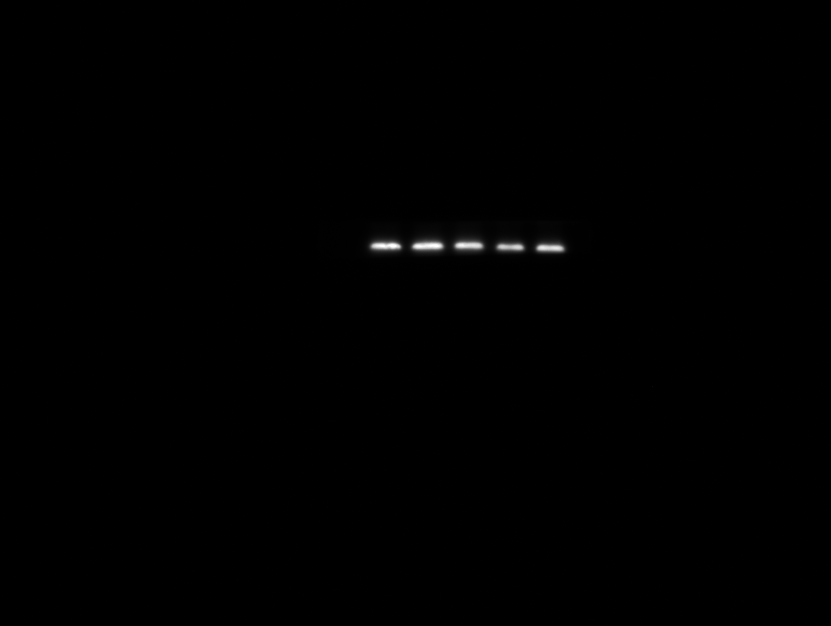

Supplement: Supplementary file 3 [file Data_Sheet_2.ZIP › Proteins for Anti-inflammation of compound 5/Ia╩Ba┴/2021-05-07_Ikba_1_16bit.png]

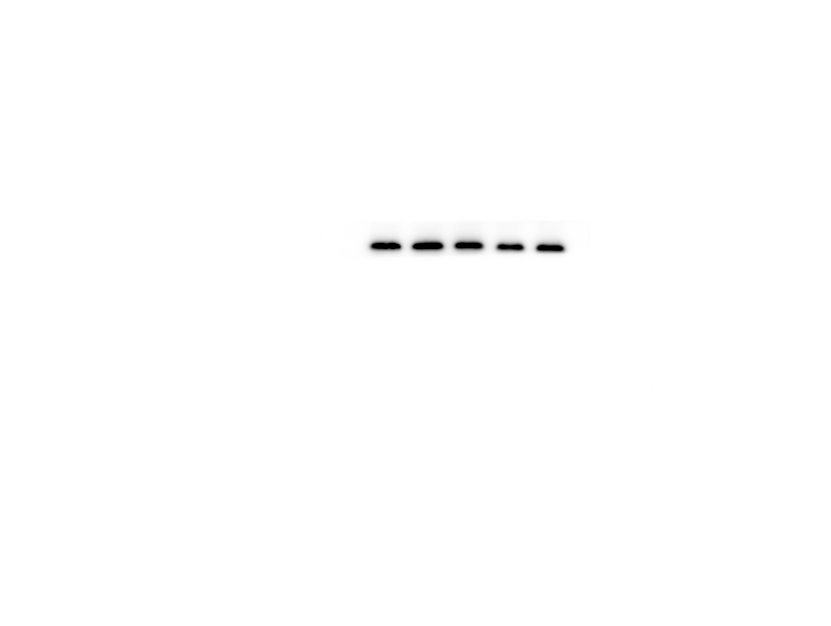

Supplement: Supplementary file 3 [file Data_Sheet_2.ZIP › Proteins for Anti-inflammation of compound 5/Ia╩Ba┴/2021-05-07_Ikba_8bit.png]

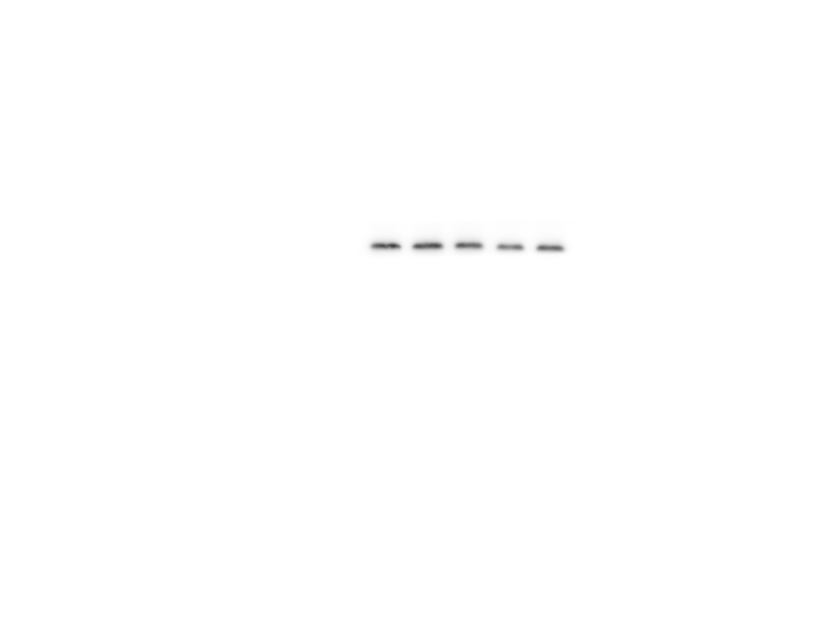

Supplement: Supplementary file 3 [file Data_Sheet_2.ZIP › Proteins for Anti-inflammation of compound 5/Ia╩Ba┴/contrast/contrast_0.png]

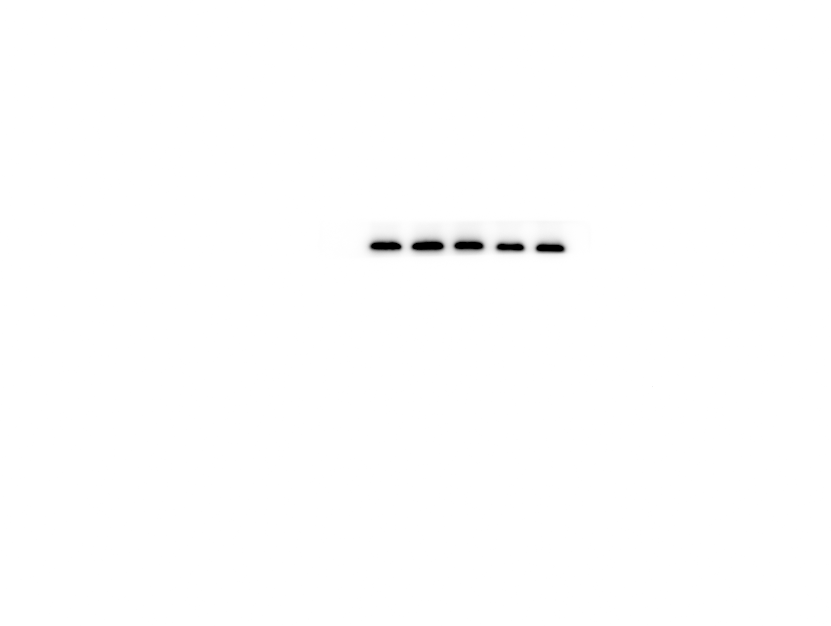

Supplement: Supplementary file 3 [file Data_Sheet_2.ZIP › Proteins for Anti-inflammation of compound 5/Ia╩Ba┴/contrast/contrast_2.png]

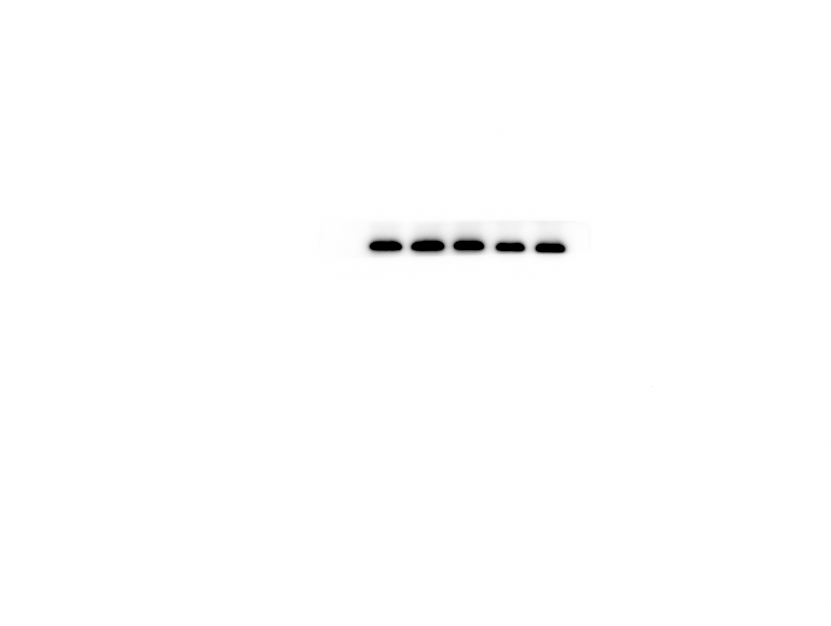

Supplement: Supplementary file 3 [file Data_Sheet_2.ZIP › Proteins for Anti-inflammation of compound 5/Ia╩Ba┴/contrast/contrast_3.png]

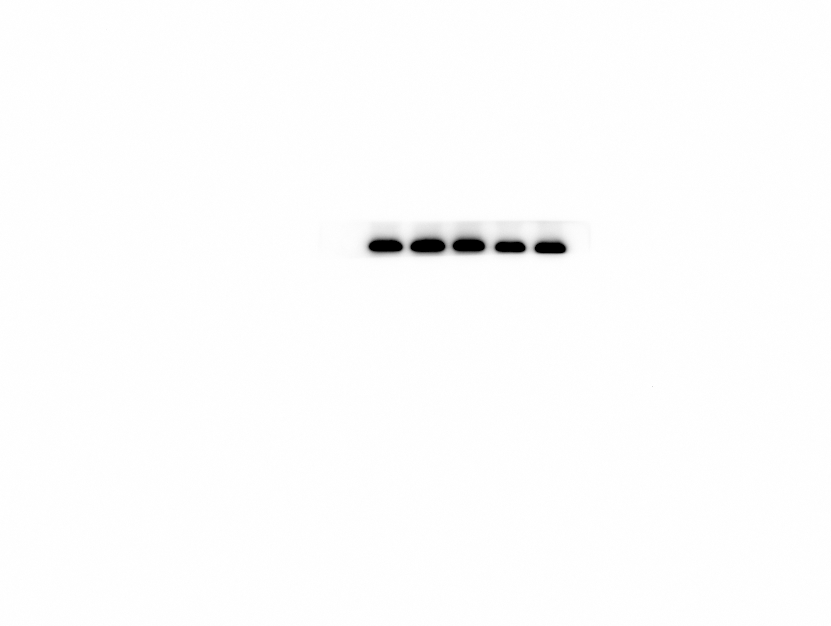

Supplement: Supplementary file 3 [file Data_Sheet_2.ZIP › Proteins for Anti-inflammation of compound 5/Ia╩Ba┴/contrast/contrast_4.png]

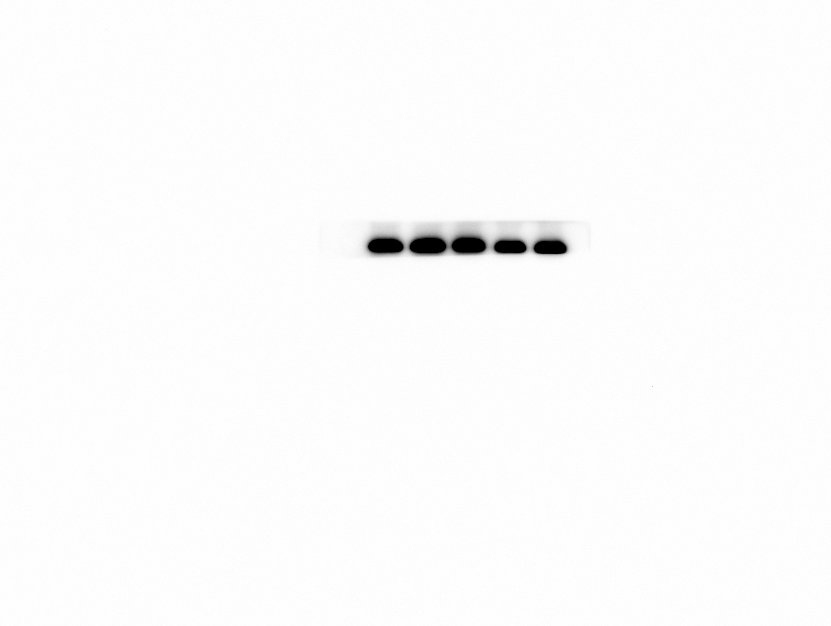

Supplement: Supplementary file 3 [file Data_Sheet_2.ZIP › Proteins for Anti-inflammation of compound 5/Ia╩Ba┴/contrast/contrast_5.png]

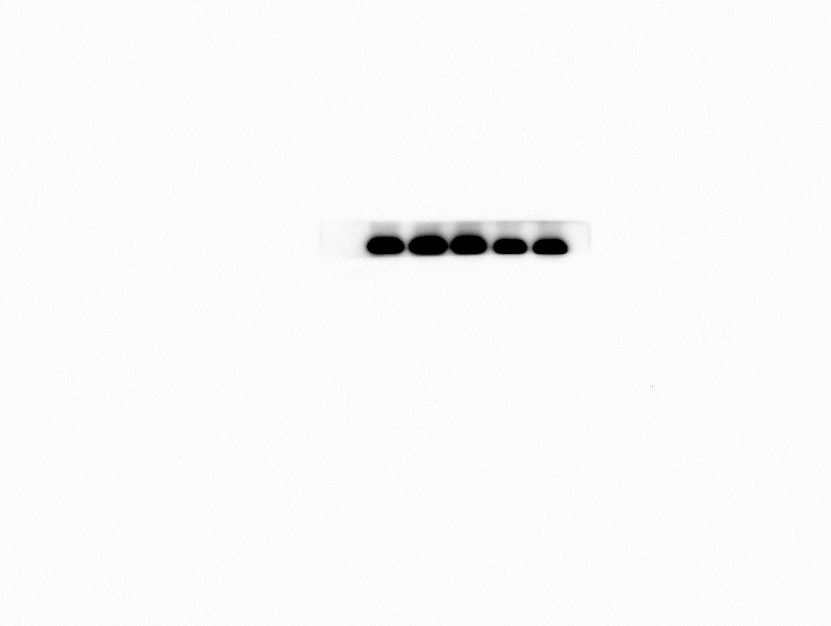

Supplement: Supplementary file 3 [file Data_Sheet_2.ZIP › Proteins for Anti-inflammation of compound 5/Ia╩Ba┴/contrast/contrast_6.png]

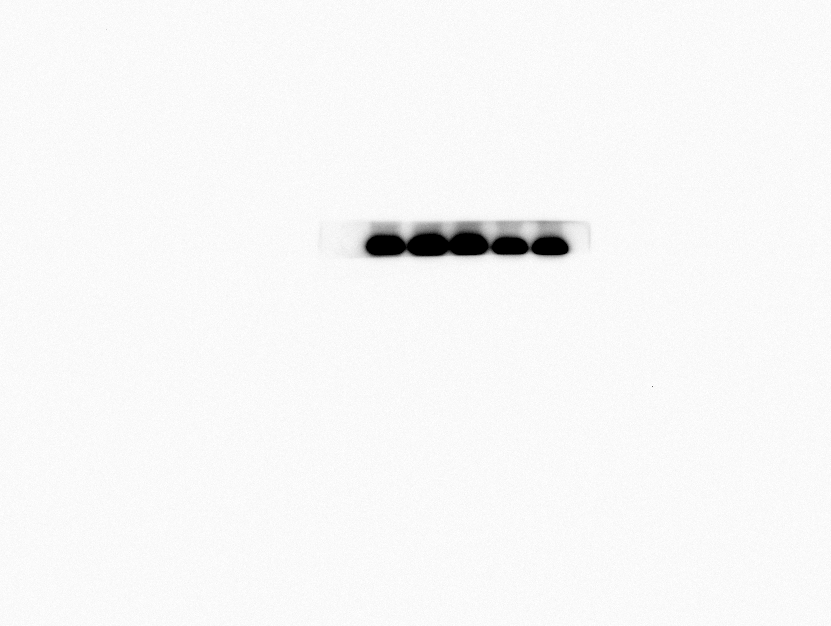

Supplement: Supplementary file 3 [file Data_Sheet_2.ZIP › Proteins for Anti-inflammation of compound 5/Ia╩Ba┴/contrast/contrast_7.png]

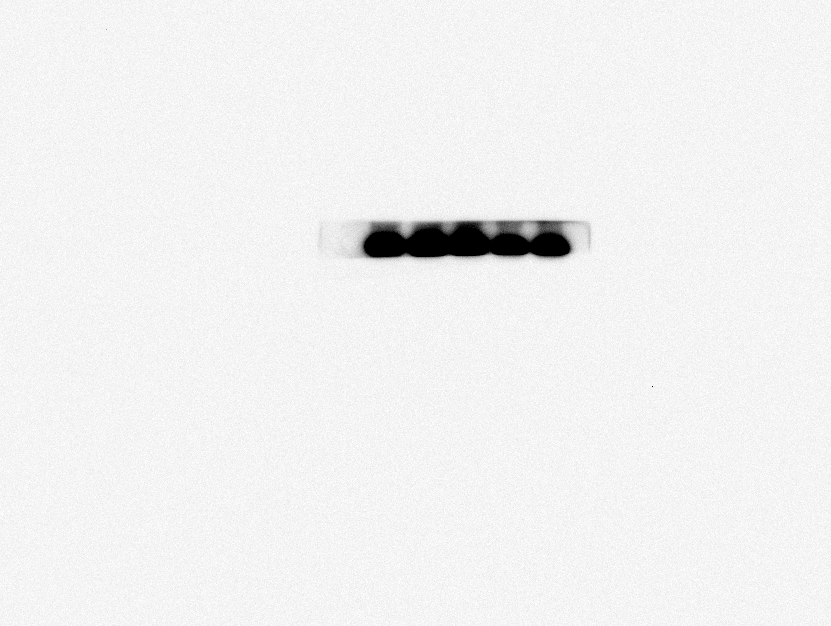

Supplement: Supplementary file 3 [file Data_Sheet_2.ZIP › Proteins for Anti-inflammation of compound 5/Ia╩Ba┴/contrast/contrast_8.png]

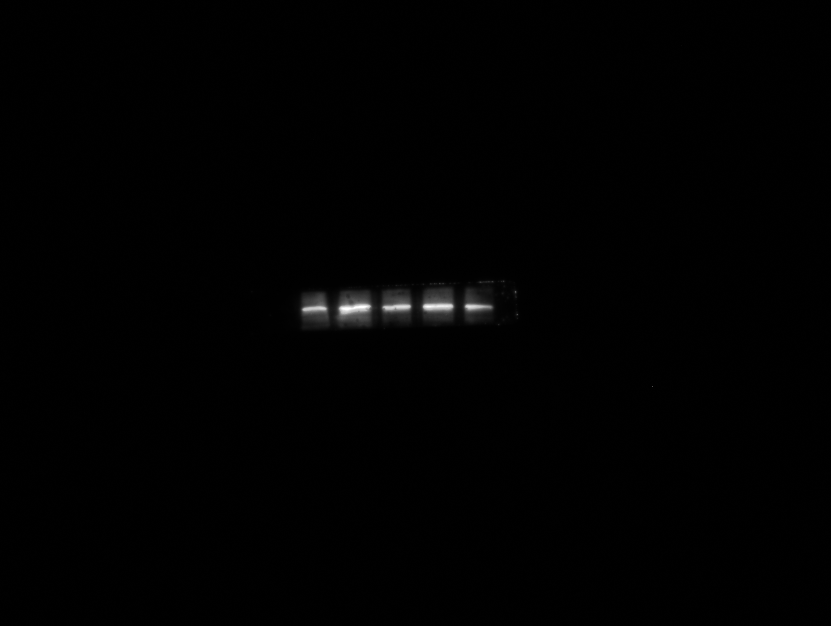

Supplement: Supplementary file 3 [file Data_Sheet_2.ZIP › Proteins for Anti-inflammation of compound 5/P-Ia╩B/2021-04-21_B3 plkb_1_16bit.png]

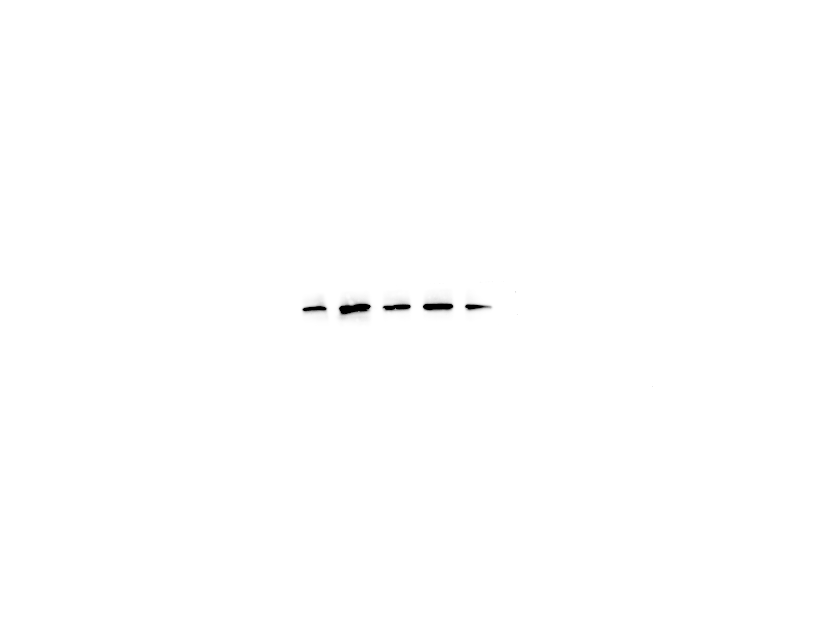

Supplement: Supplementary file 3 [file Data_Sheet_2.ZIP › Proteins for Anti-inflammation of compound 5/P-Ia╩B/2021-04-21_B3 plkb_8bit.png]

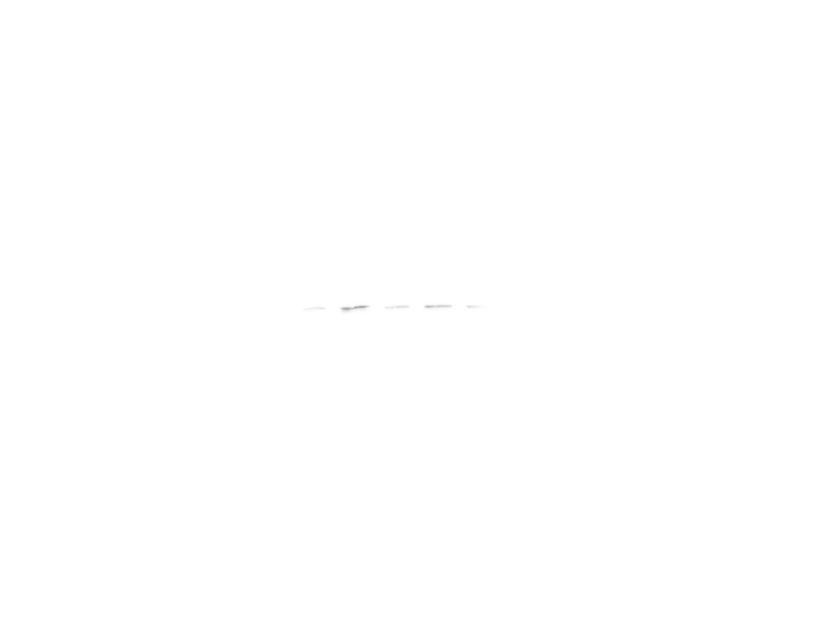

Supplement: Supplementary file 3 [file Data_Sheet_2.ZIP › Proteins for Anti-inflammation of compound 5/P-Ia╩B/contrast/contrast_0.png]

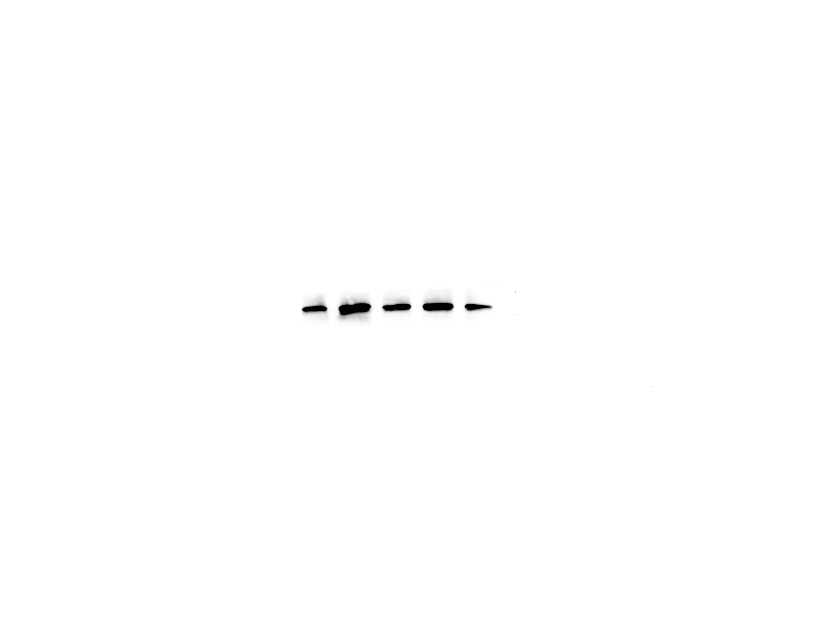

Supplement: Supplementary file 3 [file Data_Sheet_2.ZIP › Proteins for Anti-inflammation of compound 5/P-Ia╩B/contrast/contrast_2.png]

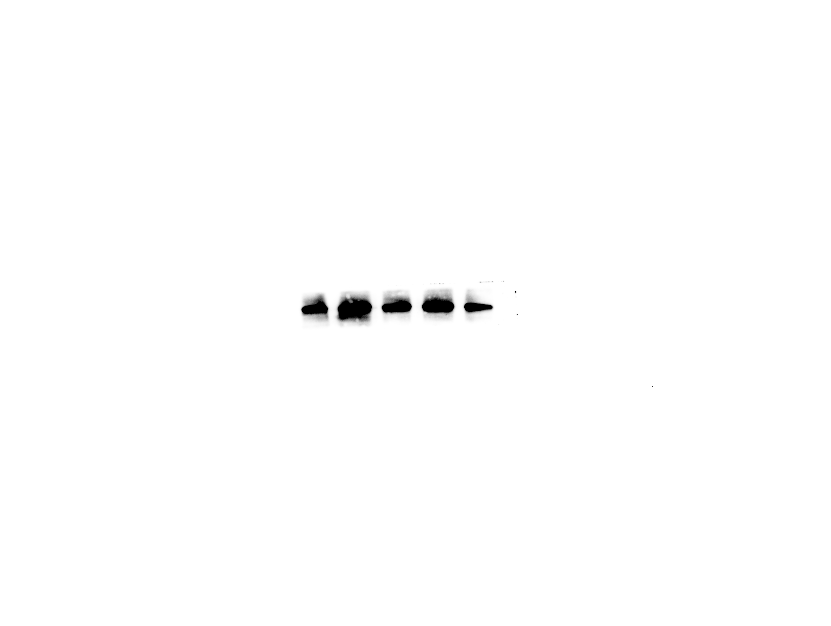

Supplement: Supplementary file 3 [file Data_Sheet_2.ZIP › Proteins for Anti-inflammation of compound 5/P-Ia╩B/contrast/contrast_3.png]

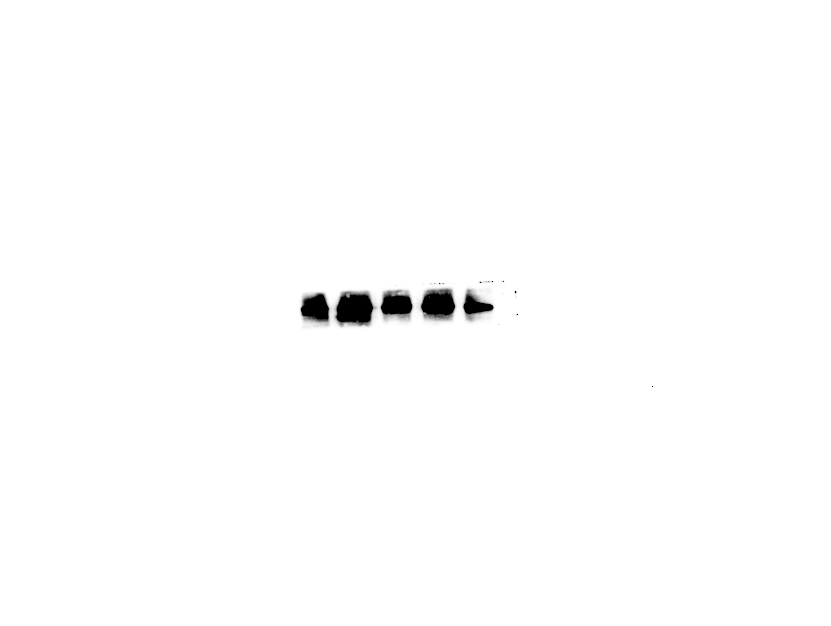

Supplement: Supplementary file 3 [file Data_Sheet_2.ZIP › Proteins for Anti-inflammation of compound 5/P-Ia╩B/contrast/contrast_4.png]

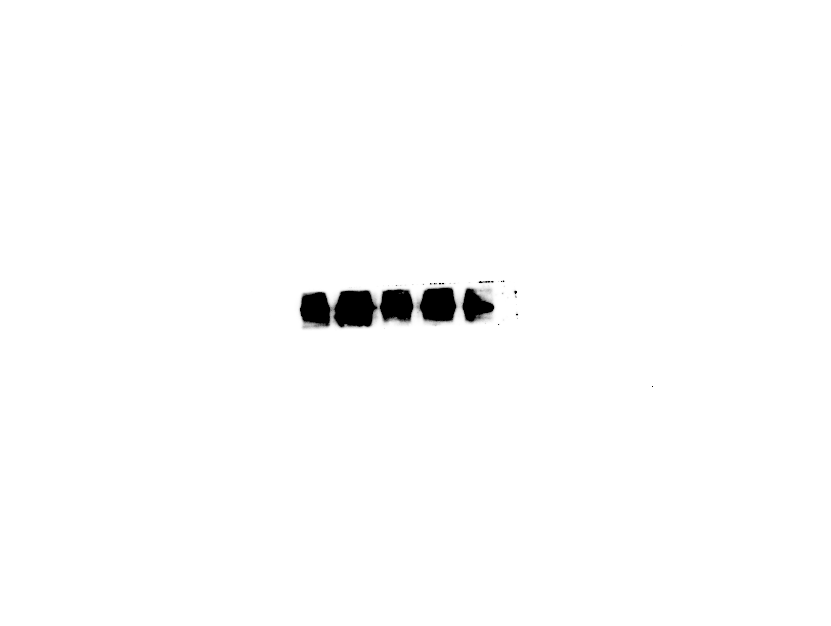

Supplement: Supplementary file 3 [file Data_Sheet_2.ZIP › Proteins for Anti-inflammation of compound 5/P-Ia╩B/contrast/contrast_5.png]

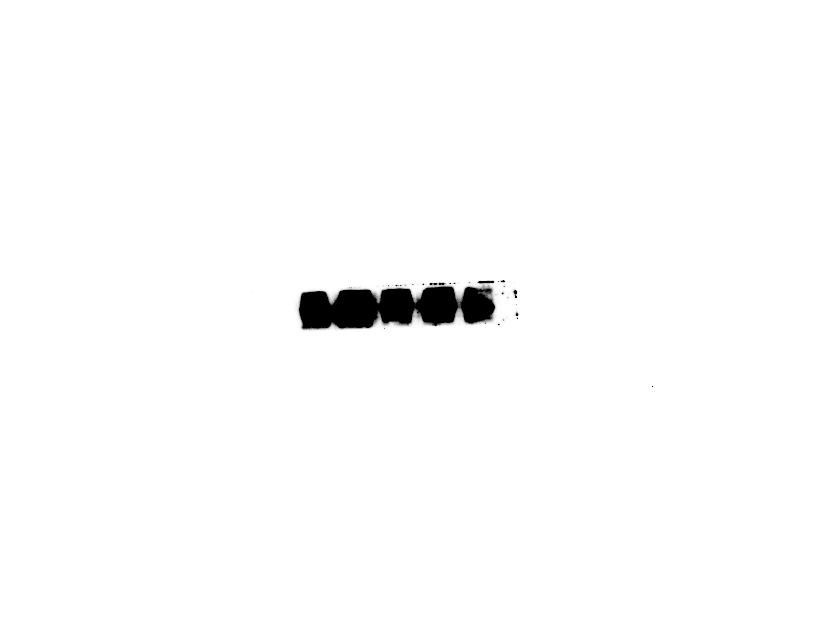

Supplement: Supplementary file 3 [file Data_Sheet_2.ZIP › Proteins for Anti-inflammation of compound 5/P-Ia╩B/contrast/contrast_6.png]

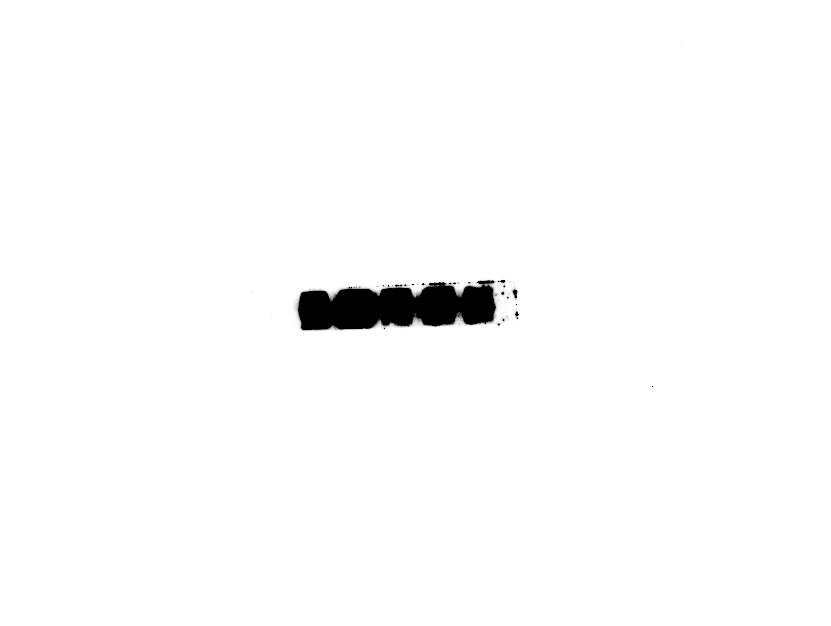

Supplement: Supplementary file 3 [file Data_Sheet_2.ZIP › Proteins for Anti-inflammation of compound 5/P-Ia╩B/contrast/contrast_7.png]

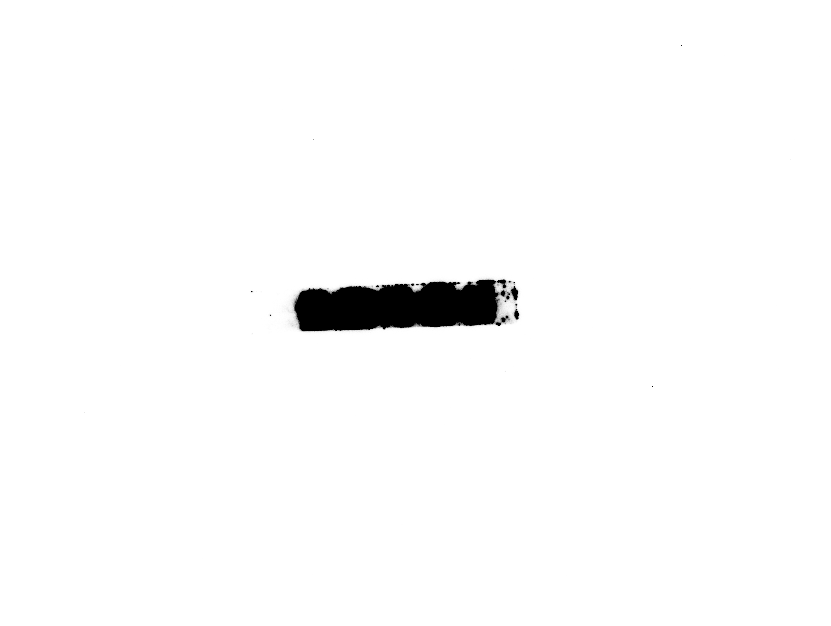

Supplement: Supplementary file 3 [file Data_Sheet_2.ZIP › Proteins for Anti-inflammation of compound 5/P-Ia╩B/contrast/contrast_8.png]

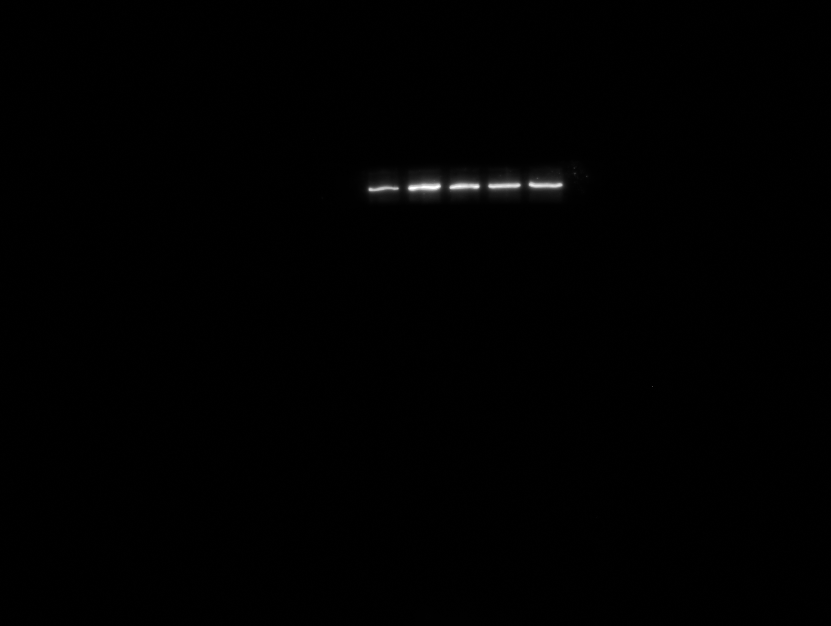

Supplement: Supplementary file 3 [file Data_Sheet_2.ZIP › Proteins for Anti-inflammation of compound 5/P-p65/2021-04-21 Zps pp65_1_16bit.png]

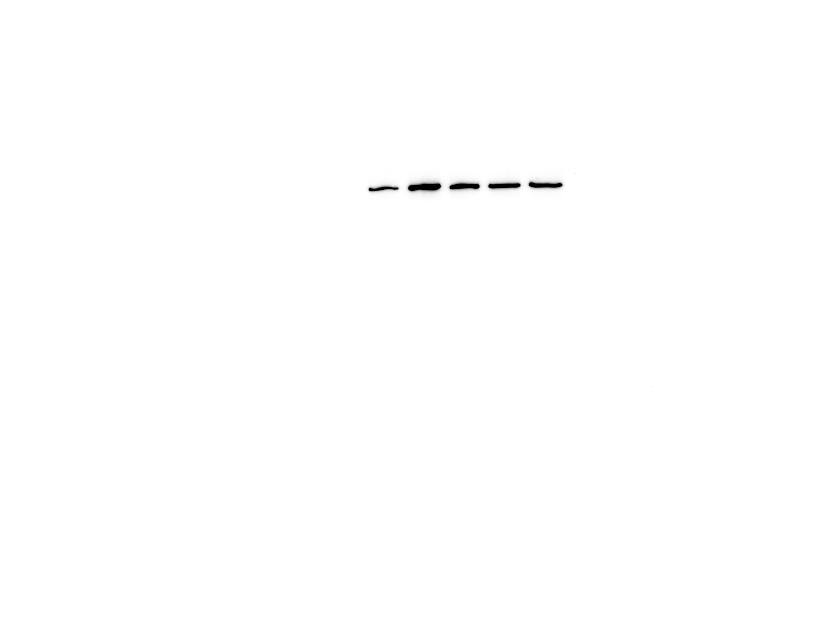

Supplement: Supplementary file 3 [file Data_Sheet_2.ZIP › Proteins for Anti-inflammation of compound 5/P-p65/2021-04-21 Zps pp65_8bit.png]

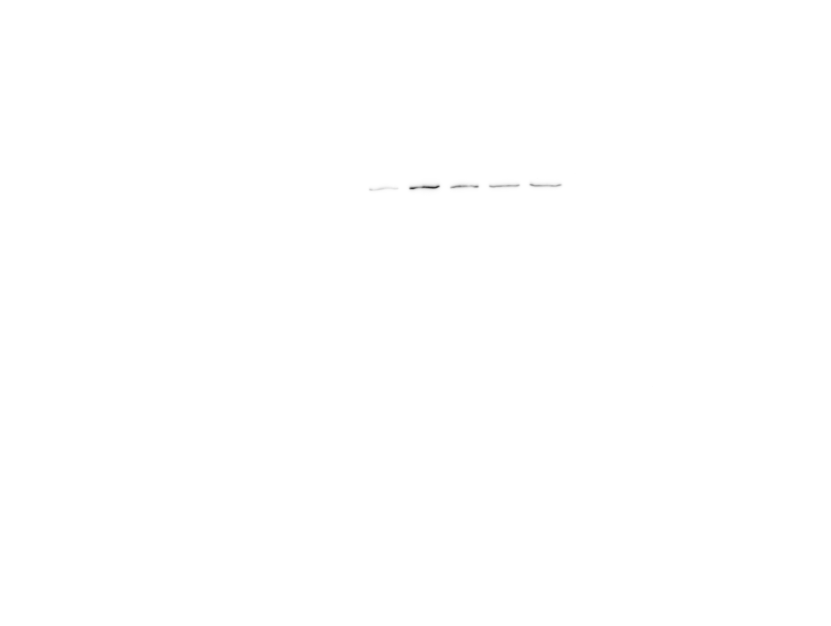

Supplement: Supplementary file 3 [file Data_Sheet_2.ZIP › Proteins for Anti-inflammation of compound 5/P-p65/contrast/contrast_0.png]

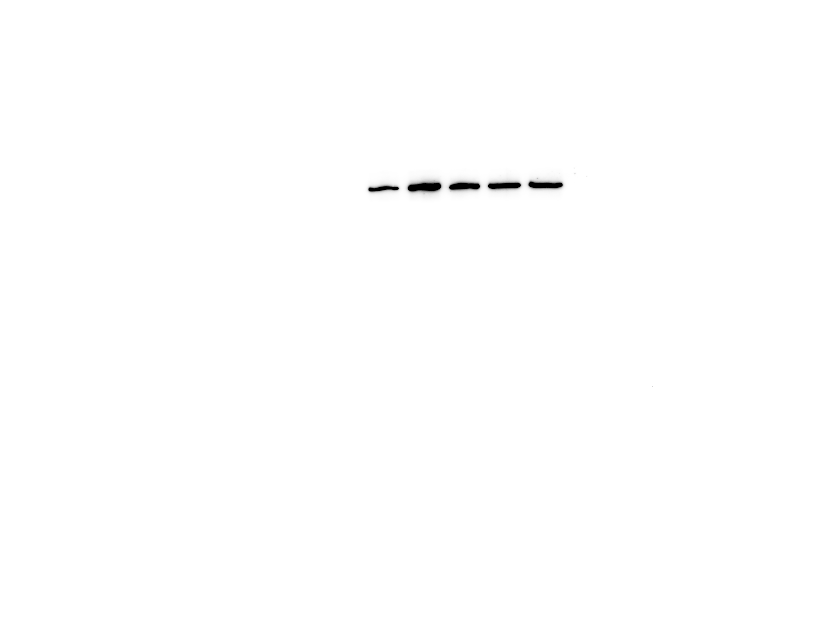

Supplement: Supplementary file 3 [file Data_Sheet_2.ZIP › Proteins for Anti-inflammation of compound 5/P-p65/contrast/contrast_2.png]

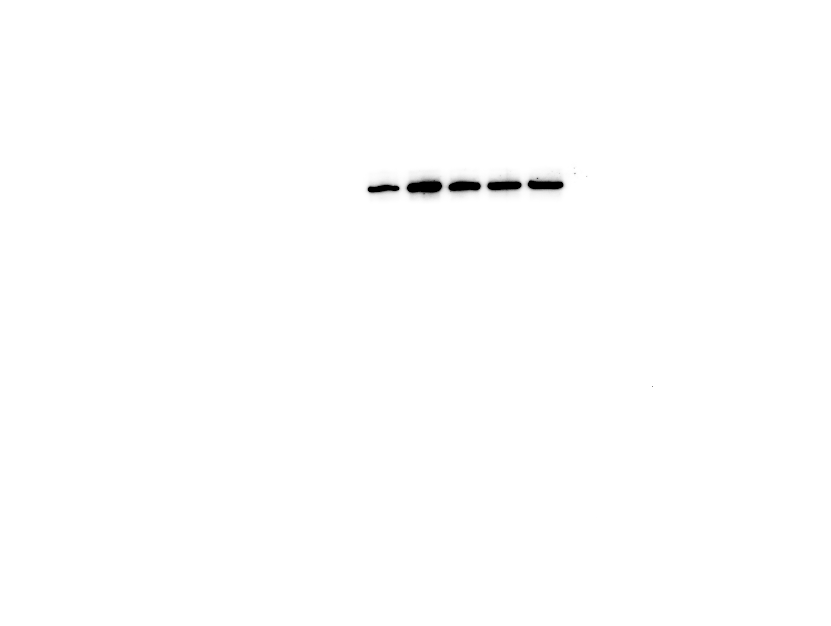

Supplement: Supplementary file 3 [file Data_Sheet_2.ZIP › Proteins for Anti-inflammation of compound 5/P-p65/contrast/contrast_3.png]

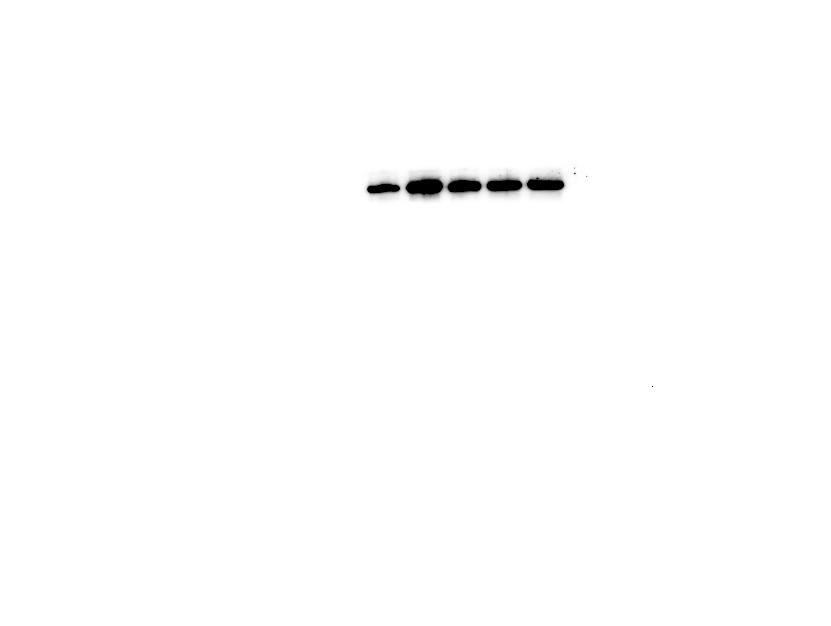

Supplement: Supplementary file 3 [file Data_Sheet_2.ZIP › Proteins for Anti-inflammation of compound 5/P-p65/contrast/contrast_4.png]

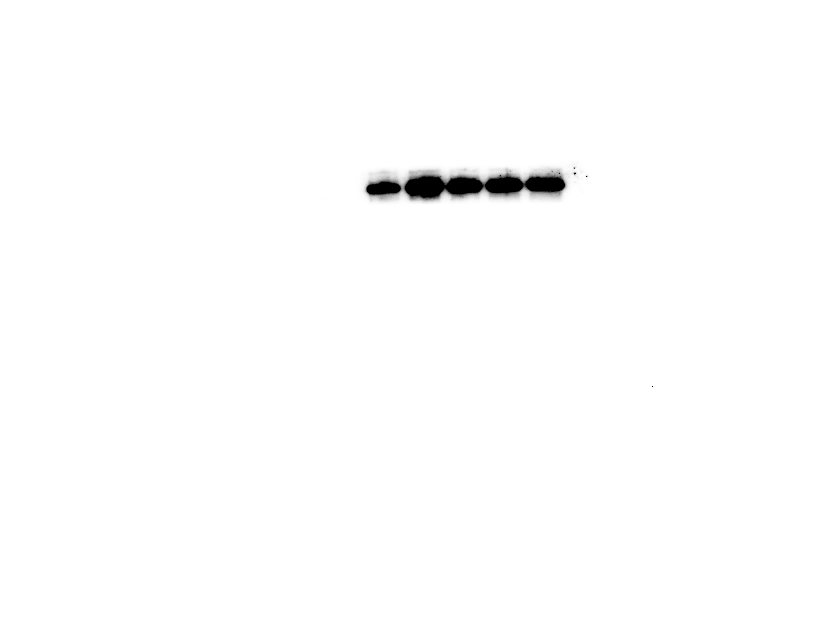

Supplement: Supplementary file 3 [file Data_Sheet_2.ZIP › Proteins for Anti-inflammation of compound 5/P-p65/contrast/contrast_5.png]

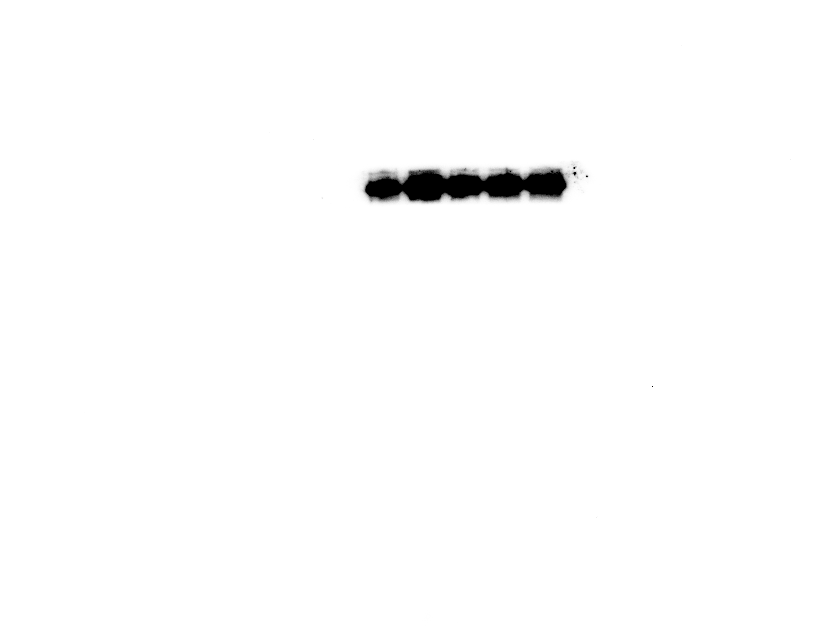

Supplement: Supplementary file 3 [file Data_Sheet_2.ZIP › Proteins for Anti-inflammation of compound 5/P-p65/contrast/contrast_6.png]

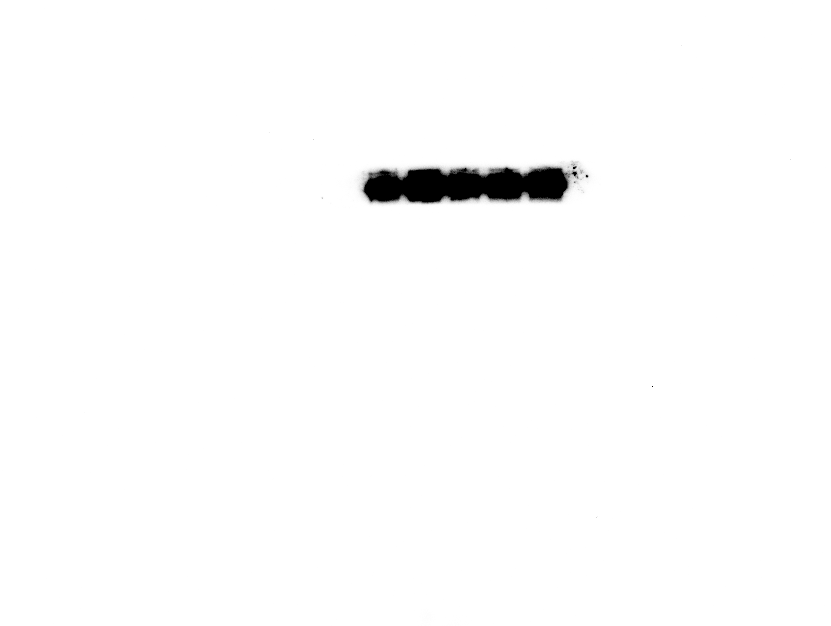

Supplement: Supplementary file 3 [file Data_Sheet_2.ZIP › Proteins for Anti-inflammation of compound 5/P-p65/contrast/contrast_7.png]

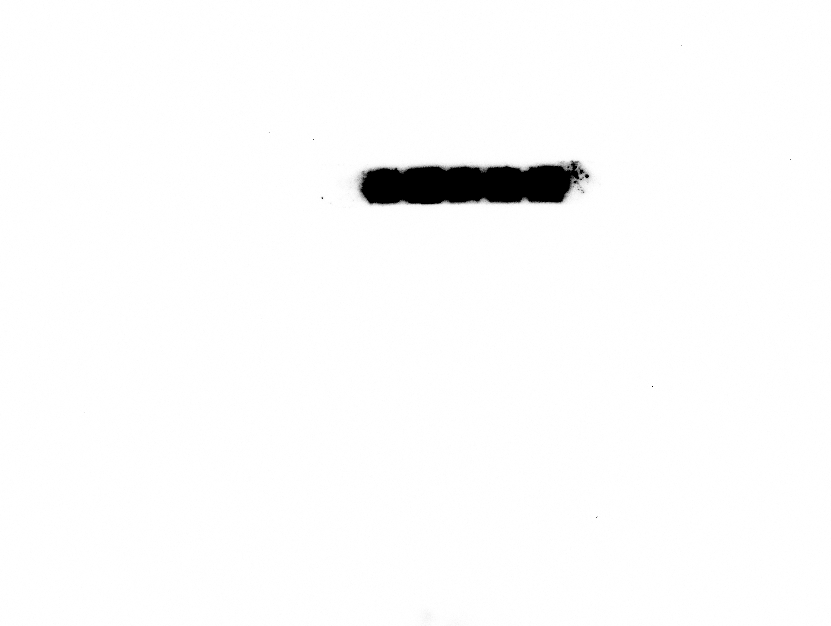

Supplement: Supplementary file 3 [file Data_Sheet_2.ZIP › Proteins for Anti-inflammation of compound 5/P-p65/contrast/contrast_8.png]

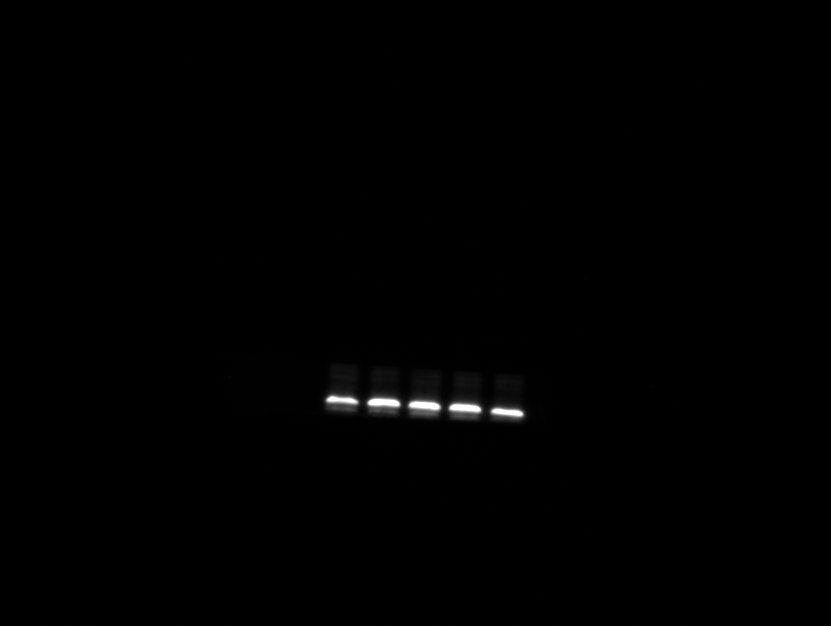

Supplement: Supplementary file 3 [file Data_Sheet_2.ZIP › Proteins for Anti-inflammation of compound 5/P65/2021-04-30_B-p65_1_16bit.png]

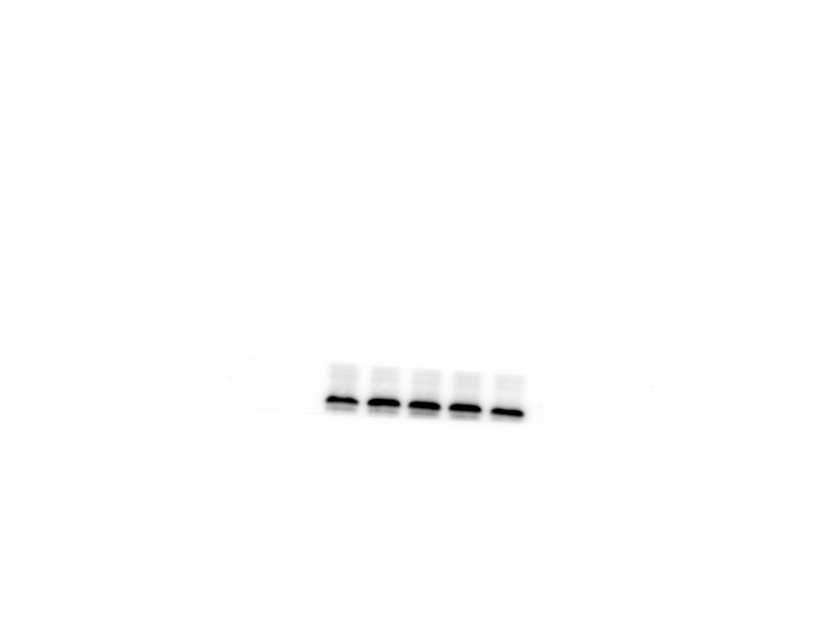

Supplement: Supplementary file 3 [file Data_Sheet_2.ZIP › Proteins for Anti-inflammation of compound 5/P65/2021-04-30_B-p65_8bit.png]

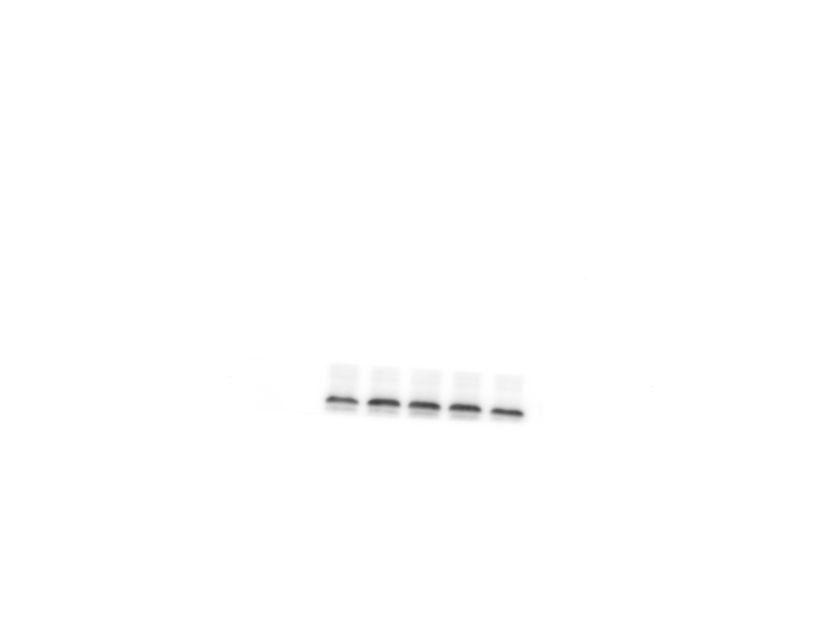

Supplement: Supplementary file 3 [file Data_Sheet_2.ZIP › Proteins for Anti-inflammation of compound 5/P65/contrast/contrast_0.png]

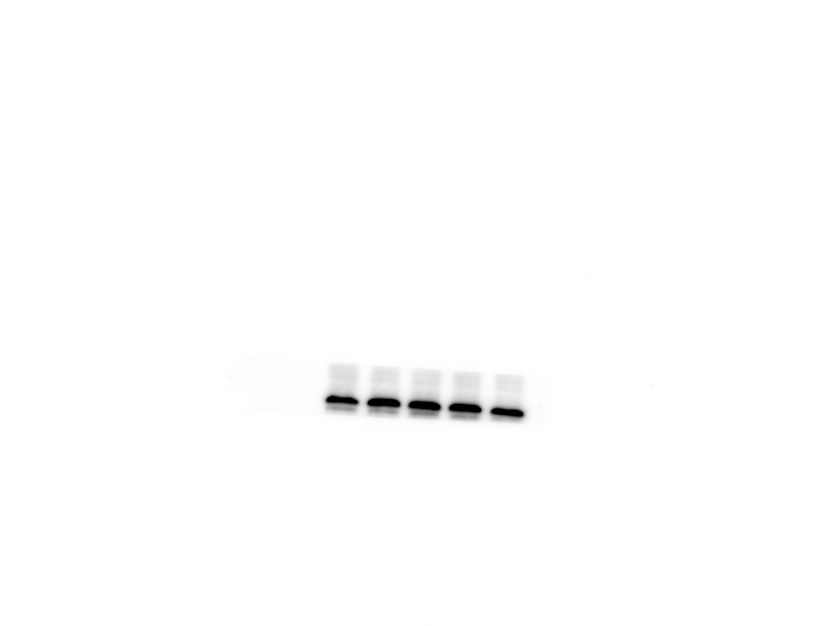

Supplement: Supplementary file 3 [file Data_Sheet_2.ZIP › Proteins for Anti-inflammation of compound 5/P65/contrast/contrast_2.png]

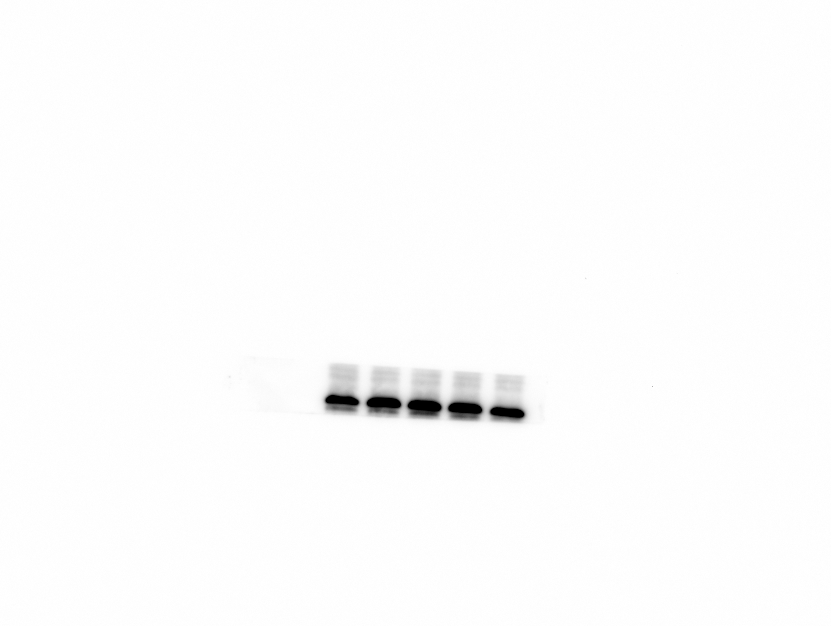

Supplement: Supplementary file 3 [file Data_Sheet_2.ZIP › Proteins for Anti-inflammation of compound 5/P65/contrast/contrast_3.png]

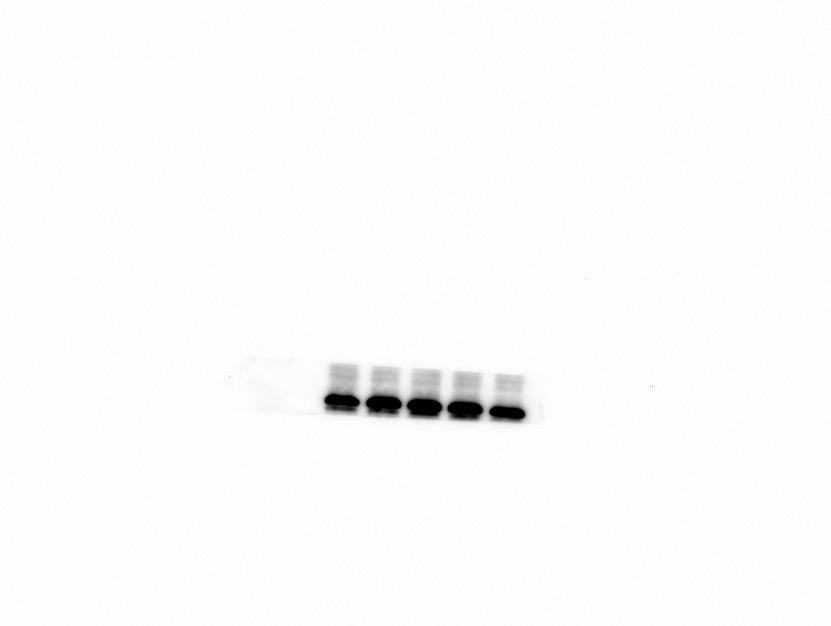

Supplement: Supplementary file 3 [file Data_Sheet_2.ZIP › Proteins for Anti-inflammation of compound 5/P65/contrast/contrast_4.png]

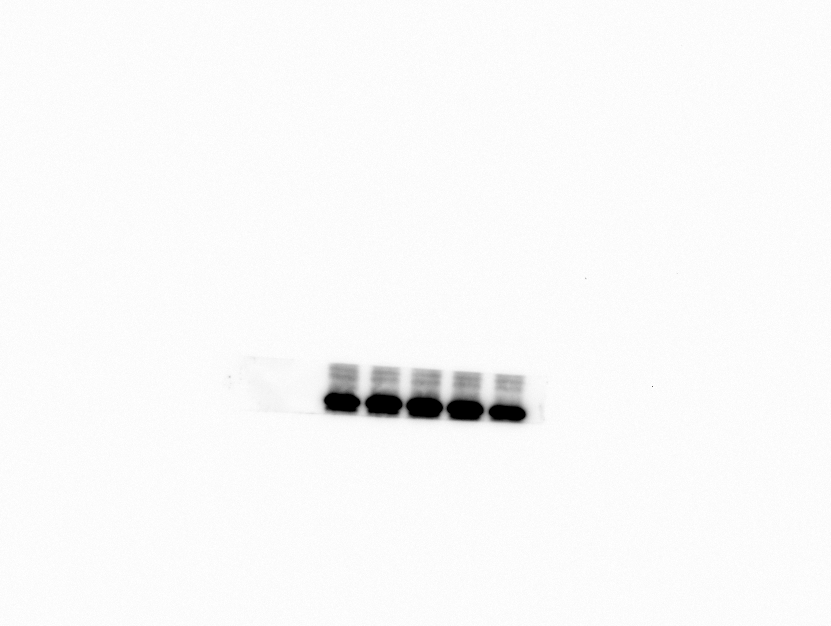

Supplement: Supplementary file 3 [file Data_Sheet_2.ZIP › Proteins for Anti-inflammation of compound 5/P65/contrast/contrast_5.png]

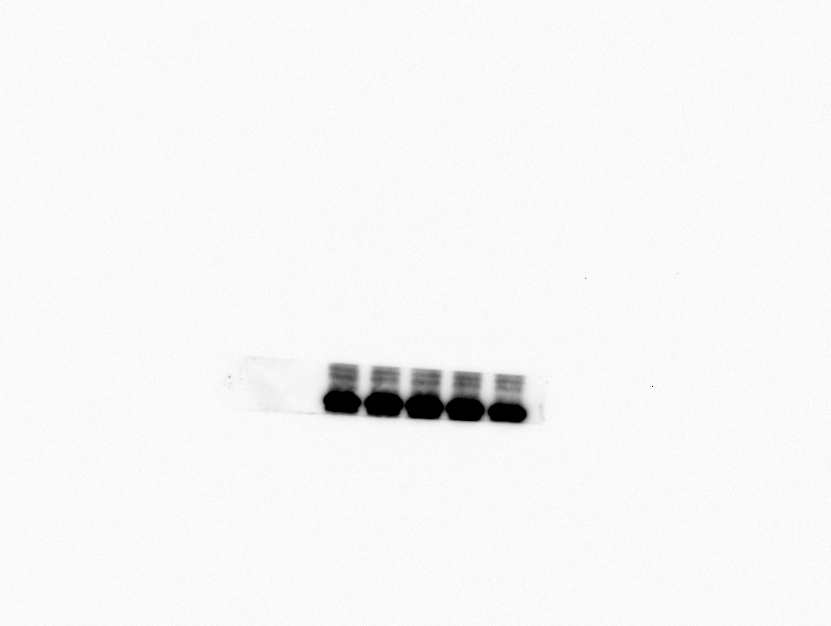

Supplement: Supplementary file 3 [file Data_Sheet_2.ZIP › Proteins for Anti-inflammation of compound 5/P65/contrast/contrast_6.png]

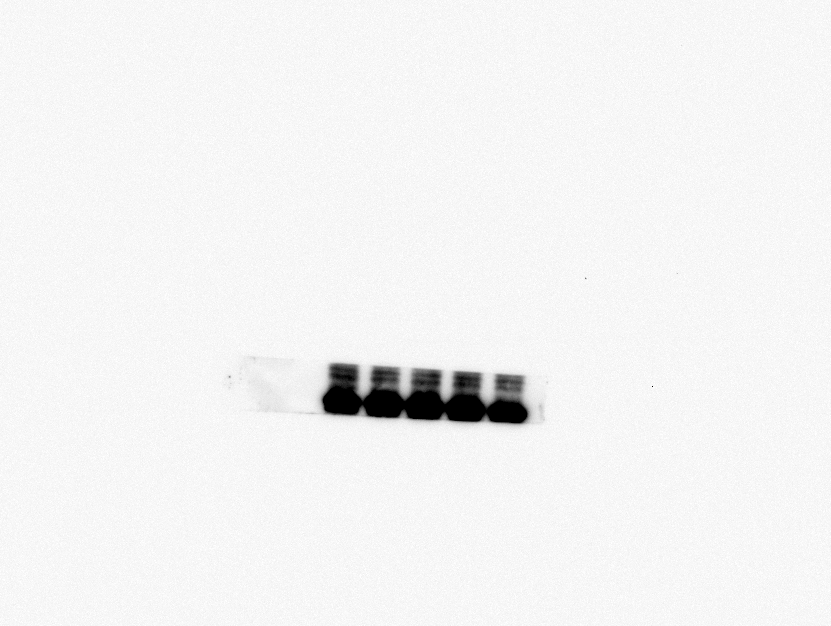

Supplement: Supplementary file 3 [file Data_Sheet_2.ZIP › Proteins for Anti-inflammation of compound 5/P65/contrast/contrast_7.png]

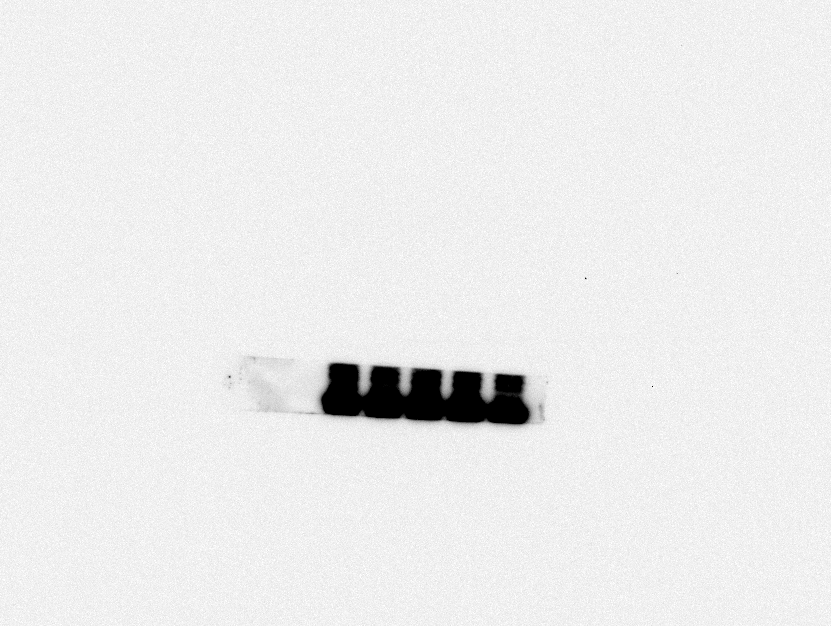

Supplement: Supplementary file 3 [file Data_Sheet_2.ZIP › Proteins for Anti-inflammation of compound 5/P65/contrast/contrast_8.png]

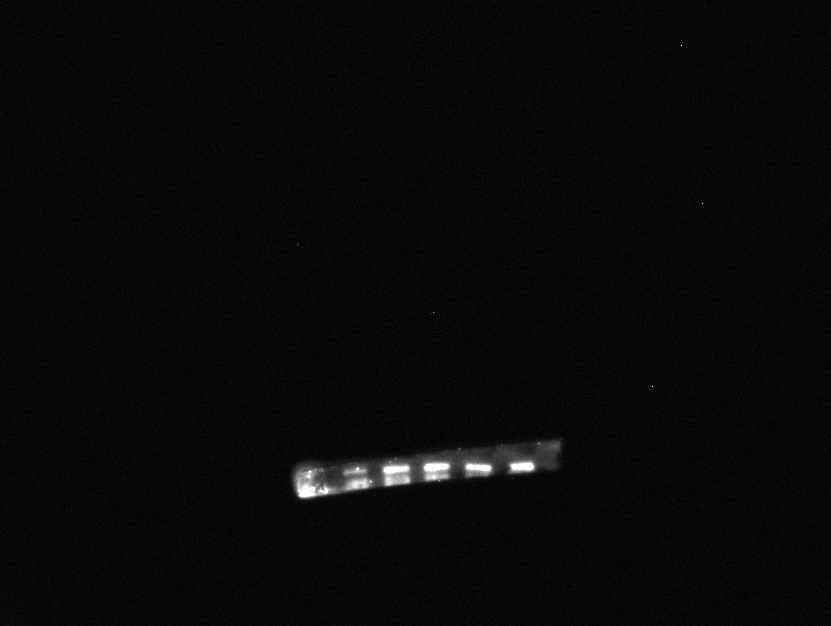

Supplement: Supplementary file 3 [file Data_Sheet_2.ZIP › Proteins for Anti-inflammation of compound 5/iNOS/2021-05-08_1Inos_1_16bit.png]

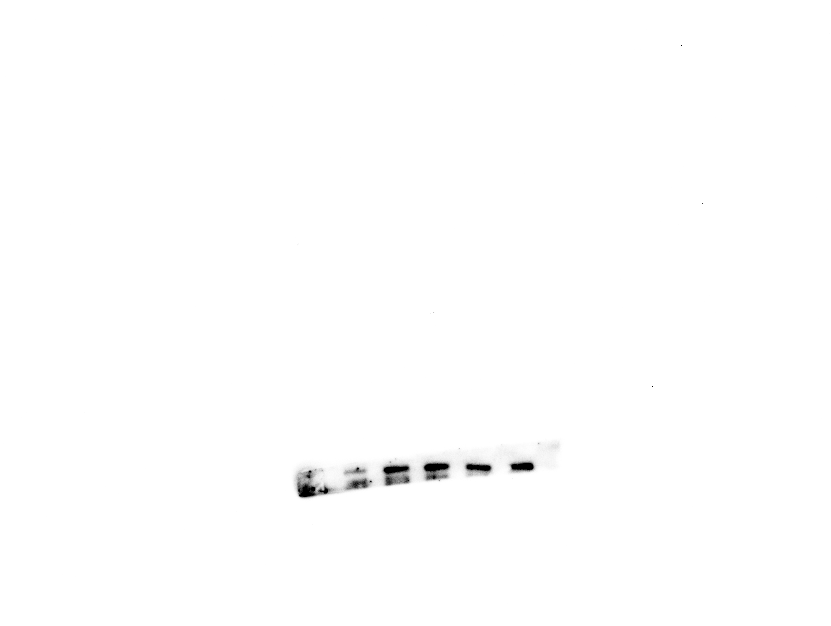

Supplement: Supplementary file 3 [file Data_Sheet_2.ZIP › Proteins for Anti-inflammation of compound 5/iNOS/2021-05-08_1Inos_8bit.png]

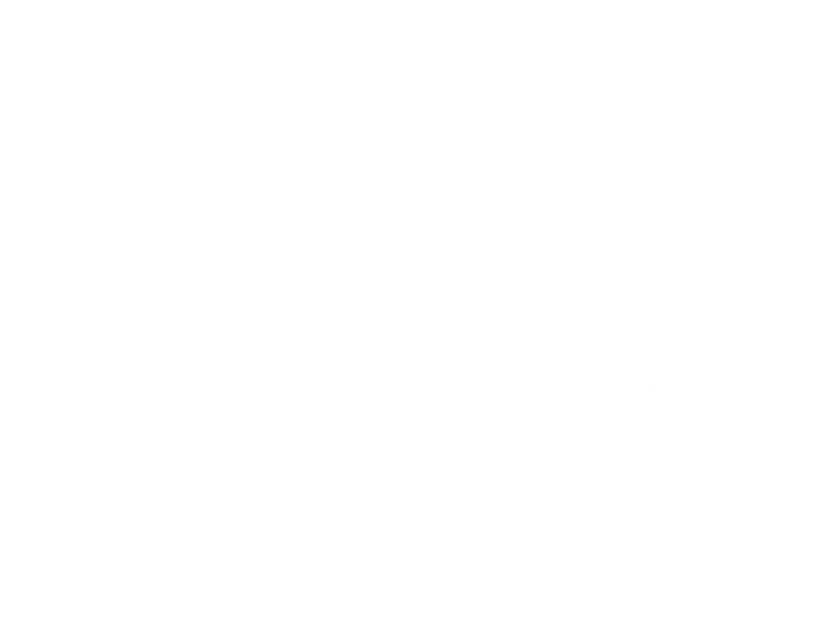

Supplement: Supplementary file 3 [file Data_Sheet_2.ZIP › Proteins for Anti-inflammation of compound 5/iNOS/contrast/contrast_0.png]

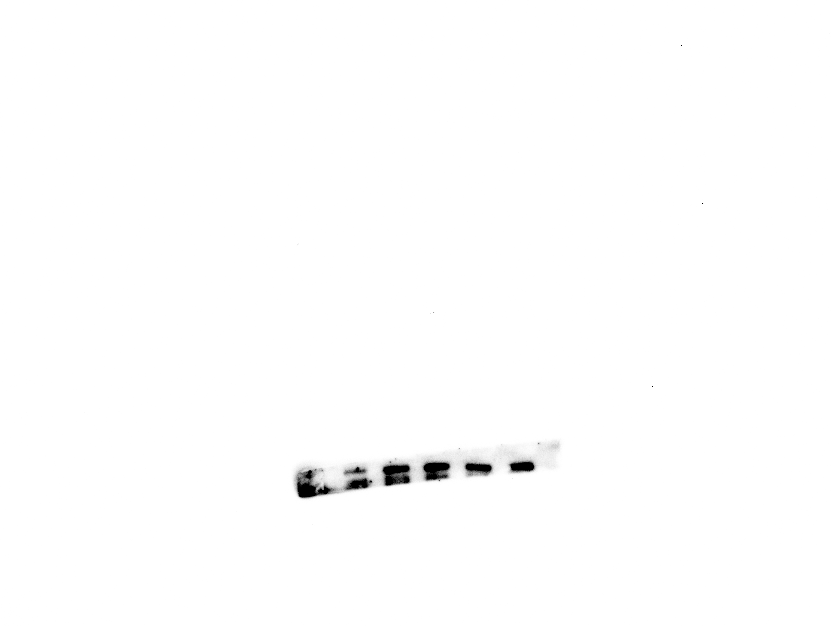

Supplement: Supplementary file 3 [file Data_Sheet_2.ZIP › Proteins for Anti-inflammation of compound 5/iNOS/contrast/contrast_2.png]

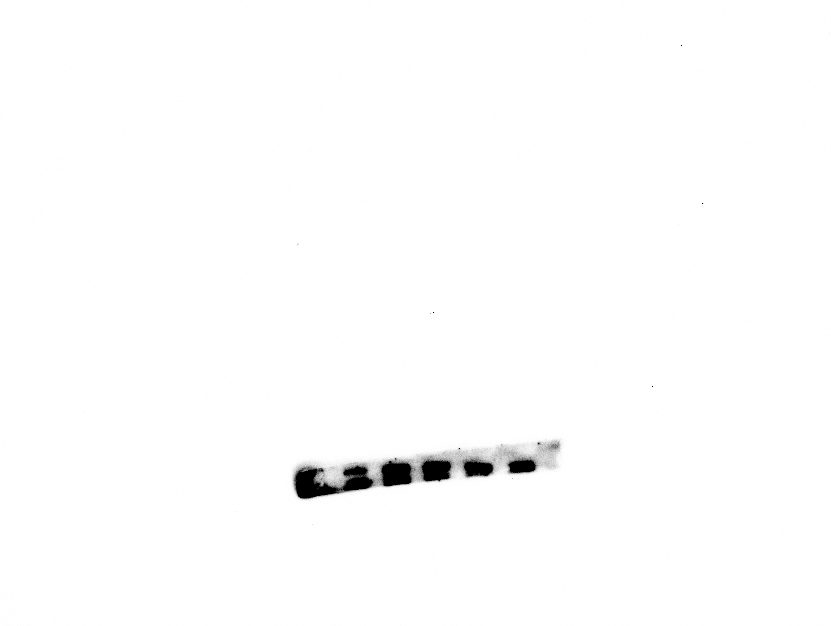

Supplement: Supplementary file 3 [file Data_Sheet_2.ZIP › Proteins for Anti-inflammation of compound 5/iNOS/contrast/contrast_3.png]

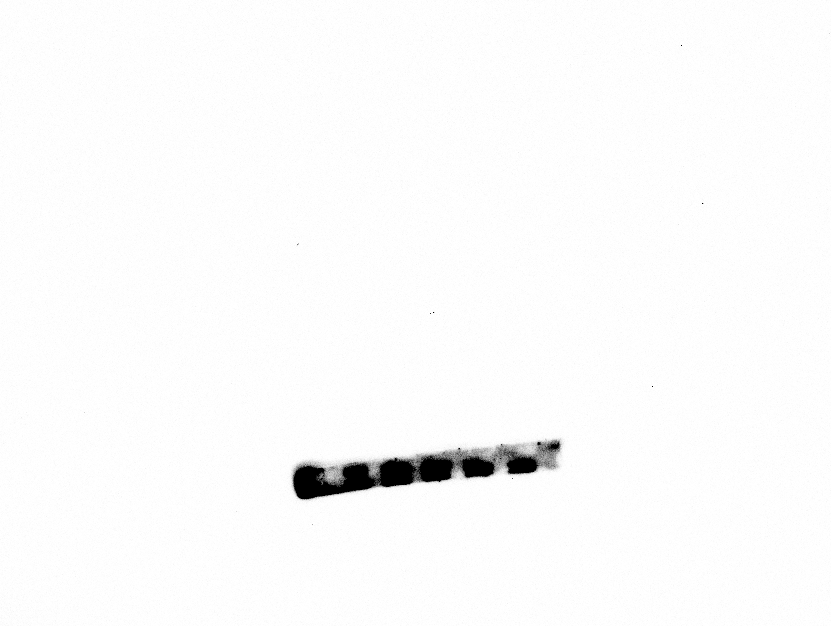

Supplement: Supplementary file 3 [file Data_Sheet_2.ZIP › Proteins for Anti-inflammation of compound 5/iNOS/contrast/contrast_4.png]

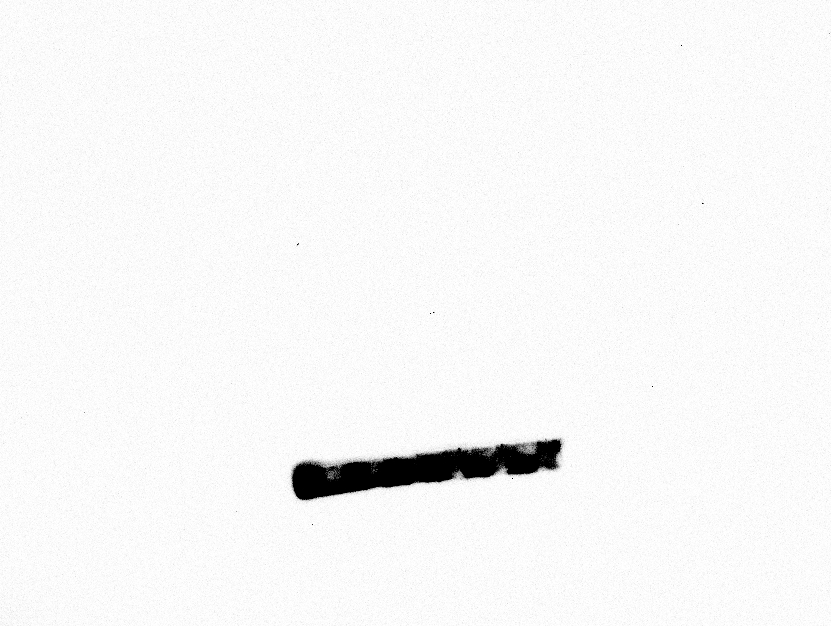

Supplement: Supplementary file 3 [file Data_Sheet_2.ZIP › Proteins for Anti-inflammation of compound 5/iNOS/contrast/contrast_5.png]

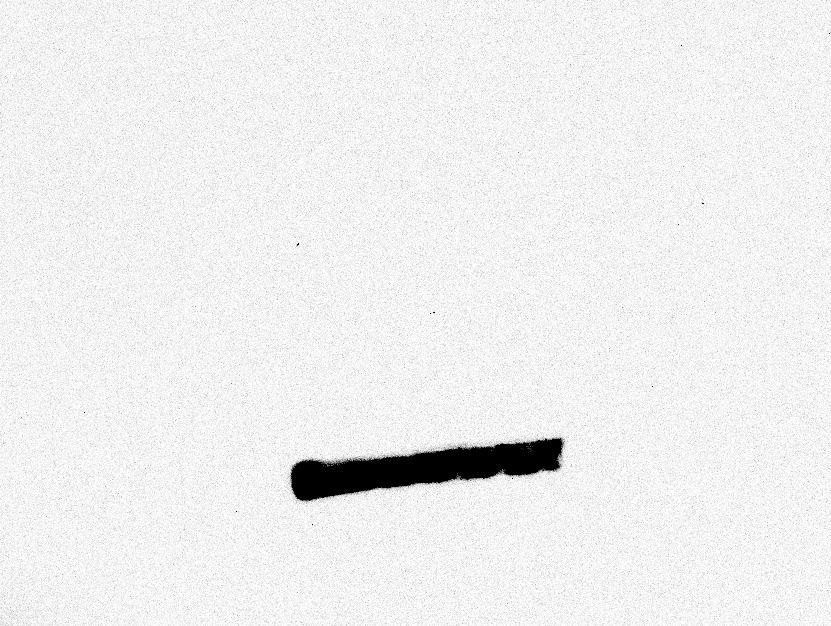

Supplement: Supplementary file 3 [file Data_Sheet_2.ZIP › Proteins for Anti-inflammation of compound 5/iNOS/contrast/contrast_6.png]

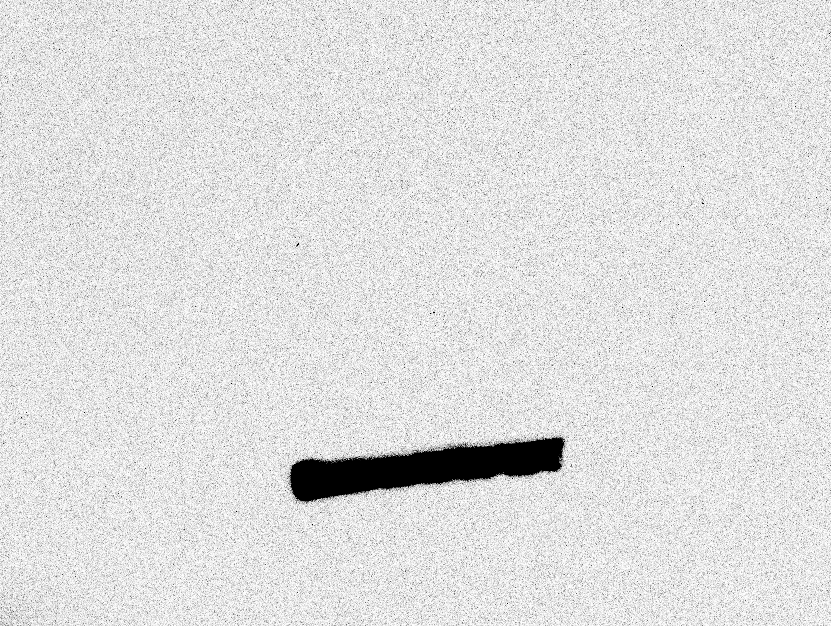

Supplement: Supplementary file 3 [file Data_Sheet_2.ZIP › Proteins for Anti-inflammation of compound 5/iNOS/contrast/contrast_7.png]

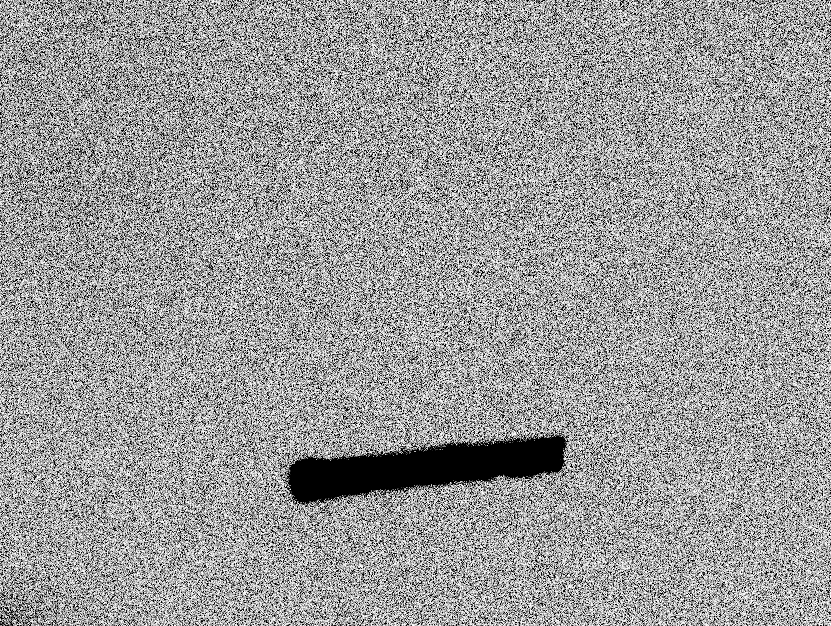

Supplement: Supplementary file 3 [file Data_Sheet_2.ZIP › Proteins for Anti-inflammation of compound 5/iNOS/contrast/contrast_8.png]

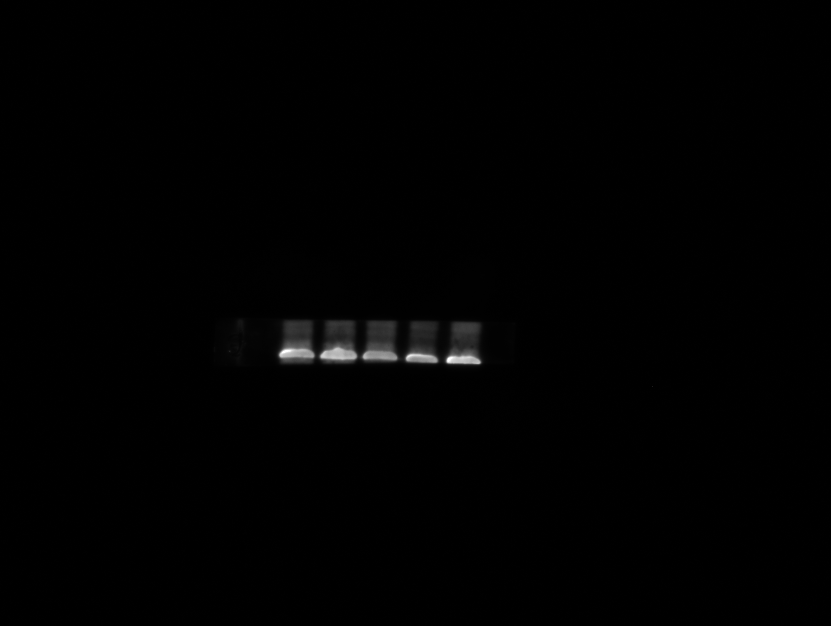

Supplement: Supplementary file 3 [file Data_Sheet_2.ZIP › Proteins for Anti-inflammation of compound 5/a┬-actin/2021-05-07_22-05-37B12_1_16bit.png]

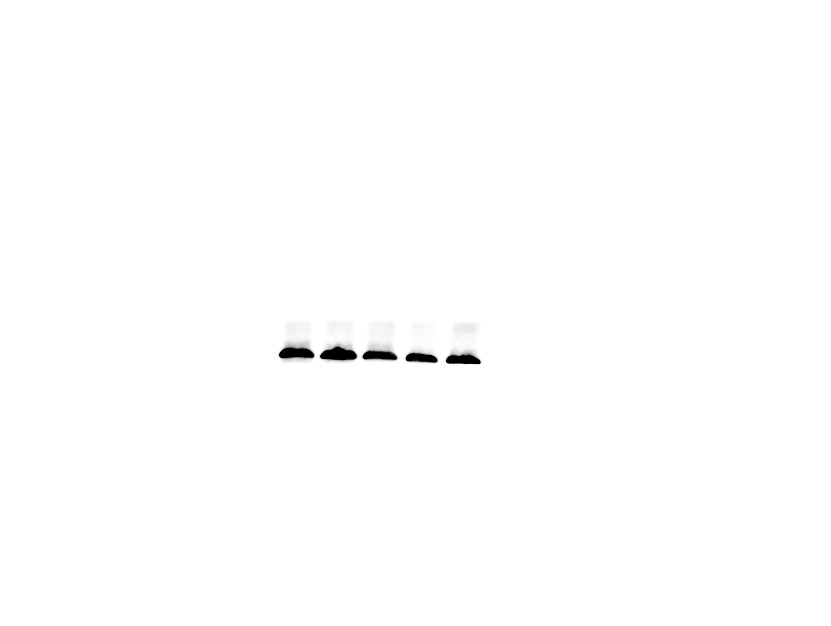

Supplement: Supplementary file 3 [file Data_Sheet_2.ZIP › Proteins for Anti-inflammation of compound 5/a┬-actin/2021-05-07_22-05-37B12_8bit.png]

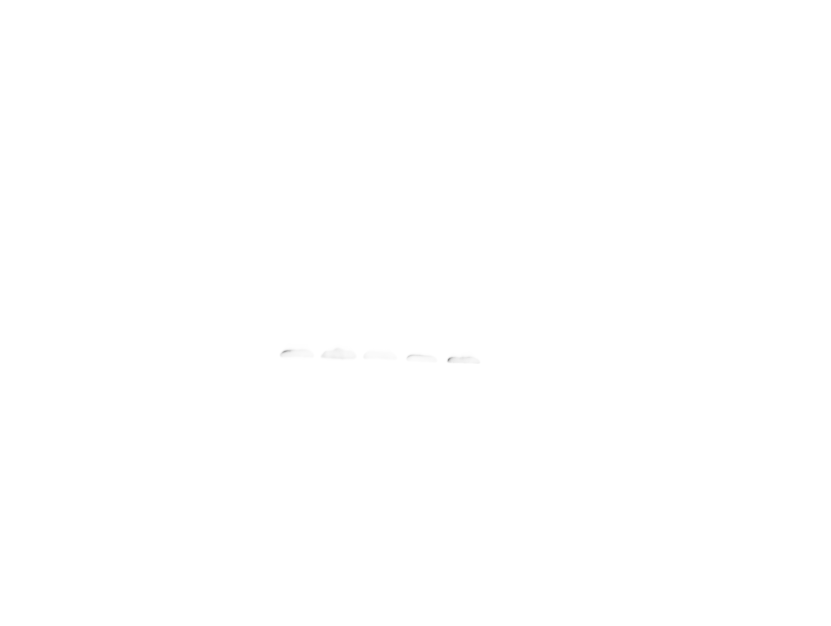

Supplement: Supplementary file 3 [file Data_Sheet_2.ZIP › Proteins for Anti-inflammation of compound 5/a┬-actin/contrast/contrast_0.png]

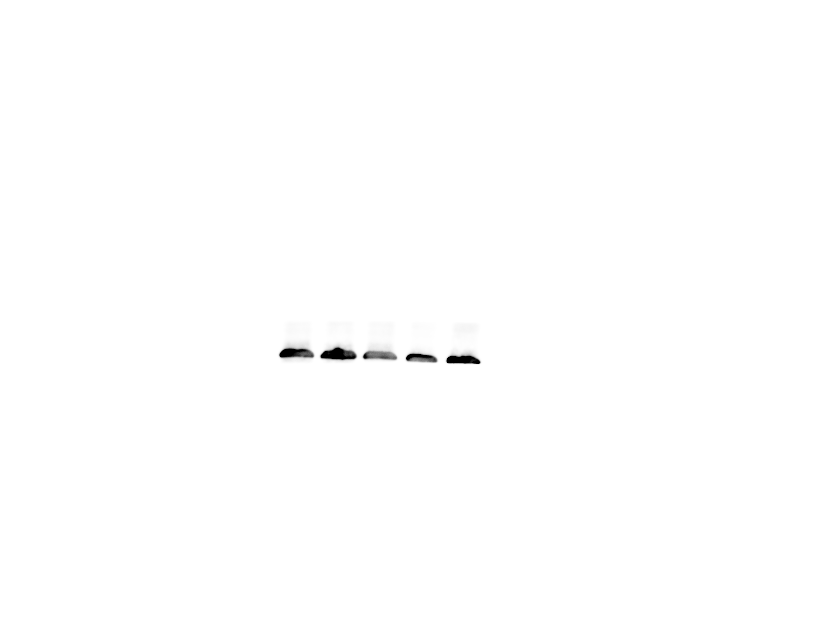

Supplement: Supplementary file 3 [file Data_Sheet_2.ZIP › Proteins for Anti-inflammation of compound 5/a┬-actin/contrast/contrast_1.png]

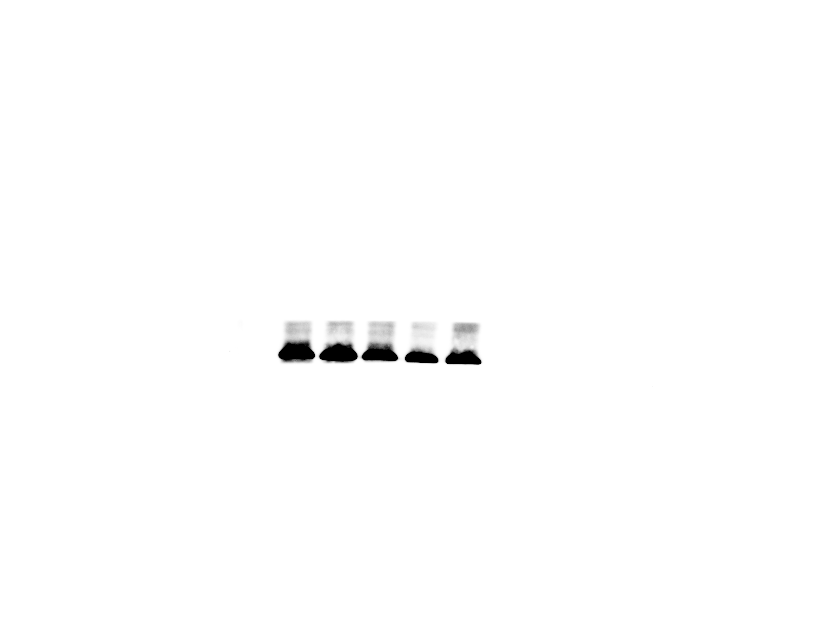

Supplement: Supplementary file 3 [file Data_Sheet_2.ZIP › Proteins for Anti-inflammation of compound 5/a┬-actin/contrast/contrast_3.png]

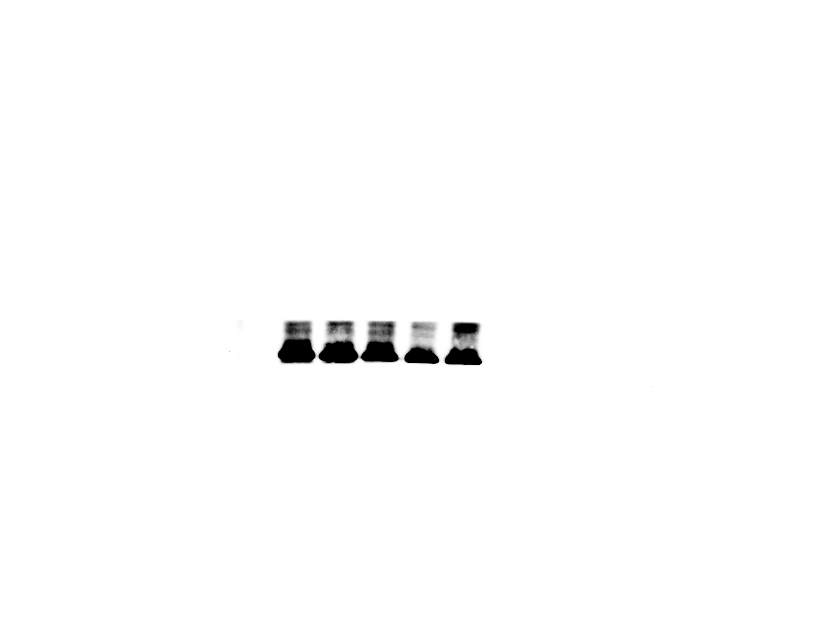

Supplement: Supplementary file 3 [file Data_Sheet_2.ZIP › Proteins for Anti-inflammation of compound 5/a┬-actin/contrast/contrast_4.png]

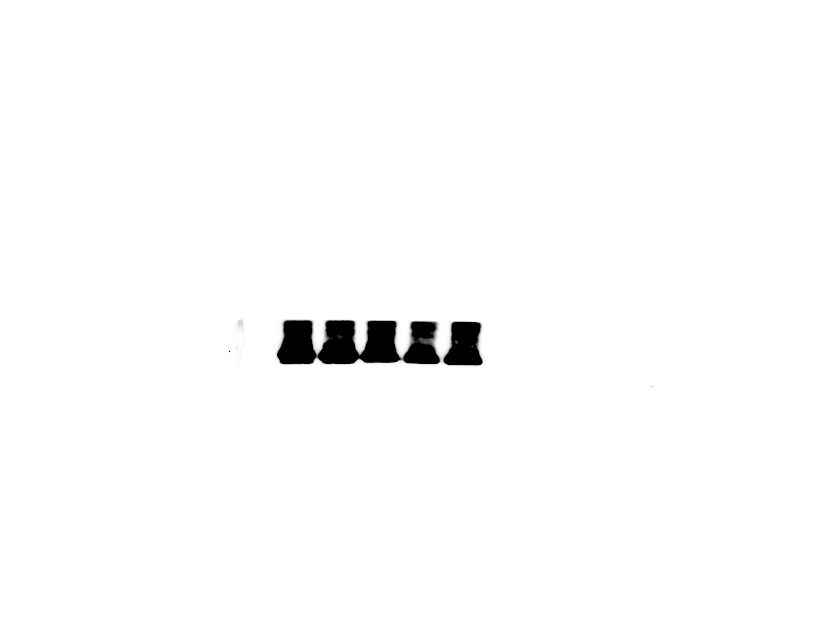

Supplement: Supplementary file 3 [file Data_Sheet_2.ZIP › Proteins for Anti-inflammation of compound 5/a┬-actin/contrast/contrast_5.png]

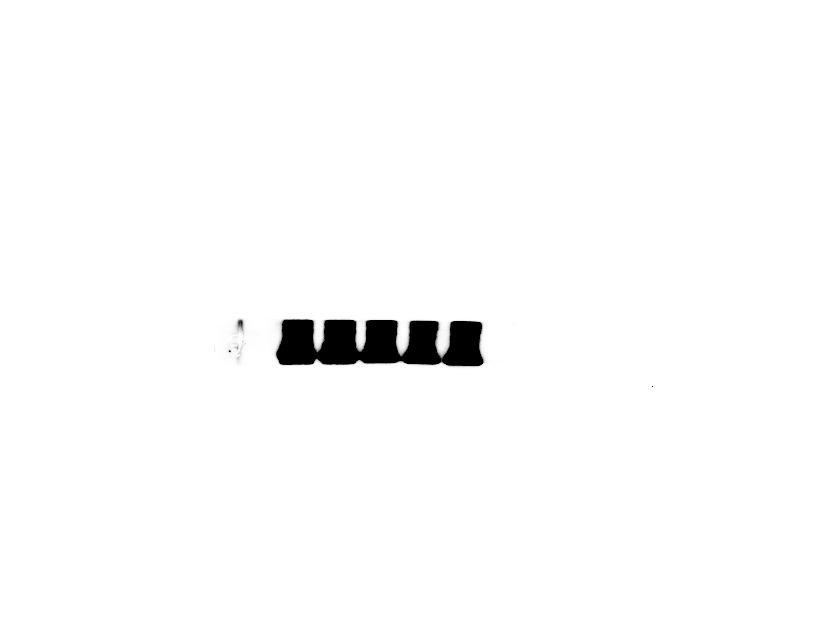

Supplement: Supplementary file 3 [file Data_Sheet_2.ZIP › Proteins for Anti-inflammation of compound 5/a┬-actin/contrast/contrast_6.png]

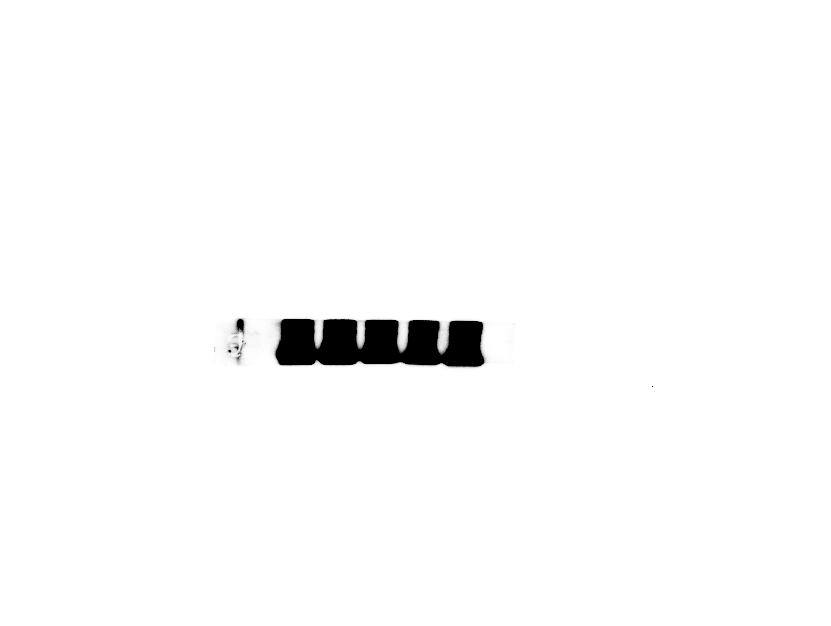

Supplement: Supplementary file 3 [file Data_Sheet_2.ZIP › Proteins for Anti-inflammation of compound 5/a┬-actin/contrast/contrast_7.png]

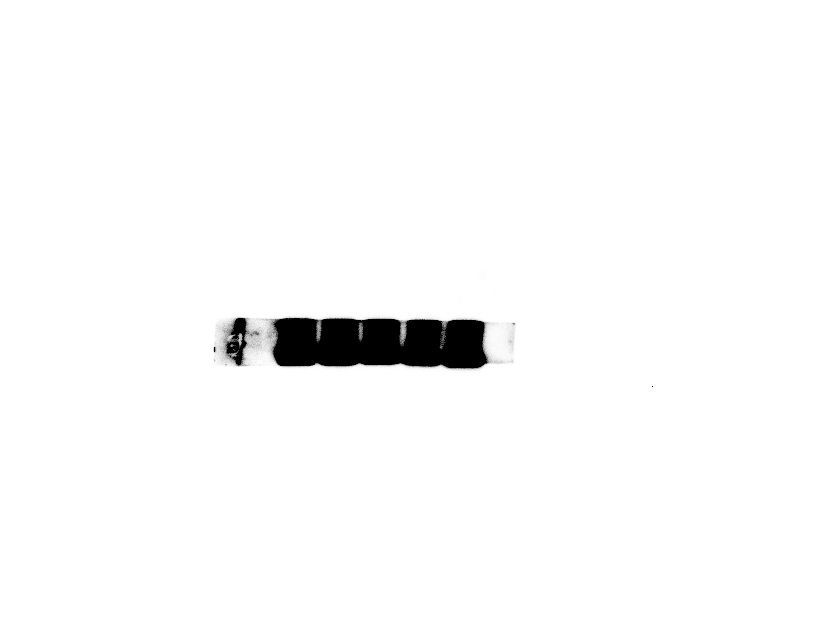

Supplement: Supplementary file 3 [file Data_Sheet_2.ZIP › Proteins for Anti-inflammation of compound 5/a┬-actin/contrast/contrast_8.png]

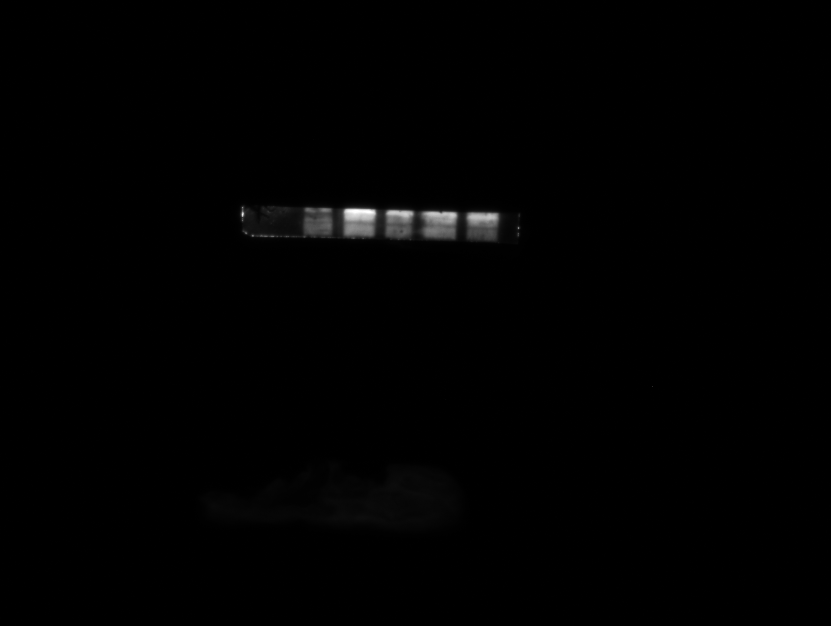

Supplement: Supplementary file 4 [file Data_Sheet_3.ZIP › Proteins for Anti-inflammation of compound 8/COX-2/2021-05-08_cox2_1_16bit.png]

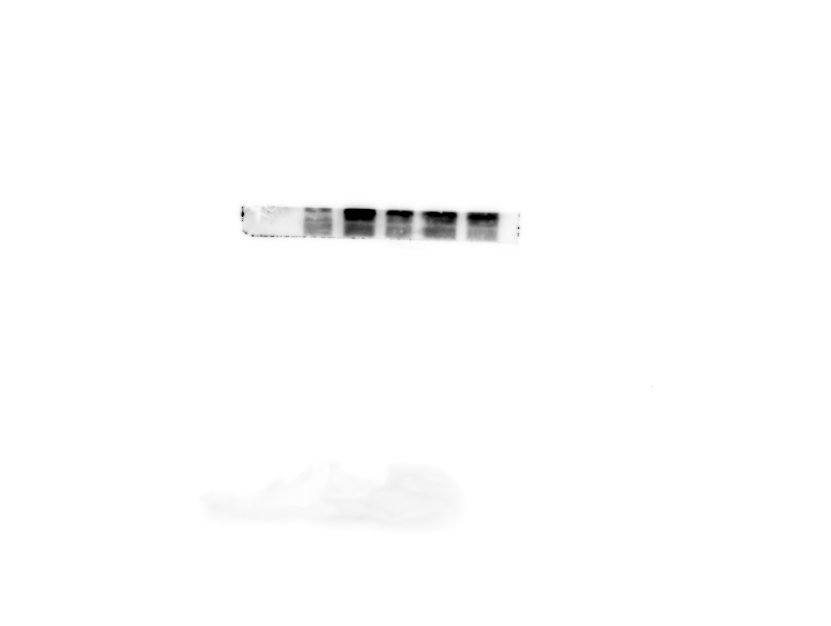

Supplement: Supplementary file 4 [file Data_Sheet_3.ZIP › Proteins for Anti-inflammation of compound 8/COX-2/2021-05-08_cox2_8bit.png]

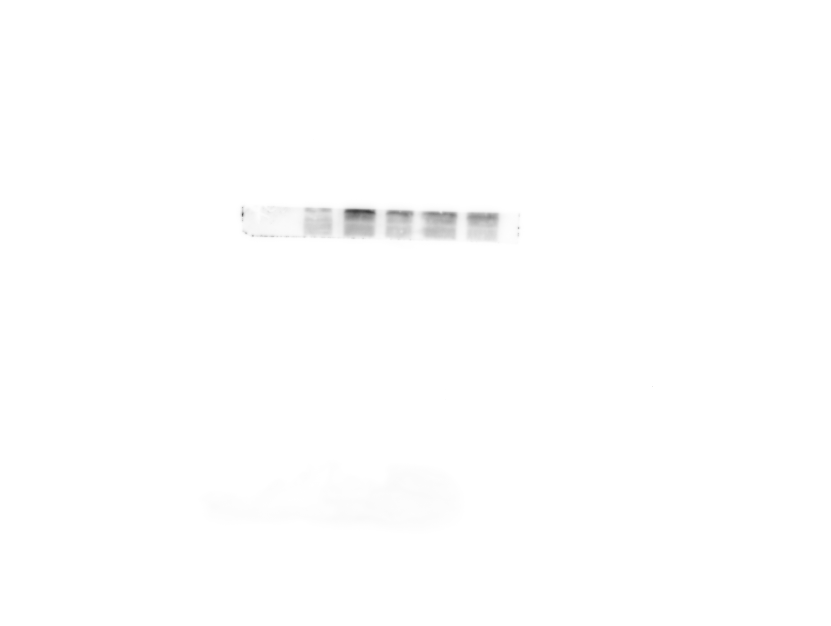

Supplement: Supplementary file 4 [file Data_Sheet_3.ZIP › Proteins for Anti-inflammation of compound 8/COX-2/contrast/contrast_0.png]

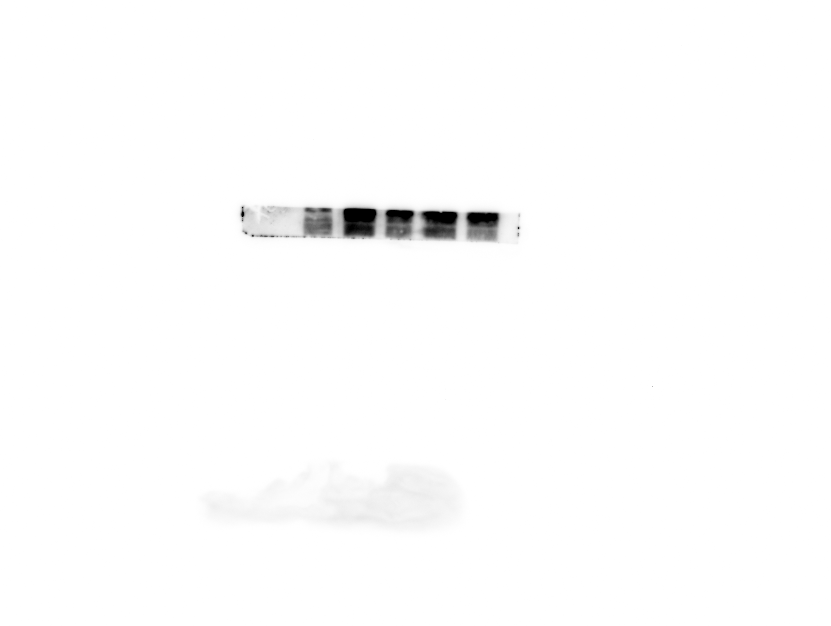

Supplement: Supplementary file 4 [file Data_Sheet_3.ZIP › Proteins for Anti-inflammation of compound 8/COX-2/contrast/contrast_2.png]

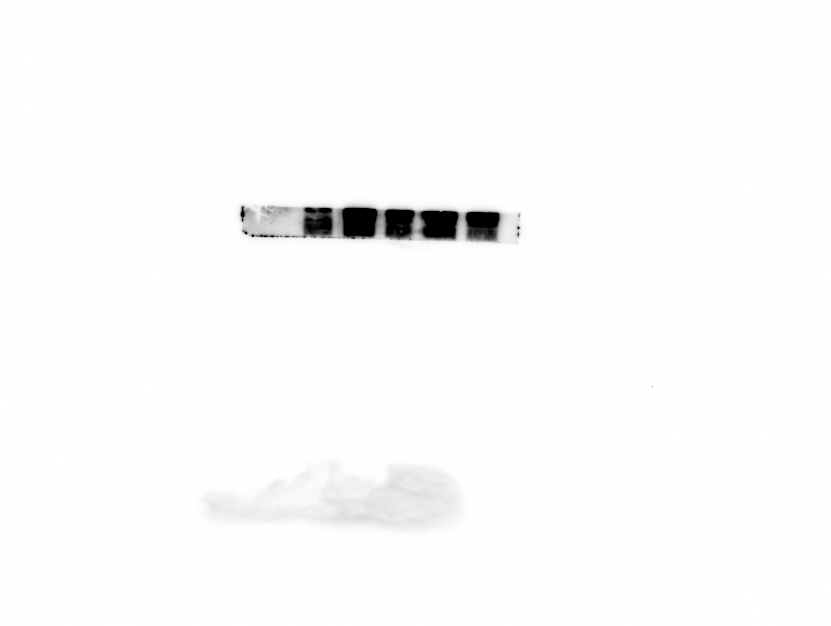

Supplement: Supplementary file 4 [file Data_Sheet_3.ZIP › Proteins for Anti-inflammation of compound 8/COX-2/contrast/contrast_3.png]

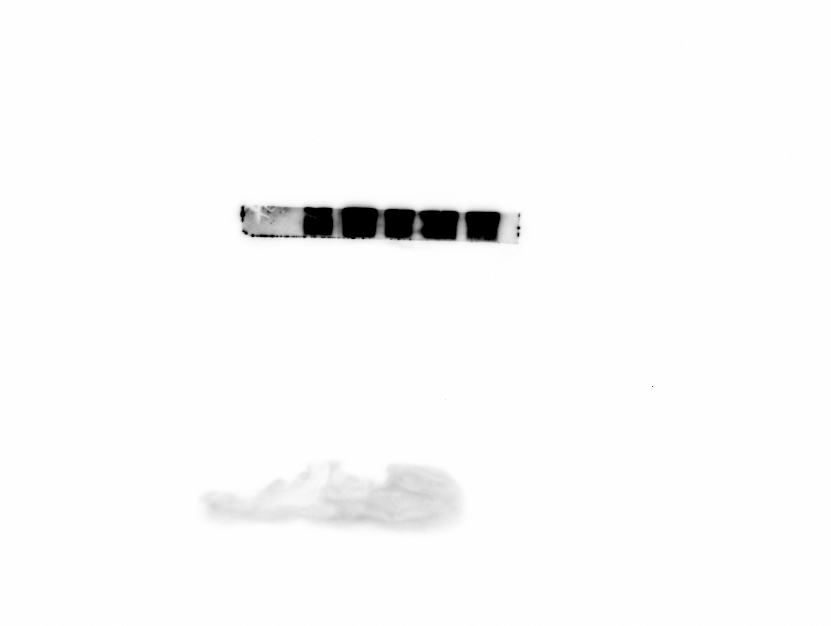

Supplement: Supplementary file 4 [file Data_Sheet_3.ZIP › Proteins for Anti-inflammation of compound 8/COX-2/contrast/contrast_4.png]

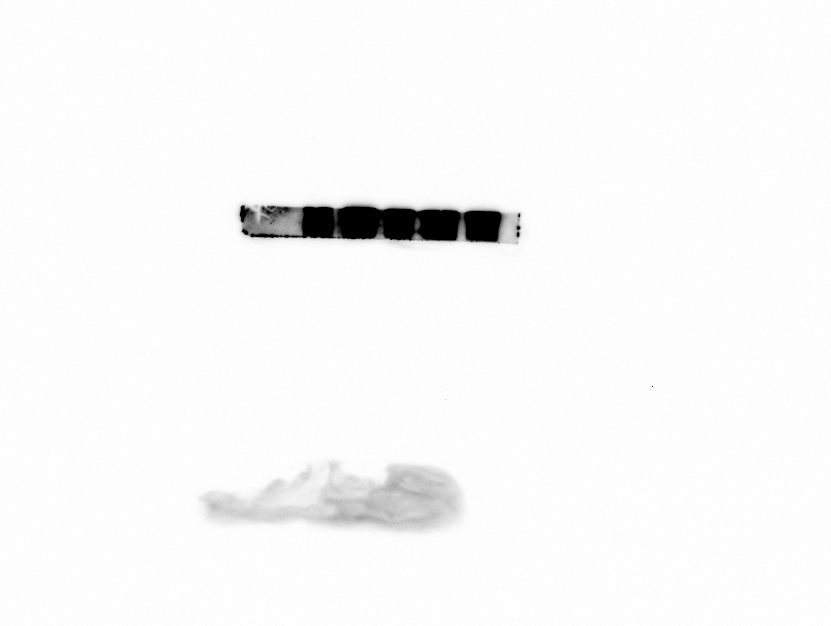

Supplement: Supplementary file 4 [file Data_Sheet_3.ZIP › Proteins for Anti-inflammation of compound 8/COX-2/contrast/contrast_5.png]

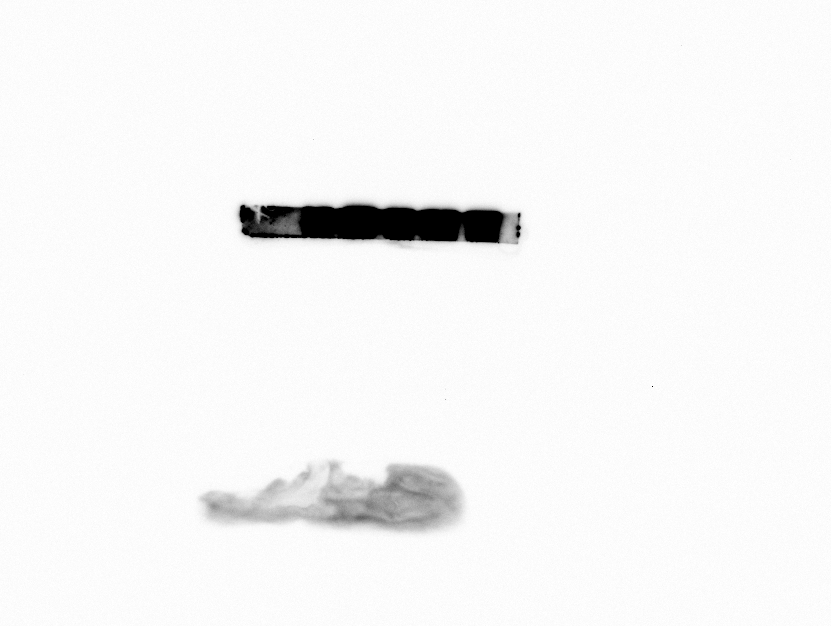

Supplement: Supplementary file 4 [file Data_Sheet_3.ZIP › Proteins for Anti-inflammation of compound 8/COX-2/contrast/contrast_6.png]

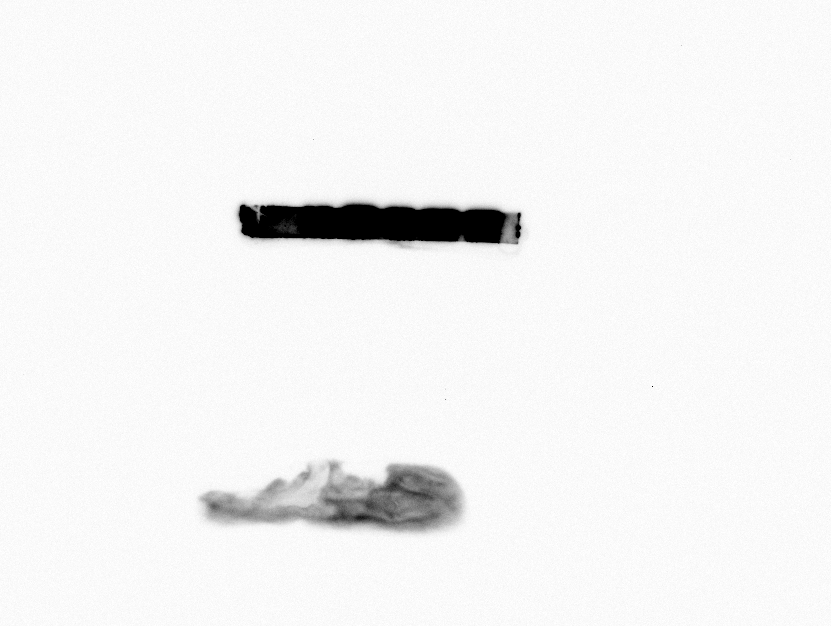

Supplement: Supplementary file 4 [file Data_Sheet_3.ZIP › Proteins for Anti-inflammation of compound 8/COX-2/contrast/contrast_7.png]

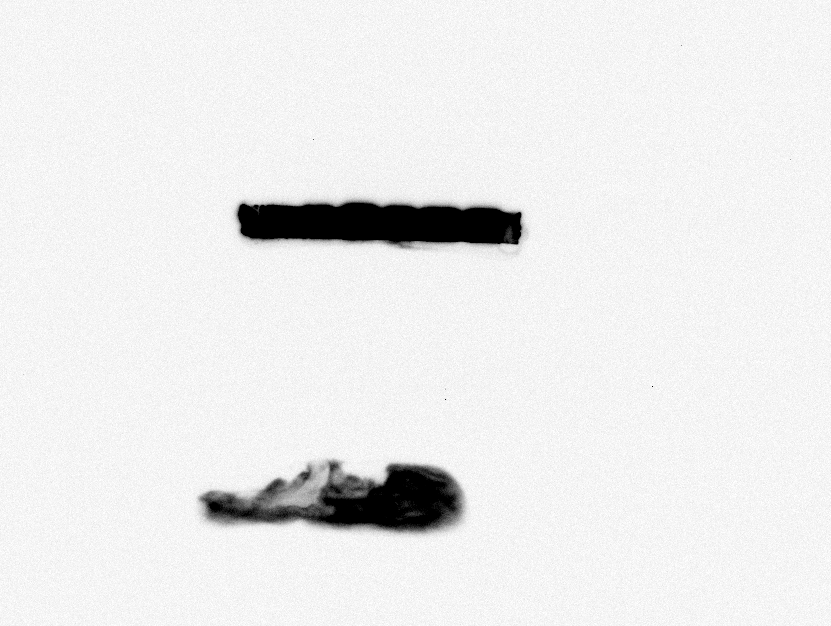

Supplement: Supplementary file 4 [file Data_Sheet_3.ZIP › Proteins for Anti-inflammation of compound 8/COX-2/contrast/contrast_8.png]

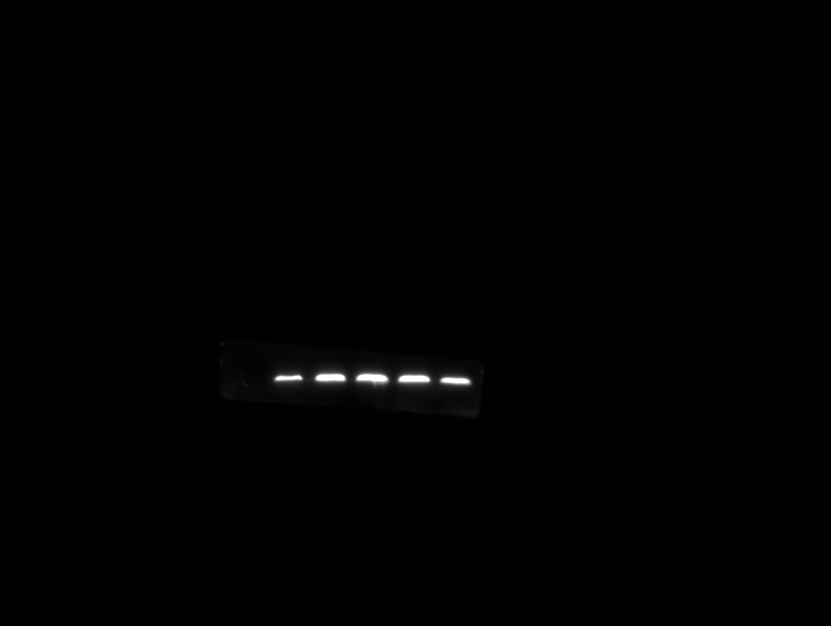

Supplement: Supplementary file 4 [file Data_Sheet_3.ZIP › Proteins for Anti-inflammation of compound 8/Ia╩Ba┴/2021-05-08_Ikba_1_16bit.png]

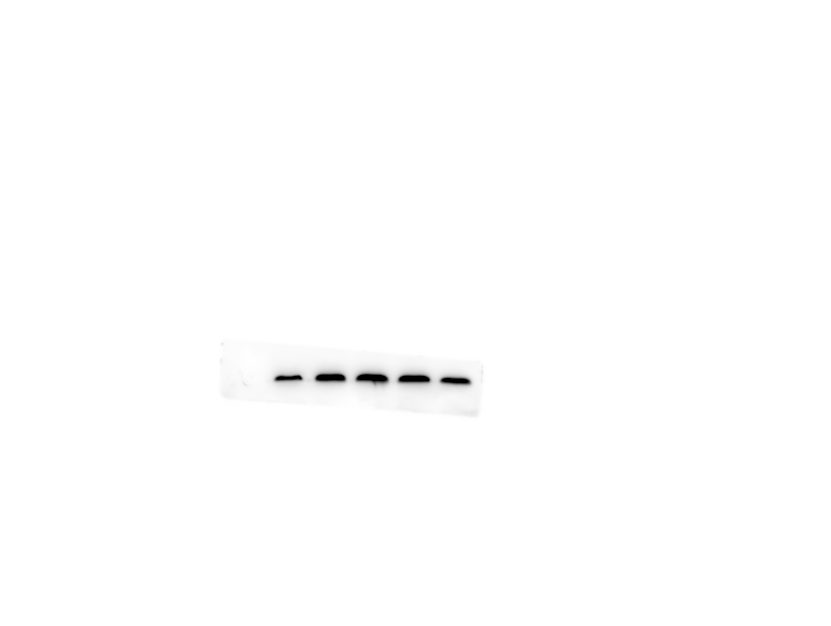

Supplement: Supplementary file 4 [file Data_Sheet_3.ZIP › Proteins for Anti-inflammation of compound 8/Ia╩Ba┴/2021-05-08_Ikba_8bit.png]

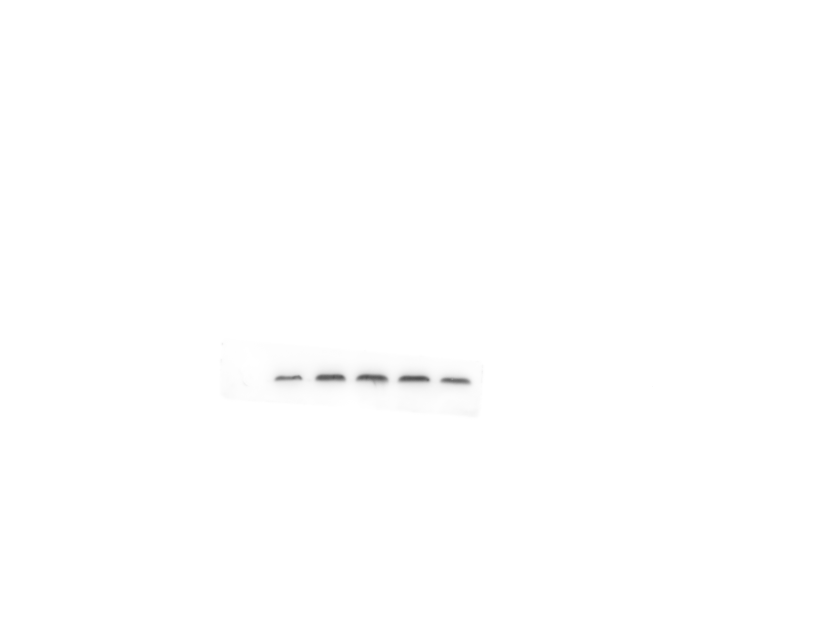

Supplement: Supplementary file 4 [file Data_Sheet_3.ZIP › Proteins for Anti-inflammation of compound 8/Ia╩Ba┴/contrast/contrast_0.png]

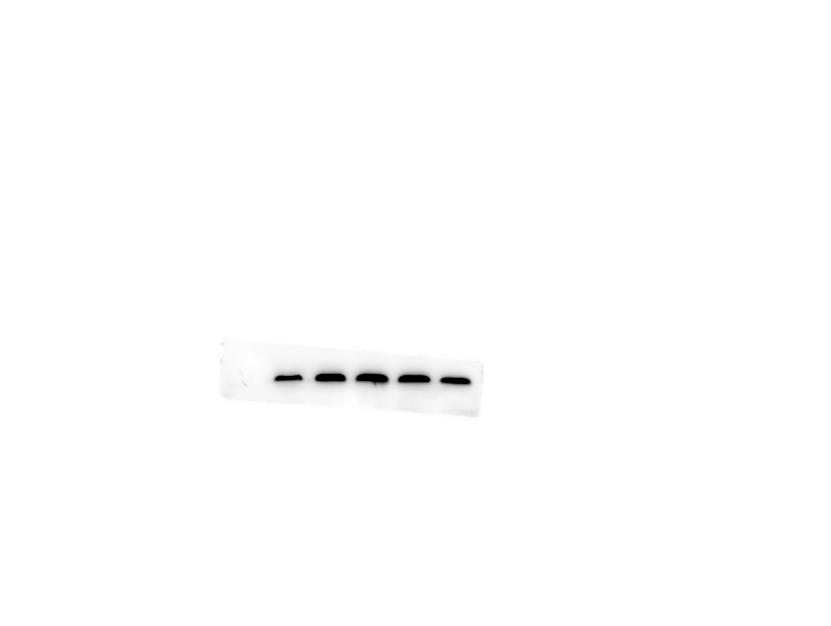

Supplement: Supplementary file 4 [file Data_Sheet_3.ZIP › Proteins for Anti-inflammation of compound 8/Ia╩Ba┴/contrast/contrast_2.png]

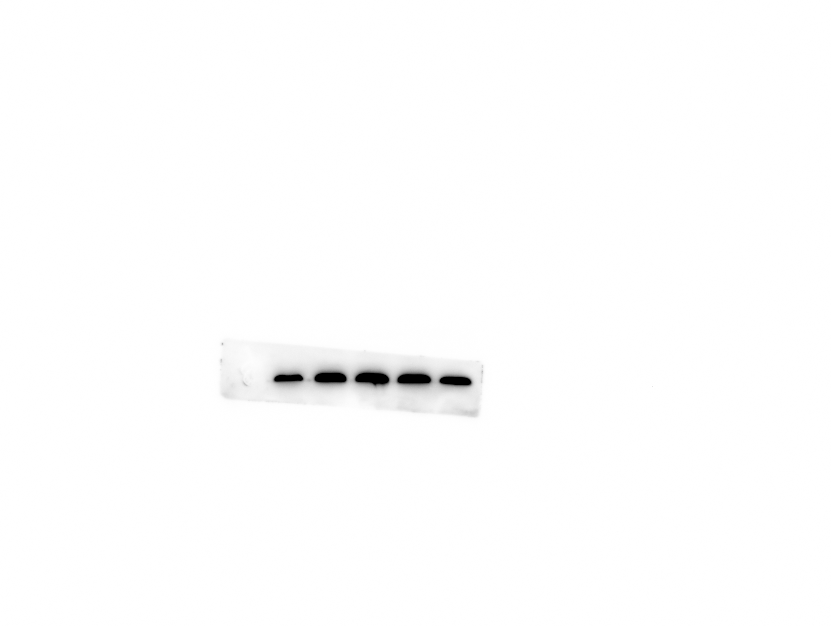

Supplement: Supplementary file 4 [file Data_Sheet_3.ZIP › Proteins for Anti-inflammation of compound 8/Ia╩Ba┴/contrast/contrast_3.png]

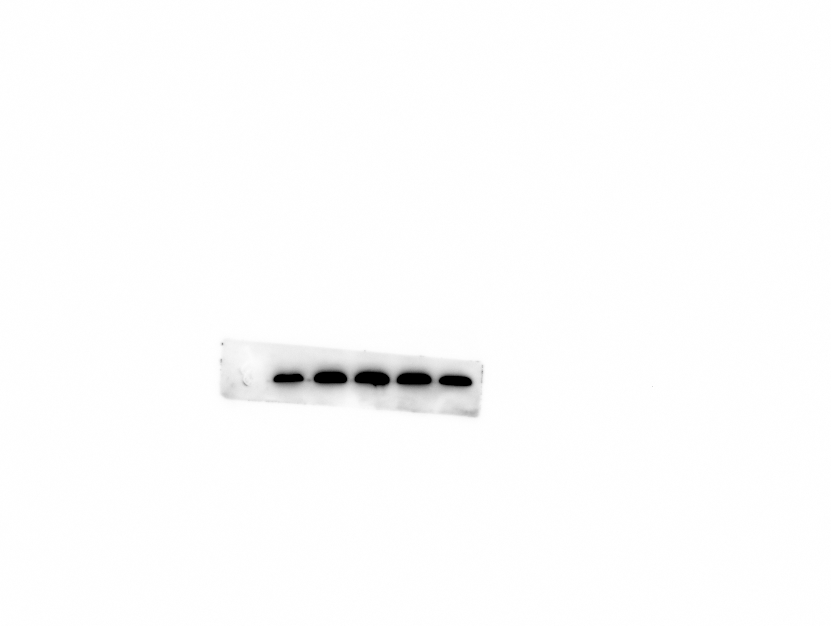

Supplement: Supplementary file 4 [file Data_Sheet_3.ZIP › Proteins for Anti-inflammation of compound 8/Ia╩Ba┴/contrast/contrast_4.png]

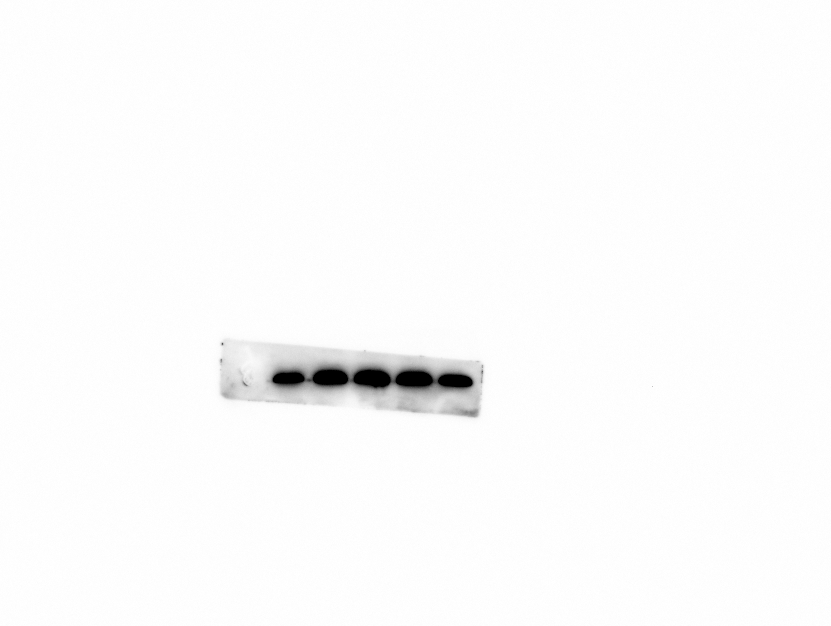

Supplement: Supplementary file 4 [file Data_Sheet_3.ZIP › Proteins for Anti-inflammation of compound 8/Ia╩Ba┴/contrast/contrast_5.png]

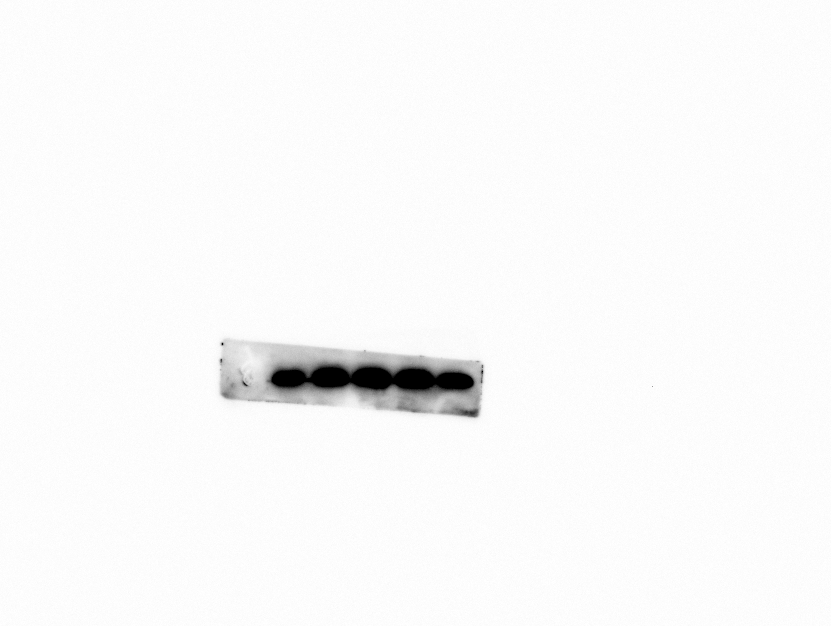

Supplement: Supplementary file 4 [file Data_Sheet_3.ZIP › Proteins for Anti-inflammation of compound 8/Ia╩Ba┴/contrast/contrast_6.png]

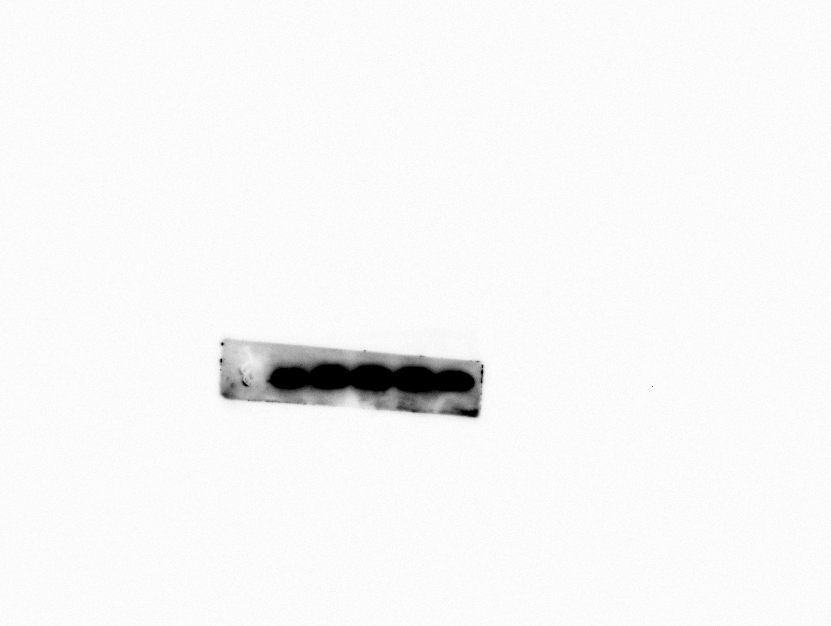

Supplement: Supplementary file 4 [file Data_Sheet_3.ZIP › Proteins for Anti-inflammation of compound 8/Ia╩Ba┴/contrast/contrast_7.png]

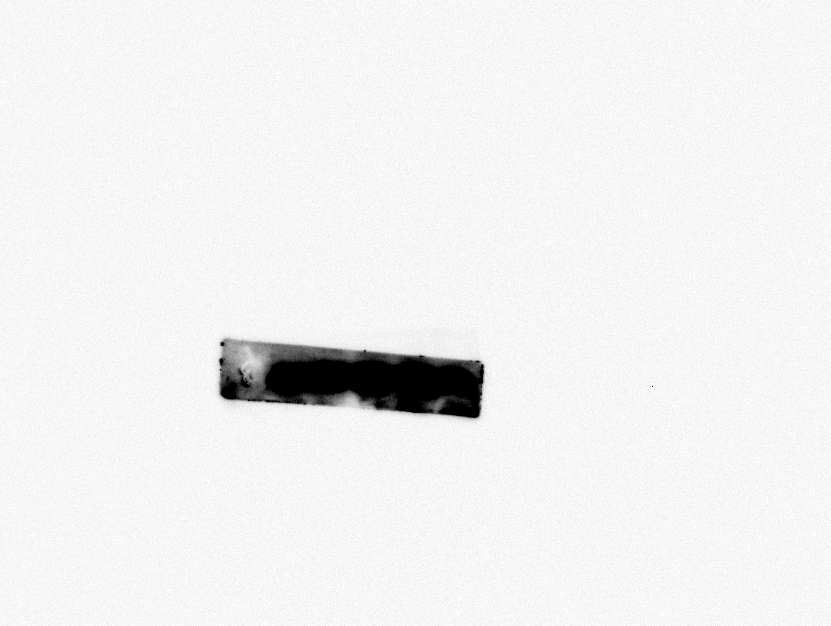

Supplement: Supplementary file 4 [file Data_Sheet_3.ZIP › Proteins for Anti-inflammation of compound 8/Ia╩Ba┴/contrast/contrast_8.png]

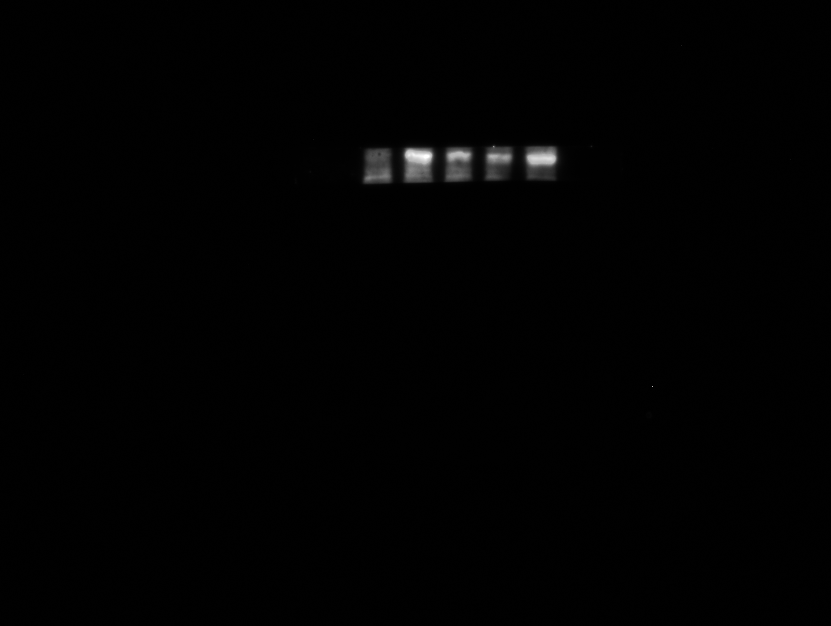

Supplement: Supplementary file 4 [file Data_Sheet_3.ZIP › Proteins for Anti-inflammation of compound 8/P-Ia╩B/2021-04-30_Pikba_1_16bit.png]

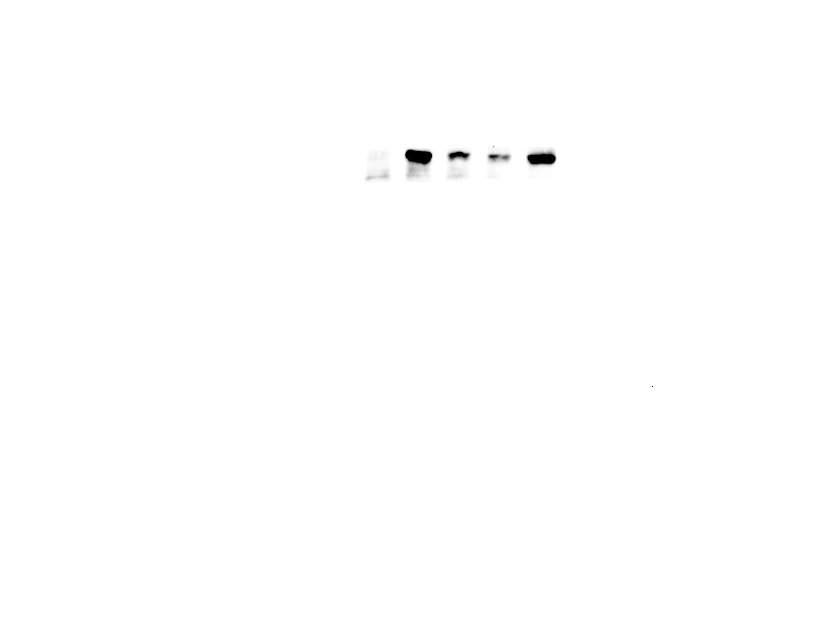

Supplement: Supplementary file 4 [file Data_Sheet_3.ZIP › Proteins for Anti-inflammation of compound 8/P-Ia╩B/2021-04-30_Pikba_8bit.png]

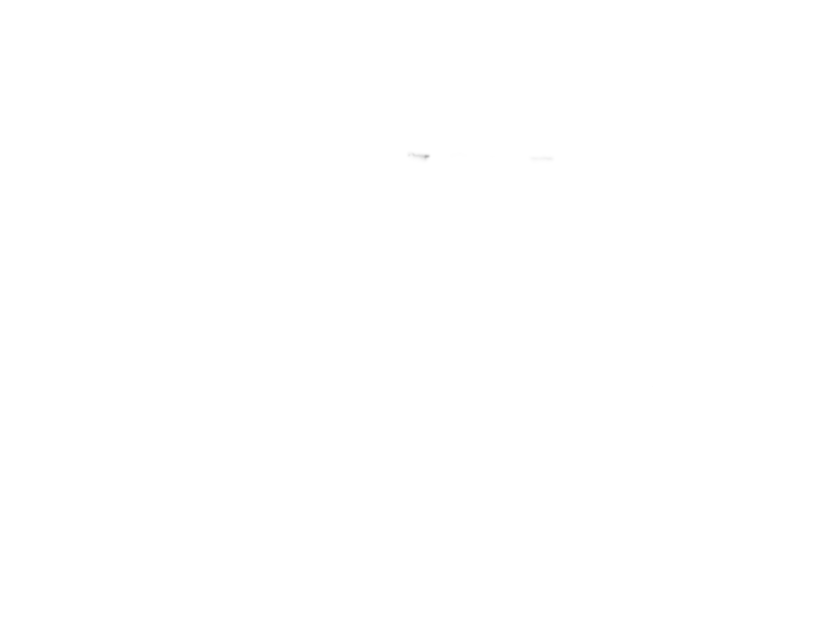

Supplement: Supplementary file 4 [file Data_Sheet_3.ZIP › Proteins for Anti-inflammation of compound 8/P-Ia╩B/contrast/contrast_0.png]

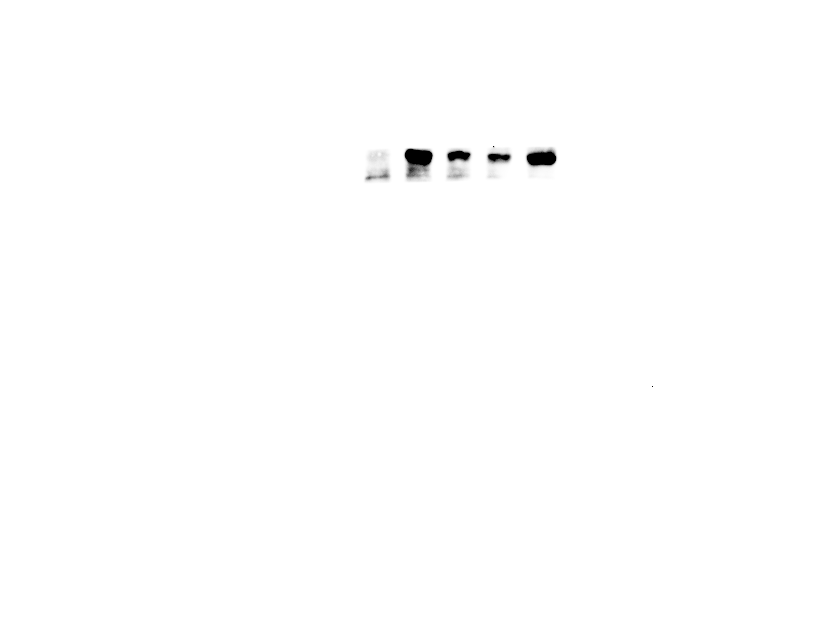

Supplement: Supplementary file 4 [file Data_Sheet_3.ZIP › Proteins for Anti-inflammation of compound 8/P-Ia╩B/contrast/contrast_2.png]

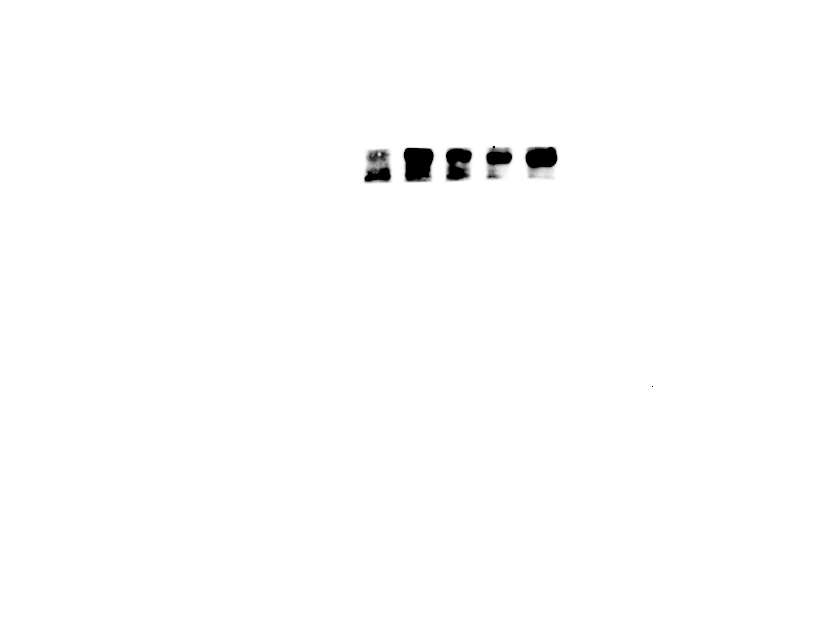

Supplement: Supplementary file 4 [file Data_Sheet_3.ZIP › Proteins for Anti-inflammation of compound 8/P-Ia╩B/contrast/contrast_3.png]

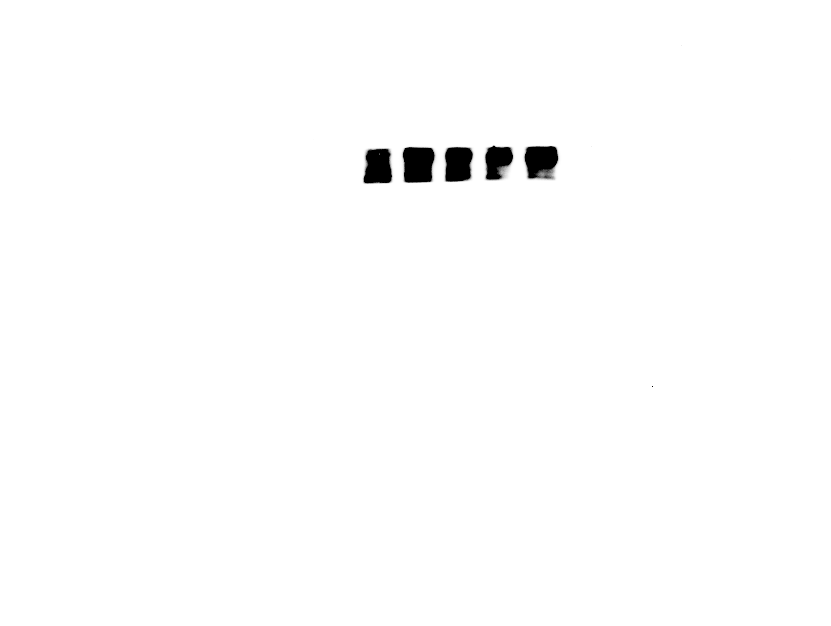

Supplement: Supplementary file 4 [file Data_Sheet_3.ZIP › Proteins for Anti-inflammation of compound 8/P-Ia╩B/contrast/contrast_4.png]

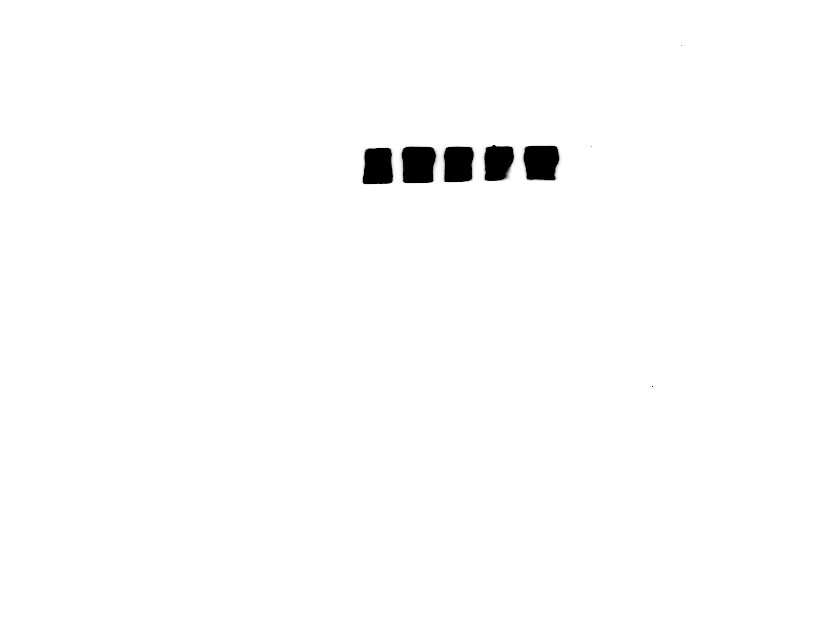

Supplement: Supplementary file 4 [file Data_Sheet_3.ZIP › Proteins for Anti-inflammation of compound 8/P-Ia╩B/contrast/contrast_5.png]

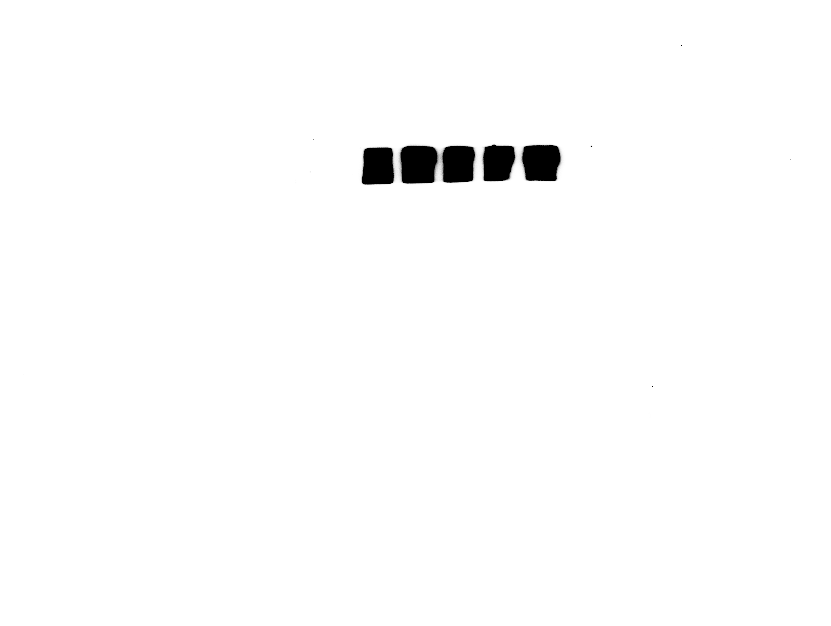

Supplement: Supplementary file 4 [file Data_Sheet_3.ZIP › Proteins for Anti-inflammation of compound 8/P-Ia╩B/contrast/contrast_6.png]

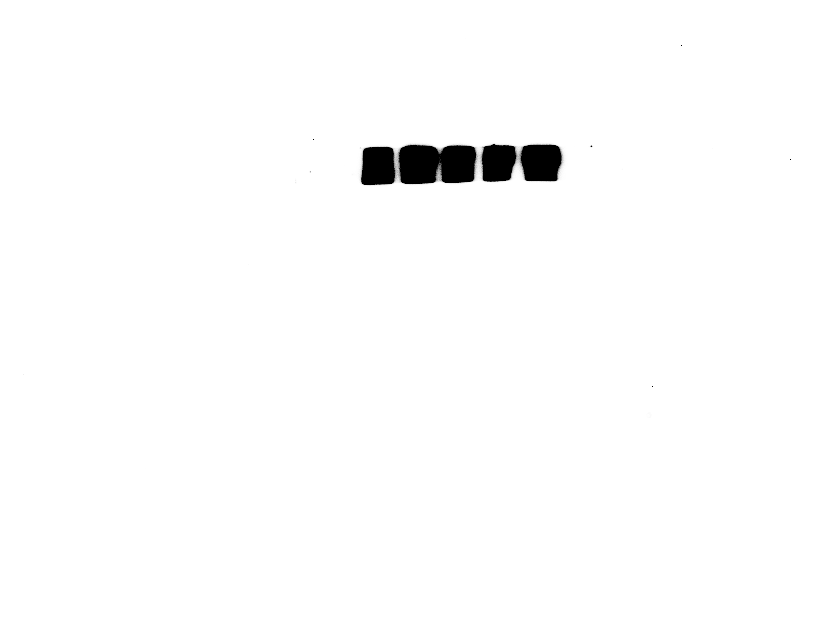

Supplement: Supplementary file 4 [file Data_Sheet_3.ZIP › Proteins for Anti-inflammation of compound 8/P-Ia╩B/contrast/contrast_7.png]

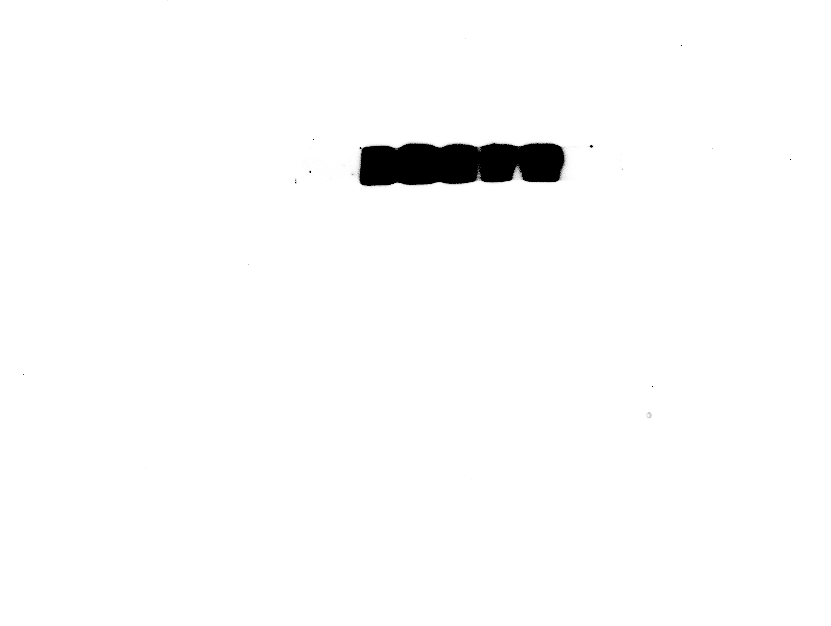

Supplement: Supplementary file 4 [file Data_Sheet_3.ZIP › Proteins for Anti-inflammation of compound 8/P-Ia╩B/contrast/contrast_8.png]
