# Supplementary figures and images for: Nigella sativa: A Dietary Supplement as an Immune-Modulator on the Basis of Bioactive Components (part 2 of 3)
Source: Front Nutr. 2021 Aug 17;8:722813. doi: 10.3389/fnut.2021.722813 (PMC8415885; doi:10.3389/fnut.2021.722813)

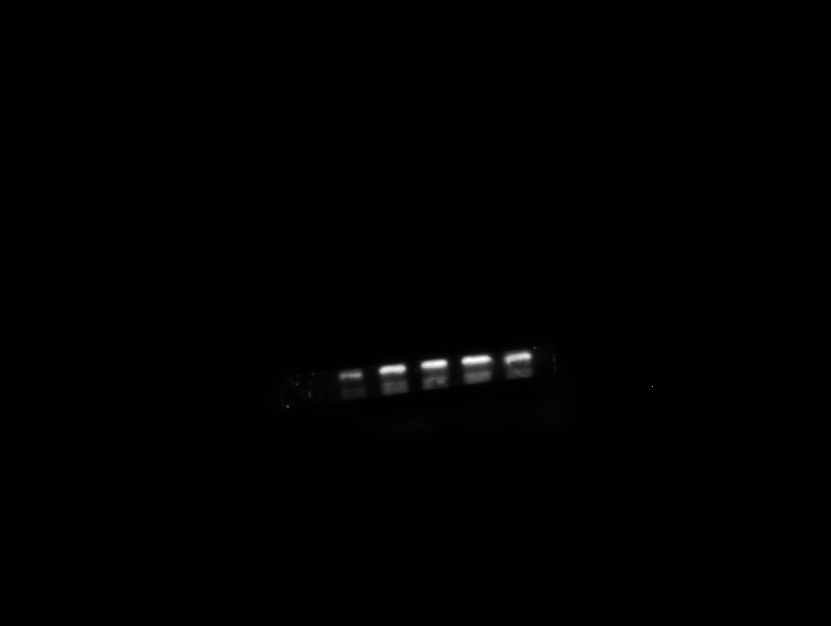

Supplement: Supplementary file 4 [file Data_Sheet_3.ZIP › Proteins for Anti-inflammation of compound 8/P-p65/2021-05-08_14-43-21_1_16bit.png]

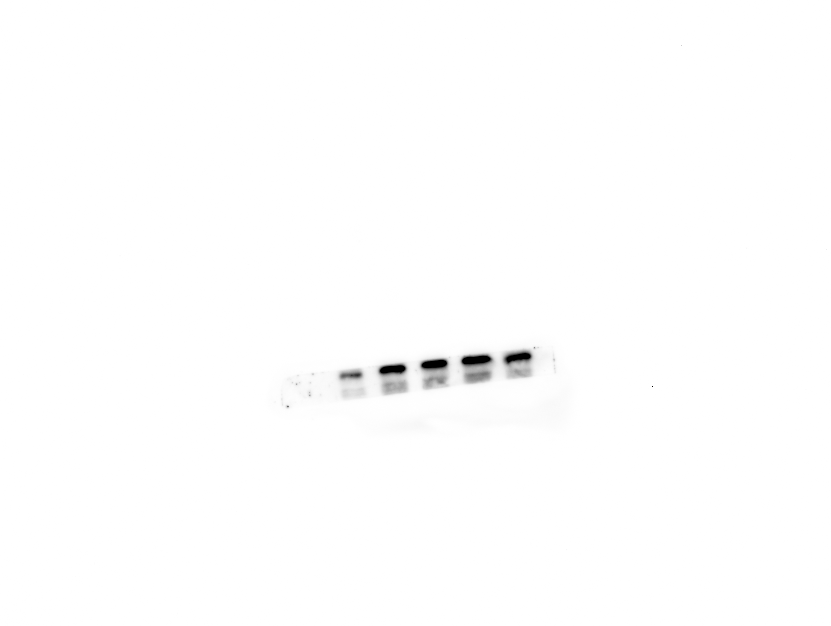

Supplement: Supplementary file 4 [file Data_Sheet_3.ZIP › Proteins for Anti-inflammation of compound 8/P-p65/2021-05-08_14-43-21_8bit.png]

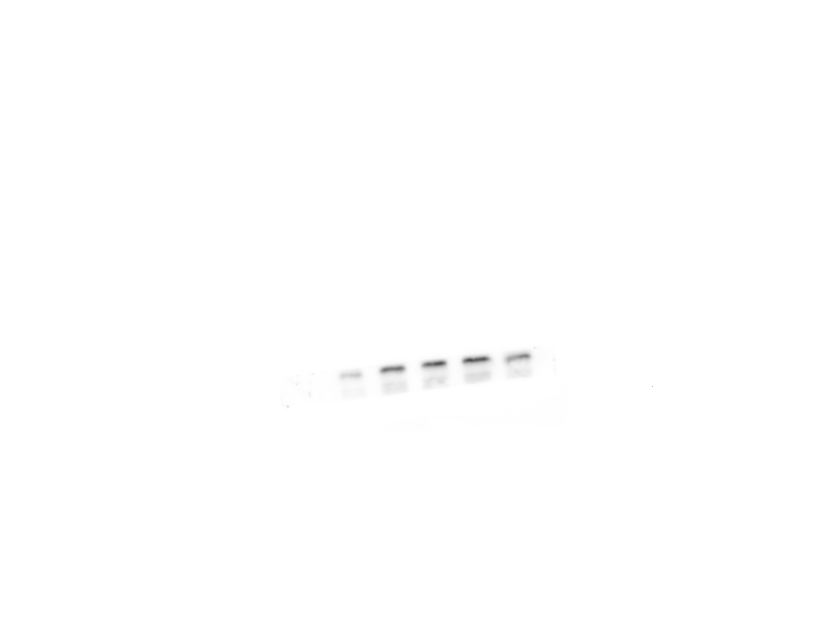

Supplement: Supplementary file 4 [file Data_Sheet_3.ZIP › Proteins for Anti-inflammation of compound 8/P-p65/contrast/contrast_0.png]

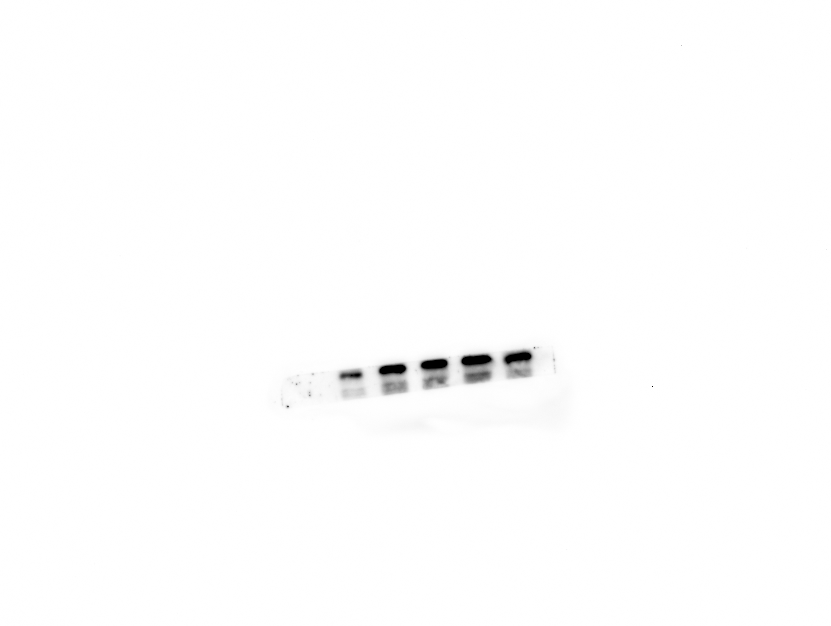

Supplement: Supplementary file 4 [file Data_Sheet_3.ZIP › Proteins for Anti-inflammation of compound 8/P-p65/contrast/contrast_2.png]

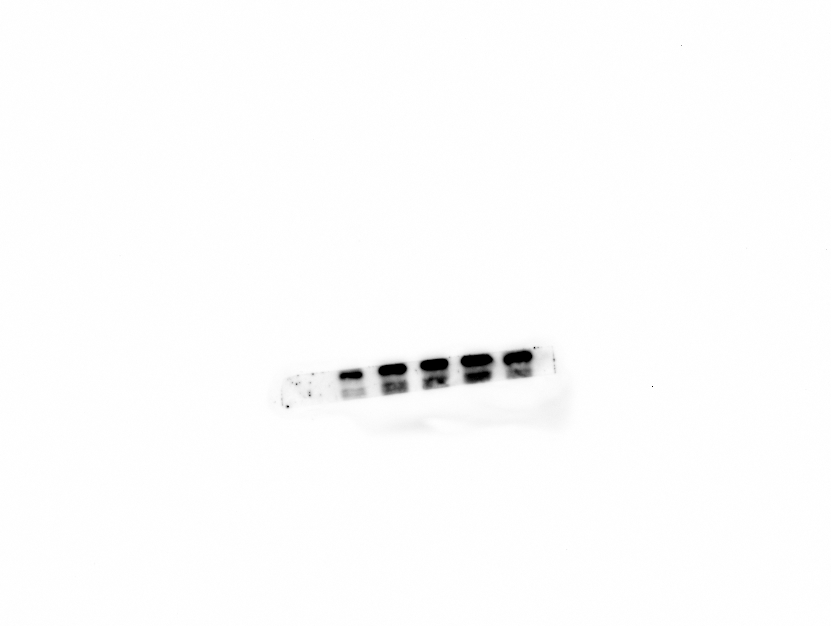

Supplement: Supplementary file 4 [file Data_Sheet_3.ZIP › Proteins for Anti-inflammation of compound 8/P-p65/contrast/contrast_3.png]

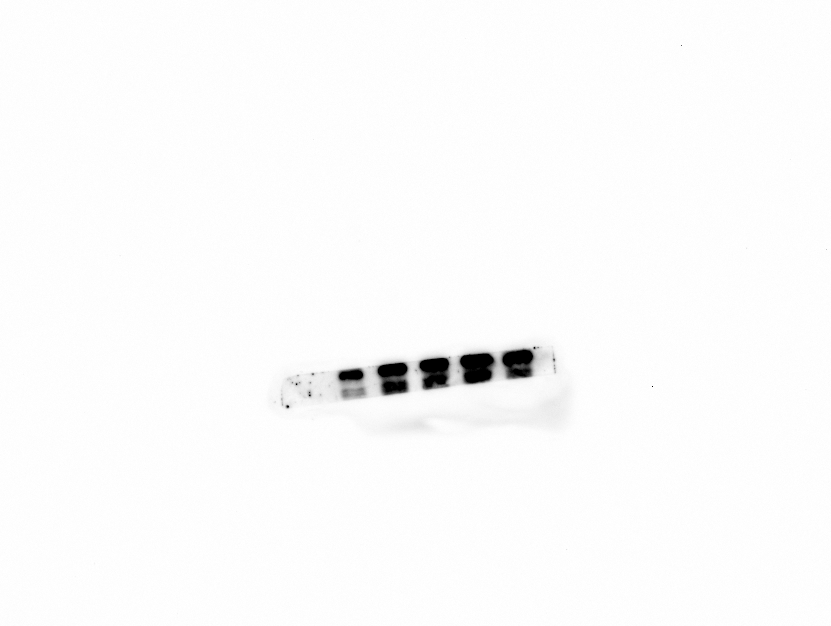

Supplement: Supplementary file 4 [file Data_Sheet_3.ZIP › Proteins for Anti-inflammation of compound 8/P-p65/contrast/contrast_4.png]

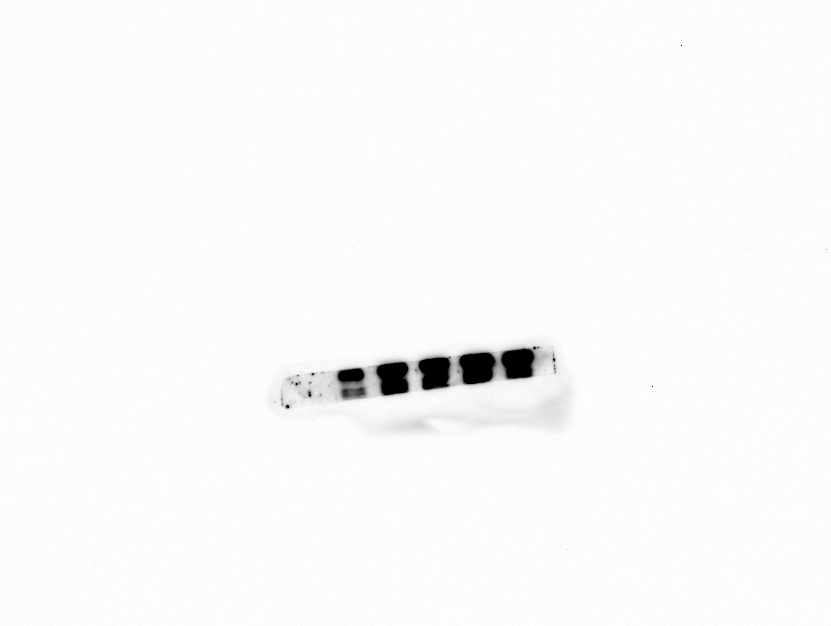

Supplement: Supplementary file 4 [file Data_Sheet_3.ZIP › Proteins for Anti-inflammation of compound 8/P-p65/contrast/contrast_5.png]

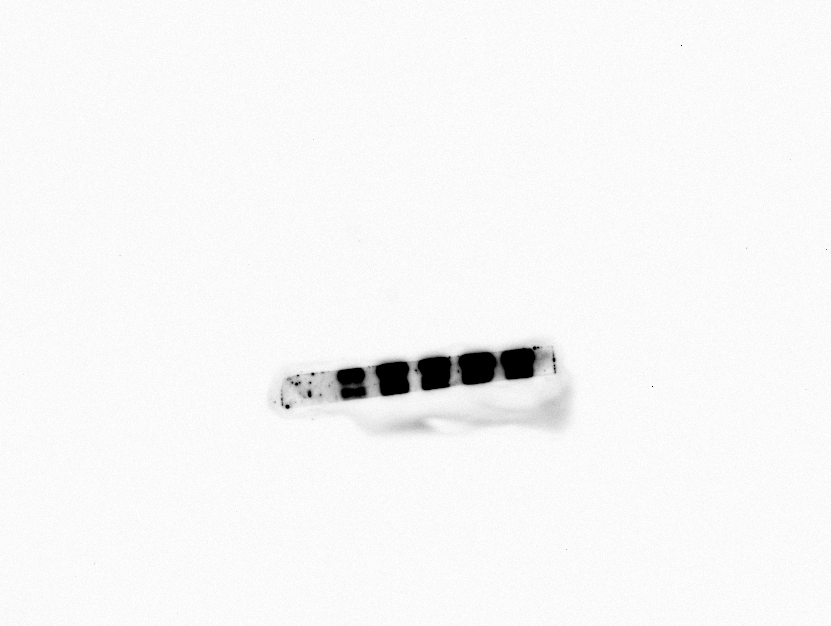

Supplement: Supplementary file 4 [file Data_Sheet_3.ZIP › Proteins for Anti-inflammation of compound 8/P-p65/contrast/contrast_6.png]

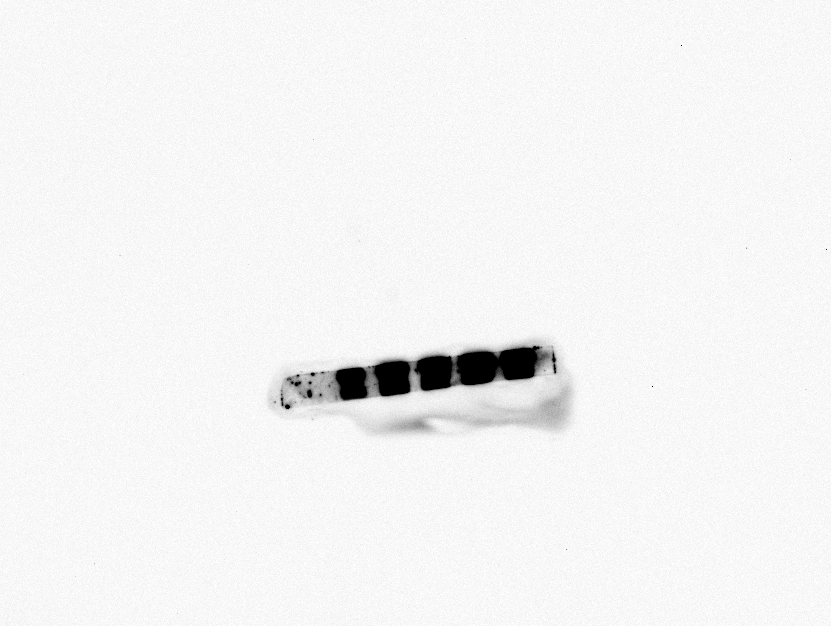

Supplement: Supplementary file 4 [file Data_Sheet_3.ZIP › Proteins for Anti-inflammation of compound 8/P-p65/contrast/contrast_7.png]

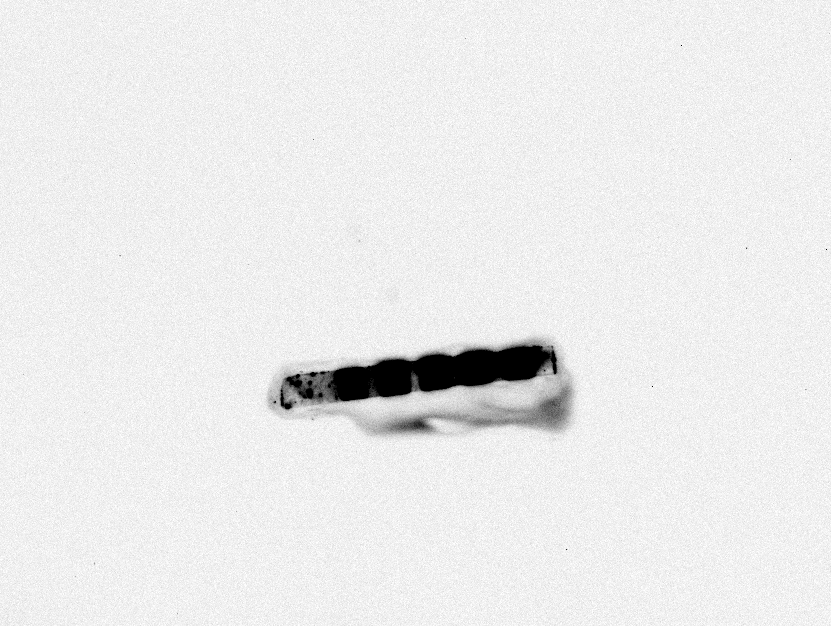

Supplement: Supplementary file 4 [file Data_Sheet_3.ZIP › Proteins for Anti-inflammation of compound 8/P-p65/contrast/contrast_8.png]

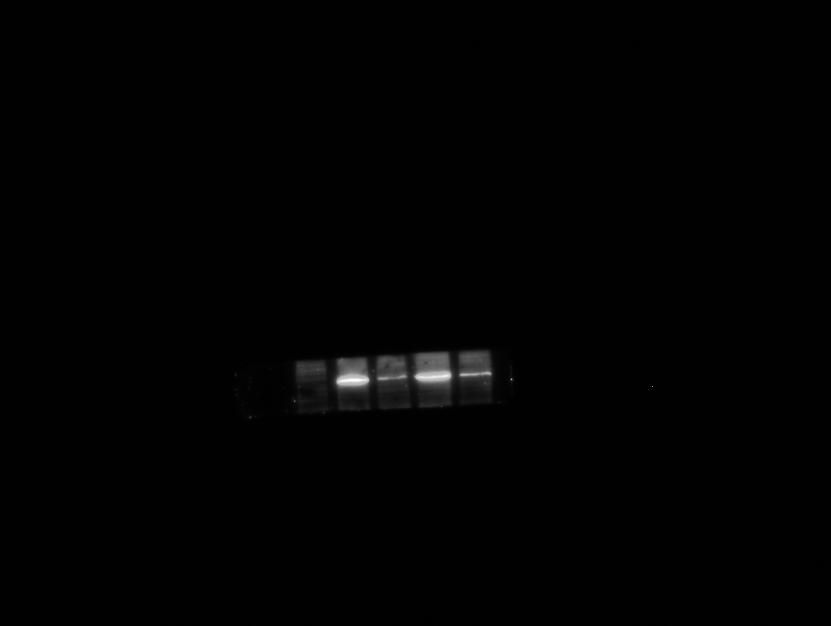

Supplement: Supplementary file 4 [file Data_Sheet_3.ZIP › Proteins for Anti-inflammation of compound 8/iNOS/2021-05-08_C7inos_1_16bit.png]

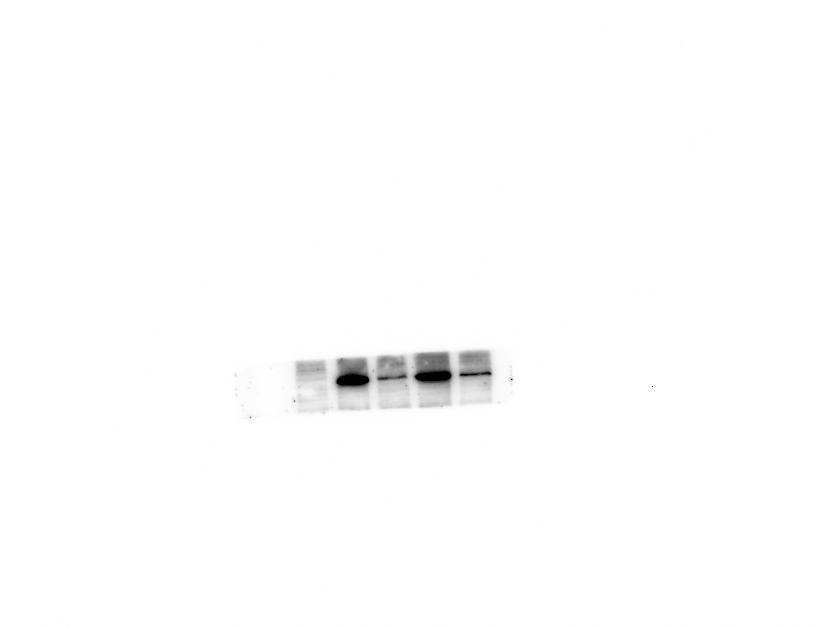

Supplement: Supplementary file 4 [file Data_Sheet_3.ZIP › Proteins for Anti-inflammation of compound 8/iNOS/2021-05-08_C7inos_8bit.png]

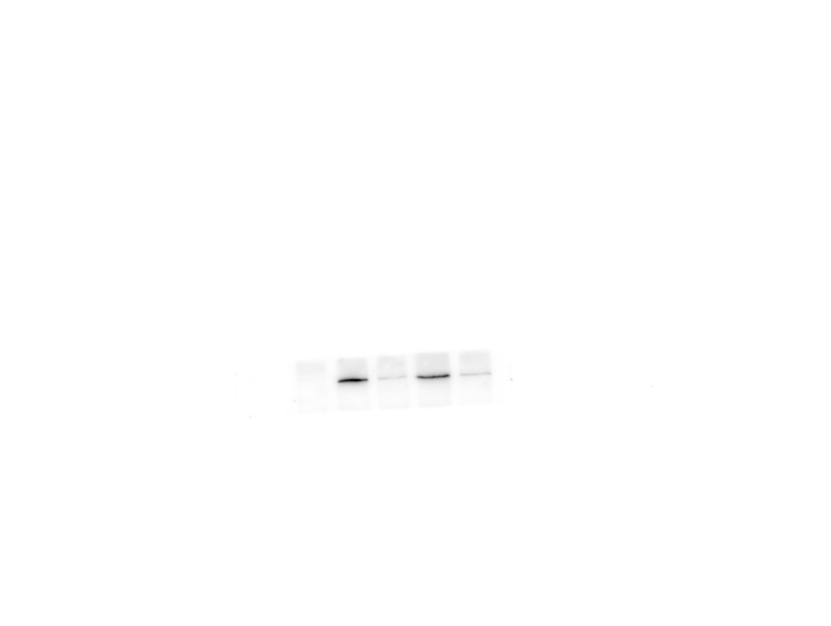

Supplement: Supplementary file 4 [file Data_Sheet_3.ZIP › Proteins for Anti-inflammation of compound 8/iNOS/contrast/contrast_0.png]

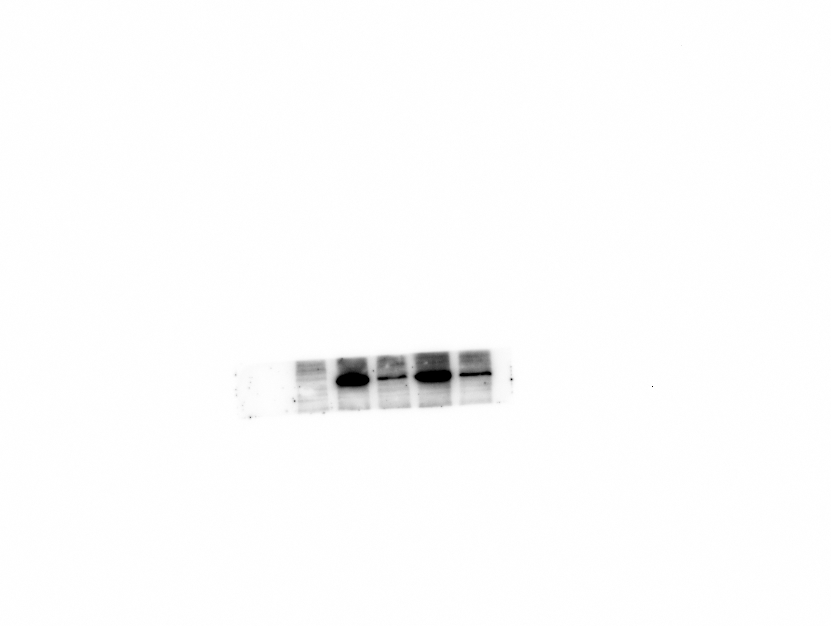

Supplement: Supplementary file 4 [file Data_Sheet_3.ZIP › Proteins for Anti-inflammation of compound 8/iNOS/contrast/contrast_2.png]

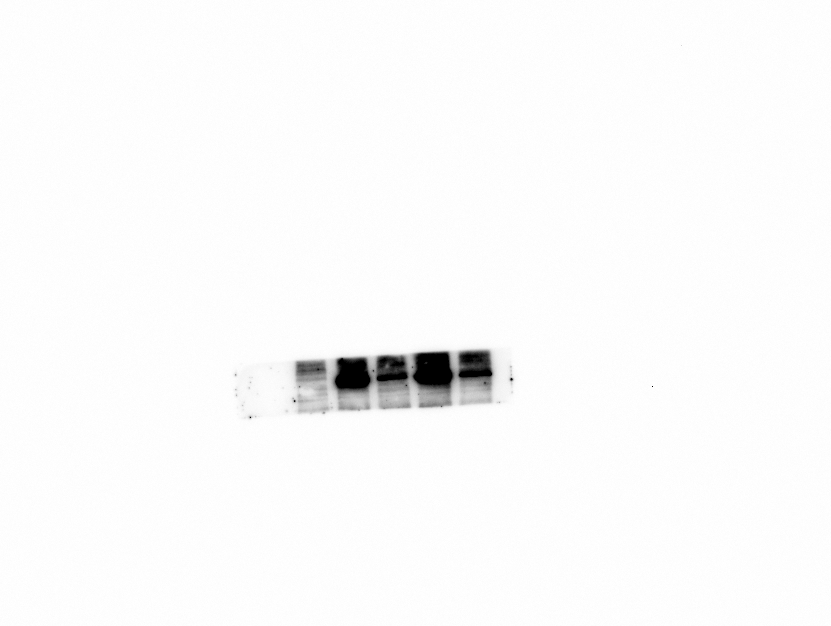

Supplement: Supplementary file 4 [file Data_Sheet_3.ZIP › Proteins for Anti-inflammation of compound 8/iNOS/contrast/contrast_3.png]

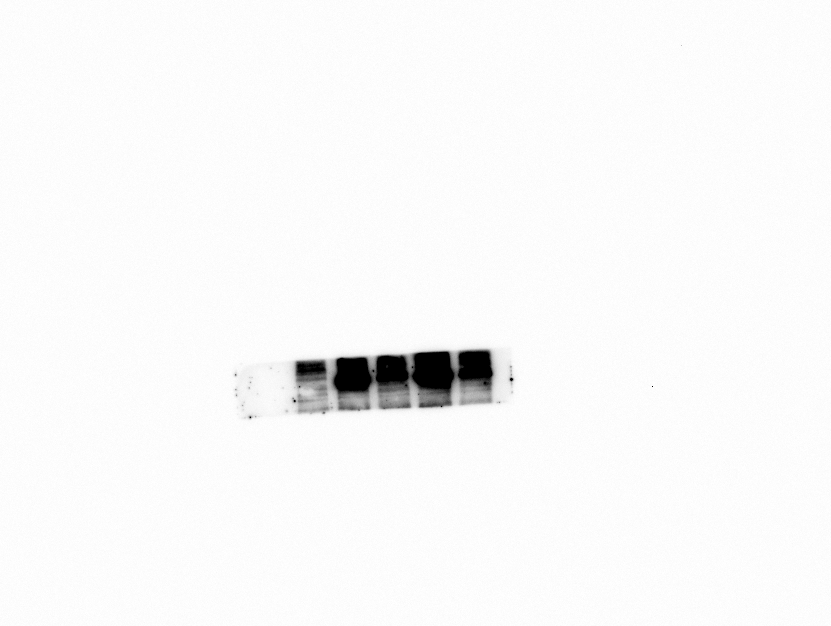

Supplement: Supplementary file 4 [file Data_Sheet_3.ZIP › Proteins for Anti-inflammation of compound 8/iNOS/contrast/contrast_4.png]

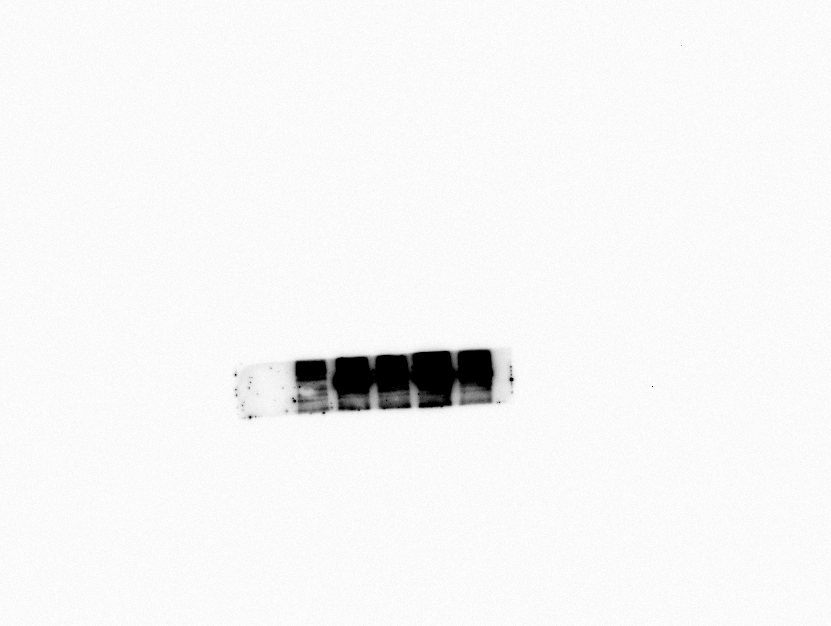

Supplement: Supplementary file 4 [file Data_Sheet_3.ZIP › Proteins for Anti-inflammation of compound 8/iNOS/contrast/contrast_5.png]

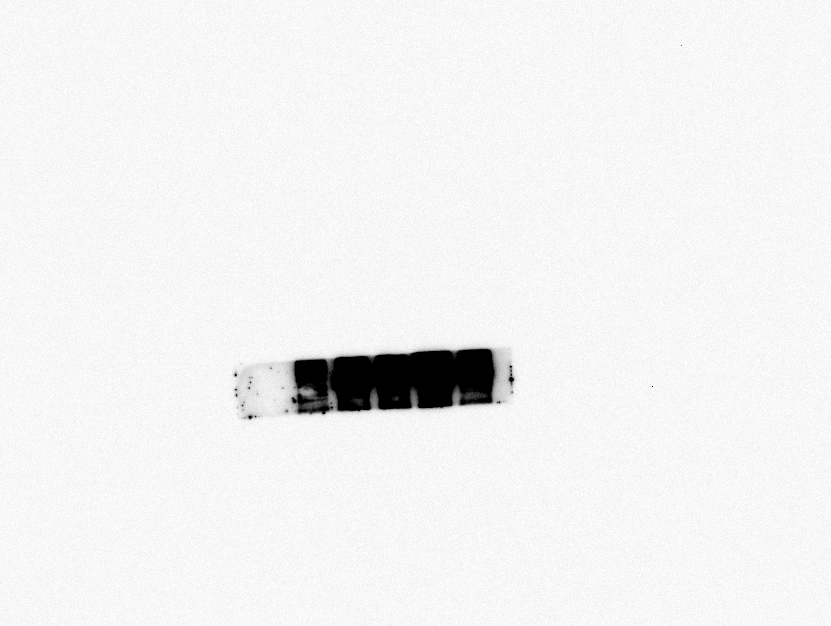

Supplement: Supplementary file 4 [file Data_Sheet_3.ZIP › Proteins for Anti-inflammation of compound 8/iNOS/contrast/contrast_6.png]

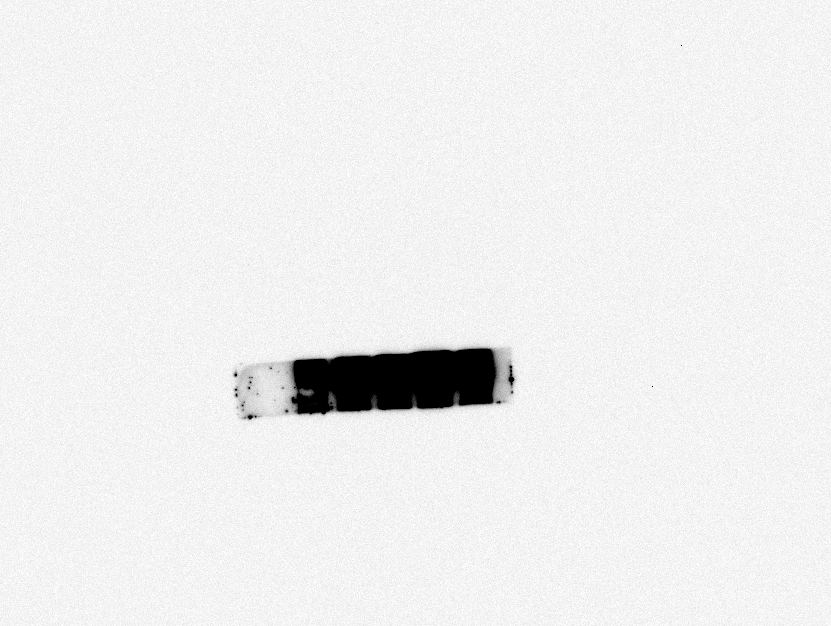

Supplement: Supplementary file 4 [file Data_Sheet_3.ZIP › Proteins for Anti-inflammation of compound 8/iNOS/contrast/contrast_7.png]

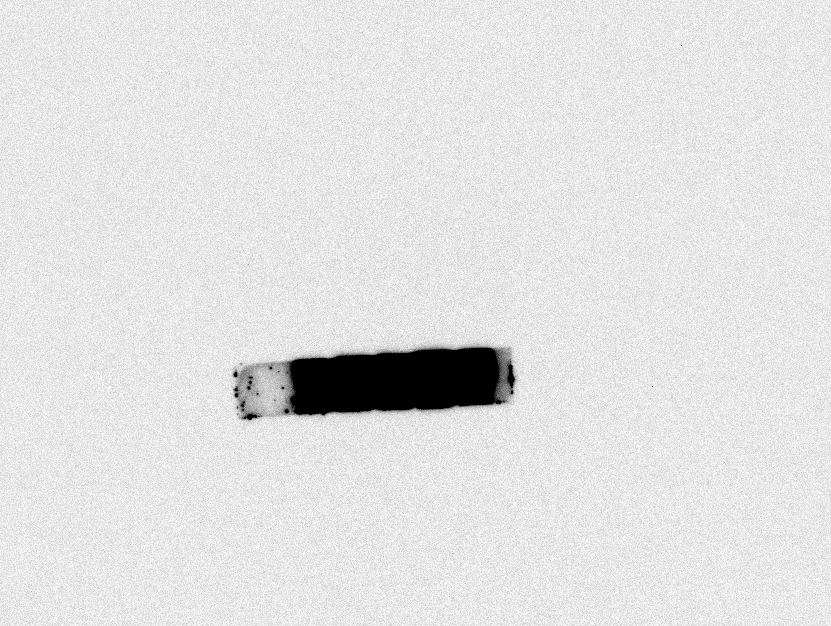

Supplement: Supplementary file 4 [file Data_Sheet_3.ZIP › Proteins for Anti-inflammation of compound 8/iNOS/contrast/contrast_8.png]

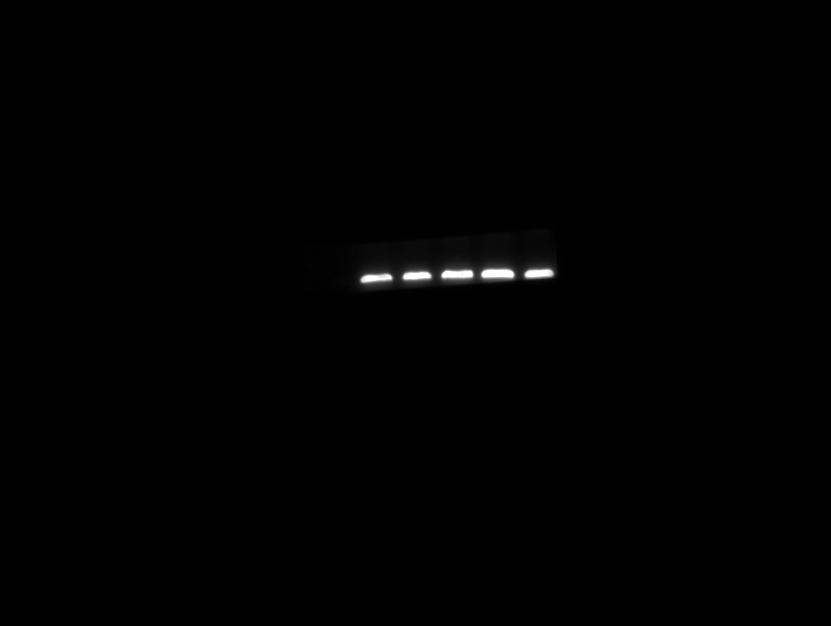

Supplement: Supplementary file 4 [file Data_Sheet_3.ZIP › Proteins for Anti-inflammation of compound 8/p65/2021-05-07_P65c7_1_16bit.png]

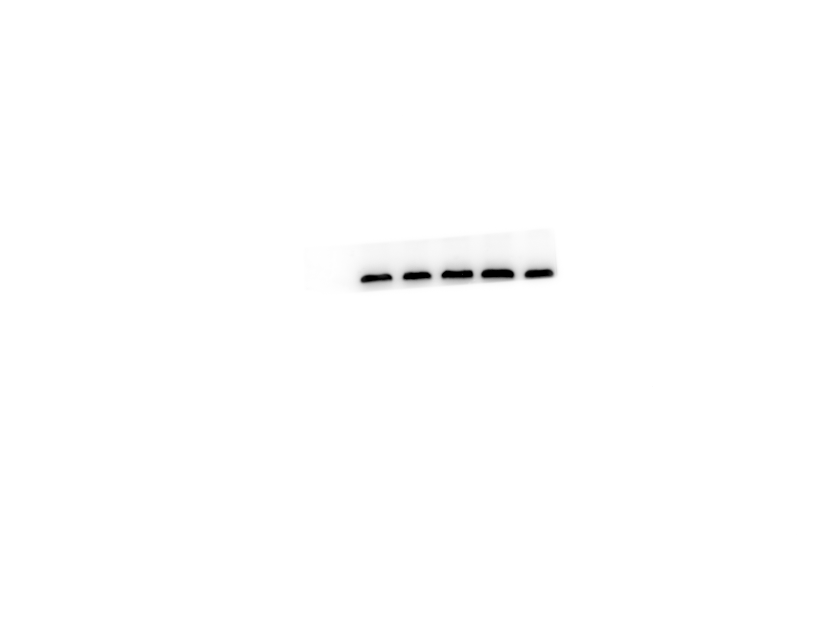

Supplement: Supplementary file 4 [file Data_Sheet_3.ZIP › Proteins for Anti-inflammation of compound 8/p65/2021-05-07_P65c7_8bit.png]

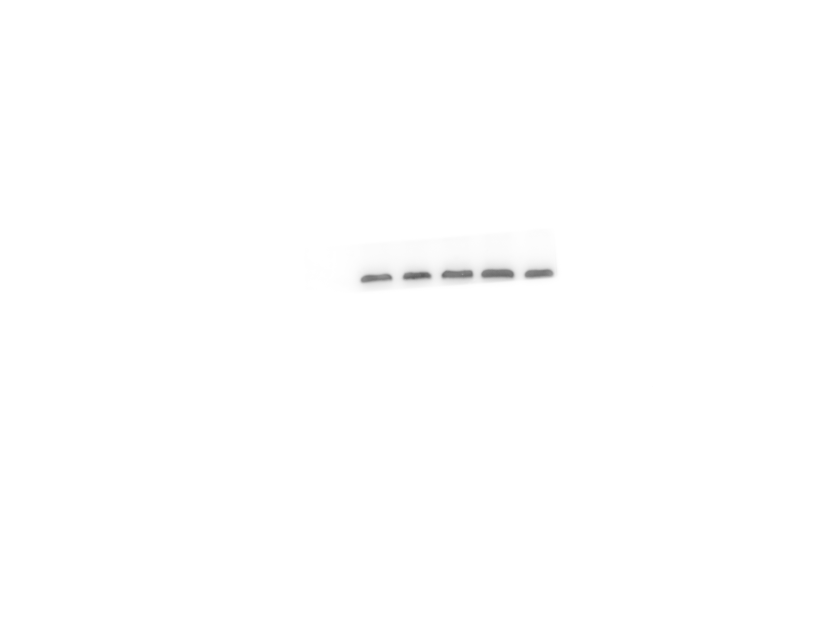

Supplement: Supplementary file 4 [file Data_Sheet_3.ZIP › Proteins for Anti-inflammation of compound 8/p65/contrast/contrast_0.png]

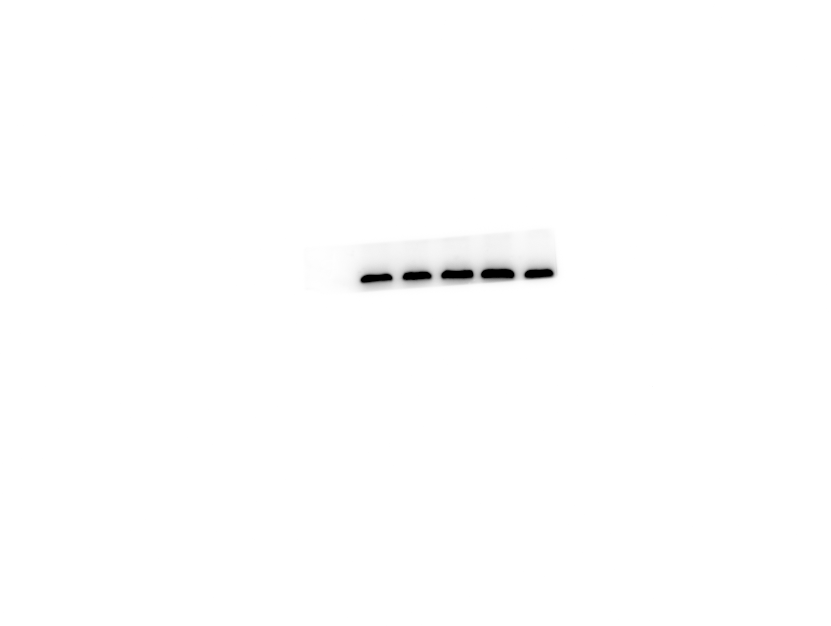

Supplement: Supplementary file 4 [file Data_Sheet_3.ZIP › Proteins for Anti-inflammation of compound 8/p65/contrast/contrast_2.png]

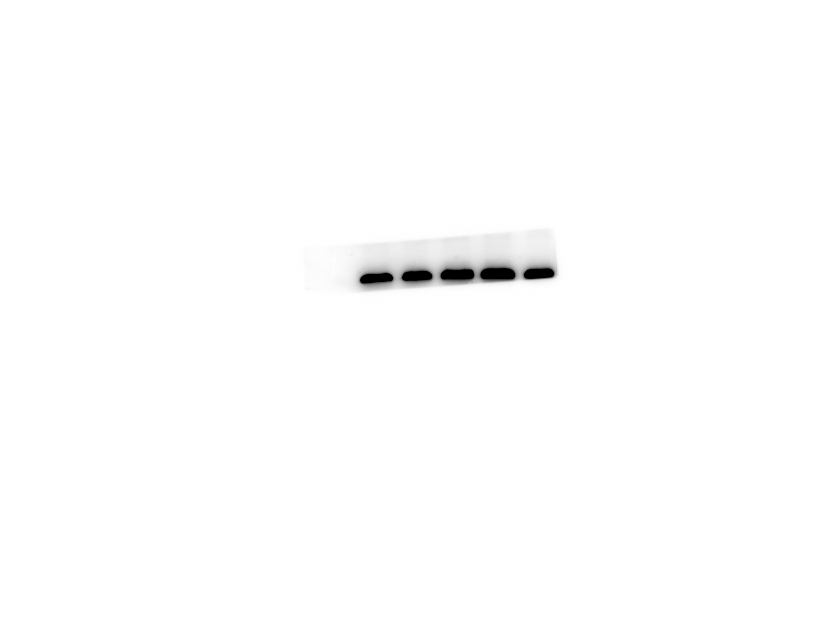

Supplement: Supplementary file 4 [file Data_Sheet_3.ZIP › Proteins for Anti-inflammation of compound 8/p65/contrast/contrast_3.png]

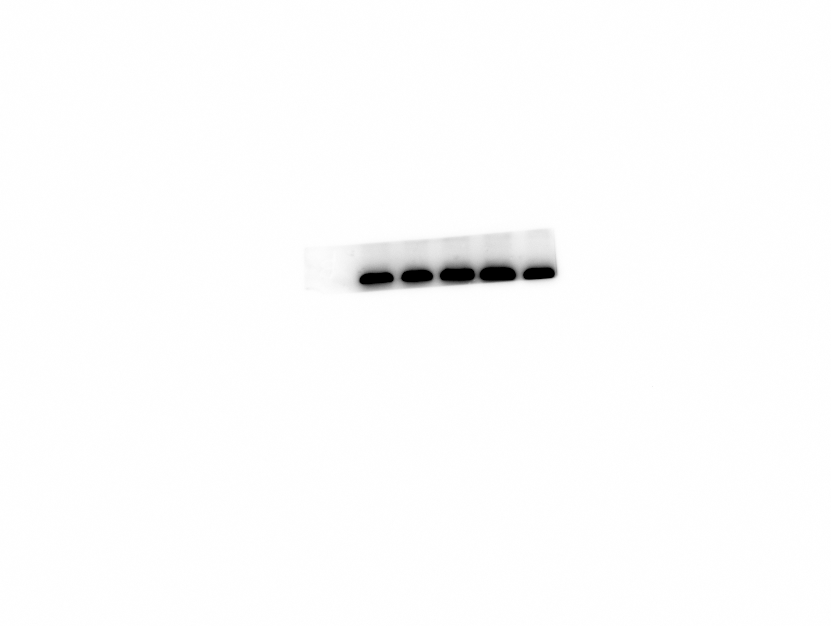

Supplement: Supplementary file 4 [file Data_Sheet_3.ZIP › Proteins for Anti-inflammation of compound 8/p65/contrast/contrast_4.png]

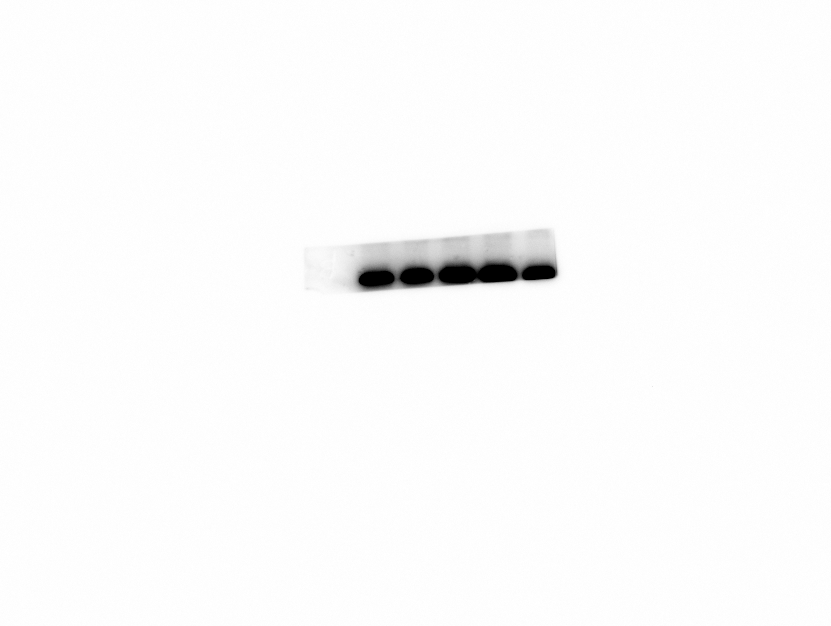

Supplement: Supplementary file 4 [file Data_Sheet_3.ZIP › Proteins for Anti-inflammation of compound 8/p65/contrast/contrast_5.png]

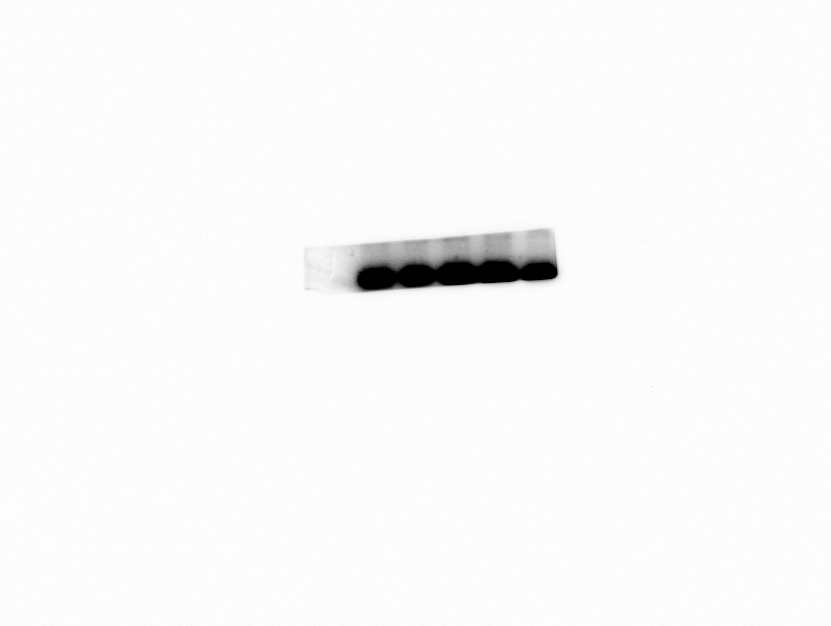

Supplement: Supplementary file 4 [file Data_Sheet_3.ZIP › Proteins for Anti-inflammation of compound 8/p65/contrast/contrast_6.png]

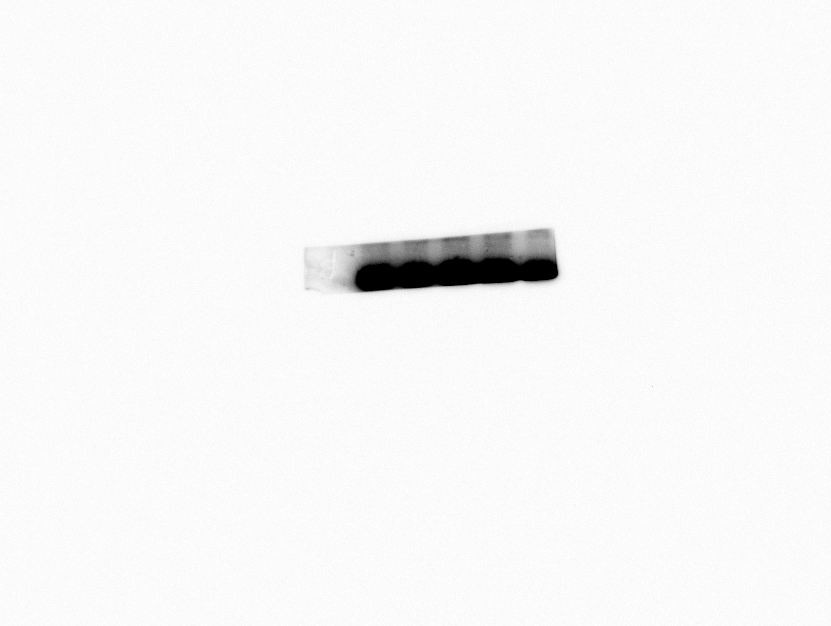

Supplement: Supplementary file 4 [file Data_Sheet_3.ZIP › Proteins for Anti-inflammation of compound 8/p65/contrast/contrast_7.png]

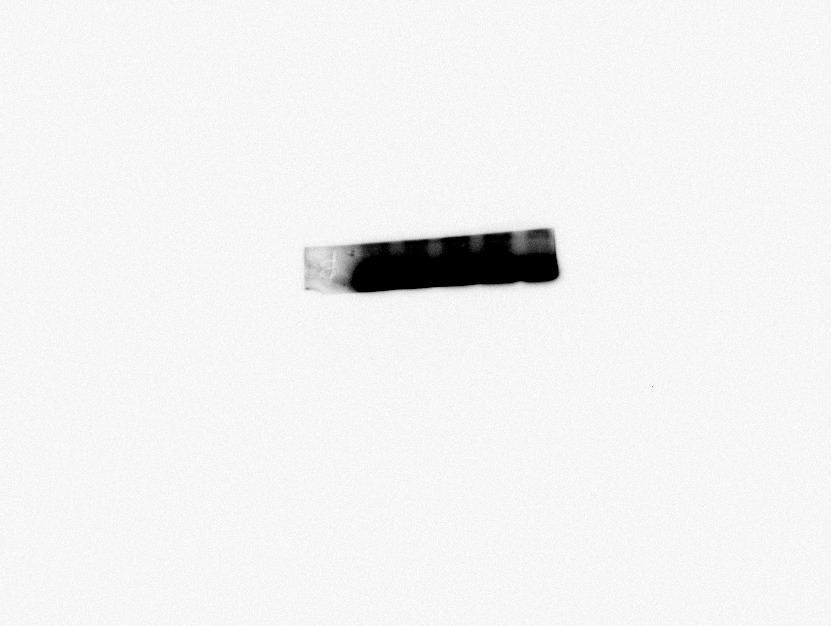

Supplement: Supplementary file 4 [file Data_Sheet_3.ZIP › Proteins for Anti-inflammation of compound 8/p65/contrast/contrast_8.png]

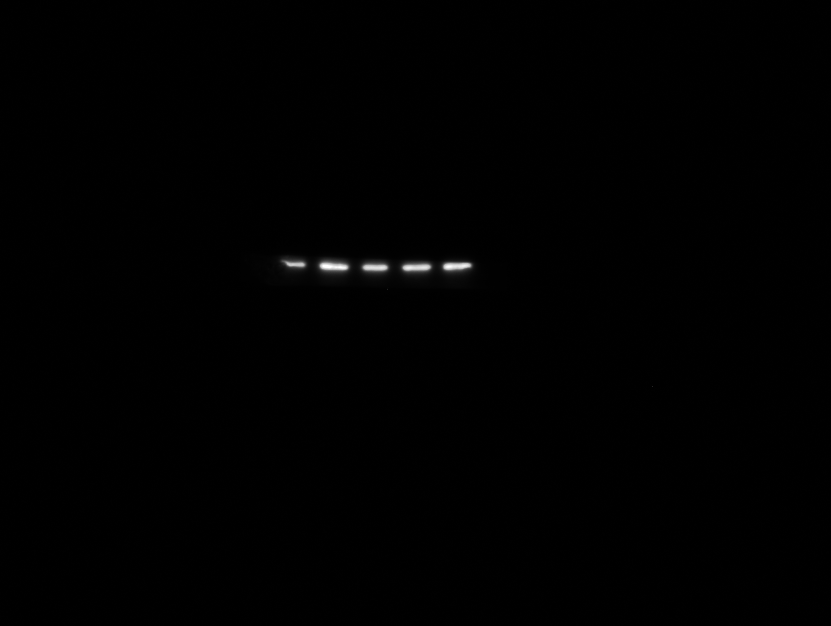

Supplement: Supplementary file 4 [file Data_Sheet_3.ZIP › Proteins for Anti-inflammation of compound 8/a┬-actin/2021-05-07_20-23-21_1_16bit.png]

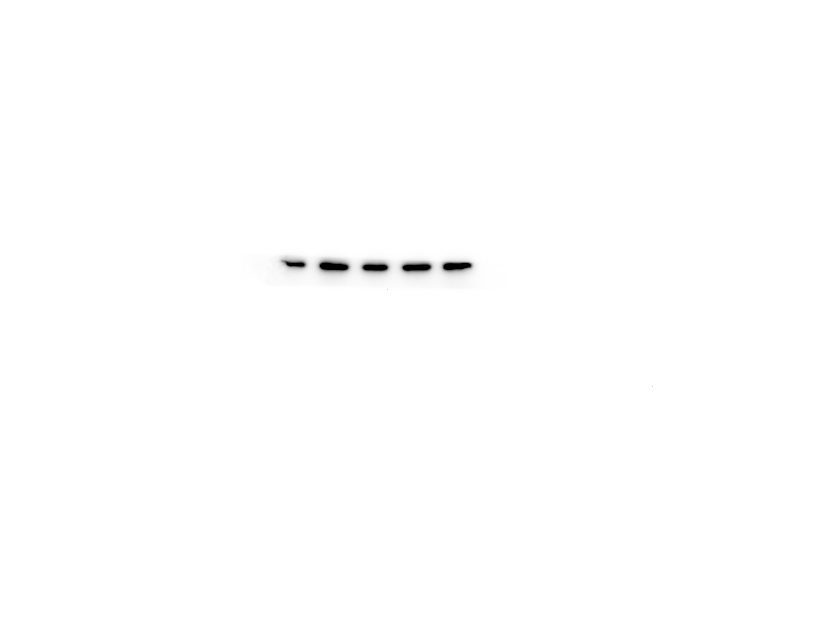

Supplement: Supplementary file 4 [file Data_Sheet_3.ZIP › Proteins for Anti-inflammation of compound 8/a┬-actin/2021-05-07_20-23-21_8bit.png]

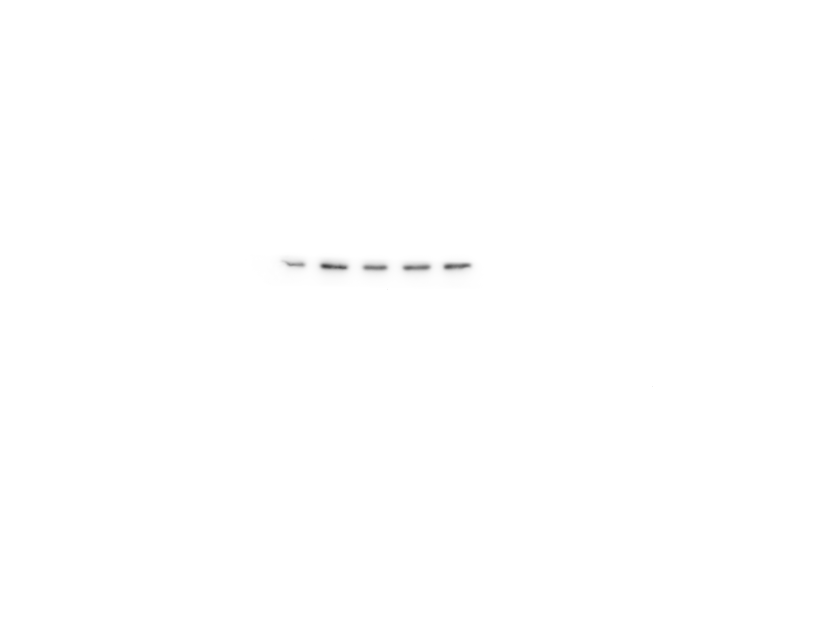

Supplement: Supplementary file 4 [file Data_Sheet_3.ZIP › Proteins for Anti-inflammation of compound 8/a┬-actin/contrast/contrast_0.png]

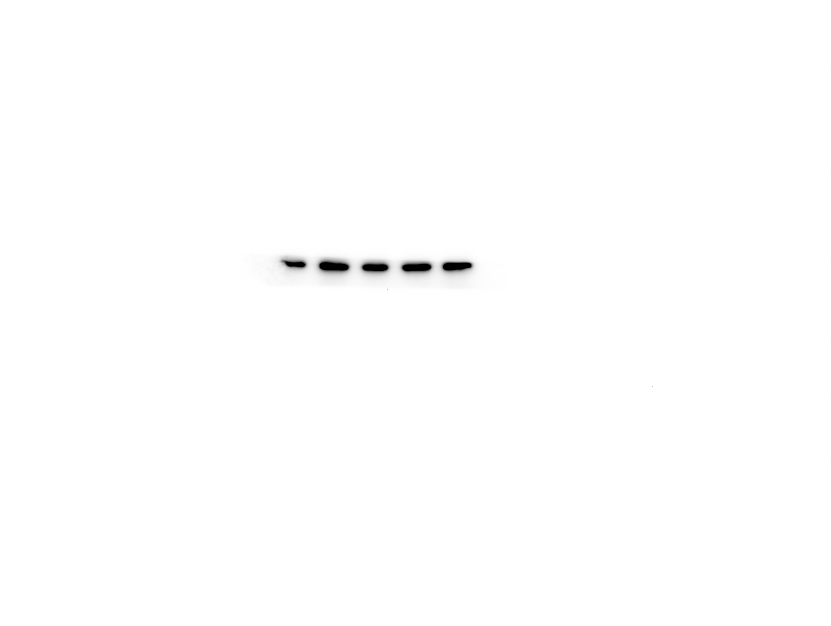

Supplement: Supplementary file 4 [file Data_Sheet_3.ZIP › Proteins for Anti-inflammation of compound 8/a┬-actin/contrast/contrast_2.png]

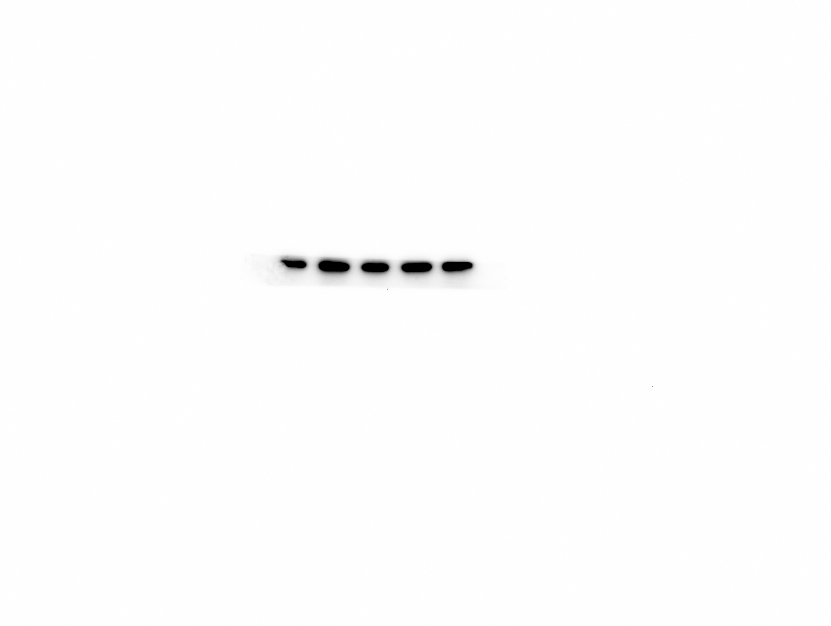

Supplement: Supplementary file 4 [file Data_Sheet_3.ZIP › Proteins for Anti-inflammation of compound 8/a┬-actin/contrast/contrast_3.png]

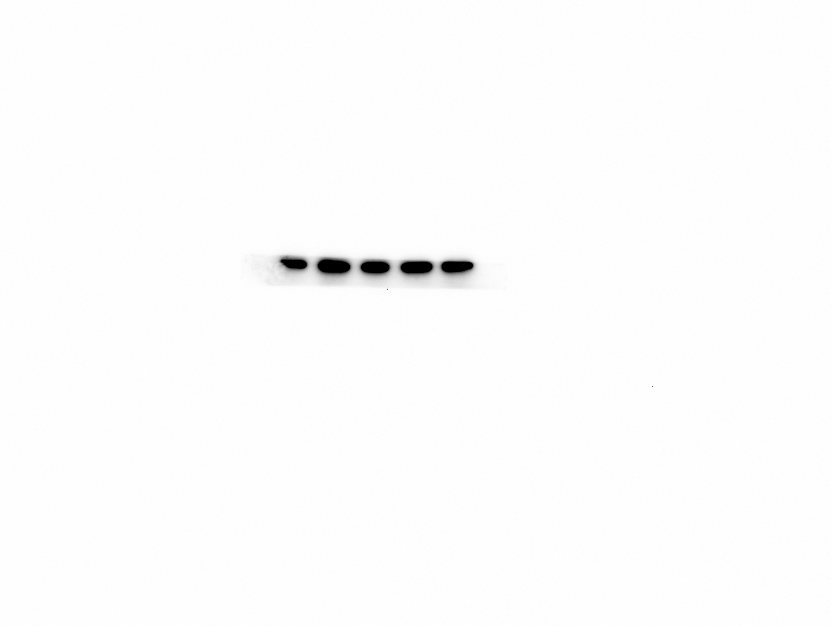

Supplement: Supplementary file 4 [file Data_Sheet_3.ZIP › Proteins for Anti-inflammation of compound 8/a┬-actin/contrast/contrast_4.png]

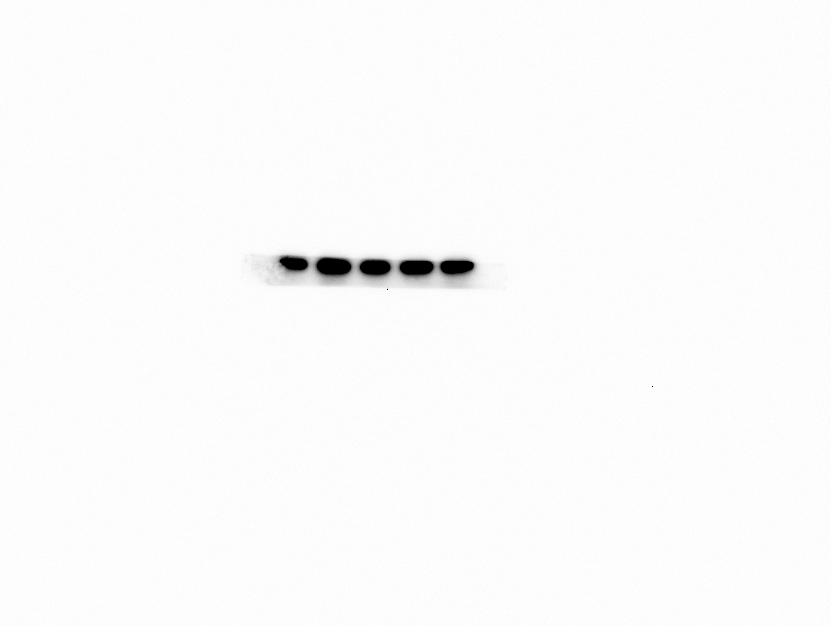

Supplement: Supplementary file 4 [file Data_Sheet_3.ZIP › Proteins for Anti-inflammation of compound 8/a┬-actin/contrast/contrast_5.png]

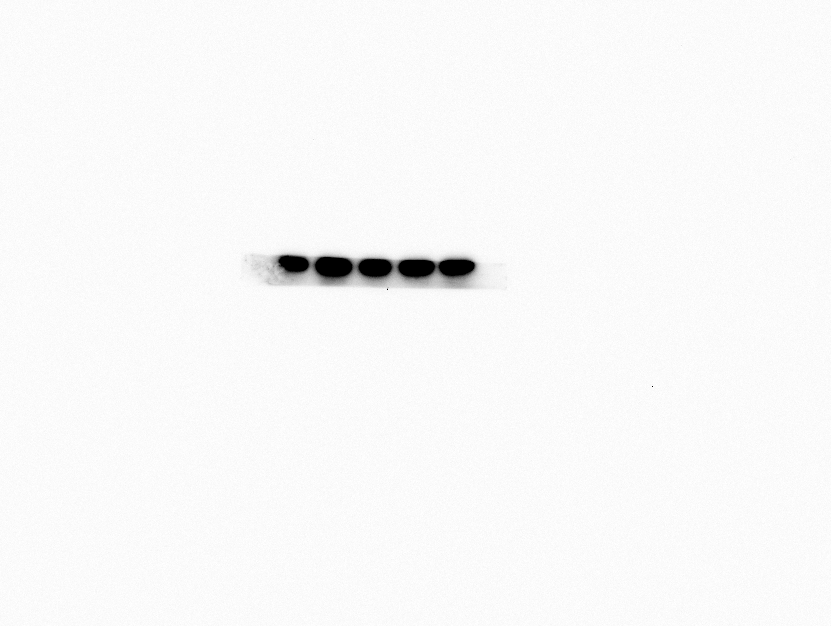

Supplement: Supplementary file 4 [file Data_Sheet_3.ZIP › Proteins for Anti-inflammation of compound 8/a┬-actin/contrast/contrast_6.png]

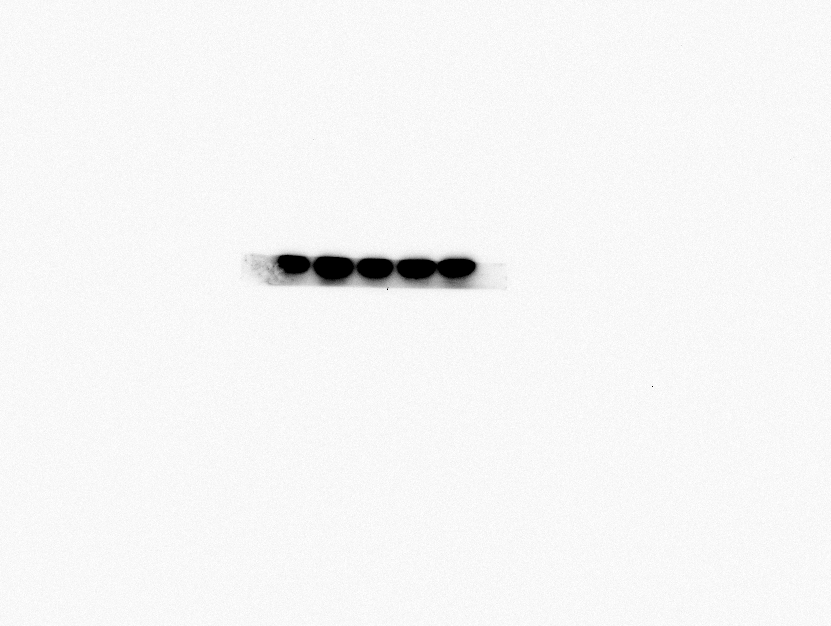

Supplement: Supplementary file 4 [file Data_Sheet_3.ZIP › Proteins for Anti-inflammation of compound 8/a┬-actin/contrast/contrast_7.png]

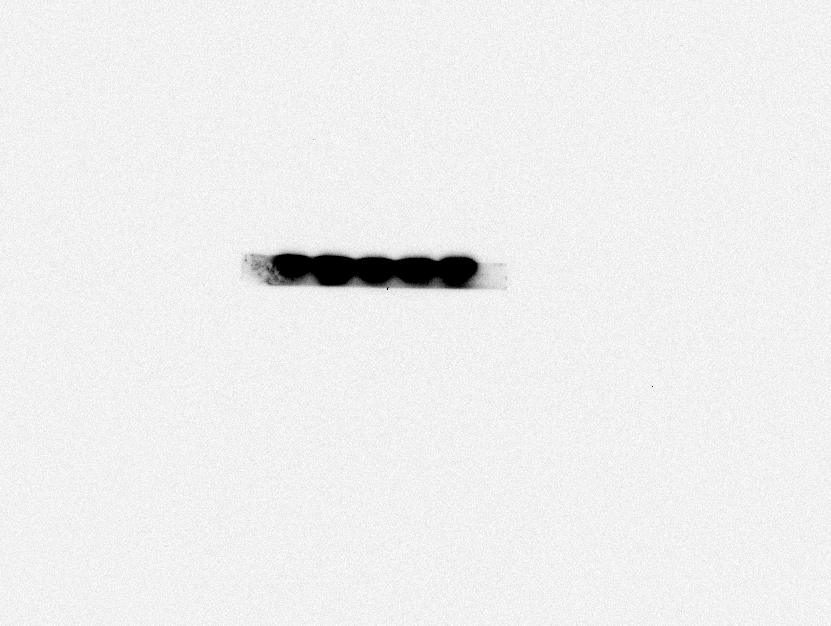

Supplement: Supplementary file 4 [file Data_Sheet_3.ZIP › Proteins for Anti-inflammation of compound 8/a┬-actin/contrast/contrast_8.png]

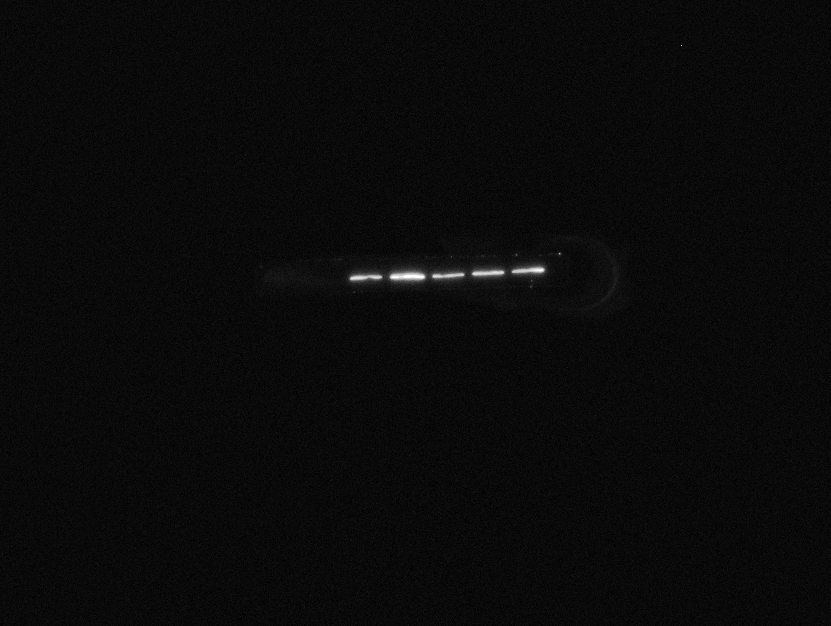

Supplement: Supplementary file 5 [file Data_Sheet_4.ZIP › Proteins for immune of compound 5/COX-2/2021-01-13Cox2_1_16bit.png]

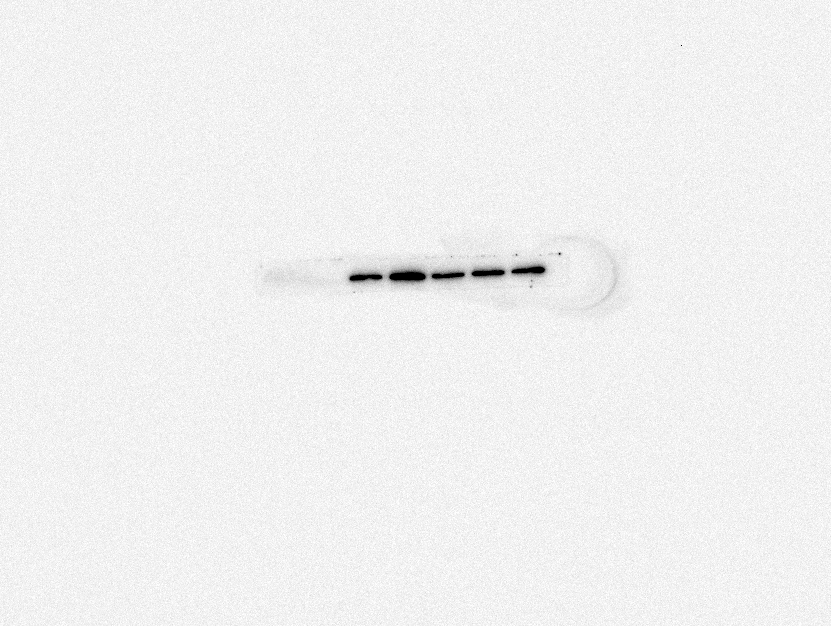

Supplement: Supplementary file 5 [file Data_Sheet_4.ZIP › Proteins for immune of compound 5/COX-2/2021-01-13Cox2_8bit.png]

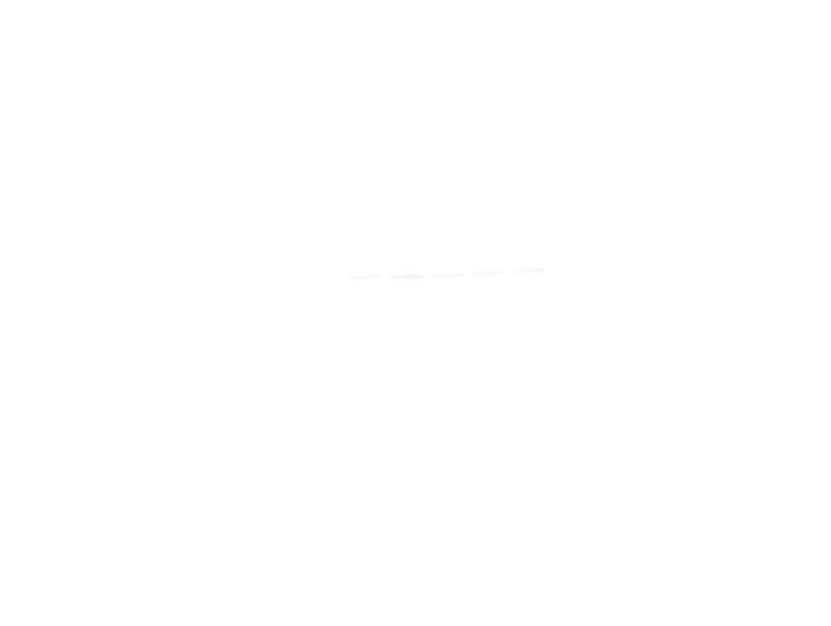

Supplement: Supplementary file 5 [file Data_Sheet_4.ZIP › Proteins for immune of compound 5/COX-2/contrast/contrast_0.png]

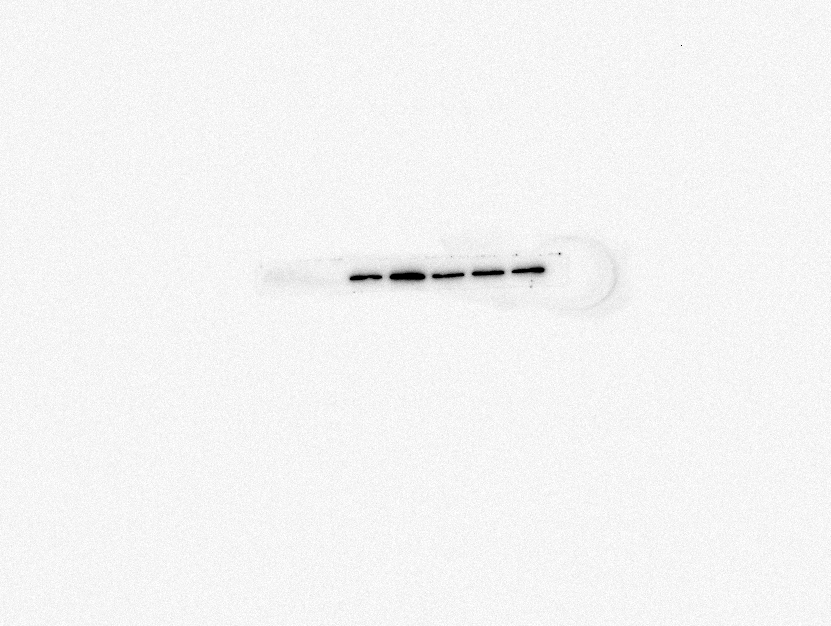

Supplement: Supplementary file 5 [file Data_Sheet_4.ZIP › Proteins for immune of compound 5/COX-2/contrast/contrast_1.png]

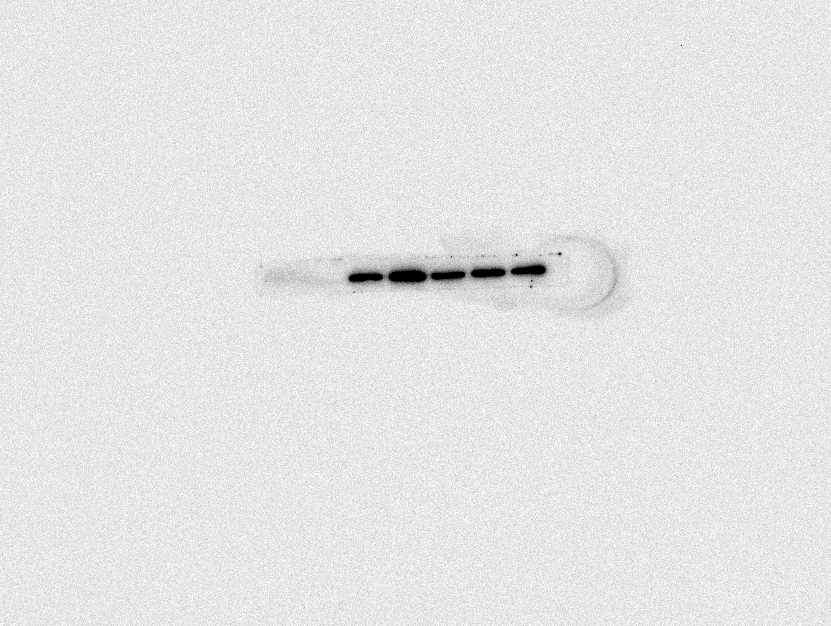

Supplement: Supplementary file 5 [file Data_Sheet_4.ZIP › Proteins for immune of compound 5/COX-2/contrast/contrast_3.png]

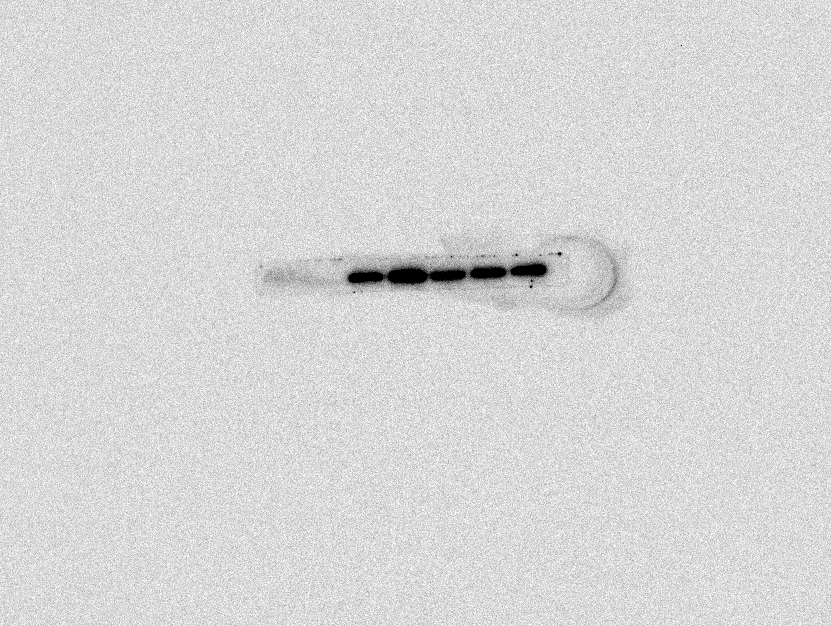

Supplement: Supplementary file 5 [file Data_Sheet_4.ZIP › Proteins for immune of compound 5/COX-2/contrast/contrast_4.png]

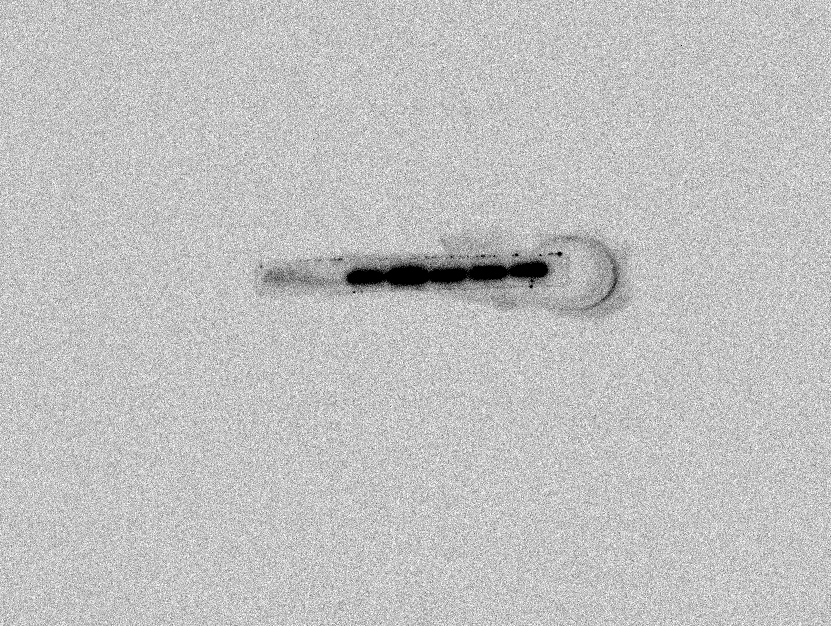

Supplement: Supplementary file 5 [file Data_Sheet_4.ZIP › Proteins for immune of compound 5/COX-2/contrast/contrast_5.png]

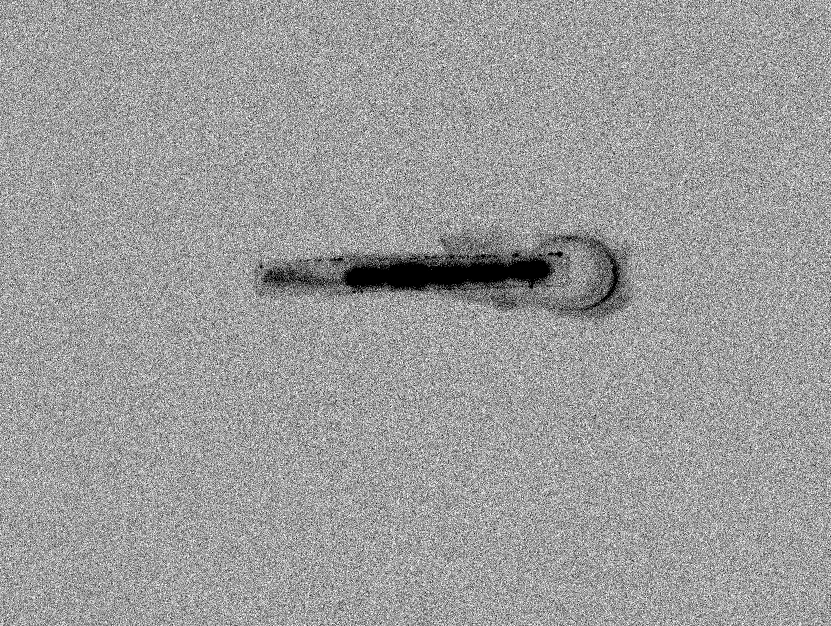

Supplement: Supplementary file 5 [file Data_Sheet_4.ZIP › Proteins for immune of compound 5/COX-2/contrast/contrast_6.png]

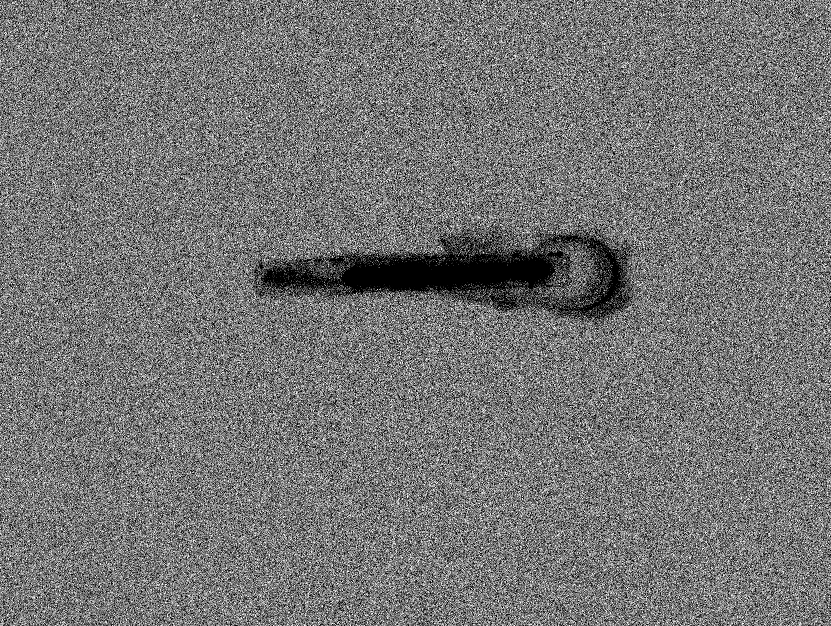

Supplement: Supplementary file 5 [file Data_Sheet_4.ZIP › Proteins for immune of compound 5/COX-2/contrast/contrast_7.png]

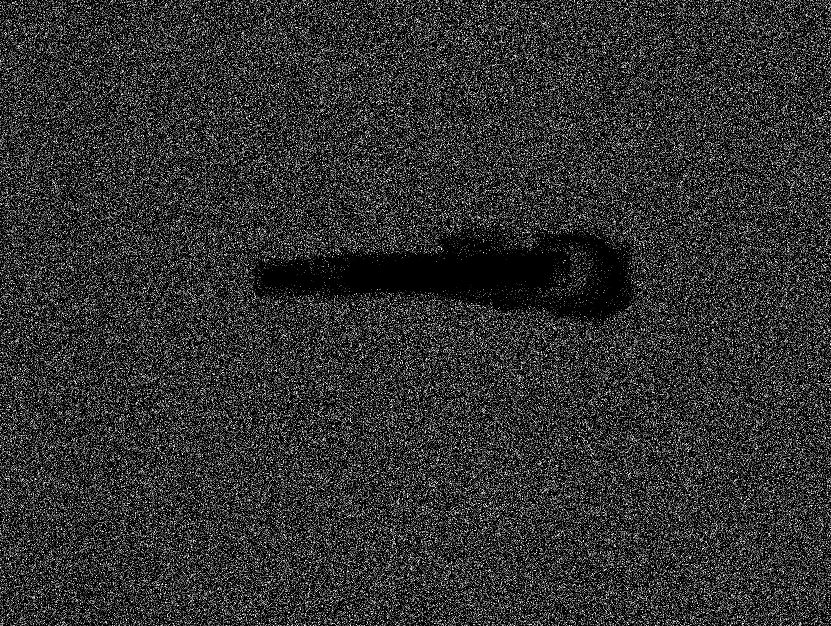

Supplement: Supplementary file 5 [file Data_Sheet_4.ZIP › Proteins for immune of compound 5/COX-2/contrast/contrast_8.png]

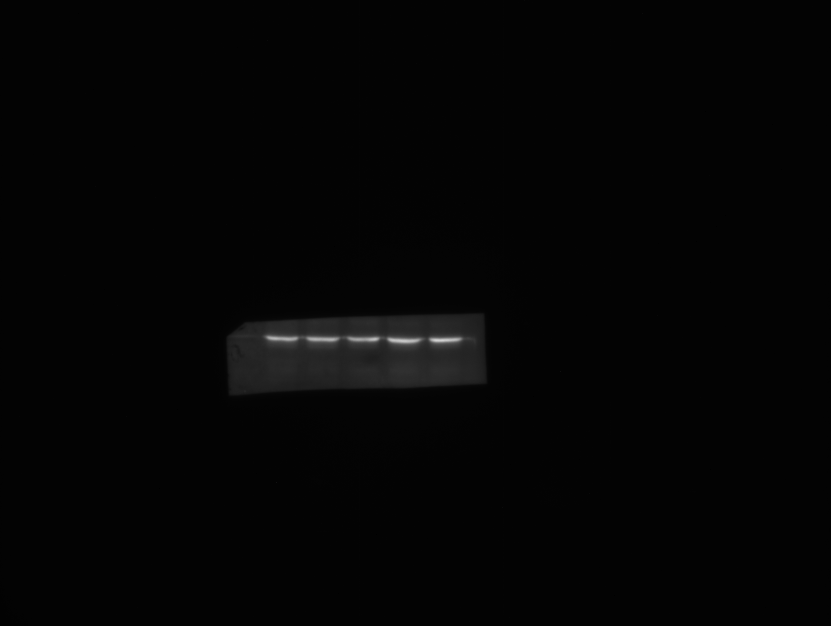

Supplement: Supplementary file 5 [file Data_Sheet_4.ZIP › Proteins for immune of compound 5/GAPDH/Z-actin_1_16bit.png]

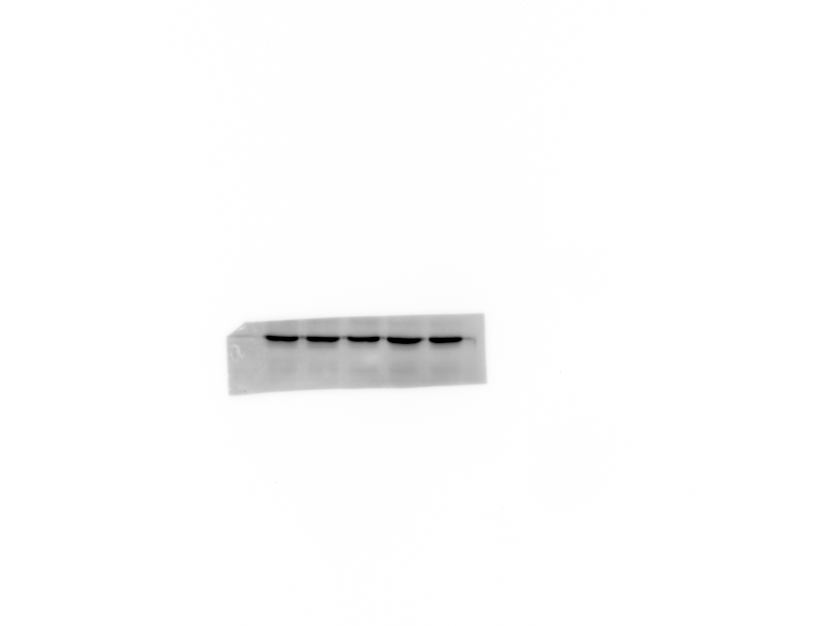

Supplement: Supplementary file 5 [file Data_Sheet_4.ZIP › Proteins for immune of compound 5/GAPDH/Z-actin_8bit.png]

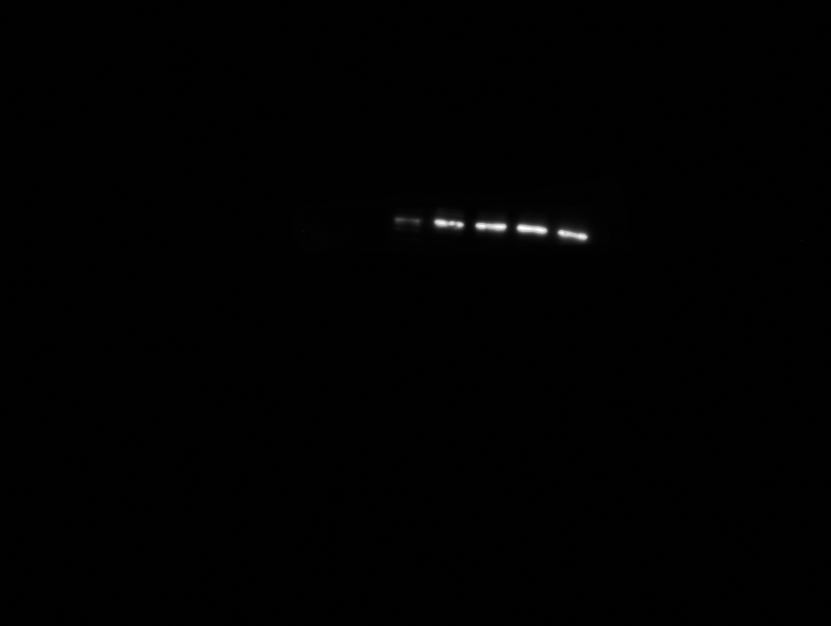

Supplement: Supplementary file 5 [file Data_Sheet_4.ZIP › Proteins for immune of compound 5/P-Ia╩Ba┴/2021-01-15_Pikba_1_16bit.png]

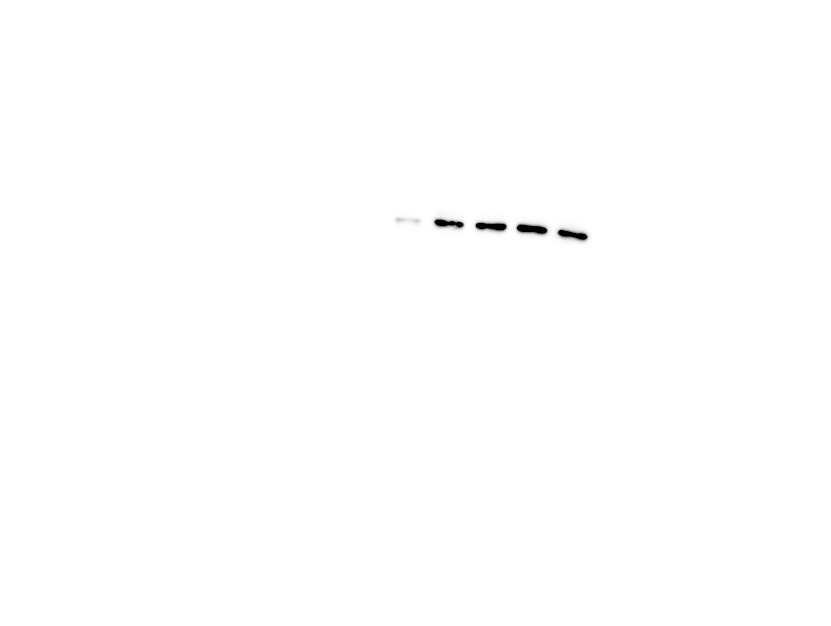

Supplement: Supplementary file 5 [file Data_Sheet_4.ZIP › Proteins for immune of compound 5/P-Ia╩Ba┴/2021-01-15_Pikba_8bit.png]

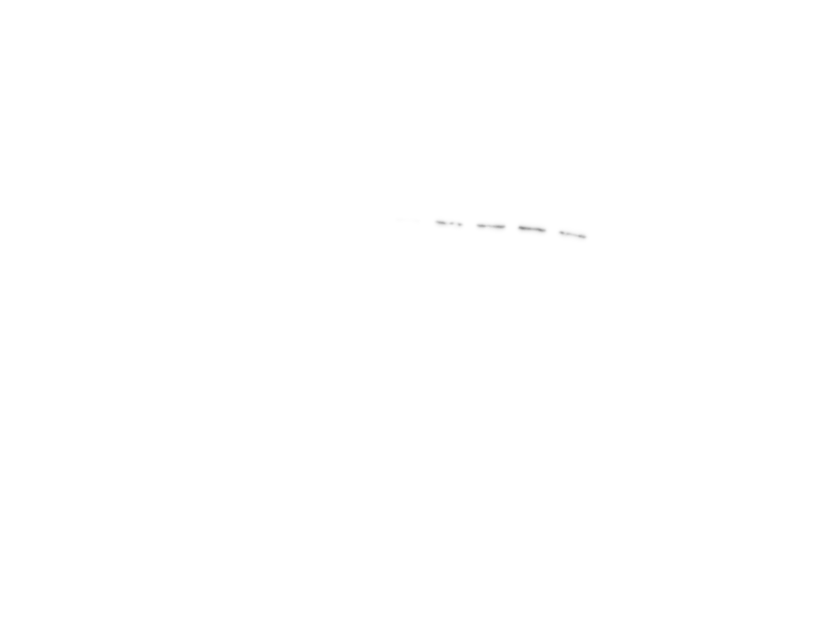

Supplement: Supplementary file 5 [file Data_Sheet_4.ZIP › Proteins for immune of compound 5/P-Ia╩Ba┴/contrast/contrast_0.png]

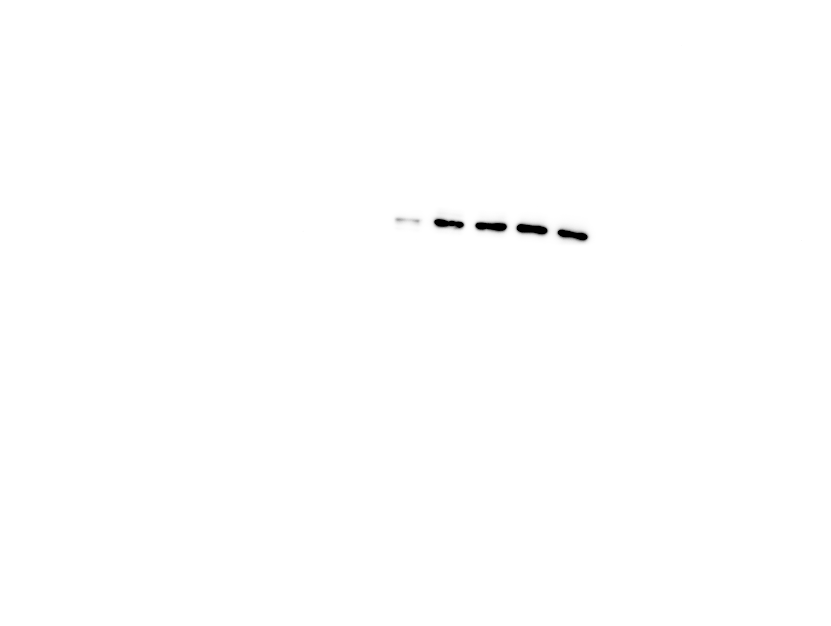

Supplement: Supplementary file 5 [file Data_Sheet_4.ZIP › Proteins for immune of compound 5/P-Ia╩Ba┴/contrast/contrast_2.png]

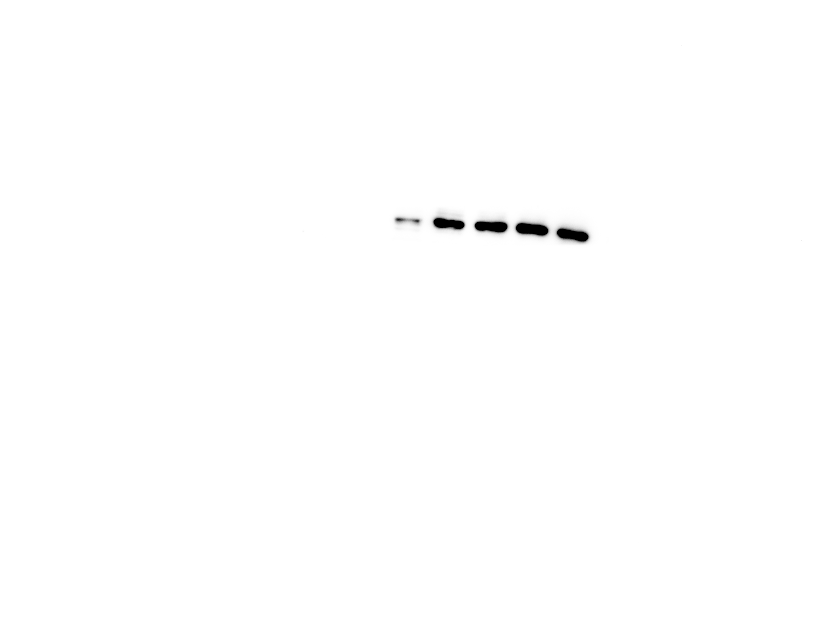

Supplement: Supplementary file 5 [file Data_Sheet_4.ZIP › Proteins for immune of compound 5/P-Ia╩Ba┴/contrast/contrast_3.png]

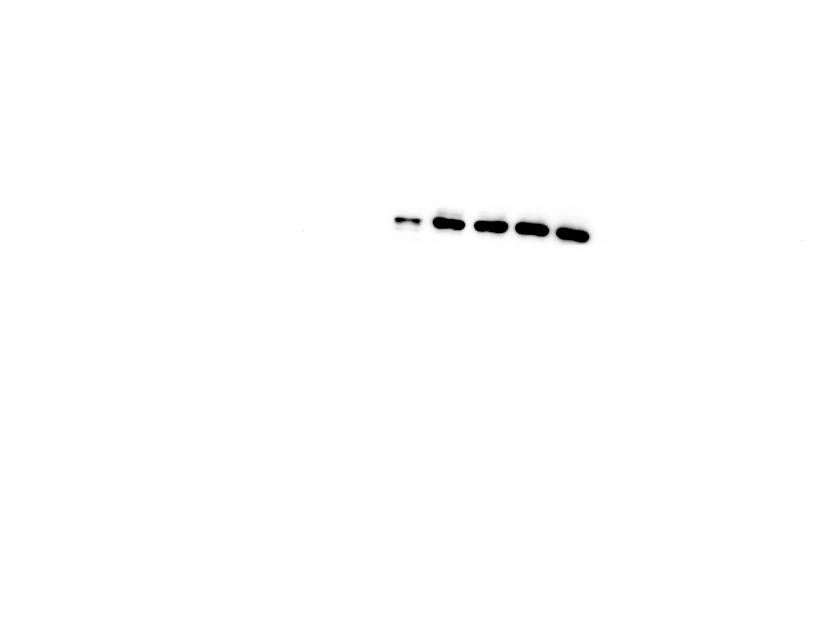

Supplement: Supplementary file 5 [file Data_Sheet_4.ZIP › Proteins for immune of compound 5/P-Ia╩Ba┴/contrast/contrast_4.png]

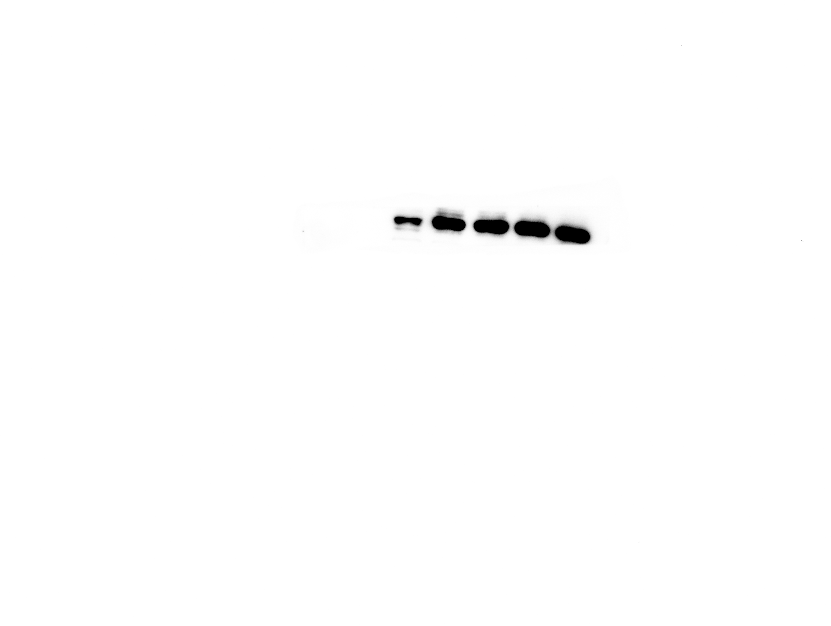

Supplement: Supplementary file 5 [file Data_Sheet_4.ZIP › Proteins for immune of compound 5/P-Ia╩Ba┴/contrast/contrast_5.png]

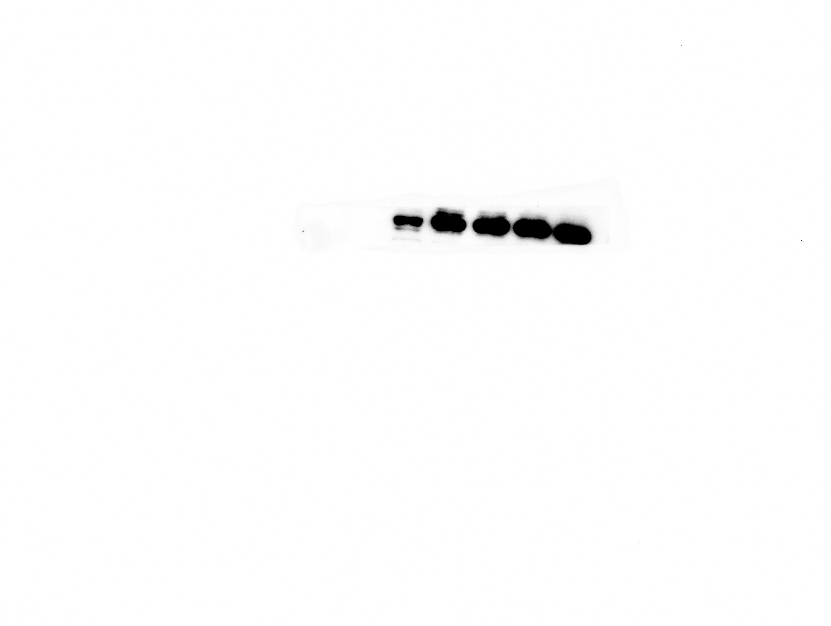

Supplement: Supplementary file 5 [file Data_Sheet_4.ZIP › Proteins for immune of compound 5/P-Ia╩Ba┴/contrast/contrast_6.png]

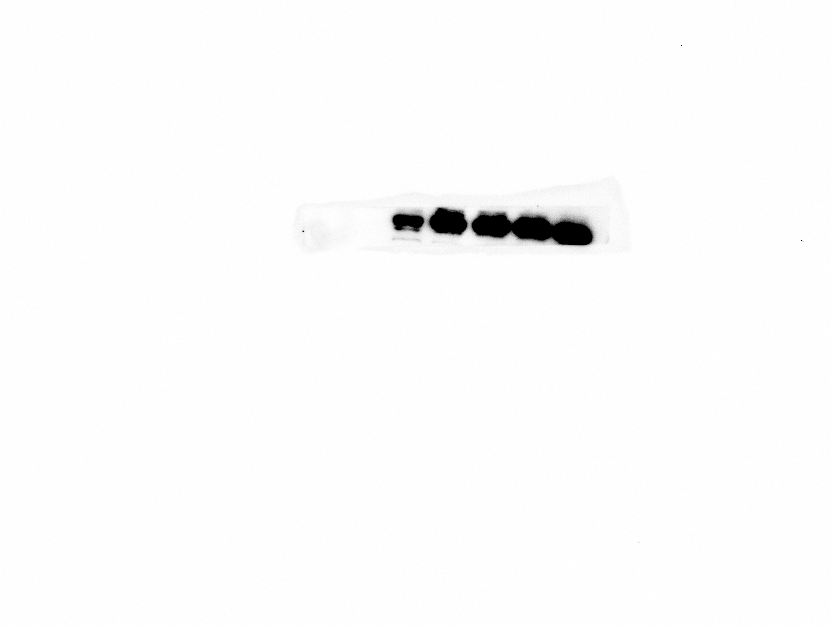

Supplement: Supplementary file 5 [file Data_Sheet_4.ZIP › Proteins for immune of compound 5/P-Ia╩Ba┴/contrast/contrast_7.png]

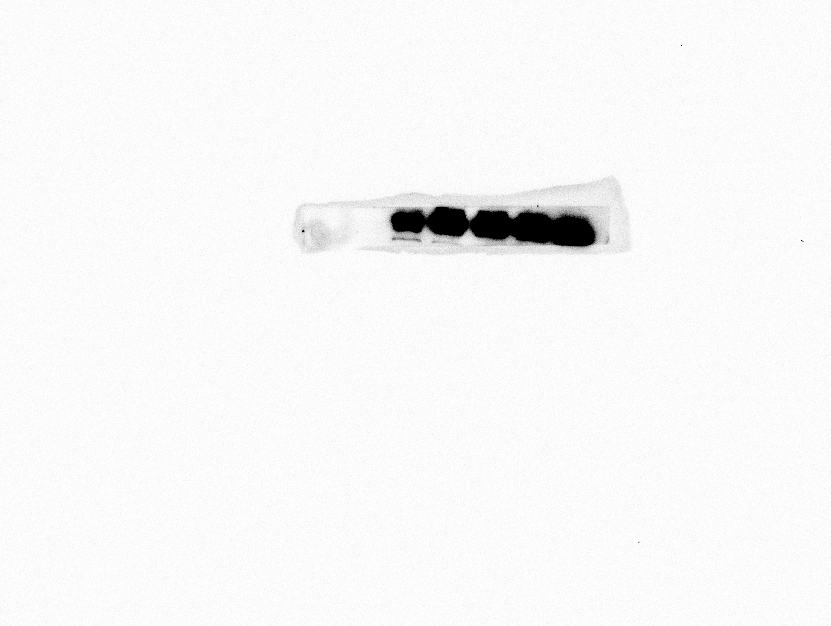

Supplement: Supplementary file 5 [file Data_Sheet_4.ZIP › Proteins for immune of compound 5/P-Ia╩Ba┴/contrast/contrast_8.png]

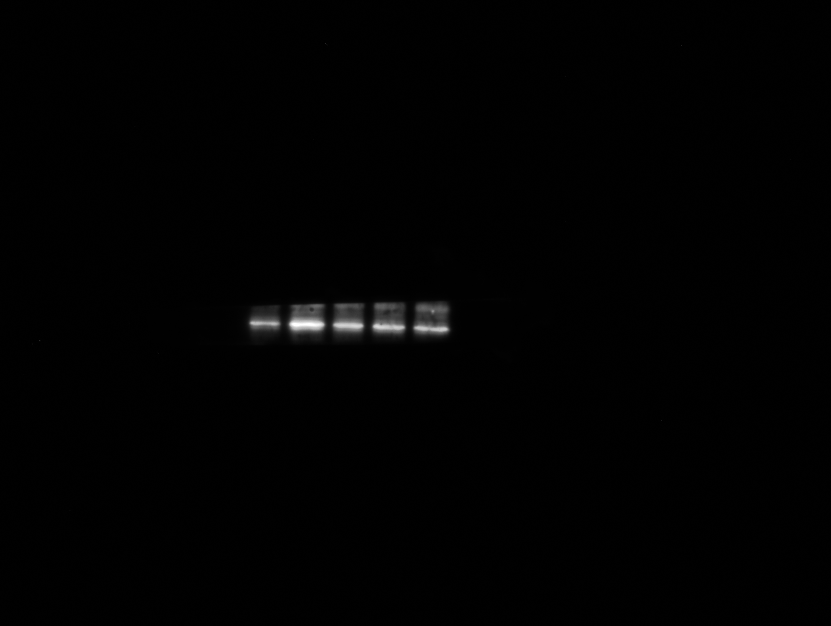

Supplement: Supplementary file 5 [file Data_Sheet_4.ZIP › Proteins for immune of compound 5/P-p65/2021-01-1 Pp65_1_16bit.png]

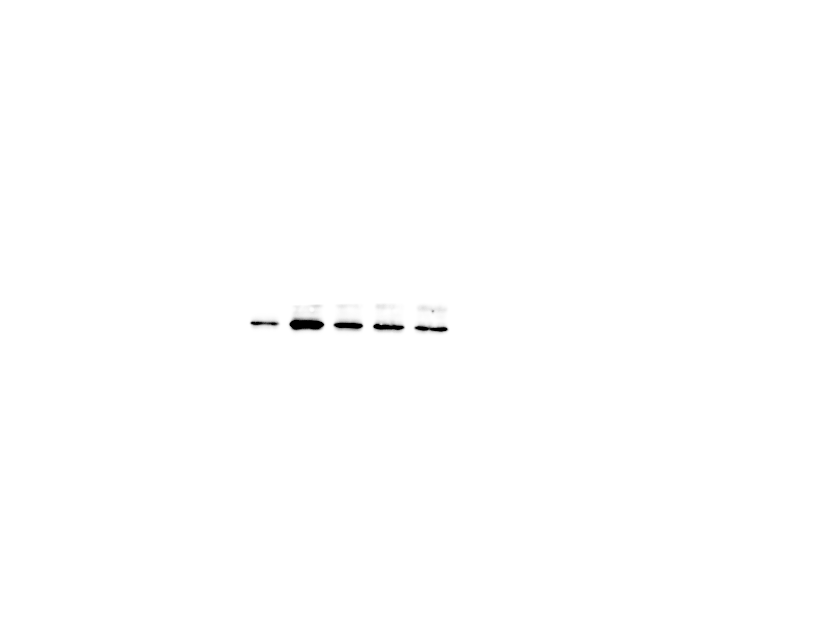

Supplement: Supplementary file 5 [file Data_Sheet_4.ZIP › Proteins for immune of compound 5/P-p65/2021-01-1 Pp65_8bit.png]

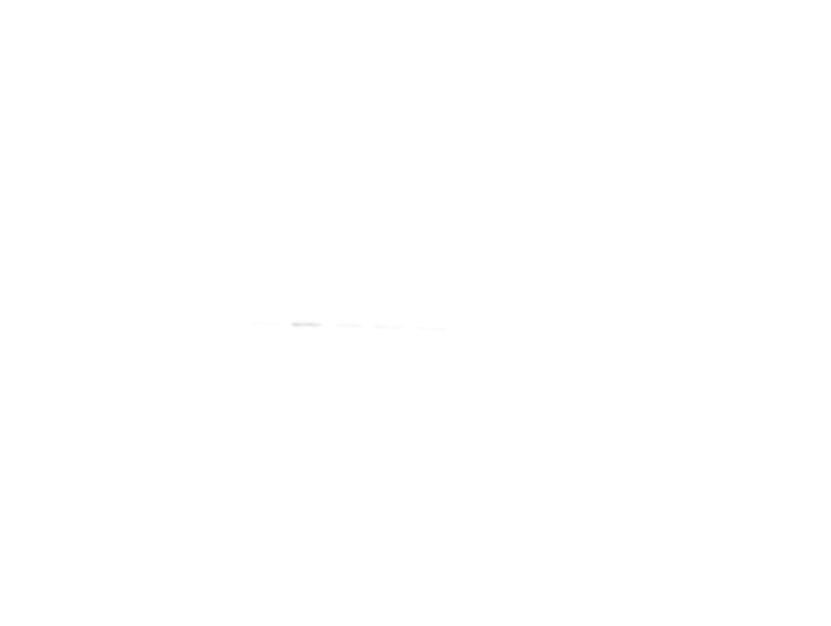

Supplement: Supplementary file 5 [file Data_Sheet_4.ZIP › Proteins for immune of compound 5/P-p65/contrast/contrast_0.png]

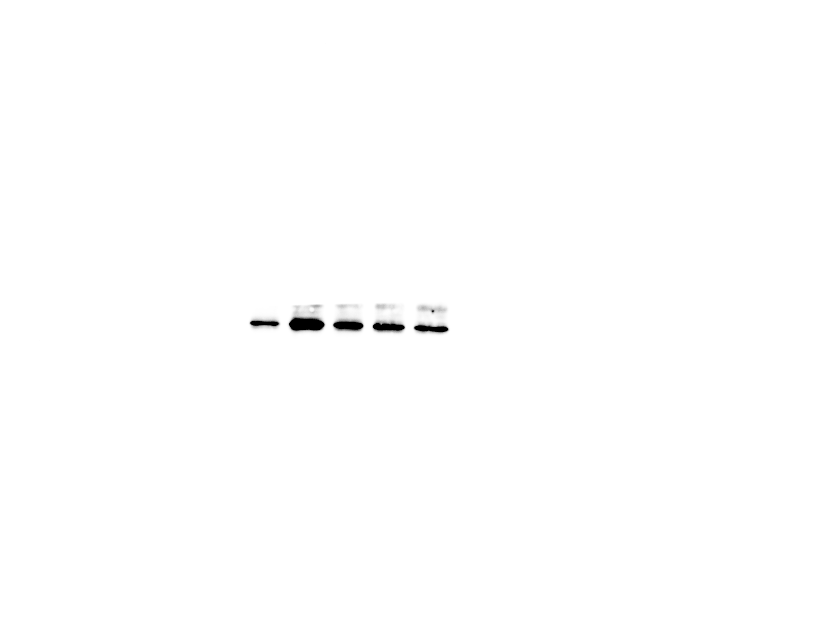

Supplement: Supplementary file 5 [file Data_Sheet_4.ZIP › Proteins for immune of compound 5/P-p65/contrast/contrast_2.png]

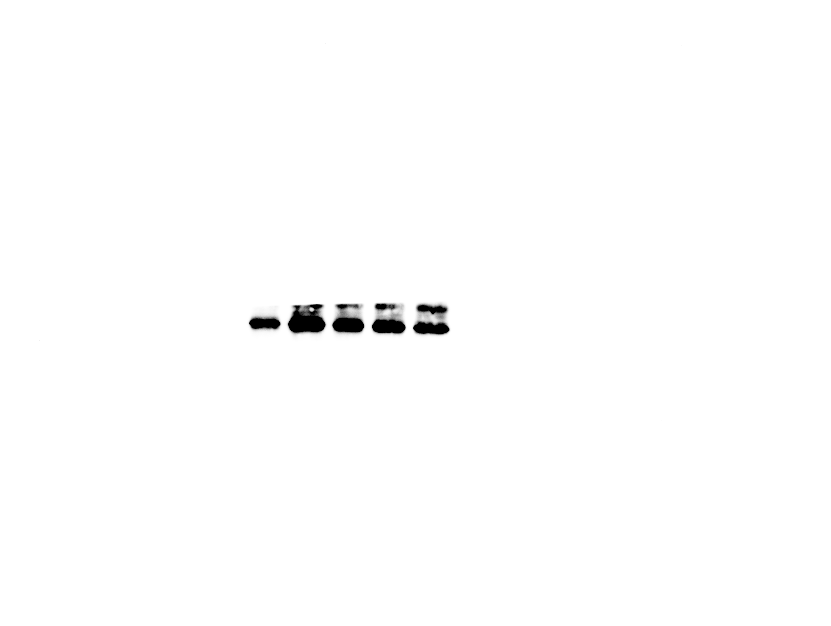

Supplement: Supplementary file 5 [file Data_Sheet_4.ZIP › Proteins for immune of compound 5/P-p65/contrast/contrast_3.png]

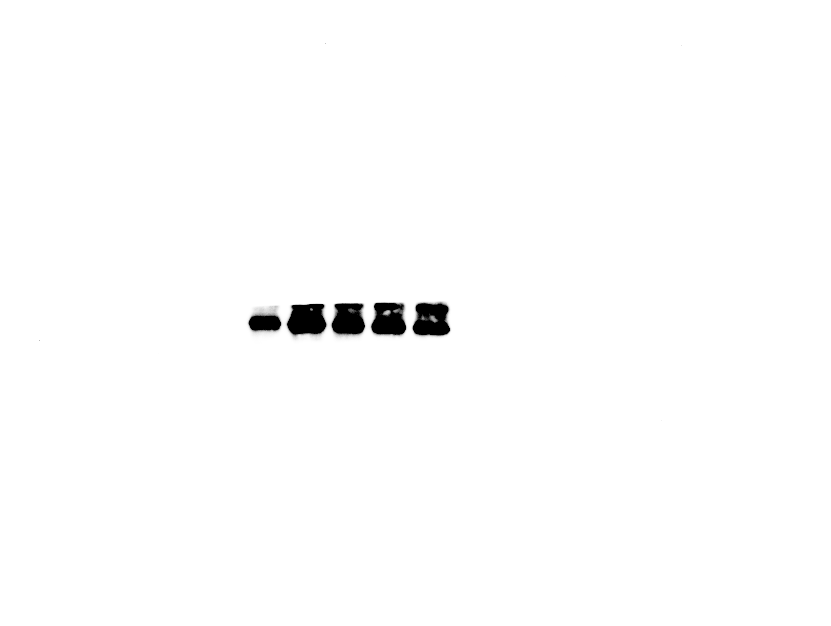

Supplement: Supplementary file 5 [file Data_Sheet_4.ZIP › Proteins for immune of compound 5/P-p65/contrast/contrast_4.png]

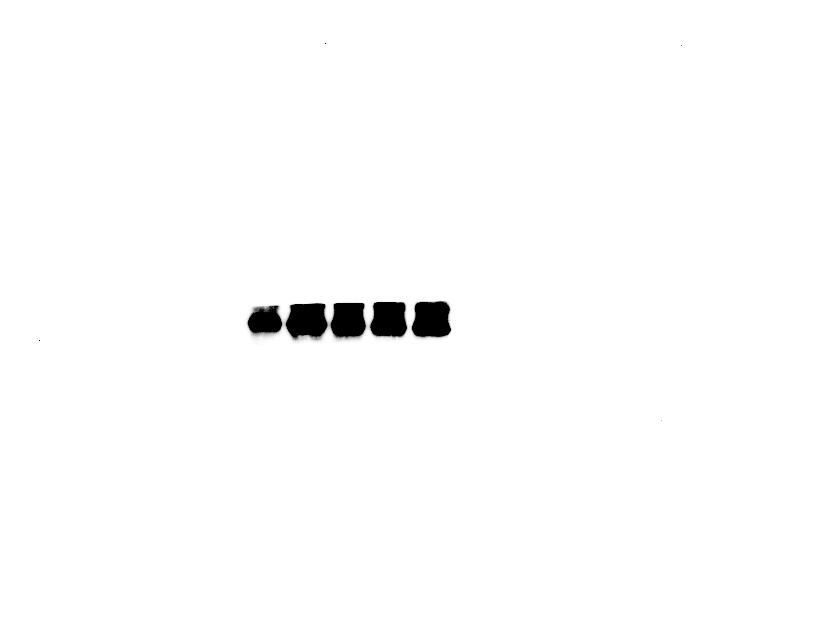

Supplement: Supplementary file 5 [file Data_Sheet_4.ZIP › Proteins for immune of compound 5/P-p65/contrast/contrast_5.png]

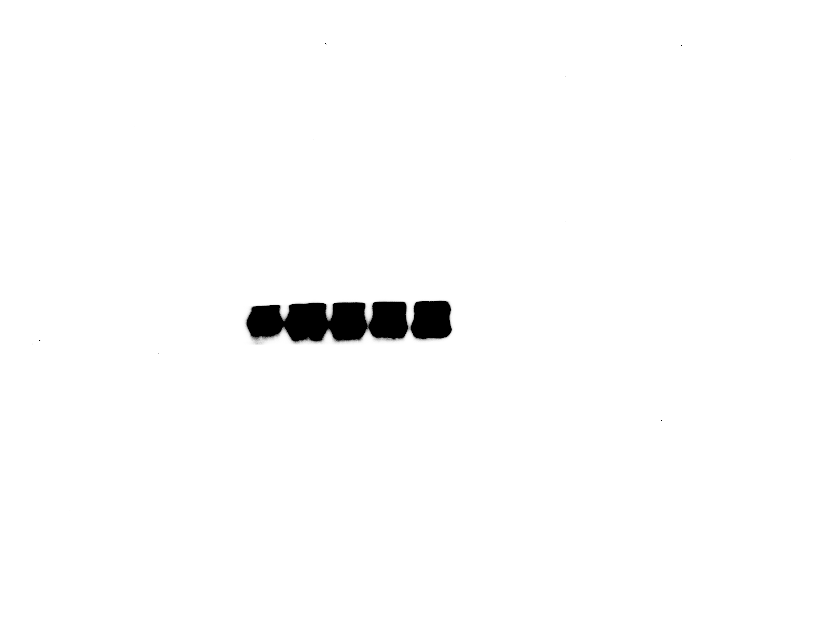

Supplement: Supplementary file 5 [file Data_Sheet_4.ZIP › Proteins for immune of compound 5/P-p65/contrast/contrast_6.png]

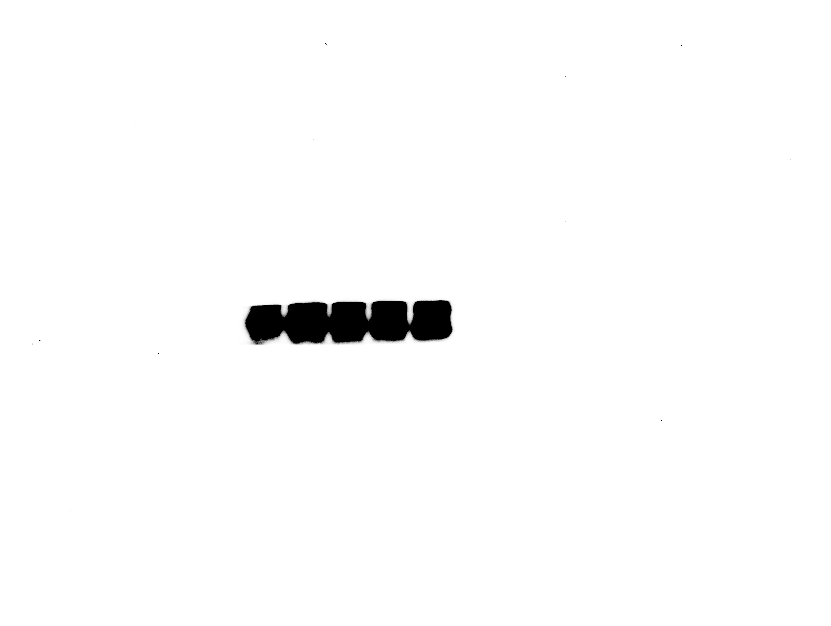

Supplement: Supplementary file 5 [file Data_Sheet_4.ZIP › Proteins for immune of compound 5/P-p65/contrast/contrast_7.png]

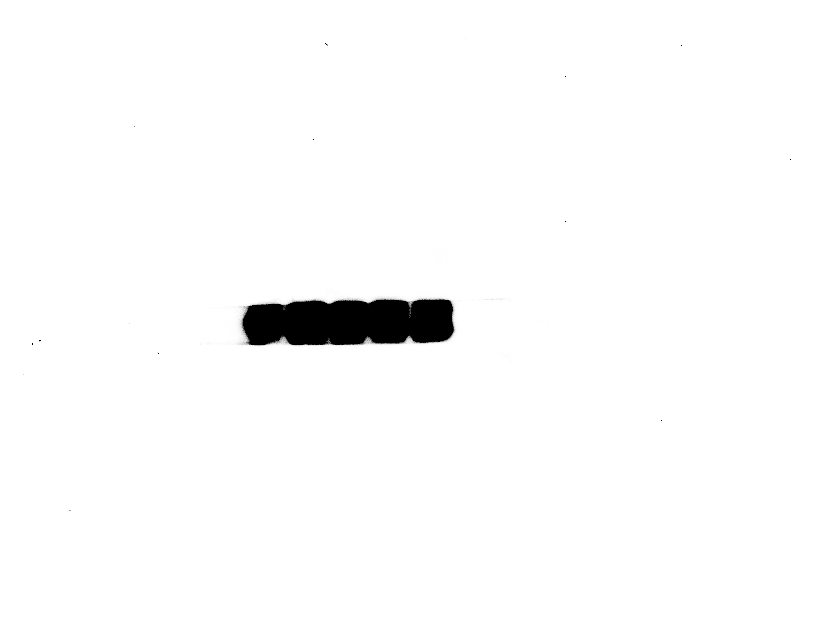

Supplement: Supplementary file 5 [file Data_Sheet_4.ZIP › Proteins for immune of compound 5/P-p65/contrast/contrast_8.png]

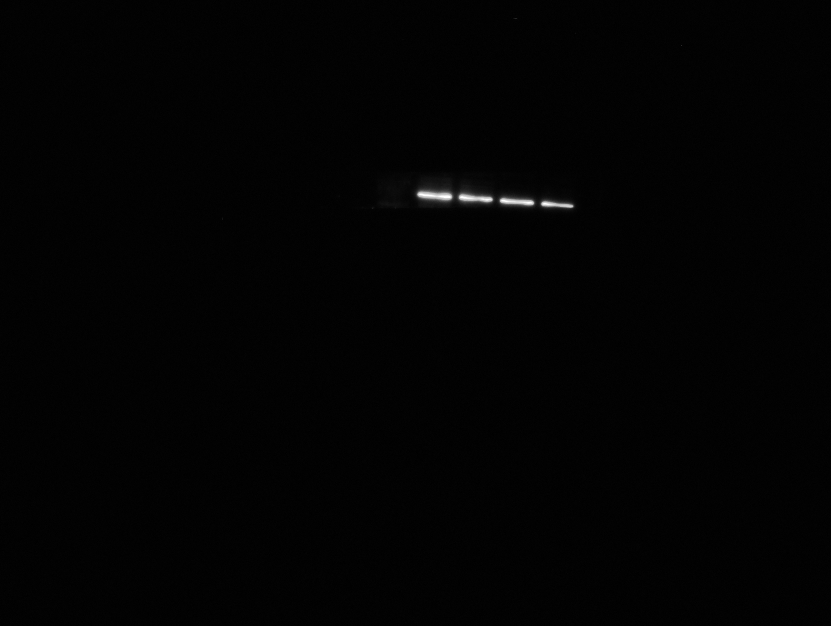

Supplement: Supplementary file 5 [file Data_Sheet_4.ZIP › Proteins for immune of compound 5/iNOS/2021-01-15_Inos_1_16bit.png]

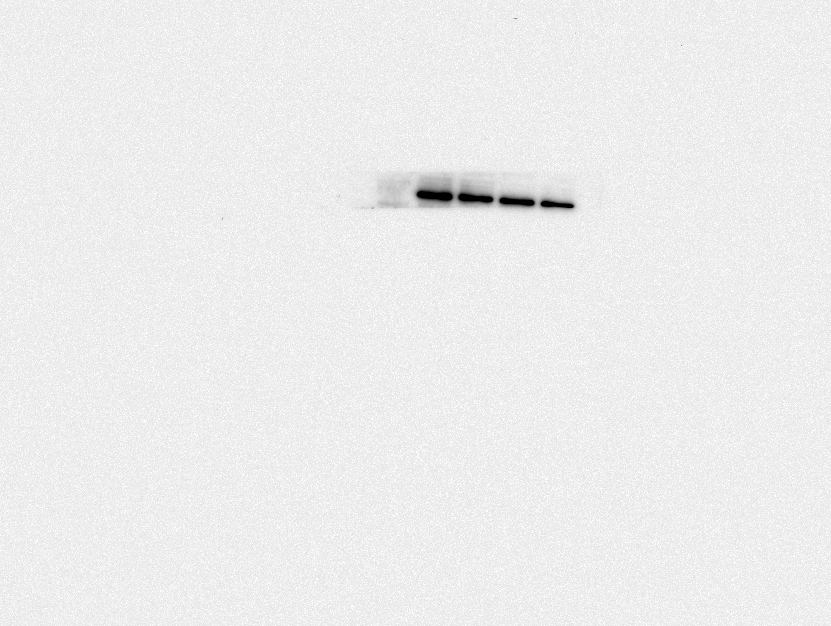

Supplement: Supplementary file 5 [file Data_Sheet_4.ZIP › Proteins for immune of compound 5/iNOS/2021-01-15_Inos_8bit.Tiff.png]

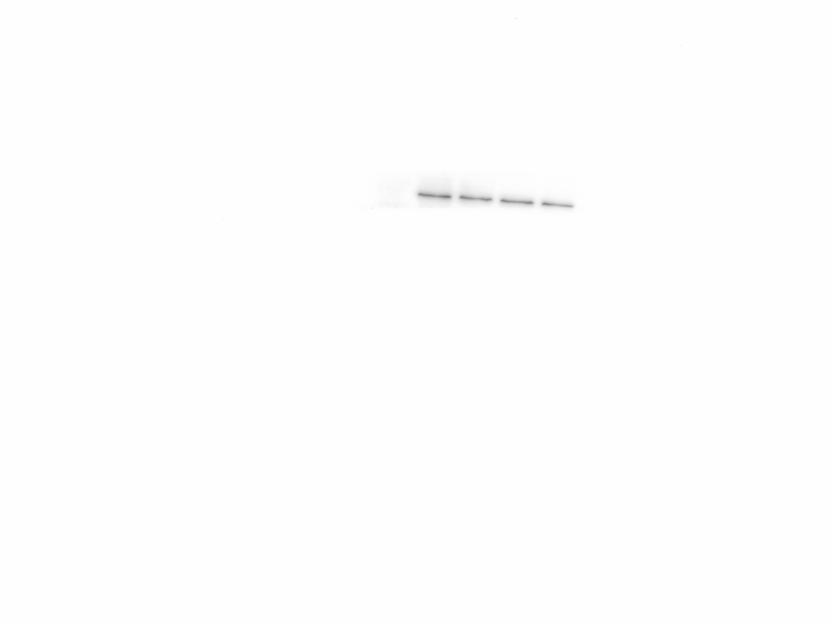

Supplement: Supplementary file 5 [file Data_Sheet_4.ZIP › Proteins for immune of compound 5/iNOS/contrast/contrast_0.png]

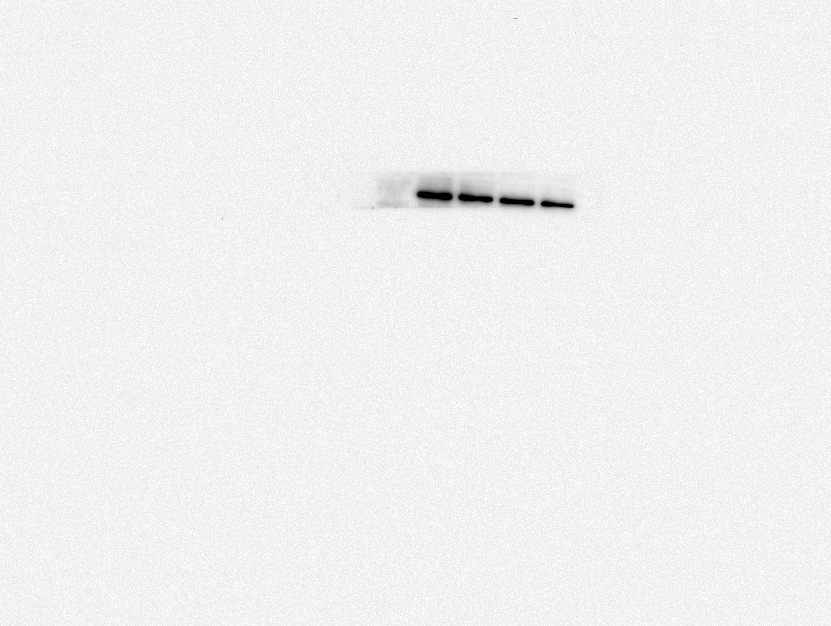

Supplement: Supplementary file 5 [file Data_Sheet_4.ZIP › Proteins for immune of compound 5/iNOS/contrast/contrast_1.png]

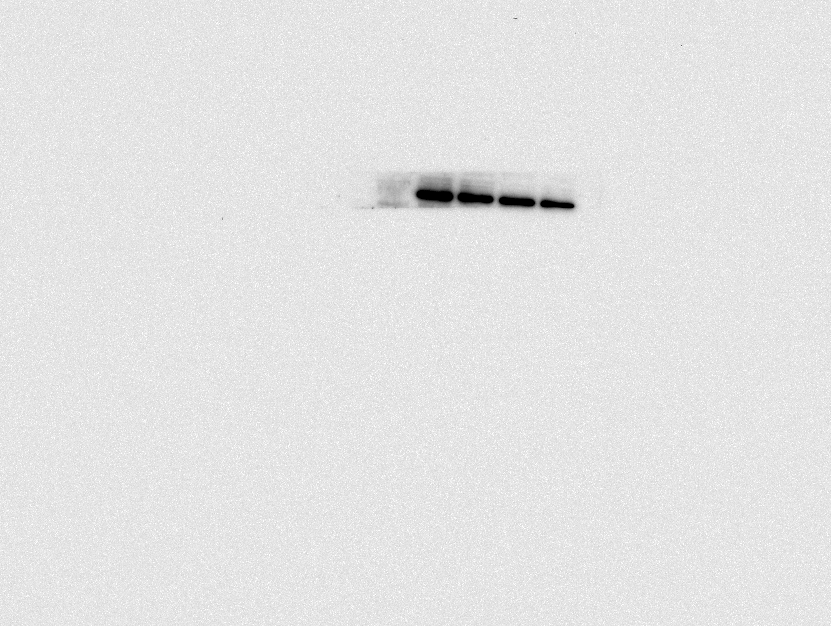

Supplement: Supplementary file 5 [file Data_Sheet_4.ZIP › Proteins for immune of compound 5/iNOS/contrast/contrast_3.png]

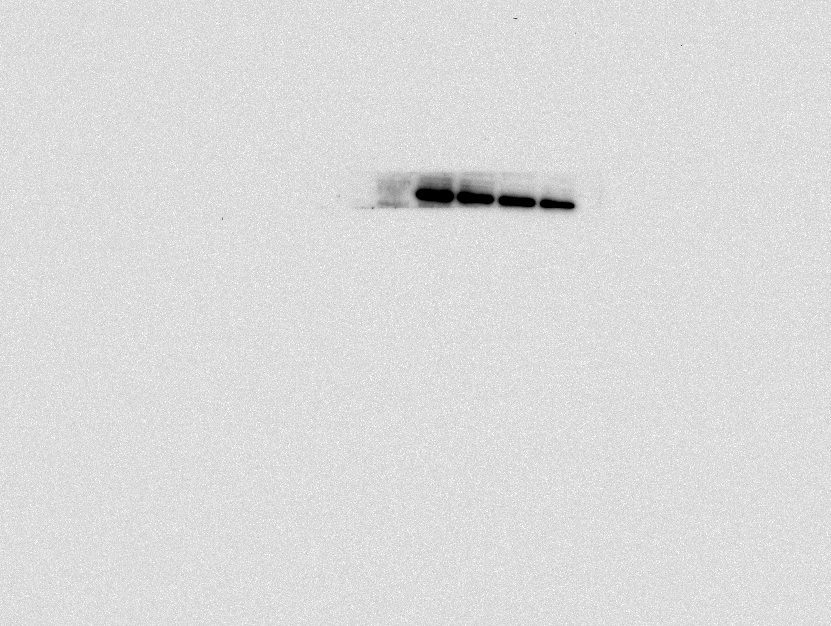

Supplement: Supplementary file 5 [file Data_Sheet_4.ZIP › Proteins for immune of compound 5/iNOS/contrast/contrast_4.png]

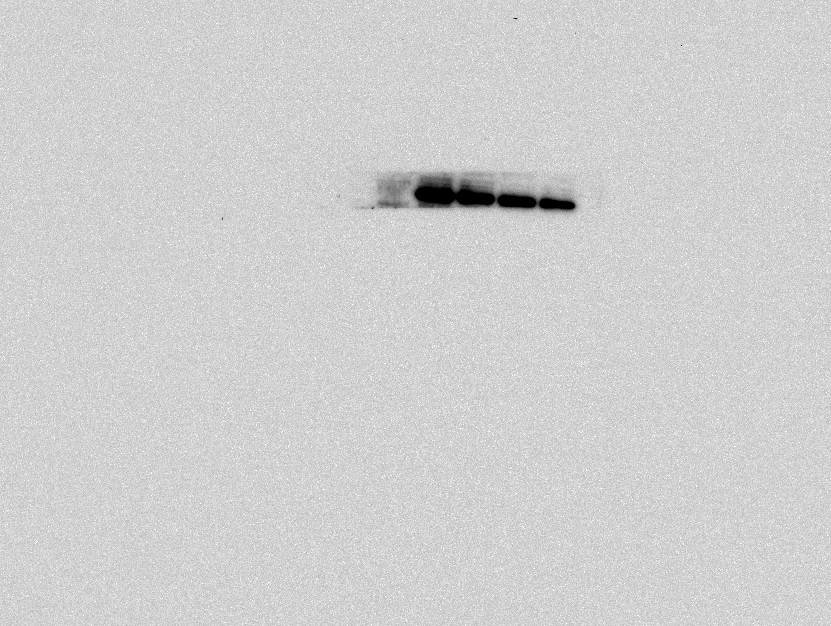

Supplement: Supplementary file 5 [file Data_Sheet_4.ZIP › Proteins for immune of compound 5/iNOS/contrast/contrast_5.png]

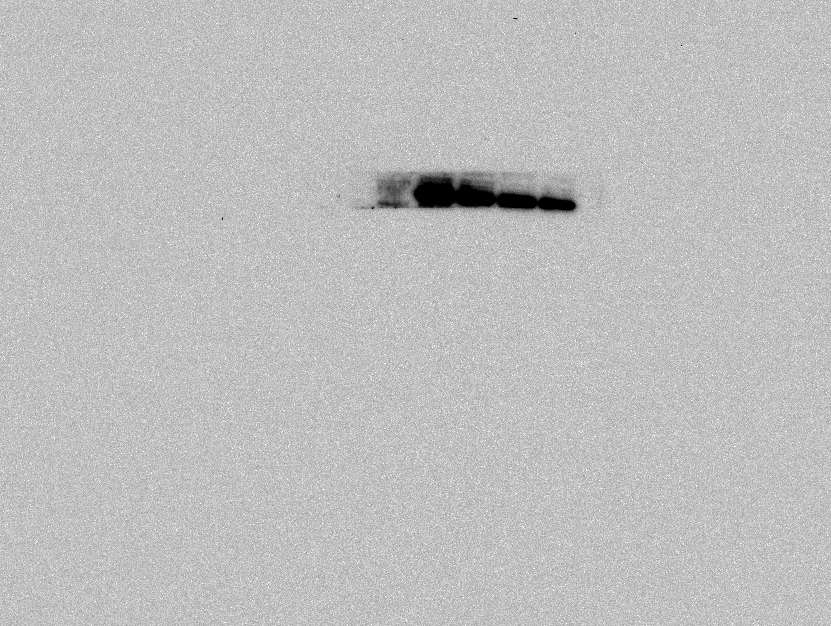

Supplement: Supplementary file 5 [file Data_Sheet_4.ZIP › Proteins for immune of compound 5/iNOS/contrast/contrast_6.png]

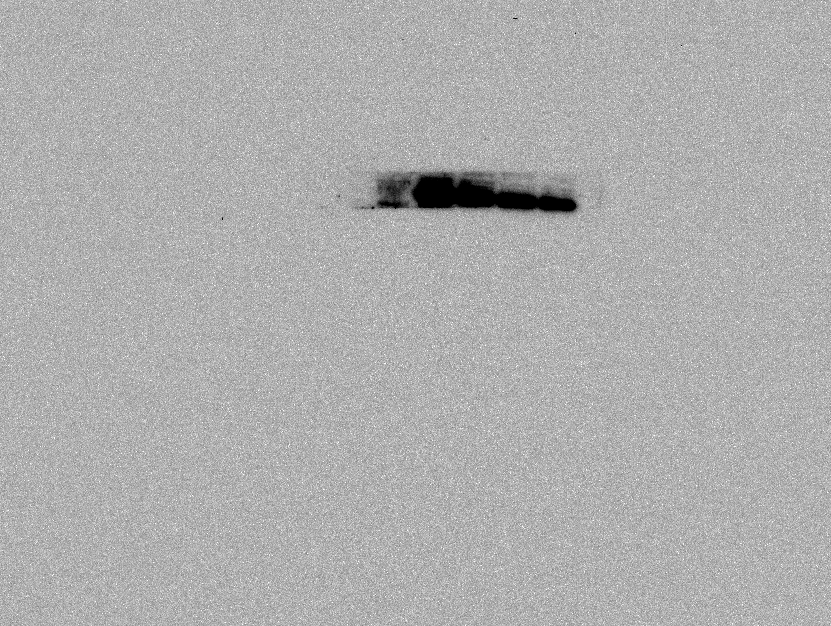

Supplement: Supplementary file 5 [file Data_Sheet_4.ZIP › Proteins for immune of compound 5/iNOS/contrast/contrast_7.png]

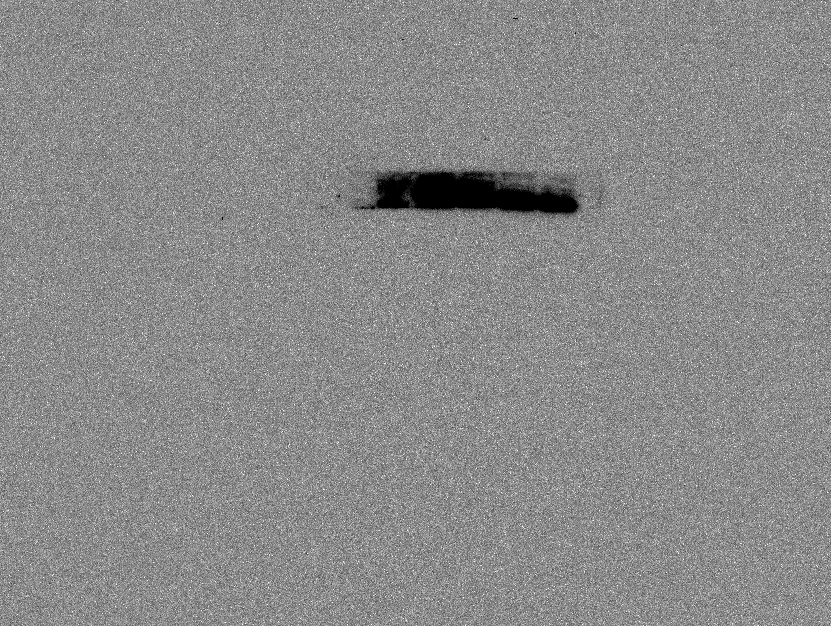

Supplement: Supplementary file 5 [file Data_Sheet_4.ZIP › Proteins for immune of compound 5/iNOS/contrast/contrast_8.png]

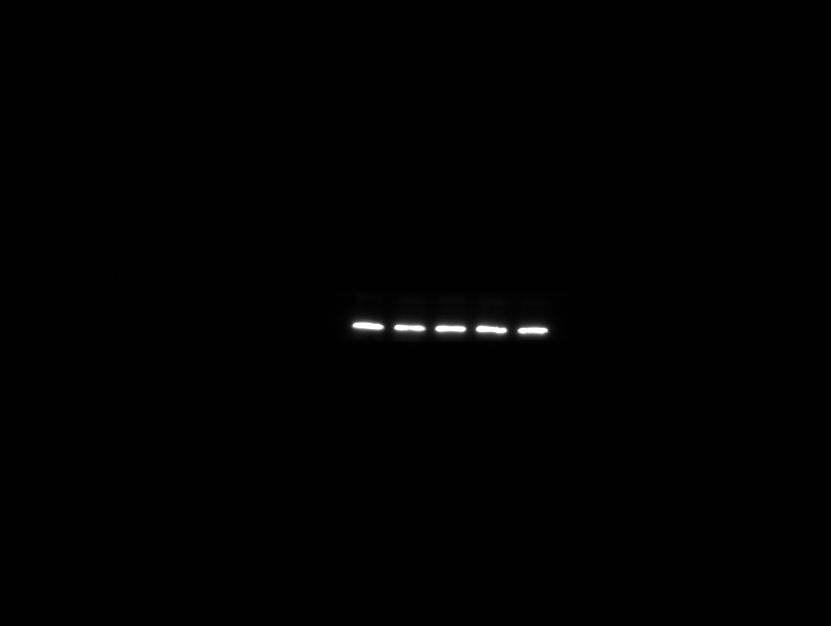

Supplement: Supplementary file 5 [file Data_Sheet_4.ZIP › Proteins for immune of compound 5/p65/2021-01-13_P65_1_16bit.png]

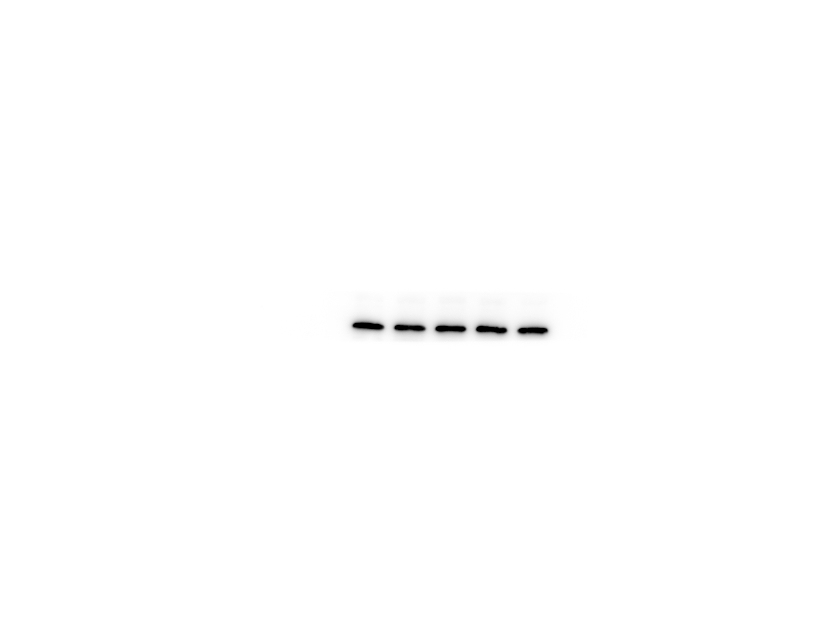

Supplement: Supplementary file 5 [file Data_Sheet_4.ZIP › Proteins for immune of compound 5/p65/2021-01-13_P65_8bit.png]

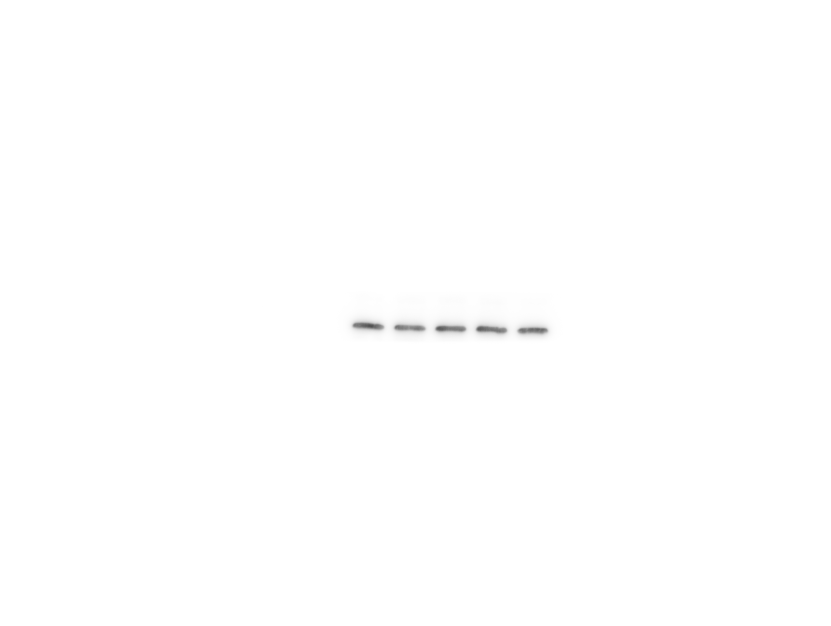

Supplement: Supplementary file 5 [file Data_Sheet_4.ZIP › Proteins for immune of compound 5/p65/contrast/contrast_0.png]

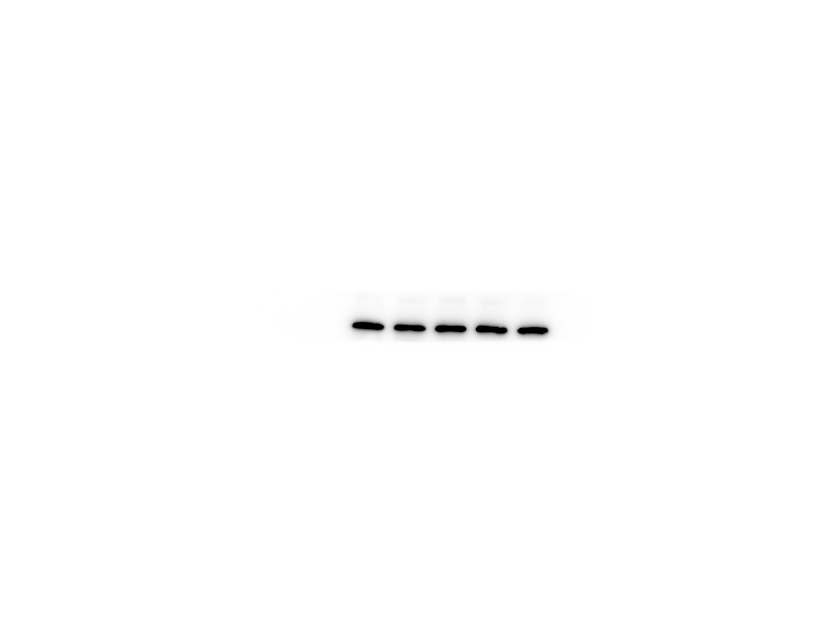

Supplement: Supplementary file 5 [file Data_Sheet_4.ZIP › Proteins for immune of compound 5/p65/contrast/contrast_2.png]

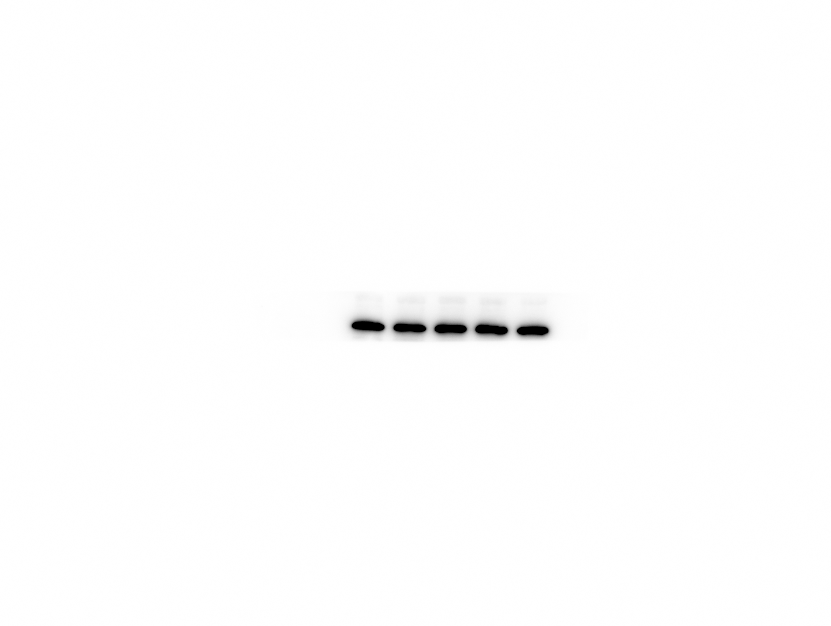

Supplement: Supplementary file 5 [file Data_Sheet_4.ZIP › Proteins for immune of compound 5/p65/contrast/contrast_3.png]

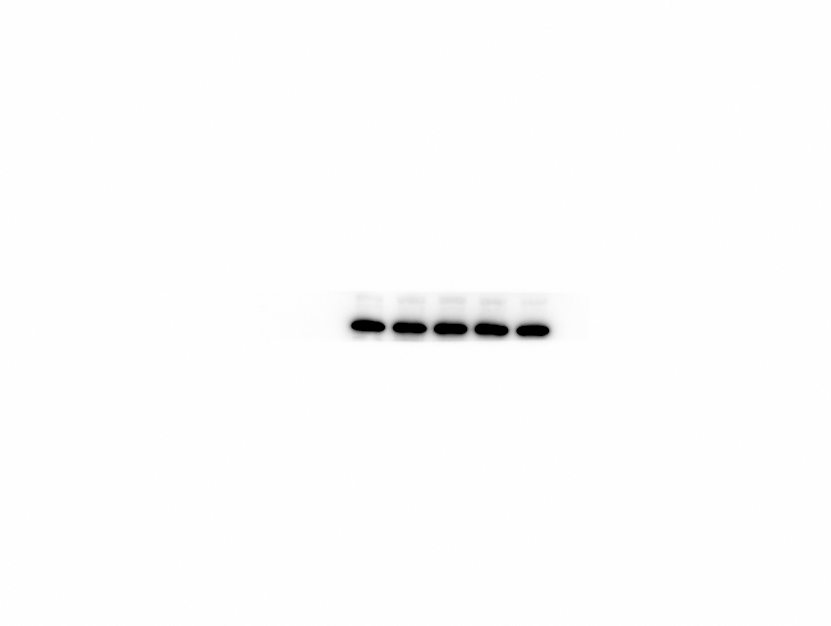

Supplement: Supplementary file 5 [file Data_Sheet_4.ZIP › Proteins for immune of compound 5/p65/contrast/contrast_4.png]

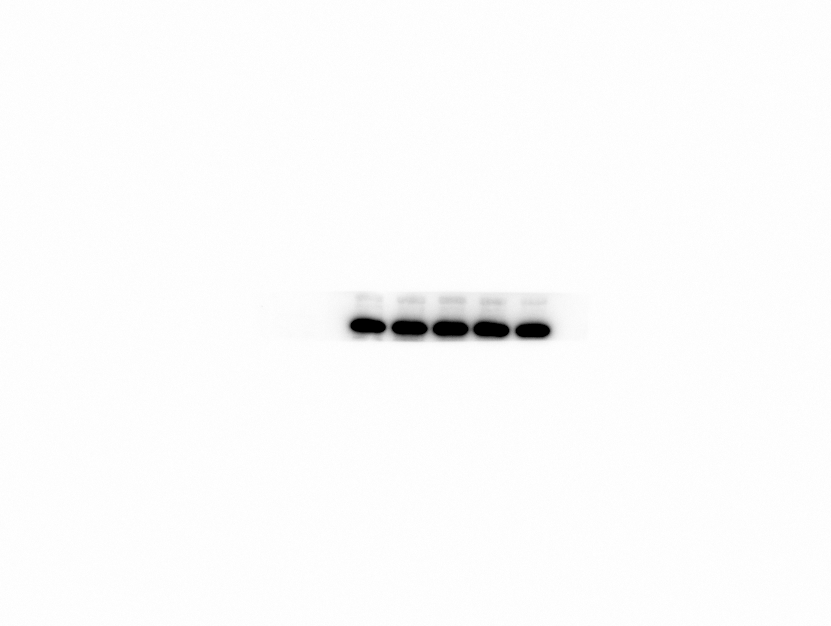

Supplement: Supplementary file 5 [file Data_Sheet_4.ZIP › Proteins for immune of compound 5/p65/contrast/contrast_5.png]

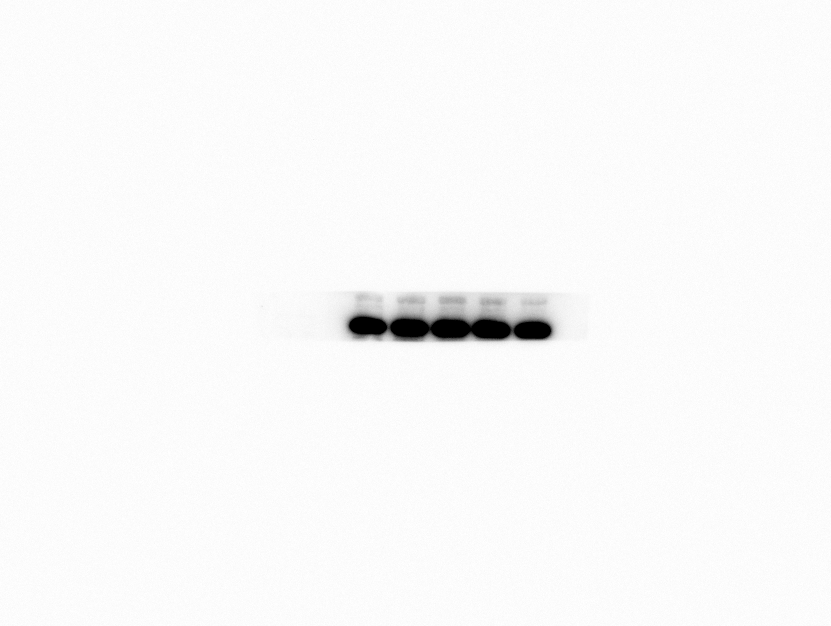

Supplement: Supplementary file 5 [file Data_Sheet_4.ZIP › Proteins for immune of compound 5/p65/contrast/contrast_6.png]

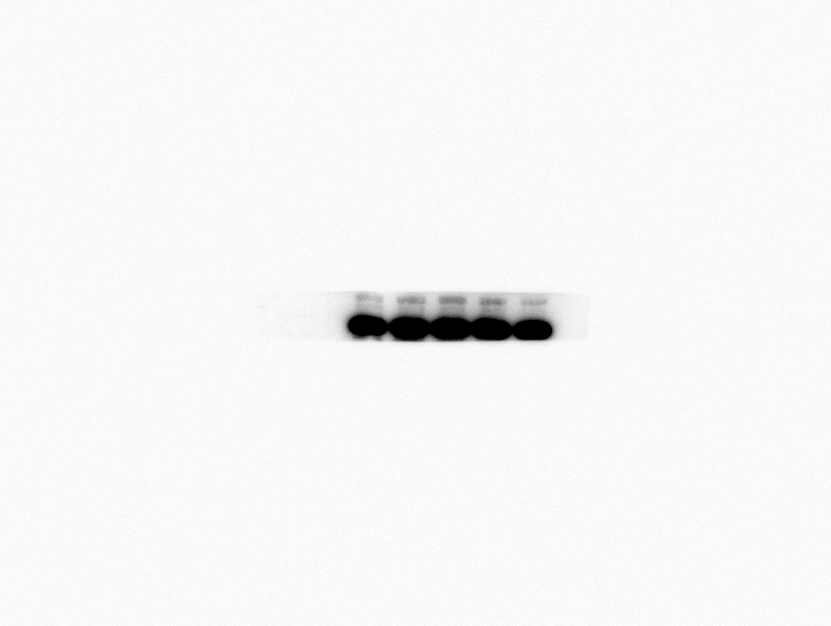

Supplement: Supplementary file 5 [file Data_Sheet_4.ZIP › Proteins for immune of compound 5/p65/contrast/contrast_7.png]

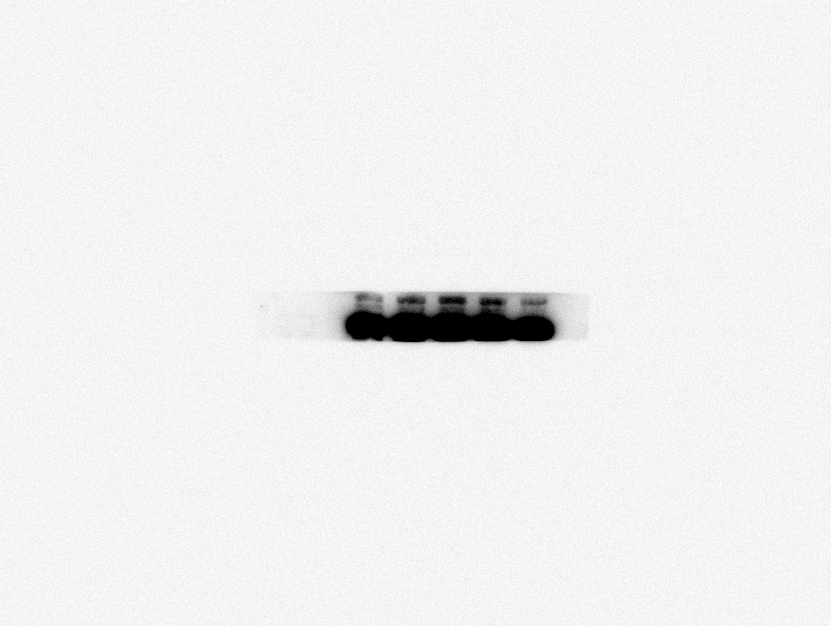

Supplement: Supplementary file 5 [file Data_Sheet_4.ZIP › Proteins for immune of compound 5/p65/contrast/contrast_8.png]

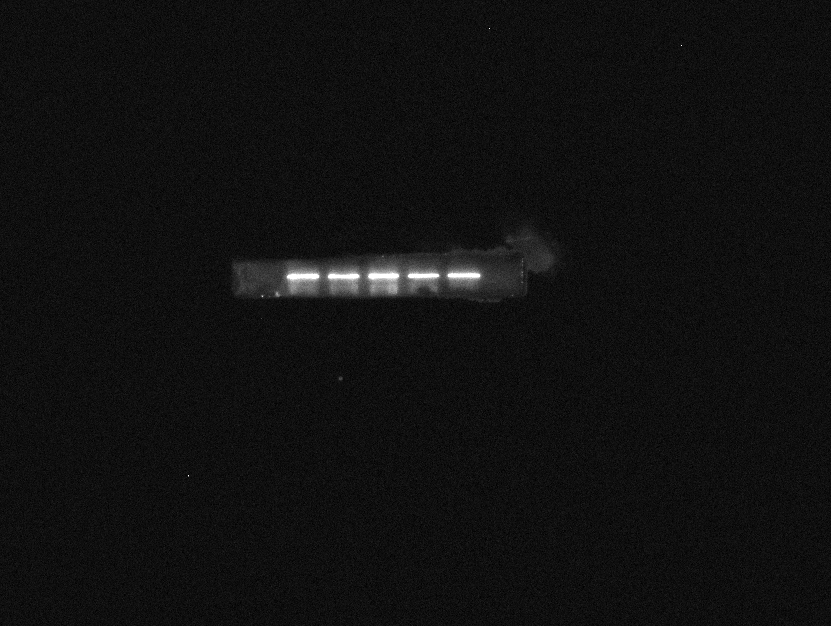

Supplement: Supplementary file 6 [file Data_Sheet_5.ZIP › Proteins for immune of compound 8/2021-01-16_GAPDH/2021-01-16_GAPDH_1_16bit.png]

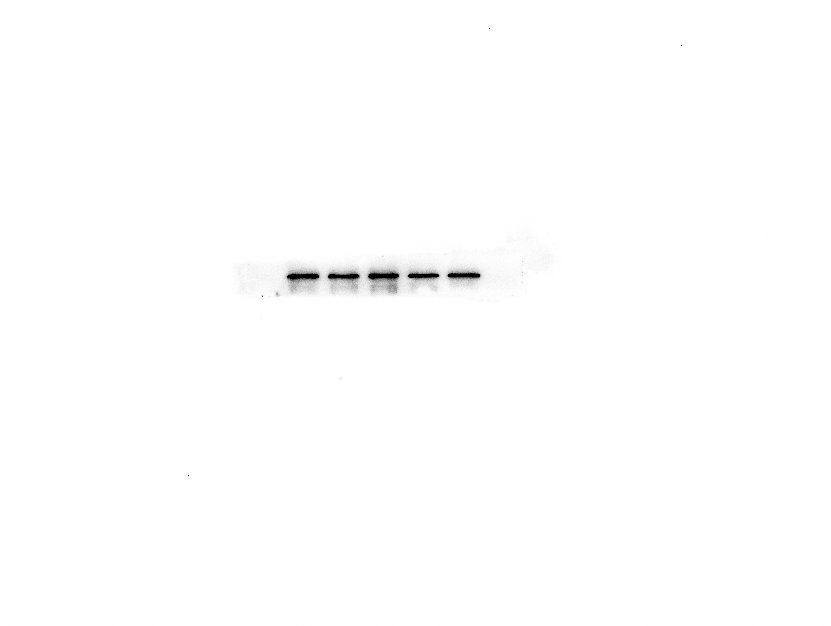

Supplement: Supplementary file 6 [file Data_Sheet_5.ZIP › Proteins for immune of compound 8/2021-01-16_GAPDH/2021-01-16_GAPDH_8bit.png]

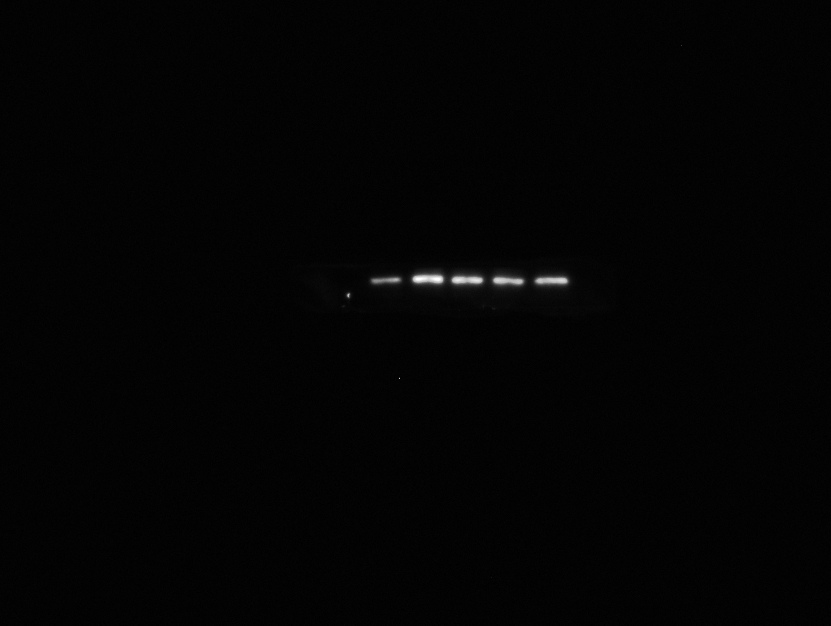

Supplement: Supplementary file 6 [file Data_Sheet_5.ZIP › Proteins for immune of compound 8/P-Ia╩Ba┴/2021-01-15_Pikba2_1_16bit.png]

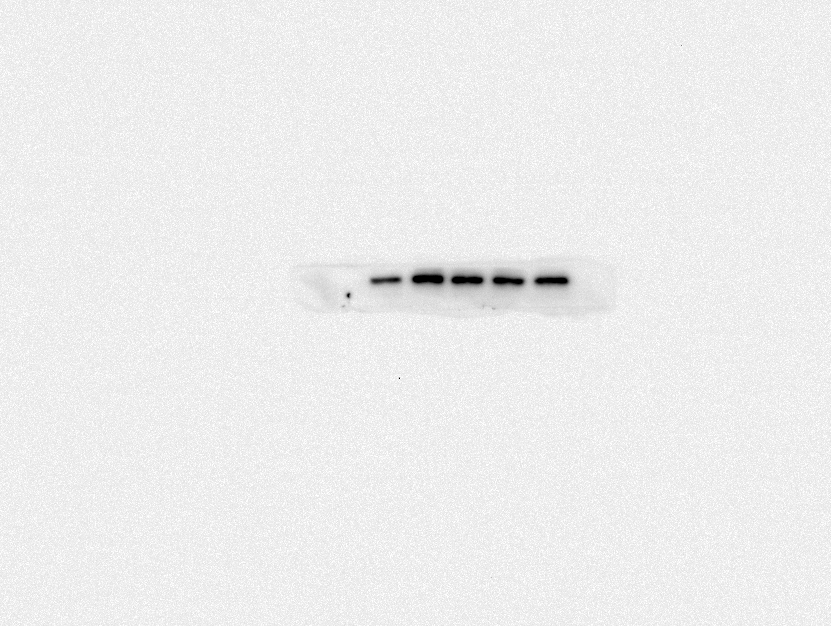

Supplement: Supplementary file 6 [file Data_Sheet_5.ZIP › Proteins for immune of compound 8/P-Ia╩Ba┴/2021-01-15_Pikba2_8bit.png]

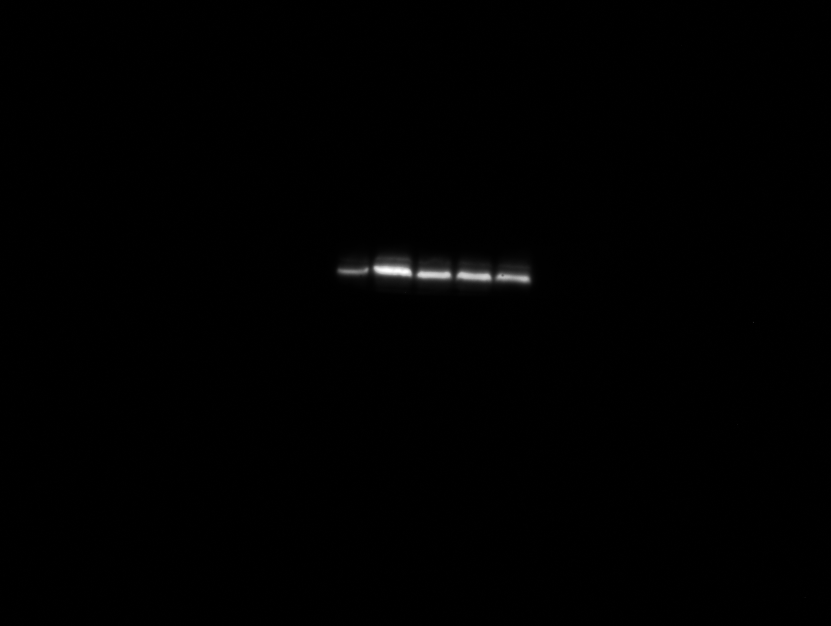

Supplement: Supplementary file 6 [file Data_Sheet_5.ZIP › Proteins for immune of compound 8/P-p65/2020-12-26_Pp65_1_16bit.png]

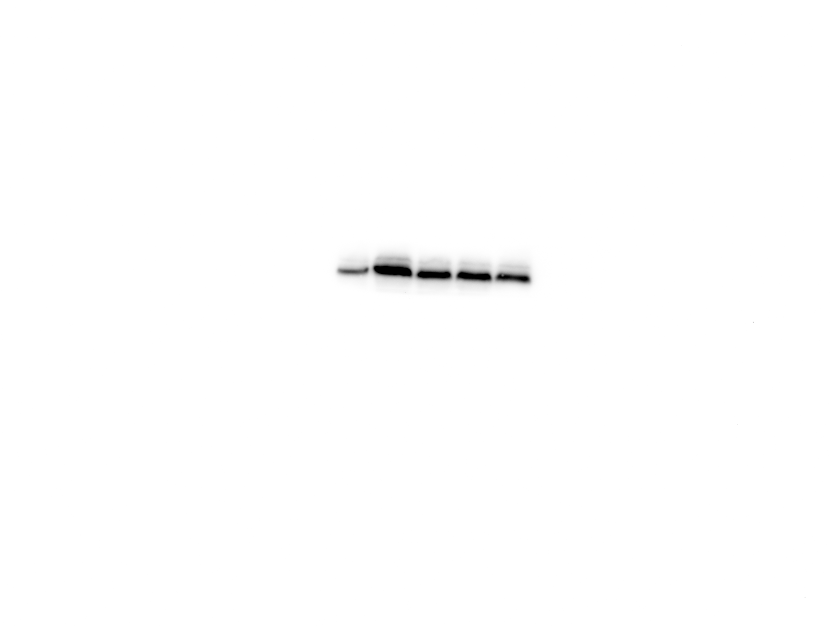

Supplement: Supplementary file 6 [file Data_Sheet_5.ZIP › Proteins for immune of compound 8/P-p65/2020-12-26_Pp65_8bit.png]

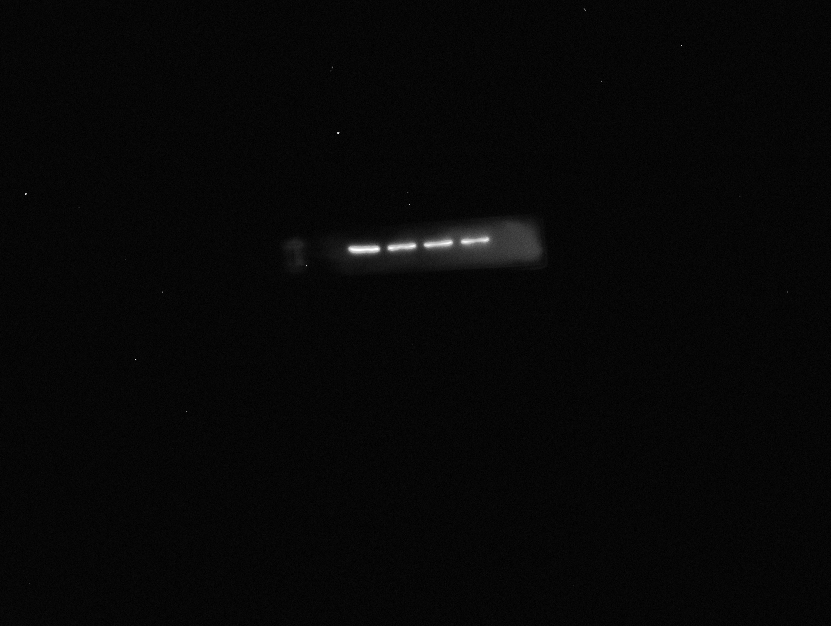

Supplement: Supplementary file 6 [file Data_Sheet_5.ZIP › Proteins for immune of compound 8/iNOS/2021-Inosc7_1_16bit.png]

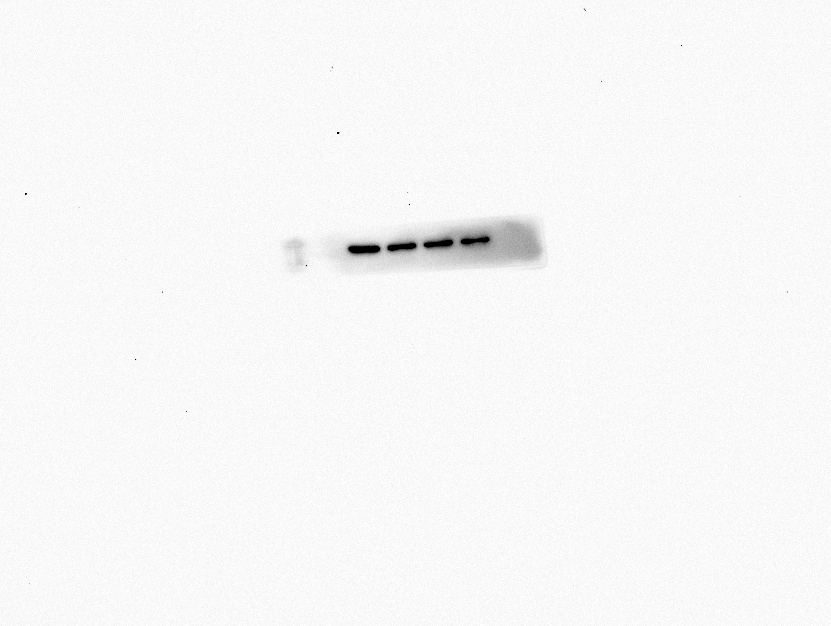

Supplement: Supplementary file 6 [file Data_Sheet_5.ZIP › Proteins for immune of compound 8/iNOS/2021-Inosc7_8bit.png]
