# Supplementary figures and images for: Nigella sativa: A Dietary Supplement as an Immune-Modulator on the Basis of Bioactive Components (part 3 of 3)
Source: Front Nutr. 2021 Aug 17;8:722813. doi: 10.3389/fnut.2021.722813 (PMC8415885; doi:10.3389/fnut.2021.722813)

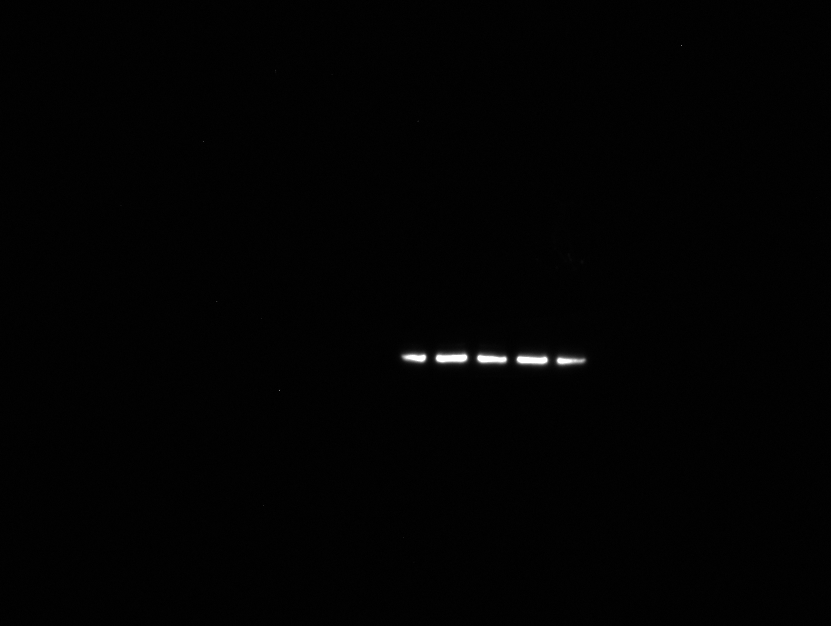

Supplement: Supplementary file 6 [file Data_Sheet_5.ZIP › Proteins for immune of compound 8/p65/2020-12-26_15-37-00_1_16bit.png]

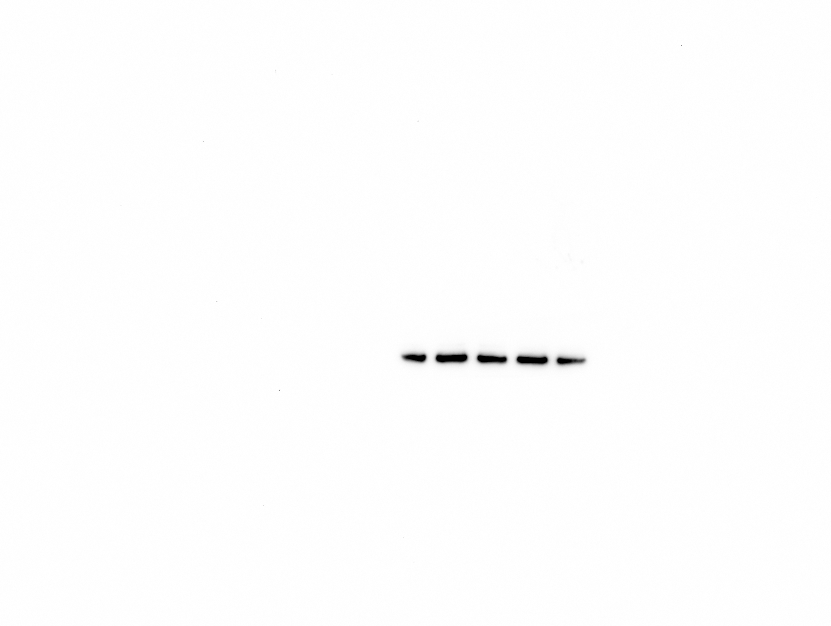

Supplement: Supplementary file 6 [file Data_Sheet_5.ZIP › Proteins for immune of compound 8/p65/2020-12-26_15-37-00_8bit.png]
